# NIDO, AMOP and vWD domains of MUC4 play synergic role in MUC4 mediated signaling

**DOI:** 10.18632/oncotarget.14420

**Published:** 2017-01-02

**Authors:** Yi Zhu, Jing-Jing Zhang, Yun-Peng Peng, Xian Liu, Kun-Ling Xie, Jie Tang, Kui-Rong Jiang, Wen-Tao Gao, Lei Tian, Kai Zhang, Ze-Kuan Xu, Yi Miao

**Affiliations:** ^1^ Pancreas Institute of Nanjing Medical University, Nanjing, People’s Republic of China; ^2^ Pancreas Center, The First Affiliated Hospital of Nanjing Medical University, Nanjing, People’s Republic of China; ^3^ Department of General Surgery, The First Affiliated Hospital of Nanjing Medical University, Nanjing, People’s Republic of China; ^4^ Department of General Surgery, The People’s Hospital of Bozhou, Bozhou, Anhui, People’s Republic of China; _5_Department of Pediatric Surgery, Nanjing Children’s Hospital Affiliated to Nanjing Medical University, Nanjing, People’s Republic of China

**Keywords:** NIDO, AMOP, vWD, synergy, MUC4/Y

## Abstract

MUC4 mucin is well known as an important potential target to overcome pancreatic cancer. Three unique domains (NIDO, AMOP, and vWD) with unclear roles only present in MUC4 but are not found in other membrane-bound mucins. Our previous studies first reported that its splice variant, *MUC4/Y* can be a model of MUC4 (MUC4 gene fragment is more than 30KB, too huge to clone and eukaryotic express) in pancreatic cancer. More importantly, based on *MUC4/Y* with the appropriate length of gene sequence, it is easy to construct the unique domain-lacking models of *MUC4/Y* (MUC4) for research. The present study focuses on investigation of the respective role of the unique NIDO, AMOP, and vWD domain or their synergistic effect on MUC4(*MUC4/Y*)-mediated functions and mechanisms by series of *in vitro* assays, sequence-based transcriptome analysis, validation of qRT-PCR & Western blot, and systematic comparative analysis. Our results demonstrate: 1) NIDO, AMOP, and vWD domain or their synergy play significant roles on *MUC4/Y*-mediated malignant function of pancreatic cancer, downstream of molecule mechanisms, particularly *MUC4/Y*-triggered malignancy-related positive feedback loops, respectively. 2) The synergistic roles of three unique domains on *MUC4/Y*-mediated functions and mechanisms are more prominent than the respective domain because the synergy of three domain plays the more remarkable effects on *MUC4/Y*-mediated signaling hub. Thus, to improve reversed effects of domain-lacking and break the synergism of domains will contribute to block *MUC4/Y*(MUC4) triggering various oncogenic signaling pathways.

## INTRODUCTION

As a high-molecular-weight member of the transmembrane mucin family [1, 2], MUC4 mucin plays important roles in the carcinogenesis and malignant progression of human pancreatic cancer [1, 3–5]. MUC4 is not expressed on in normal parts of the pancreas, but aberrantly overexpressed in pancreatic ductal adenocarcinoma (PDAC) and precancerous pancreatic intraepithelial neoplasias(PanIN) [6–9]. The levels of MUC4 expression rise consistently with the PanIN-PDAC progression model, and correlate significantly with poor prognosis of PDAC [7, 9, 10]. As our and other studies in the literature have reported, pancreatic cancer cells can exploit the multi-functions of MUC4 to trigger malignant activities including proliferation [11, 12, 14, 16], resistance to apoptosis [14, 16], motility [16], invasiveness & neural invasion [15, 16, 17], angiogenesis [12, 16], metastasis [11–13, 15, 16], supressing immune [18], and chemoresistance [19–21]. Thus MUC4 is an important potential target to overcome pancreatic cancer.

MUC4 has been mapped to chromosome 3 in the q29 region [22], which was cloned from the human tracheobronchial chromosomal DNA library and a human pancreatic tumor cell line [2, 23, 24]. The full-length MUC4 gene (*abbr*. FL-MUC4. NCBI Reference Sequence: NM_018406.6) contains 26 exons, which encode various functional domains from the amino end to carboxyl end, in order as follows: a 27 residue signal peptide, serine & threonine-enriched imperfect repetition motifs, a centrally located large tandem repeat (TR) domain, nidogen (NIDO)-like domain, adhesion-associated domain (AMOP; present in MUC4 and other proteins), von Willebrand factor (vWD; type D domain), and three EGF-like domains [2, 22–25], hydrophobic transmembrane region by which MUC4 is anchored to the cell surface, followed by a short cytoplasmic tail of 22 amino acids. Among them, it has been proved that the EGF-like domains present in MUC4 interact with ErbB2 and ErbB3 receptors to trigger intrinsic protein–tyrosine kinase activity and further activate intracellular signaling pathways (e.g., mitogen-activated protein kinase [MAPK], phosphatidylinositol-3-kinase [PI3K]–Akt, protein kinase C [PKC] pathways), and coactivate transcription of the downstream effector molecules to mediate malignant functions of tumor, including pancratic cancer [15, 16, 26–30]. Notably, the unique domains present in the MUC4 mucin but not found in other membrane-bound mucins are NIDO, AMOP, and vWD domains [31]. A homology analysis between MUC4 proteins in different vertebrate species reveals NIDO, AMOP, and vWD domains are conservative and important motifs [3, 23]. However, the role or effect of the three unique domains on MUC4-mediated functions and mechanisms is unclear, especially when in different tumor microenvironment.

In our previous studies [14, 16], we first proved that its splice variant, *MUC4/Y* (NCBI Reference Sequence: NM_004532.5) can be as a model of MUC4 (MUC4 gene fragment is more than 30KB, too huge to clone and eukaryotic express) in pancreatic cancer for function and mechanism research, as shown in Additional File 4 (Supplementary Figure S1). More importantly, based on *MUC4/Y* with the appropriate lenth of gene sequence, it is easy to construct the unique domain-lacking models of *MUC4/Y* (MUC4) for research. Thus, the present study aimed to investigate the respective role of the unique NIDO, AMOP, and vWD domain or their synergistic effect on MUC4(*MUC4/Y*)-mediated functions and mechanisms: 1) Series of stable PANC-1 cell strains transfected by the *MUC4/Y* gene without or with domain-lacking were established and consistent overexpression quantity of target genes were verified for comparison of different quality caused by domain-lacking. 2) Series of *in vitro* assays were conducted to detect the changes of malignant activities of PANC-1 cells caused by domain-lacking. 3) sequence-based transcriptome analysis, validation of qRT-PCR & Western blot, and systematic comparative analysis were carried out to find the effect afforded by domain-lacking on MUC4-mediated molecule mechanisms, particularly the impact on *MUC4/Y*(MUC4)-triggered malignancy-related positive feedback loops. 4) Comparison and induction were done to illustrate the universality, individuality and synergism of the role of these three unique domains, which can contribute to provide potential targets for overcome pancreatic cancer.

## RESULTS

### Establishment of series of stable PANC-1 cell strains with consistent expression quantity of target genes for comparison of different quality caused by domain-lacking

To investigate the function change of *MUC4/Y* gene without or with domain-lacking in pancreatic cancer, PANC-1 cells, which do not express endogenous MUC4 [32], were stable transfected by target genes, and selected using 10% DMEM containing puromycin (2.0 μg/mL), respectively.

**Figure 1:**
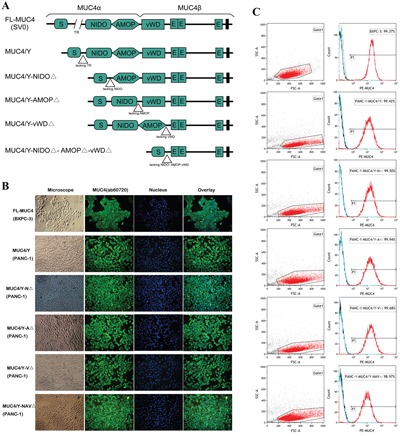
Design and identification of stable consistent expression of the *MUC4/Y* gene without or with domain-lacking **A**. Schematic representation of the design strategy. **B**. IF demonstrating the expression and subcellular localization of *MUC4/Y* gene with domain-lacking (*MUC4/Y*-NIDO^△^, *MUC4/Y*-AMOP^△^, *MUC4/Y*-vWD^△^, *MUC4/Y*-NIDO^△^-AMOP^△^-vWD^△^) was same as that of *MUC4/Y* and wild-type MUC4. **C**. FCM analysis confirmation of target gene expression on cell membrane surface and high purities of the series stable PANC-1 cell clones with overexpression of target gene, respectively.

Figure 1A depicts the design of the *MUC4/Y* gene without or with domain-lacking, corresponding amino acids sequences of domains denoted by different color in Additional File 1. The stable PANC-1 cell clones (N^△^, A^△^, V^△^ and NAV^△^) with over-expression of target genes in mRNA and protein level were verified separately. Figure 1B shows that the expression and subcellular localization of *MUC4/Y* gene with domain-lacking (*MUC4/Y*-NIDO^△^, *MUC4/Y*-AMOP^△^, *MUC4/Y*-vWD^△^, *MUC4/Y*-NIDO^△^-AMOP^△^-vWD^△^) was same as that of *MUC4/Y*, which was both membranous and cytoplasmic staining, indicating similar protein processing in these clones after transfected by target genes, respectively. These distributions were as same as the pancreatic cancer cell line BXPC-3 which is wild-type MUC4 positive-expression as positive control [33]. Cell membrane surface expression was determined by FCM analysis. Figure 1C shows that the frequency of expression of the phenotypic marker MUC4 was 99.37% in BXPC-3 cell line, 99.42% in Y cell clone, 99.50% in N^△^ cell clone, 99.94% in A^△^ cell clone, 99.68% in V^△^cell clone, 98.97% in NAV^△^ cell clone, respectively, indicating high purities of the series stable PANC-1 cell clones and stable overexpression of target genes. These results demonstrated that stable PANC-1 cell strains of overexpression of *MUC4/Y* gene without or with domain-lacking were established, and they were consistent with each other in expression quantity. Thus the different quality between different groups lies in domain-lacking.

### Roles of *MUC4/Y*’s unique domains in cell proliferation, DNA replication, cell cycle and anti-apoptosis under low-nutritional-pressure

**Figure 2:**
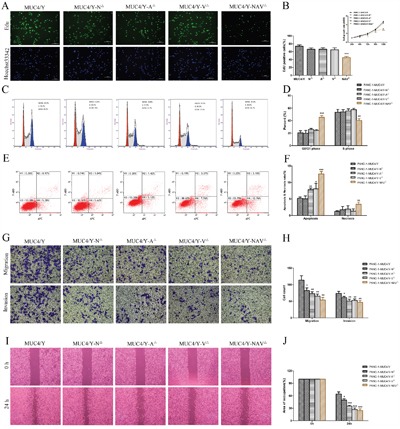
Domain-lacking weakened the role of *MUC4/Y* on malignant activities of PANC-1 cell **A and B**. The altered DNA replication ability was determined by the EdU incorporation assay at various *MUC4/Y*-domains-lacking groups and the control group. The cells growth rates were determined by CCK-8 proliferation assays at various time points. Cell growth rate = point-in-time of the absorbance at 450 nm(A450) / Mean of A450 in 24h. **C and D**. Cell cycle distribution was analyzed by using flow cytometry with PI staining. The percentages of cells at the G0/G1 & S & G2/M phase were plotted. **E and F**. Representative templates of FCM analysis showing the proportion of cells positive for annexin V(-APC) and 7-AAD (top right quadrant) representing the percentage of necrotic cells; the proportion of cells that were annexin V(-APC)–positive and 7-AAD–negative (bottom right quadrant) represented the percentage of apoptotic cells. Bar denotes the percentage of apoptotic and necrotic cells in PANC-1–derived clones. **G** and **H**. Metastatic potential *in vitro* was detected by matrigel migration and invasion assay. Bar graph shows the number of PANC-1–derived clones that had migrated or invaded through the Matrigel. **I** and **J**. Migration capability was detected by wound healing assay. Wound closure was delayed in different groups at 24 h. Occupied area of wound closure by migrated cells was calculated and is depicted in the bar chart. “*”(*P*<0.05), “**”(*P*<0.01), and “***”(*P*< 0.001) indicate a significant difference from PANC-1-*MUC4/Y* control cells.

Figure 2A and 2B shows that under stress from low nutritional status (1% serum), the percentage of EdU-positive cells of NAV^△^ group decreased significantly (*P* = 0.0004), and cell proliferation rate of NAV^△^ group decreased significantly at 72h, 96h, 120 h (*P* = 0.038, 0.005, 0.000, respectively), compared to the PANC-1-*MUC4/Y* control group. These consistent results suggests the simultaneous lack of NIDO, AMOP and vWD domains can lower significantly the ability of cell proliferation and DNA replication in compared to the control (*MUC4/Y* gene without domain-lacking) under stress from low nutritional status.

We further detected cell cycle distribution cultured in low-serum (1% FBS) medium by flow cytometry. As shown in Figure 2C&2D, compared with PANC-1-*MUC4/Y* control groups, cells transfected with *MUC4/Y*-NAV^△^ presented a prominent accumulation of cells in the G0/G1 phase (*P* = 0.0006) and a decrease in the S phase (*P* = 0.007), indicating that the simultaneous lack of NIDO, AMOP and vWD domains can reduce cell proliferation rate by facilitating cell cycle arrest at G0/G1 phase in compared to the control under stress from low nutritional status.

Moreover, as shown in Figure 2E&2F, apoptosis assay showed under stress from low nutritional status (1% serum), compared to the PANC-1-*MUC4/Y* control cells, there were significant increases of apoptosis rate in A^△^, V^△^, NAV^△^ group (*P* = 0.002, 0.045, 0.0001, respectively), and there was the significant increase of necrosis rate in PANC-1-*MUC4/Y*-NAV^△^ cells (*P* = 0.005), indicating that the lack of AMOP or vWD, and the simultaneous lack of NIDO, AMOP and vWD domains (NAV^△^) can restore the cellular response to apoptotic stimuli caused by stress from low nutritional status.

Altogether, these results show that synergistic effect of NIDO, AMOP and vWD domains on proliferation, DNA replication, cell cycle and anti-apoptosis of PANC-1 cells is significant, AMOP or vWD domain of *MUC4/Y* also has significant role on anti-apoptosis of PANC-1 cells.

### Roles of *MUC4/Y*’s unique domains in cell migration and invasion

We used Transwells without or with Matrigel-coated membranes to examine cell migration and invasion, respectively, *in vitro*. Figure 2G&2H shows that compared to the PANC-1-*MUC4/Y* control cells, there were significant decreases of average number of migrating cells in N^△^, A^△^, V^△^, NAV^△^ groups (*P* =0.025, 0.008, 0.005, 0.003, respectively), and there were significant decreases of average number of cells invading through the Matrigel in N^△^, A^△^, V^△^, NAV^△^ groups (*P* = 0.034, 0.008, 0.008, 0.005, respectively). Consistently, as shown in Figure 2I&2J, wound healing assays showed that the migration capability of N^△^, A^△^, V^△^, NAV^△^ groups was less than that of control cells (*P* = 0.013, 0.0007, 0.0005, 0.0004, respectively). These data suggest that the lack of domains can significantly down regulate the effects of *MUC4/Y* on pancreatic cancer cells migration and invasion, and indicate that NIDO, AMOP, vWD, or synergism of them play roles in migration and invasion of PANC-1 cells.

### DEG screening and functional annotation for global mRNA analysis of *MUC4/Y*’s unique domains triggered signatures

We have revealed 1575 differentially expressed genes (DEGs) of *MUC4/Y* over-expressing PANC-1 cells compared with the blank and negative controls in earlier research [16]. Here domain-lacking triggered signatures were analyzed. The filtering condition of DEGs was intersection sets beween DEGs of Domain-lacking triggering and DEGs of *MUC4/Y* gene triggering, absolute value of log2 ratio ≥ 1. Comparative analyses of Domain-lacking groups *vs* control group (PANC-1-*MUC4/Y*) revealed 932 DEGs for N^△^ *vs* Y, 990 DEGs for A^△^ *vs* Y, 1033 DEGs for V^△^ *vs* Y, 1214 DEGs for NAV^△^ *vs* Y, respectively, as shown in Supplementary Table S1-S4 (Additional File 2).

As shown in Supplementary Table S5-S10 (Additional File 3), DEGs annotated against GO and KEGG databases were enriched to identify significant GO biological process terms and pathways, respectively, and adjusted with corrected *P* ≤ 0.05 for GO analysis and pathways, as follows: 1) N^△^ *vs* Y: The significant GO biological process terms were cell projection, synaptic vesicle (*P* = 0.03743, 0.00213, successively); None of pathways were enriched to identify significantly. 2) A^△^ *vs* Y: The significant GO biological process terms were cell projection, membrane, membrane part, intrinsic to membrane, extracellular region, extracellular region part, neuron projection (*P* = 4.74e-05, 0.00100, 0.00207, 0.00385, 0.01009, 0.01542, 0.00213, successively) in “Cellular Component”subdirectory of GO, and signaling, signal transmission (*P* = 0.02800, 0.04822, successively) in “Biological Process”subdirectory of GO; None of pathways were enriched to identify significantly. 3) V^△^ *vs* Y: The significant GO biological process terms were cell projection, neuron projection, membrane, membrane part, intrinsic to membrane, extracellular region (*P* = 1.08e-07, 2.56e-05, 8.63e-05, 0.00054, 0.00105, 0.03525, successively) in “Cellular Component”subdirectory of GO, and signaling, signal transmission, signaling process, neurogenesis, generation of neurons, nervous system development, neuron differentiation, regulation of multicellular organismal development, signal transduction (*P* = 8.42e-05, 0.00016, 0.00020, 0.00121, 0.00659, 0.00860, 0.01776, 0.01919, 0.02644, successively) in “Biological Process”subdirectory of GO; The significant pathway-enriched terms were MAPK signaling pathway, Complement and coagulation cascades, Chemokine signaling pathway, Calcium signaling pathway, Cytokine-cytokine receptor interaction (*P* = 0.01527118, 0.03150312, 0.03150312, 0.04294926, 0.04324608, successively). 4) NAV^△^ *vs* Y: The significant GO biological process terms were cell projection, membrane, intrinsic to membrane, membrane part, integral to membrane (*P* = 0.00033, 0.00138, 0.00502, 0.01920, 0.04597, successively) in “Cellular Component”subdirectory of GO, GTPase regulator activity (*P* =0.04792) in “Molecular Function”subdirectory of GO, and signaling, signal transmission, signaling process (*P* = 2.52e-06, 3.68e-05, 4.65e-05, successively) in “Biological Process”subdirectory of GO; The significant pathway-enriched terms were MAPK signaling pathway (*P* = 0.000554462).

Altogether, the above enrichment results and analyses of GO function and the KEGG pathway show the global function triggered by NIDO(N), or AMOP(A), or vWD(V), or synergism of NIDO, AMOP and vWD domain(NAV) of *MUC4/Y:* 1) The universality of N, A, V, NAV is the function about “cell projection”; The common characters of A, V, NAV are function about “membrane” and “signaling”; The similar characters of A, V are function about “extracellular region” and “neuron projection”. 2) The individuality is the function about “synaptic vesicle” in N, “nervous system generation, development, differentiation” and “regulation of multicellular organismal development” and “effect on Immune system” in V, “GTPase regulator activity” in NAV. 3) As shown in Supplementary Table S1-S4 (Additional File 2), comparative analyses between [domain-lacking groups *vs* control group(PANC-1-*MUC4/Y*)] and [*MUC4/Y* over-expressing groups *vs* control group(PANC-1 cells transfected with empty lentiviral vectors were designated PANC-1-EV. Wild-type PANC-1 and PANC-1-EV cells were used as blank and negative control groups, respectively)] revealed that the expression levels (in transcripts per million, TPM) of most DEGs of *MUC4/Y* triggering were reversed afforded by domain-lacking to a variable extent. Altered gene expression was then confirmed by carrying out qRT–PCR and Western blotting as follows, which results in variation trends were consistent with sequence-based transcriptome analysis.

### QPCR validation of roles of *MUC4/Y*’s unique domains on *MUC4/Y*-mediated mechanisms

To verify that the expression levles of most DEGs of *MUC4/Y* triggering can be reversed by the absence of unique *MUC4/Y* domains, we selected a batch of DEG molecules of *MUC4/Y* triggering for QPCR validation, and we focused on *MUC4/Y*-mediated key mechanisms. Additional File 4 (Supplementary Figure S1-S2) list the summary of representative downstream effector molecules of *MUC4/Y* to activate malignant functions, trigger the positive feedback regulatory loops, and relate with energy metabolism, protein synthesis & modification.

**Figure 3:**
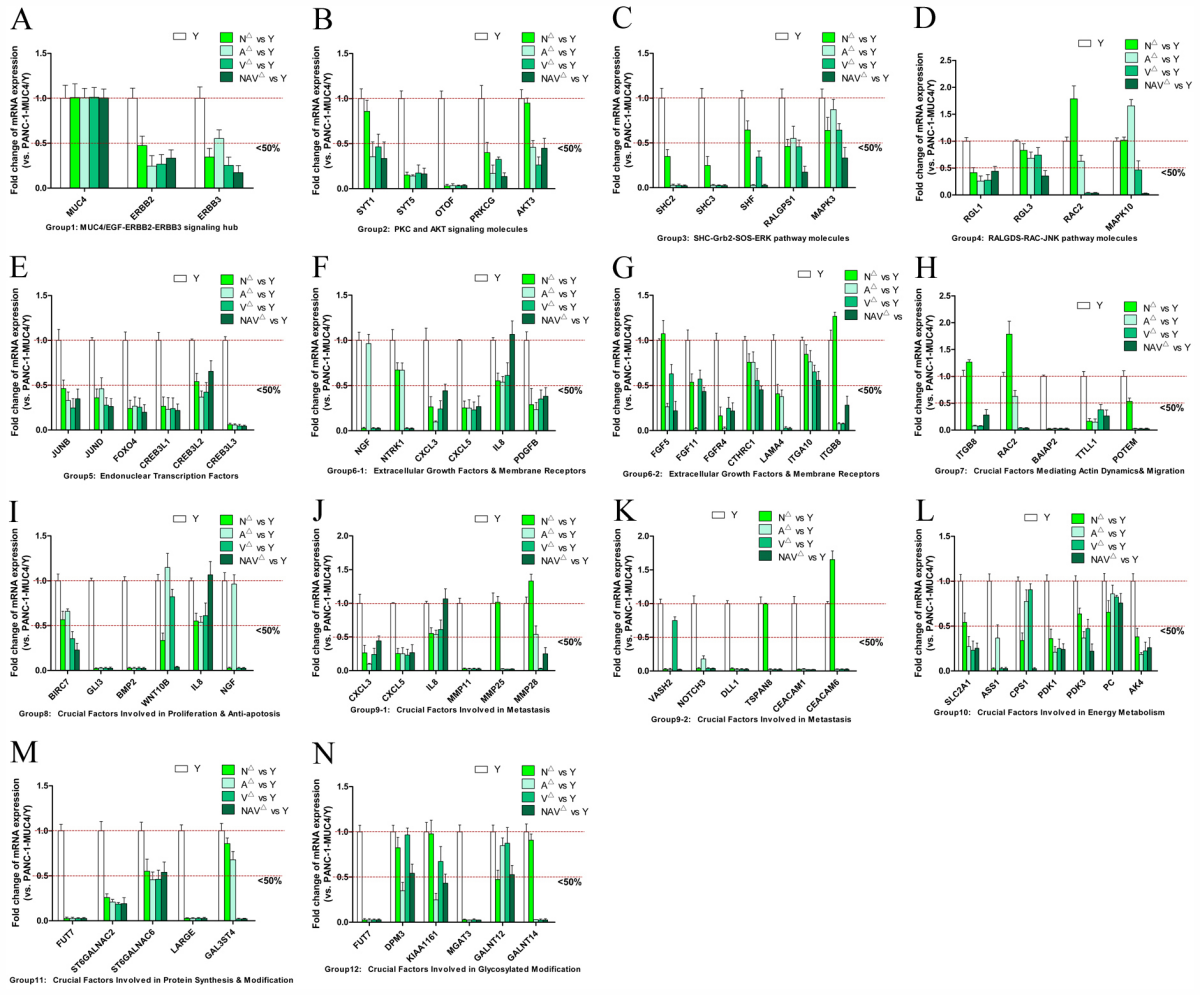
The expression of most DEGs of *MUC4/Y* triggering was reversed in the absence of unique *MUC4/Y* domains(NIDO, AMOP, vWD, or synergy) Representative downstream effector molecules of *MUC4/Y* (i.e. DEGs of *MUC4/Y* over-expressing PANC-1 cells compared with controls in earlier research [16], as shown in Additional File 4) were classified as twelve feature subsets, i.e. Group1-12. QPCR validation results of altered gene expression of these molecules in lacking-domains groups (N^△^, A^△^, V^△^, NAV^△^) and control group (*MUC4/Y*-overexpression). The expression quantity in controls is defined as 1.0, so under the line of “1.0” represents mean expression quantity of experimental groups below that of control group, and under the line of “0.5” represents mean expression quantity of experimental group less than 50% expression of control group.

These selected molecules were classified as twelve feature subsets, i.e. group1-12, as shown in Figure 3. The twelve feature subsets (Group1-12) were “MUC4/EGF-ERBB2-ERBB3 signaling hub”, “PKC and AKT signaling molecules”, “SHC-Grb2-SOS-ERK pathway molecules”, “RALGDS-RAC-JNK pathway molecules”, “Endonuclear Transcription Factors”, “Extracellular Growth Factors & Membrane Receptors”, “Crucial Factors Mediating Actin Dynamics & Migration”, “Crucial Factors Involved in Proliferation & Anti-apotosis”, “Crucial Factors Involved in Metastasis”, “Crucial Factors Involved in Energy Metabolism & Mitochondrial function”, “Crucial Factors Involved in Protein Synthesis & Modification”, “Crucial Factors Involved in Glycosylated Modification”, successively.

To investigate the role of *MUC4/Y*’s domains (N, A, V, NAV) on *MUC4/Y*-mediated mechanisms, altered gene expression of the lacking-domains groups (N^△^, A^△^, V^△^, NAV^△^) *vs*. control group (*MUC4/Y*-overexpression) was validated by independent qRT-PCR test. The results showed the expression levels of most DEGs of *MUC4/Y* triggering were reversed by the absence of unique *MUC4/Y* domains. And the expression levels of representative 68 molecules were all reversed in V^△^ group (*vs* Y). Only small number of molecules expression levels were not reversed, as follows: 1) N^△^ *vs* Y: 8 molecules which failed to reverse expression were RAC2, MAPK10, FGF5, ITGB8, MMP25, MMP28, TSPAN8, CEACAM6 (Figure 3D, 3G, 3H, 3J, 3K, successively) 2) A^△^ *vs* Y: 2 molecules which failed to reverse expression were MAPK10, WNT10B (Figure 3D, 3I, successively) 3) NAV^△^ *vs* Y: one molecule which failed to reverse expression was IL8 (Figure 3F, 3I, 3J, successively). These results were also consistent with sequence-based transcriptome analysis, indicating that our sequencing approach and analytical pipeline were reliable.

Altogether, these results show that *MUC4/Y*’s unique domains (i.e. NIDO, AMOP, vWD, or synergism of them) have roles in *MUC4/Y*-mediated downstream of molecule mechanisms.

### WB validation of effects of *MUC4/Y*’s unique domains on *MUC4/Y*-mediated signaling pathways

To investigate the effect of *MUC4/Y*’s domains (N, A, V, NAV) on *MUC4/Y*-mediated main signaling pathways, the expression changes of the key nodes in the signal path, especially in protein phosphorylation, were verified and compared between the lacking-domains groups (N^△^, A^△^, V^△^, NAV^△^) and control group (*MUC4/Y*-overexpression) by western blotting and measurement of optical density value.

Additional File 4 (Supplementary Figure S1) also shows the main signaling pathways mediated by *MUC4/Y*, which include *MUC4/Y*-dependent increase of ERBB2 and ERBB3 phosphorylation activation, paralleled by the upregulation of key molecules (Ras, Src, focal adhesion kinase [FAK], extracellular signal–regulated kinase [ERK], c-Jun amino-terminal kinase [JNK], AKT, nuclear factor κB [NFκB], inhibitor of NFκB [IκBα], c-Jun), as either total or phosphorylated proteins, in the downstream signaling pathways.

**Figure 4:**
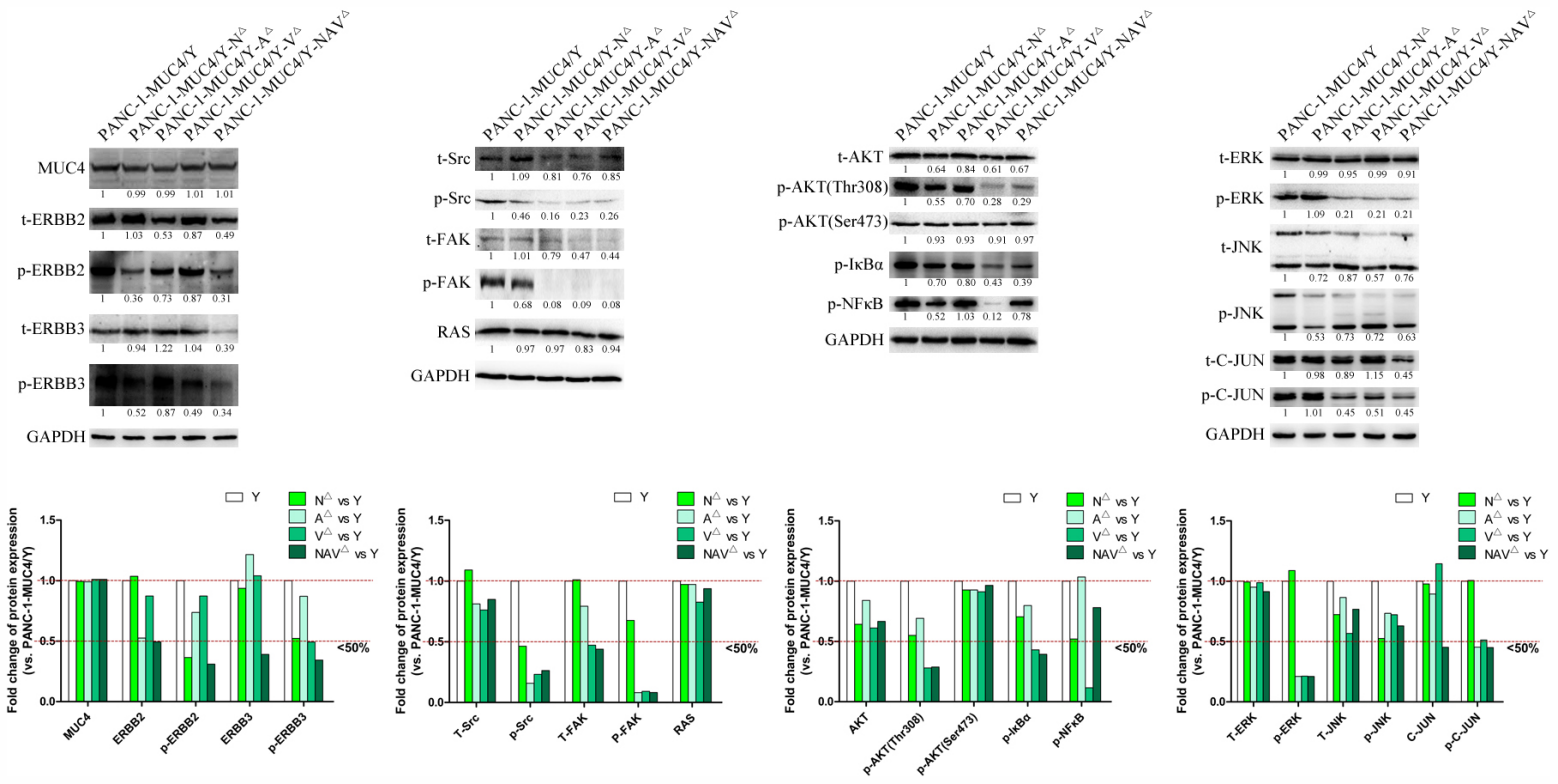
Domain-lacking reduced protein expression level of key nodes of *MUC4/Y*-mediated signal path in the majority Representative signaling pathways mediated by *MUC4/Y* were described as earlier research [16] (Additional File 4, Supplementary Figure S1). WB validation results of the expression changes of the key nodes in the signal path and measurement of optical density value by Image J software. The expression quantity in controls is defined as 1.0, so under the line of “1.0” represents mean expression quantity of experimental groups below that of control group, and under the line of “0.5” represents mean expression quantity of experimental group less than 50% expression of control group.

As shown in Figure 4, domain-lacking reduced protein expression levels of key nodes of *MUC4/Y*-mediated signal path in the majority. And the expression levels of representative 20 molecules were all reversed in NAV^△^ group (*vs* Y). Non- reverse effects were in the minority, as follows: 1) N^△^ *vs* Y: Molecules which failed to lower 6 proteins expression were ERBB2, Src, FAK, ERK, Phospho-ERK, Phospho-c-JUN. 2) A^△^ *vs* Y: Molecules which failed to reduce 2 proteins expression were ERBB3, Phospho-NFκB. 3) V^△^ *vs* Y: Molecules which failed to down- regulate 2 proteins expression were ERBB3, c-JUN. So, these results indicate that *MUC4/Y*’s unique domains have effects on *MUC4/Y*-mediated downstream of signaling pathways, consistently with sequence-based transcriptome analysis and QPCR validation.

### Systematic comparative analysis of FDR-corrected *P*-values of the 18 sigificant enriched functional categories of *MUC4/Y* and reverse effects triggered by domain-lacking

**Figure 5:**
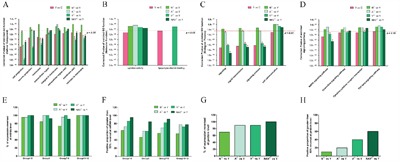
Systematic comparative analysis of FDR-corrected *P*-values of the 18 sigificant enriched functional categories of *MUC4/Y* and reverse effects triggered by domain-lacking **A**. The comparison of corrected *P*-value of enriched GO function (Cellular Component). **B**. The comparison of corrected *P*-value of enriched GO function (Molecular Function). Three Groups (N^△^ *vs* Y, A^△^ *vs* Y, NAV^△^ *vs* Y) failed to enriched on the functional subset “lipopolysaccharide binding”, so they lacked the *P*-value. **C**. The comparison of corrected *P*-value of enriched GO function (Biological Process). **D**. The comparison of corrected *P*-value of enriched signaling pathway. To (A-D), under the red line of “0.5”, or labeled with red asterisk, *, represents FDR-corrected *P*-values, ≤0.05. **E**. Positive proportion of genes (verified by QPCR) number of expression-reversed at different groups to compare reverse-effect rate. **F**. Positive proportion of greater than 50% reversal rate (verified by QPCR) at different groups to compare reverse-effect extent. To (E&F), Group1-5 on behalf of the feature subset “crucial factors involved in signaling hub”. Group 6 on behalf of the feature subset “extracellular growth factors & membrane receptors”. Group7-9 on behalf of the feature subset “crucial factors involved in oncogenic function”. Group10-12 on behalf of the feature subset “crucial factors involved in energy metabolism (Mitochondrial function) and protein synthesis & modification including glycosylated modification (Golgi function)”. **G**. Positive proportion of genes (verified by WB) number of expression-reversed at different groups to compare reverse-effect rate. **H**. Positive proportion of greater than 50% reversal rate (verified by WB) at different groups to compare reverse-effect extent.

As mentioned above, on the whole, loss of *MUC4/Y*’s unique domains weakened the roles of *MUC4/Y* on malignant activities, and impaired expression levels of downstream effector molecules of *MUC4/Y* triggering, demonstrating that *MUC4/Y*’s unique domains have significant roles in *MUC4/Y*-mediated malignant function of pancreatic cancer, downstream of molecule mechanisms, including signaling pathways, respectively. To illustrate the separate feature (of N, A, V, NAV) and reverse-effect rate or extent (of N^△^, A^△^, V^△^, NAV^△^) to *MUC4/Y* functional categories, systematic comparative analysis were carried out, as shown in Figure 5A-5H.

Figure 5A-5D show that the validated sigificant enrichment of GO function and the KEGG pathway of *MUC4/Y* overexpression in PANC-1 cells focuses on 18 functional categories [16] (marked with pink, under the red line of “0.5” represented FDR-corrected *P*-values, *≤0.05*), as follows: cell projection, neuron projection, membrane, extracellular region, integral to membrane, extracellular region part, membrane part, intrinsic to membrane, cytokine activity, lipopolysaccharide binding, signaling, signal transmission, signaling process, cell communication, MAPK signaling pathway, Chemokine signaling pathway, Cytokine-cytokine receptor interaction, TGF-beta signaling pathway.

Compared with *MUC4/Y* overexpression in PANC-1 cells, DEGs of separate domain-lacking groups with reversed expression level were also enriched significantly (labeled with red asterisk, *, Figure 5A-5D), as follows: N^△^ *vs* Y (cell projection), A^△^ *vs* Y(cell projection, neuron projection, membrane, extracellular region, extracellular region part, membrane part, intrinsic to membrane, signaling, signal transmission), V^△^ *vs* Y (cell projection, neuron projection, membrane, extracellular region, membrane part, intrinsic to membrane, signaling, signal transmission, signaling process, MAPK signaling pathway, Chemokine signaling pathway, Cytokine-cytokine receptor interaction), NAV^△^ *vs* Y (cell projection, membrane, integral to membrane, membrane part, intrinsic to membrane, signaling, signal transmission, signaling process, MAPK signaling pathway).

The comparison of FDR-corrected *P*-values of the above18 functional categories (of *MUC4/Y*) showed that the least *P*-values of “extracellular region”and “extracellular region part” were acquired by A^△^ *vs* Y, suggesting that the two functional categories of *MUC4/Y* are the most highly correlated to the function or role of AMOP domain.

The least *P*-values of “cell projection”, “neuron projection”, “membrane”, “membrane part”, “intrinsic to membrane”, “Chemokine signaling pathway”, and “Cytokine-cytokine receptor interaction” were acquired by V^△^ *vs* Y, suggesting that the seven functional categories of *MUC4/Y* are the most highly correlated to the function or role of vWD domain.

The least *P*-values of “signaling”, “signal transmission”, “signaling process”, “MAPK signaling pathway” were acquired by NAV^△^ *vs* Y, suggesting that the four functional categories are the most highly correlated to the synergistic role of unique domains.

Figure 5E-5F show the reverse-effect rate or extent of different domains-lacking at mRNA level verified by QPCR. As shown in Figure 3, representative downstream effector molecules of *MUC4/Y* were classified as twelve feature subsets, i.e. group1-12. Group1-5 can be incorporated as a feature subset of crucial factors involved in signaling hub. Group 6 is a feature subset of extracellular growth factors & membrane receptors. Group7-9 can be incorporated as a feature subset of crucial factors involved in oncogenic function. Group10-12 can be incorporated as a feature subset of crucial factors involved in energy metabolism (Mitochondrial function) and protein synthesis & modification including glycosylated modification (Golgi function). Figure 5E shows positive proportion of genes number of expression-reversed at group N^△^, A^△^, V^△^, NAV^△^, as follows: 1) Group1-5: 95.45% (21/22), 95.45% (21/22), 100% (22/22), 100% (22/22), respectively. 2) Group 6: 84.61% (11/13), 100% (13/13), 100% (13/13), 92.31% (12/13), respectively. 3) Group7-9: 73.91% (17/23), 95.65% (22/23), 100% (23/23), 91.30% (21/23), respectively. 4) Group10-12: 100% (18/18), 100% (18/18), 100% (18/18), 100% (18/18), respectively. Figure 5F shows positive proportion of greater than 50% reversal rate at group N^△^, A^△^, V^△^, NAV^△^, as follows: 1) Group1-5: 63.64% (14/22), 72.73% (16/22), 86.36% (19/22), 95.45% (21/22), respectively. 2) Group 6: 46.15% (6/13), 61.54% (8/13), 61.54% (8/13), 84.62% (11/13), respectively. 3) Group7-9: 56.53% (13/23), 69.57% (16/23), 82.61% (19/23), 91.30% (21/23), respectively. 4) Group10-12: 55.56% (10/18), 77.78% (14/18), 72.22% (13/18), 77.78% (14/18), respectively.

Figure 5G-5H show the reverse-effect rate or extent of different domains-lacking at protein level confirmed by WB. As shown in Figure 4, the expression of representative key nodes in the signal pathways mediated by *MUC4/Y* were detected. Figure 5G shows positive proportion of genes number of expression-reversed at group N^△^, A^△^, V^△^, NAV^△^ was 70% (14/20), 90% (18/20), 90% (18/20), 100% (20/20), respectively. Figure 5H shows positive proportion of greater than 50% reversal rate at group N^△^, A^△^, V^△^, NAV^△^ was 10% (2/20), 20% (4/20), 40% (8/20), 60% (12/20), respectively.

Altogether, these results indicate that the lack of three unique domains (N^△^, A^△^, V^△^) plays the definite effects on reversing *MUC4/Y*-mediated malignant function and downstream of molecule mechanisms, among which N^△^ has the weakest effects on that. Notably, the simultaneous lack of three unique domains (NAV^△^) plays the most significant effects on reversing *MUC4/Y*-mediated downstream of signal pathways than the respective lack groups (N^△^, A^△^, V^△^), which is consistent with above mentioned that four functional categories of *MUC4/Y* (signaling, signal transmission, signaling process, MAPK signaling pathway) were the most highly correlated to the synergistic role of unique domains.

## DISCUSSION

In the present study, we focus on investigate the respective role of the unique NIDO, AMOP, and vWD domain or their synergistic effect on *MUC4/Y*(MUC4)-mediated functions and mechanisms. Based on *MUC4/Y*, we initially constructed homologous genes lacking unique domains present in MUC4, i.e., *MUC4/Y*-NIDO^△^, *MUC4/Y*-AMOP^△^, *MUC4/Y*-vWD^△^, *MUC4/Y*-NIDO^△^-AMOP^△^-vWD^△^. We also constructed series of stable PANC-1 cell strains transfected by the *MUC4/Y* gene without or with domain-lacking, which with consistent forced gene expression rate and subcellular localization, for comparison of different quality caused by domain-lacking.

On the whole, the results of function assays *in vitro*, sequence-based transcriptome analysis and confirmatory testing were similar in variation trends, as follows: 1) Domain-lacking weakened the roles of *MUC4/Y* on malignant activities of PANC-1 cell *in vitro*, which include the significant decrease of cell proliferation and DNA replication, consistently followed by the cell cycle arrest at G0/G1 phase, significant increase of apoptosis & necrosis rate under stress from low nutritional status, which also include significant decrease of the capabilities of migration and invasion. 2) Domain-lacking reversed the expression levels of the most of differentially expressed genes (DEGs) caused by *MUC4/Y* over-expressing PANC-1 cells compared with the blank and negative controls. Firstly, sequence-based transcriptome analysis revealed that among 1575 DEGs of *MUC4/Y*-overexpression triggered, the expression levels (in transcripts per million, TPM) of 932 genes were reversed 2 folds or more than by NIDO-lacking triggered, 990 genes by AMOP-lacking triggered, 1033 genes by vWD-lacking triggered, 1214 genes by three domains-simultaneously-lacking triggered, as shown in Additional File 2 (Supplementary Table S1-S4). Secondly, QPCR validation of representative downstream effector molecules of *MUC4/Y* (which to trigger malignancy-related positive feedback regulatory loops, and relate with energy metabolism, protein synthesis & modification) revealed that the expression levels of representative 68 molecules were all reversed by vWD-lacking with 100% reversed rate, NIDO-lacking with 88.24% (60/68) reversed rate, AMOP-lacking with 97.06% (66/68) reversed rate, three domains-simultaneously-lacking with 98.53% (67/68) reversed rate. Thirdly, WB validation of the expression changes (causesd by domain-lacking) of the key nodes on *MUC4/Y*-mediated main signaling pathways revealed that the expression levels of representative 20 molecules were all reversed by three domains-simultaneously-lacking with 100% reversed rate, NIDO-lacking with 70% (14/20) reversed rate, AMOP-lacking with 90% (18/20) reversed rate, vWD-lacking with 90% (18/20) reversed rate. Altogether, these results demonstrate that *MUC4/Y*’s unique domains have significant roles in *MUC4/Y*-mediated malignant function of pancreatic cancer, downstream of molecule mechanisms, particularly *MUC4/Y*-triggered malignancy-related positive feedback loops, respectively.

Notably, the simultaneous lack of three unique domains (NAV^△^) had the most significant effects on reversing *MUC4/Y*-mediated functions *in vitro* (as shown in Figure 2). That was consistent with the most significant effects on reversing *MUC4/Y*-mediated molecule mechanisms than the respective lack groups (N^△^, A^△^, V^△^), as shown in Figure 5F & 5H, NAV^△^ group ranked the highest overall on positive proportion of greater than 50% reversal rate (95.45% in Group1-5 at mRNA level, 84.62% in Group6 at mRNA level, 91.30% in Group7-9 at mRNA level, 77.78% in Group10-12 at mRNA level, and 60% at protein level). Coincidentally, the above two points are consistent with the results of systematic comparative analysis, which revealed that the four enriched functional categories of *MUC4/Y* (signaling, signal transmission, signaling process, MAPK signaling pathway) were the most highly correlated to the synergy of three unique domains (NAV). Thus, we conclude that the synergistic roles of NIDO, AMOP and vWD domains on *MUC4/Y*-mediated functions and mechanisms are more prominent than the respective domain because the synergy of three domain plays the more remarkable effects on *MUC4/Y*-mediated signaling hub.

In addition, the results and analyses of GO function and the KEGG pathway show that the functional category “GTPase regulator activity” was enriched or triggered only by the synergy of three unique domains (NAV). The functional category “GTPase regulator activity” plays roles in the growth control, regulating the organization and remodelling of the actin cytoskeleton, regulating cell migration, regulating cancer metastasis via modulation of GTPases and GTP hydrolysis [34, 35]. Consistently, our results showed the simultaneous lack of NIDO, AMOP and vWD domains (NAV^△^) weakened the roles of *MUC4/Y* on malignant activities of PANC-1 cell *in vitro*, including cancer metastasis-related capabilities. And NAV^△^ reversed batches of crucial factors involved in oncogenic function (crucial factors mediating proliferation, anti-apotosis, actin dynamics & migration, metastasis, etc). Thus, finding paths to break the synergy of three unique domains (NAV) will be helpful to weaken capabilities of pancreatic cancer metastasis, specially to the patients with MUC4 positive expression.

In contrast to previous studies [3, 23, 36, 40–44], this article is not confined to a piont. Instead, based on transcriptome analysis with big data and accurate statistic mathematic model, systematic validation and comparison, we found and noted that the respective roles of the unique NIDO, AMOP, and vWD domain or their synergistic effects on *MUC4/Y* (MUC4)-mediated mechanisms were with complex features, even the overlapping in different dimension, in accordance with *MUC4/Y* (MUC4)-mediated mechanisms.

Our previous studies [16] have detailed the enormity of the potential regulatory circuitry in pancreatic cancer afforded by *MUC4/Y* (MUC4), which is with remarkable features, as follows: 1) The malignancy-related positive feedback loops work on the ring circuit path, i.e., triggering(MUC4/EGF-ERBB2-ERBB3 signaling hub)- activating(main MAPK signaling pathways)- transmitting(endonuclear transcription factors)- producing or upregulating(cytokines, growth factors, extracellular matrix, integrins, membrane receptors)- activating(main MAPK signaling pathways)- transmitting(endonuclear transcription factors)- sustained upregulating (MUC4/EGF-ERBB2-ERBB3 signaling hub). 2) Producing or upregulating crucial factors involved in oncogenic function is for malignant activities of pancreatic cancer. 3) Producing or upregulating crucial factors involved in energy metabolism, protein synthesis & modification is for supplying energy and survial material. 4) Producing or upregulating cytokines, growth factors, and adhesion molecules is for pancreatic cancer cell to affect the tumor milieu by cell–ECM and cell–cell interplay. 5) Complex interplays between several signaling pathways form network. So, MUC4 overexpression correlates significantly with poor prognosis of PDAC, not only because it plays important roles in the carcinogenesis and malignant progression of human pancreatic cancer, but also its triggered malignancy-related positive feedback loops are the root of resistance to chemotherapy and molecular targeted therapy.

Thus, in light of *MUC4/Y* (MUC4)-mediated complex mechanisms, batches of *MUC4/Y* triggering DEG molecules which can represent above mentioned five remarkable features were selected for QPCR and western blotting validation of changes afforded by different domains-lacking, followed by systematic comparison to effect extent of different domains-lacking on two levels. Importantly, the present study is the first to demonstrate that NIDO, AMOP, and vWD domain or their synergy play significant roles on *MUC4/Y* (MUC4)- triggered malignancy-related positive feedback loops of pancreatic cancer to a variable extent. Excitingly, we find and verify that the absence of the unique domains (NIDO, AMOP, vWD) respectively or simultanously contributes to weaken MUC4-mediated malignant activities, cut off *MUC4/Y*-triggered malignancy-related positive feedback loops, and down-regulating transcription of the cascading downstream effectors in pancreatic cancer. These mean that in order to defeat the refractory and drug-resistant pancreatic cancer with MUC4 expression, some pathways can be explored besides repressing MUC4 transcription [45, 46], as follows: 1) To improve reversed effects of domain-lacking. 2) To break the synergism of domains of MUC4. 3) To disrupt MUC4/EGF-ERBB2-ERBB3 signaling hub. 4) Above three remedy combined chemotherapy.

Additionally, in this paper, the results of sequence-based functional annotation for global mRNA analysis also are in agreement with the results of QPCR validation, and are helpful for illustrating the universality and individuality of the role of these three unique domains. For example, the enrichment analyses of GO function [47] and the KEGG pathway [48] show that the common function of NIDO(N), or AMOP(A), or vWD(V), or the synergism of NIDO, AMOP and vWD domain(NAV) were enriched in the feature set “cell projection”, consistently with the structural features of MUC4 and *MUC4/Y*, i.e., MUC4 or *MUC4/Y* is anchored to the cell surface by hydrophobic transmembrane region, which locates carboxyl terminal of NIDO, AMOP and vWD domains, so the three domains protrude from the cell surface. The common characters of A, V, NAV were enriched in two function-sets “membrane” and “signaling”, suggesting that A, V, NAV have the more significant role on *MUC4/Y*-mediate membrane-related molecules expression and signal activation and transmission, which contribute to *MUC4/Y*-triggered malignancy-related positive feedback loops. The common characters of A, V were enriched in two function-sets “extracellular region” and “neuron projection”, suggesting that both of the domains play more prominent roles in cell-cell interaction, adhesion to the extracellular matrix, and specialized features of neuron. Furthermore, systematic comparative analysis revealed that the two enriched functional categories of *MUC4/Y* (extracellular region, extracellular region part) were the most highly correlated to AMOP domain.

Interestingly, GO function and the KEGG pathway show that the function on “nervous system generation, development, differentiation” and “regulation of multicellular organismal development” and “effect on Immune system”was enriched or triggered only by vWD domain, which is consistent with systematic comparative analysis results that the seven enriched functional categories of *MUC4/Y* (cell projection, neuron projection, membrane, membrane part, intrinsic to membrane, chemokine signaling pathway, cytokine-cytokine receptor interaction) were the most highly correlated to vWD domain. Altogether, these individuality of vWD domain suggests MUC4-vWD domain may be involved in various biological function owing to its structure characteristics, i.e., vWD carry the putative GDPH cleavage site in its N-terminal region [23] and adjacent to MUC4-EGF domains with the the nearest distance [3, 23, 31], suggesting vWD domain may be the most potential target for blocking MUC4-triggered malignancy-related regulatory circuitry. Thus, further studies on vWD domain is ongoing in our laboratory.

## MATERIALS AND METHODS

### Establishment and identification of series of PANC-1 cell strains expressing *MUC4/Y* with domain-lacking stablely

The PANC-1 pancreatic cancer cell line was obtained from the Shanghai Institutes for Biological Sciences, Chinese Academy of Sciences. As described earlier [16], we estalished series of PANC-1 cell strains expressing *MUC4/Y* with domain-lacking stably, major workflow as follows: 1) Series of cDNA fragments encoding the *MUC4/Y* gene with domain-lacking were designed (Additional File 1), including the lack of NIDO, or AMOP, or vWD, or the simultaneous lack of NIDO, AMOP and vWD domain, named *MUC4/Y*-NIDO^△^, *MUC4/Y*-AMOP^△^, *MUC4/Y*-vWD^△^, *MUC4/Y*-NIDO^△^-AMOP^△^-vWD^△^ correspondingly. A Kozak sequence (GCCACC) before the ATG initiation codon for optimal translation and with unique restriction sites present in the multiple clone site (MCS) of the lentiviral vector but absent from the target cDNA sequence was contained, respectively. These target sequences were synthesized and cloned in a pUC57vector (GenScript). 2) The series of target cDNA sequences were subcloned into the lentiviral vector pCDH-CMV-MCS-EF1-Puro (Cat.#CD510B-1, System Biosciences, USA) respectively, and lentiviral supernatant were produced by 293T cells which were transiently transfected with pCDH-CMV-MCS-EF1-Puro/ target-gene and the pPACKH1 Lentivector Packaging Kit (Cat. #LV500A-1, System Biosciences, USA) with Lipofectamine 2000 (Invitrogen LifeTechnology) according to the manufacturer’s instructions. 3) PANC-1 cells were transfected by series of target genes by carrying out at 20 multiplicity of infection with the lentivirus and using polybrene (8 μg/mL; Sigma-Aldrich) to augment infection efficiency. Stable clones were then selected in medium containing puromycin (2 μg/mL; Sigma-Aldrich). 4) As described previously [16], stable transfected PANC-1 cells overexpressing the *MUC4/Y* gene were designated PANC-1-*MUC4/Y*(*abbr*. Y). Correspondingly, stable transfected PANC-1 cells overexpressing the series of *MUC4/Y* with domain-lacking genes AMOP were designated PANC-1- *MUC4/Y*-NIDO^△^(*abbr*. N^△^), PANC-1- *MUC4/Y*-AMOP^△^(*abbr*. A^△^), PANC-1- *MUC4/Y*-vWD^△^(*abbr*. V^△^), PANC-1- *MUC4/Y*-NIDO^△^-AMOP^△^-vWD^△^(*abbr*. NAV^△^), respectively.

To verify these stable clones and identify the characteristic of expression and localization of target genes, a variety of detection methods were used, including immunofluorescence (IF), flow cytometry (FCM), quantitative real-time PCR (QPCR), and western blotting (WB).

We used anti-MUC4 mouse monoclonal antibody (#ab60720, Abcam, Cambridge, UK) to detect specific protein expression because this specific antibody is against N-terminal amino acids 79-189 of Human MUC4 (http://www.abcam.com/muc4-antibody-ab60720.html), which is existing consistently in the sequence of *MUC4/Y*, *MUC4/Y*-NIDO^△^, *MUC4/Y*-AMOP^△^, *MUC4/Y*-vWD^△^, *MUC4/Y*-NIDO^△^-AMOP^△^-vWD^△^, respectively. Detailed methods of IF, FCM and WB have been described earlier [16, 18, 44].

As described previously [16], the specific primers (forward: 5′-TGGGTGTCCCTGAGCTGC-3′, reverse: 5′-TGATGTGGCTGTGCGTCTC-3′) and TaqMan probe (5′-ATGTGGTCCCAGGAATGACAACACCGT-3′) designed for *MUC4/Y*, also lie in target Sequences of *MUC4/Y*-NIDO^△^, *MUC4/Y*-AMOP^△^, *MUC4/Y*-vWD^△^, and *MUC4/Y*-NIDO^△^-AMOP^△^-vWD^△^, so the specific primers and TaqMan probe were used to detect the expression of *MUC4/Y* with domain-lacking genes in mRNA level. Each quantification PCR was performed in triplicate and repeated thrice independently.

### Proliferation assays and Edu retention assays

A Cell Counting Kit-8 (#C0038; Beyotime) cell proliferation assay was performed according to the manufacturer’s instructions. Cells were grown in low-serum (1% FBS) medium as described earlier [16]. Cell growth rate = point-in-time of the absorbance at 450 nm(A450) / Mean of A450 in 24h. Edu retention assays were performed to examine DNA replication. Dissociated cells were exposed to 25 μM of 5-ethynyl-2′-deoxyuridine (Edu, RiboBio, Guangzhou, China) for 2 hr at 37°C, and then the cells were fixed in 4% paraformal-dehyde. After permeabilization with 0.5% Triton-X, the cells were reacted with 1× Apollo reaction cocktail (RiboBio) for 30 min. Subsequently, the DNA contents of the cells were stained with Hoechst 33342 for 30 min and visualized under a fluorescence microscope. The experiments were repeated thrice independently.

### Cell cycle analysis

Cells were treated with trypsin at 72 hours after incubation with low-serum (1% FBS) medium and fixed in 70% ethanol for 2 hours at 4°C. After being washed twice with phosphate-buffered saline, the cells were incubated with 0.1 mg/mL RNase A (Sigma-Aldrich Co., St Louis, MO, USA) at 37°C for 30 minutes. The cells were then resuspended in 0.05 mg/mL of propidium iodide (Keygen Biotech, Nanjing, People’s Republic of China) at 4°C for 30 minutes while being protected from light. Finally, the processed cells were analyzed in a FACSort flow cytometer (BD Biosciences, San Jose, CA, USA). Evaluation of the data was performed by CellQuest software (BD Biosciences). The experiments were repeated thrice independently.

### Apoptosis assay

Following 48-h treatment with low-serum (1% FBS) medium, PANC-1–derived clones were collected in PBS for apoptosis assay and flow cytometric analysis as described previously [16]. The experiments were repeated thrice independently.

### *In vitro* migration and invasion assays

We used modified 24-well Boyden chambers for the cell migration and invasion assays as described earlier [16]. For the *in vitro* wound-healing assay, a cell-free area of the culture medium was wounded by scratching with a 200-μL pipette tip. Cell migration into the wound area was monitored in serum-free medium and photographed under a fluorescence microscope at 0 and 24 h. The experiments were repeated thrice independently.

### Sequence-based digital gene expression analysis, DEG Gene Ontology functional enrichment and pathway enrichment analysis

For the five groups of cells: PANC-1-*MUC4/Y*, PANC-1-*MUC4/Y*-N^△^, PANC-1-*MUC4/Y*-A^△^, PANC-1-*MUC4/Y*-V^△^, PANC-1-*MUC4/Y*-NAV^△^, same protocol was carried out, as described previously [16], mainly including extracting total RNA from different groups of cells, confirming RNA integrity, transcriptome analysis, Illumina sequencing, screening of differentially expressed genes (DEGs), and functional annotation through the in-house bioinformatics analysis pipeline. DEGs annotated against the Gene Ontology (GO) and Kyoto Encyclopedia of Genes and Genomes (KEGG) databases were enriched to identify significant GO biological process terms and pathways, respectively, and adjusted with corrected *P* ≤ 0.05 for GO and pathways analysis.

### Validation of altered gene expression of different groups by QPCR

Altered gene expression of the lacking- domains groups *vs. MUC4/Y*-overexpression group were validated by Quantitative real-time PCR (QPCR), which was performed using standard procedures using a SYBR Premix Ex *Taq* Kit (TaKaRa, China) with specific primers as described previously [16]. cDNA was generated using an iScript cDNA Synthesis Kit (Bio-Rad). Ct values were normalized to the *18S* gene and a relative quantitative method (ΔΔCt) was used to evaluate quantitative variation. The relative expression level (defined as the fold change) of target genes were calculated to the relative expression detected in the corresponding control cells, which was defined as 1.0.

### Validation of altered expression of crucial factors involved in signaling hub in different groups by WB

Altered expression of crucial factors involved in signaling hub in different groups were validated by western blotting (WB), which was performed using standard procedures as described previously [16]. Briefly, cell lysates were prepared as described previously [49]. After the concentrations were determined using the Bradford assay, proteins (30 μg/lane) were resolved on 4-20% Mini-PROTEAN TGX precast gels (#456-1093; Bio-Rad). The resolved proteins were transferred onto polyvinylidene difluoride membranes, blocked with 5% non-fat milk in phosphate-buffered saline (PBS) for 2 h, and immunoblotted with a primary antibody. After incubation with a secondary antibody, blots were visualized by enhanced chemiluminescence (Millipore, Billerica, MA). GADPH was used as the loading control.

### Statistical analysis

Statistical analysis was performed using SPSS 20.0 (IBM SPSS Inc.). The results were confirmed by conducting at least three independent experiments in the present study. All data presented are the mean ± standard deviation (SD) of *n* independent measurements unless noted otherwise. Statistical analysis was performed with one-way ANOVA for multiple groups and the unpaired Student *t*-test for individual groups. *P*<0.05 was considered statistically significant.

## SUPPLEMENTARY DATA






