# Supplementary material for: NIDO, AMOP and vWD domains of MUC4 play synergic role in MUC4 mediated signaling
Source: Oncotarget. 2017 Jan 2;8(6):10385–99. doi: 10.18632/oncotarget.14420 (PMC5354666; doi:10.18632/oncotarget.14420)
Supplement: Supplementary file 1 [file oncotarget-08-10385-s001.pdf]

NIDO, AMOP and vWD domains of MUC4 play synergic role in MUC4 mediated signaling

SUPPLYMENTARY DATA

Additional File 1: Design and identification of stable expression of the *MUC4/Y* gene without or with domain-lacking

See Additional File 1.

Additional File 2 (Supplementary Table 1-4): Differentially expressed genes (DEGs) of different domain-lacking groups *vs* control group (PANC-1-*MUC4/Y*), respectively.

Supplementary Table 1: List of 932 DEGs for N△ *vs* Y.

See Supplementary File 1.

Supplementary Table 2: List of 990 DEGs for A△ *vs* Y.

See Supplementary File 1.

Supplementary Table 3: List of 1033 DEGs for V△ *vs* Y.

See Supplementary File 1.

Supplementary Table 4: List of 1214 DEGs for NAV△ *vs* Y.

See Supplementary File 1.

Additional File 3 (Supplementary Table 5-10): Sequence-based global annotation of GO function and the KEGG pathway analysis illustrate the universality and individuality of the role of these three unique domains.

Supplementary Table 5: Functional categories of common differentially expressed genes (DEGs) in PANC-1 cells of overexpressing *MUC4/Y*-N△ as compared with PANC-1 cells of overexpressing *MUC4/Y*. DEGs were annotated according to the indicated Gene Ontology system. *P*-values indicate the statistical significance of the enrichment of these terms as calculated by Fisher's exact test and corrected for multiple testing using the Bonferroni method, $P \leq 0.05$.

See Supplementary File 2.

Supplementary Table 6: Functional categories of common differentially expressed genes (DEGs) in PANC-1 cells of overexpressing *MUC4/Y*-A△ as compared with PANC-1 cells of overexpressing *MUC4/Y*. DEGs were annotated according to the indicated Gene Ontology system. *P*-values indicate the statistical significance of the enrichment of these terms as calculated by Fisher's exact test and corrected for multiple testing using the Bonferroni method, $P \leq 0.05$.

See Supplementary File 2.

Supplementary Table 7: Functional categories of common differentially expressed genes (DEGs) in PANC-1 cells of overexpressing *MUC4/Y*-V△ as compared with PANC-1 cells of overexpressing *MUC4/Y*. DEGs were annotated according to the indicated Gene Ontology system. *P*-values indicate the statistical significance of the enrichment of these terms as calculated by Fisher's exact test and corrected for multiple testing using the Bonferroni method, $P \leq 0.05$.

See Supplementary File 2.

Supplementary Table 8: Functional categories of common differentially expressed genes (DEGs) in PANC-1 cells of overexpressing MUC4/Y-NAV△ as compared with PANC-1 cells of overexpressing MUC4/Y. DEGs were annotated according to the indicated Gene Ontology system. *P*-values indicate the statistical significance of the enrichment of these terms as calculated by Fisher's exact test and corrected for multiple testing using the Bonferroni method, $P \leq 0.05$.

See Supplementary File 2.

Supplementary Table 9: Representative KEGG pathways from signaling pathway impact analysis of DEGs in PANC-1 cells of overexpressing *MUC4/Y*-V△ as compared with PANC-1 cells of overexpressing *MUC4/Y*. DEGs were annotated with the indicated KEGG database. *P*-values were FDR-corrected for multiple testing, $P \leq 0.05$. Status predictions were obtained by signaling pathway impact analysis[50]taking fold-change estimates and pathway topology into account.

See Supplementary File 2.

Supplementary Table 10: Representative KEGG pathways from signaling pathway impact analysis of DEGs in PANC-1 cells of overexpressing *MUC4/Y*-NAV△ as compared with PANC-1 cells of overexpressing *MUC4/Y*. DEGs were annotated with the indicated KEGG database. *P*-values were FDR-corrected for multiple testing, $P \leq 0.05$. Status predictions were obtained by signaling pathway impact analysis[46], taking fold-change estimates and pathway topology into account.

See Supplementary File 2.

Additional File 4 (Supplementary Figure 1-2): The summary of representative downstream effector molecules of *MUC4/Y* to activate malignant functions, trigger the positive feedback regulatory loops, and relate with energy metabolism, protein synthesis & modification, for QPCR and western blotting validation of changes afforded by different domains-lacking, followed by systematic comparison to effect extent of different domains-lacking on two levels.

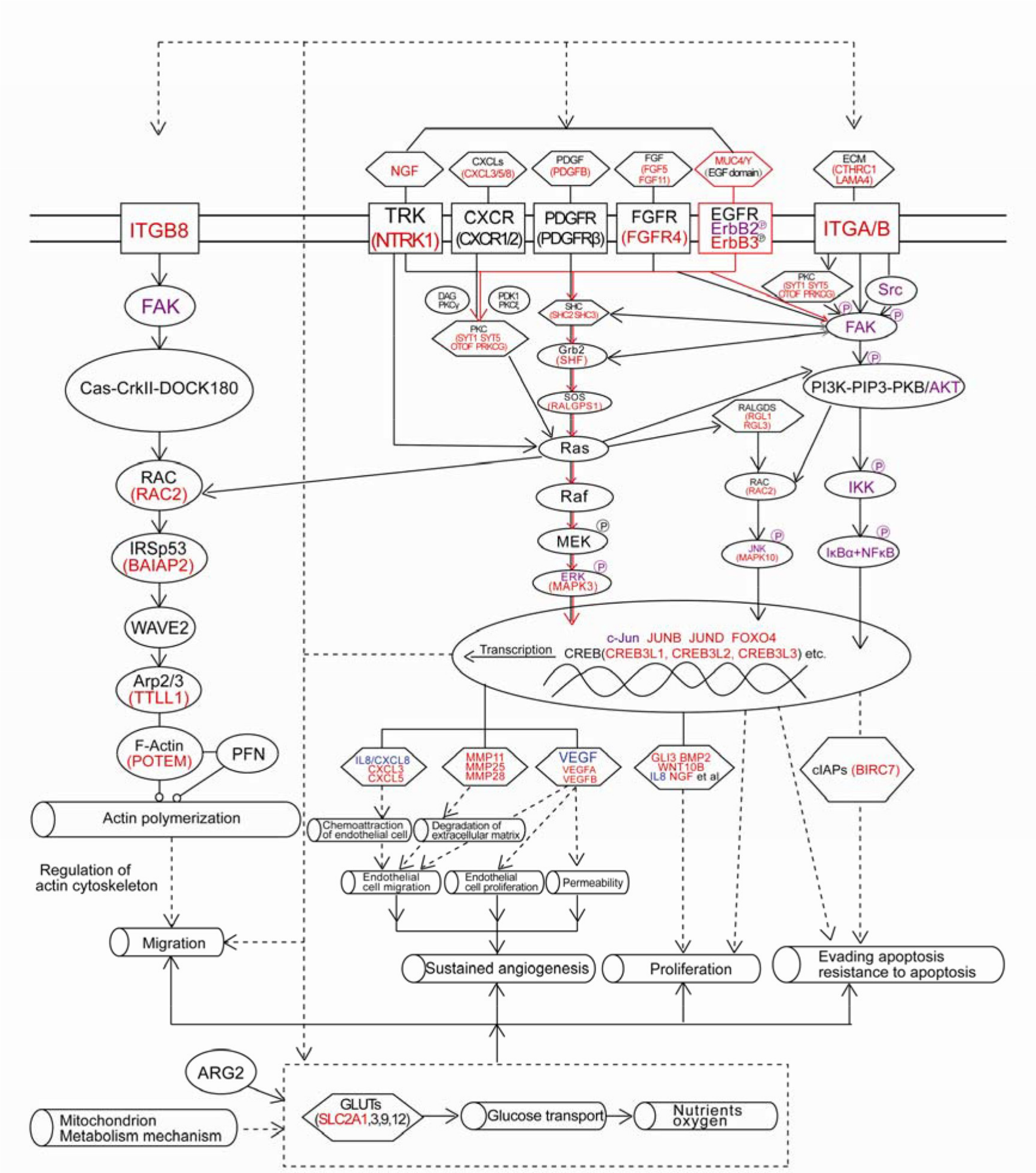

Supplementary Figure 1: Our previous studies[16] have detailed the enormity of the potential regulatory circuitry in pancreatic cancer afforded by MUC4(*MUC4/Y*) as a condensed version depicted in that literature. MUC4/Y-dependent pathways involved in malignant activity. Scheme depicts gene complexes and families (hexagons), membrane receptors (rectangles), function (hollow cylinders), and others (ovals). Red, purple, and blue key factors in the signaling pathways were verified by qRT-PCR, western blotting, and ELISA, respectively. Red lines denote the triggers.

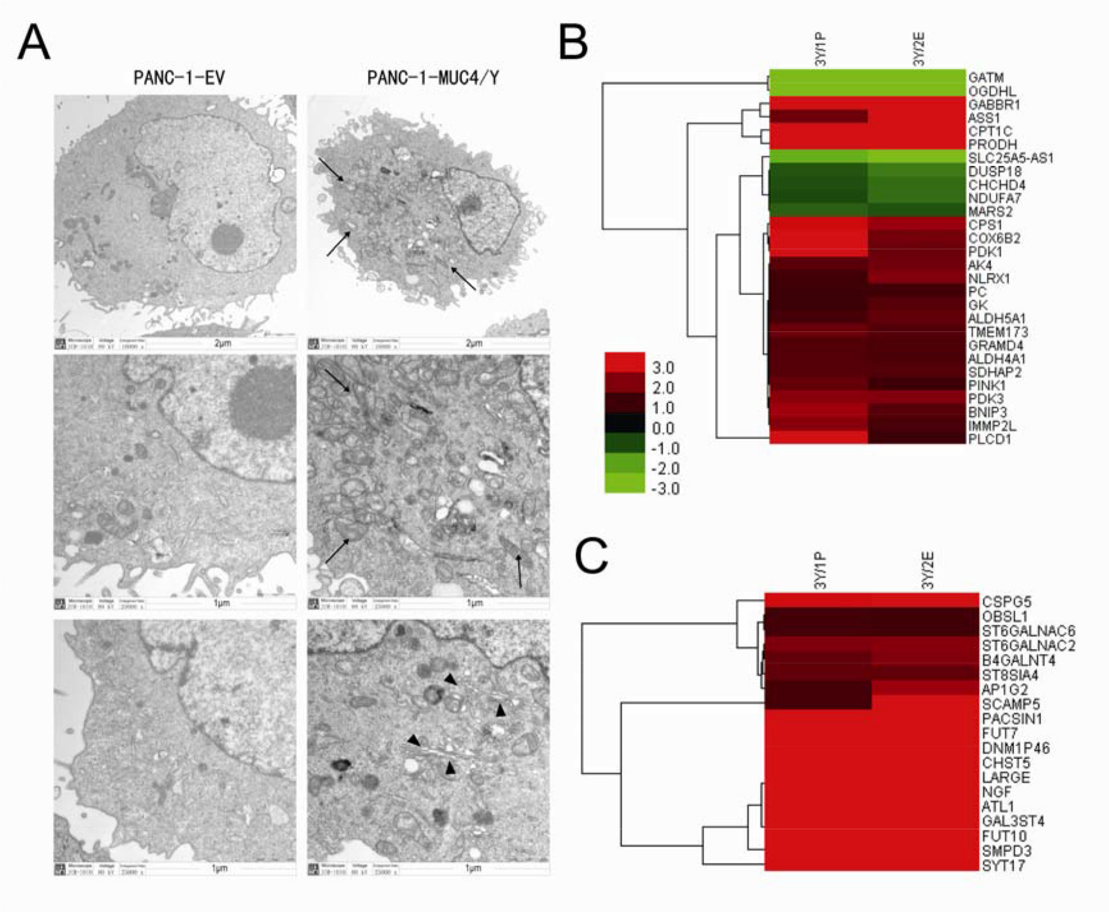

Supplementary Figure 2: *MUC4/Y* induces increased mitochondrial mass and Golgi bodies in pancreatic cancer cells and triggers the expression-changes of downstream effector molecules related with energy metabolism, protein synthesis & modification. A. Representative ultrathin electron microscopy sections were viewed and photographed at 80 kV. Left: PANC-1-EV cell (negative control); right: PANC-1-MUC4/Y cell. Twenty cells from each cell line were observed in two independent experiments. Arrows (↑) indicate dividing mitochondria in PANC-1 cells transfected with MUC4/Y construct, arrowheads (▲) indicate the Golgi complex. Top: ×10000 magnification; middle and bottom: ×25000 magnification. **B.** DEGs relate to mitochondrial component and energy metabolism function. **C.** DEGs relate to Golgi body component and protein synthesis & modification function. Genes depicted in red were upregulated; genes depicted in green were downregulated in PANC-1-MUC4/Y cells compared to controls. 3Y/1P: gene expression level in PANC-1-MUC4/Y cells compared to that in PANC-1 cells; 3Y/2E: gene expression level in PANC-1-MUC4/Y cells compared to that in PANC-1-EV cells. PANC-1 cells transfected with empty lentiviral vectors (EV) were designated PANC-1-EV. Wild-type PANC-1 and PANC-1-EV cells were used as blank and negative control groups, respectively.
