# Supplementary material for: NIDO, AMOP and vWD domains of MUC4 play synergic role in MUC4 mediated signaling
Source: Oncotarget. 2017 Jan 2;8(6):10385–99. doi: 10.18632/oncotarget.14420 (PMC5354666; doi:10.18632/oncotarget.14420)
Supplement: Supplementary file 2 [file oncotarget-08-10385-s002.docx]

**Additional file 2: Differentially expressed genes (DEGs) of different domain-lacking groups** ***vs* control group (PANC-1-MUC4/Y),respectively, absolute value of log2 ratio ≥1.**

Common Notes: Y:**PANC-1-MUC4/Y;** BC: Wild-type PANC-1as blank control; NC: PANC-1 cells transfected with empty lentiviral vectors as negative control; N^△^: **PANC-1-**MUC4/Y-NIDO^△^; A^△^: **PANC-1-**MUC4/Y-AMOP^△^; V^△^: **ANC-1-** MUC4/Y-vWD^△^; NAV^△^: **PANC-1-** MUC4/Y-NIDO^△^-AMOP^△^-vWD^△^

**Table S1. List of 932 DEGs for N^△^ *vs* Y.**

| **Gene ID** | **Symbol** | **log_2_Ratio (Y *vs* BC)** | **log_2_Ratio (Y *vs* NC)** | **log_2_Ratio (N^△^ *vs* Y)** |
| --- | --- | --- | --- | --- |
| 90993 | CREB3L1 | 9.361943774 | 4.11401626 | -2.123539034 |
| 4035 | LRP1 | 9.139551352 | 3.306661338 | -1.446064395 |
| 10023 | FRAT1 | 8.820178962 | 2.265590111 | -2.265590111 |
| 7464 | CORO2A | 8.820178962 | 2.591360272 | -1.265590111 |
| 79783 | C7orf10 | 8.758223215 | 1.519818475 | -1.357343778 |
| 11247 | NXPH4 | 8.758223215 | 8.758223215 | -2.203634363 |
| 10382 | TUBB4A | 8.479780264 | 8.479780264 | -1.241375525 |
| 80022 | MYO15B | 8.479780264 | 8.479780264 | -1.241375525 |
| 93233 | CCDC114 | 8.405141463 | 2.572251449 | -3.15721395 |
| 64798 | DEPTOR | 8.405141463 | 8.405141463 | -1.850552611 |
| 113612 | CYP2U1 | 8.405141463 | 2.176322773 | -2.597786541 |
| 83715 | ESPN | 8.321928095 | 2.489038081 | -1.501749132 |
| 25759 | SHC2 | 8.321928095 | 8.321928095 | -1.501749132 |
| 4055 | LTBR | 8.321928095 | 2.489038081 | -2.514573173 |
| 2788 | GNG7 | 8.233619677 | 2.004800986 | -2.004800986 |
| 84152 | PPP1R1B | 8.233619677 | 8.233619677 | -2.985692163 |
| 6820 | SULT2B1 | 8.233619677 | 2.985692163 | -1.413440714 |
| 388886 | FAM211B | 8.139551352 | 1.31937239 | -1.910732662 |
| 4854 | NOTCH3 | 8.038918989 | 8.038918989 | -8.038918989 |
| 54933 | RHBDL2 | 8.038918989 | 2.790991476 | -8.038918989 |
| 64284 | RAB17 | 7.936637939 | 2.688710426 | -2.688710426 |
| 84812 | PLCD4 | 7.936637939 | 1.707819249 | -2.129283017 |
| 51760 | SYT17 | 7.820178962 | 7.820178962 | -2.572251449 |
| 57168 | ASPHD2 | 7.820178962 | 2.572251449 | -2.572251449 |
| 4093 | SMAD9 | 7.820178962 | 1.987288948 | -2.572251449 |
| 4868 | NPHS1 | 7.820178962 | 1.265590111 | -7.820178962 |
| 7592 | ZNF41 | 7.693486957 | 1.464668267 | -1.886132035 |
| 144132 | DNHD1 | 7.693486957 | 1.464668267 | -1.138898106 |
| 9625 | AATK | 7.693486957 | 7.693486957 | -7.693486957 |
| 56521 | DNAJC12 | 7.693486957 | 1.138898106 | -1.464668267 |
| 23416 | KCNH3 | 7.554588852 | 7.554588852 | -1.74723393 |
| 400242 | DICER1-AS1 | 7.554588852 | 2.306661338 | -2.306661338 |
| 342184 | FMN1 | 7.554588852 | 7.554588852 | -1.325770161 |
| 222962 | SLC29A4 | 7.554588852 | 7.554588852 | -2.306661338 |
| 9746 | CLSTN3 | 7.554588852 | 1.721698838 | -1.74723393 |
| 5662 | PSD | 7.554588852 | 7.554588852 | -7.554588852 |
| 8497 | PPFIA4 | 7.554588852 | 7.554588852 | -7.554588852 |
| 9966 | TNFSF15 | 7.554588852 | 1.325770161 | -1.74723393 |
| 3429 | IFI27 | 7.400879436 | 1.172060746 | -1.172060746 |
| 3201 | HOXA4 | 7.400879436 | 2.152951923 | -1.172060746 |
| 139105 | BEND2 | 7.400879436 | 1.172060746 | -7.400879436 |
| 100129518 | LOC100129518 | 7.400879436 | 1.567989422 | -7.400879436 |
| 4760 | NEUROD1 | 7.400879436 | 1.172060746 | -1.172060746 |
| 90019 | SYT8 | 7.400879436 | 7.400879436 | -1.593524514 |
| 27151 | CPAMD8 | 7.400879436 | 7.400879436 | -2.152951923 |
| 9022 | CLIC3 | 7.238404739 | 1.405514725 | -7.238404739 |
| 9056 | SLC7A7 | 7.238404739 | 7.238404739 | -1.431049817 |
| 5333 | PLCD1 | 7.238404739 | 1.009586049 | -7.238404739 |
| 9473 | C1orf38 | 7.238404739 | 1.009586049 | -1.990477226 |
| 1298 | COL9A2 | 7.238404739 | 7.238404739 | -7.238404739 |
| 10316 | NMUR1 | 7.238404739 | 7.238404739 | -1.009586049 |
| 146771 | TCAM1P | 7.238404739 | 1.990477226 | -1.009586049 |
| 594842 | HAS2-AS1 | 7.238404739 | 1.405514725 | -1.431049817 |
| 100506046 | LOC100506046 | 7.238404739 | 7.238404739 | -1.009586049 |
| 259217 | HSPA12A | 7.238404739 | 7.238404739 | -1.990477226 |
| 388152 | LOC388152 | 7.238404739 | 7.238404739 | -7.238404739 |
| 4902 | NRTN | 7.044394119 | 7.044394119 | -7.044394119 |
| 79611 | ACSS3 | 7.044394119 | 7.044394119 | -7.044394119 |
| 344148 | NCKAP5 | 7.044394119 | 7.044394119 | -1.796466606 |
| 105 | ADARB2 | 7.044394119 | 7.044394119 | -7.044394119 |
| 284161 | GDPD1 | 7.044394119 | 7.044394119 | -1.237039197 |
| 338328 | GPIHBP1 | 7.044394119 | 1.211504105 | -7.044394119 |
| 4059 | BCAM | 7.044394119 | 7.044394119 | -7.044394119 |
| 634 | CEACAM1 | 7.044394119 | 1.796466606 | -7.044394119 |
| 158046 | NXNL2 | 7.044394119 | 7.044394119 | -1.796466606 |
| 643180 | CCT6P3 | 7.044394119 | 1.796466606 | -1.237039197 |
| 50619 | DEF6 | 7.044394119 | 7.044394119 | -7.044394119 |
| 126129 | CPT1C | 7.044394119 | 7.044394119 | -1.796466606 |
| 400643 | LOC400643 | 6.820178962 | 1.572251449 | -6.820178962 |
| 9481 | SLC25A27 | 6.820178962 | 6.820178962 | -1.572251449 |
| 100422970 | MIR1273D | 6.820178962 | 6.820178962 | -1.01282404 |
| 768 | CA9 | 6.820178962 | 6.820178962 | -6.820178962 |
| 100134868 | LOC100134868 | 6.820178962 | 1.572251449 | -1.572251449 |
| 55876 | GSDMB | 6.820178962 | 6.820178962 | -6.820178962 |
| 29935 | RPA4 | 6.820178962 | 6.820178962 | -6.820178962 |
| 221806 | VWDE | 6.820178962 | 1.572251449 | -1.572251449 |
| 7379 | UPK2 | 6.820178962 | 6.820178962 | -1.01282404 |
| 90525 | SHF | 6.820178962 | 6.820178962 | -6.820178962 |
| 3039 | HBA1 | 6.820178962 | 1.572251449 | -1.01282404 |
| 79750 | ZNF385D | 6.820178962 | 6.820178962 | -6.820178962 |
| 8382 | NME5 | 6.820178962 | 6.820178962 | -6.820178962 |
| 100132832 | LOC100132832 | 6.820178962 | 1.572251449 | -6.820178962 |
| 79695 | GALNT12 | 6.820178962 | 6.820178962 | -1.01282404 |
| 89846 | FGD3 | 6.820178962 | 1.572251449 | -6.820178962 |
| 283551 | C14orf182 | 6.820178962 | 6.820178962 | -1.01282404 |
| 9363 | RAB33A | 6.554588852 | 6.554588852 | -6.554588852 |
| 5327 | PLAT | 6.554588852 | 6.554588852 | -1.306661338 |
| 5313 | PKLR | 6.554588852 | 6.554588852 | -6.554588852 |
| 114897 | C1QTNF1 | 6.554588852 | 6.554588852 | -1.306661338 |
| 7781 | SLC30A3 | 6.554588852 | 6.554588852 | -1.306661338 |
| 1815 | DRD4 | 6.554588852 | 6.554588852 | -6.554588852 |
| 25802 | LMOD1 | 6.554588852 | 6.554588852 | -6.554588852 |
| 4137 | MAPT | 6.554588852 | 6.554588852 | -6.554588852 |
| 80725 | SRCIN1 | 6.554588852 | 6.554588852 | -6.554588852 |
| 5657 | PRTN3 | 6.554588852 | 6.554588852 | -1.306661338 |
| 23508 | TTC9 | 6.554588852 | 1.306661338 | -6.554588852 |
| 55512 | SMPD3 | 6.554588852 | 6.554588852 | -6.554588852 |
| 122945 | NOXRED1 | 6.554588852 | 6.554588852 | -6.554588852 |
| 728743 | LOC728743 | 6.554588852 | 1.306661338 | -6.554588852 |
| 401106 | FLJ34208 | 6.554588852 | 6.554588852 | -6.554588852 |
| 283731 | LOC283731 | 6.554588852 | 6.554588852 | -1.306661338 |
| 54518 | APBB1IP | 6.554588852 | 6.554588852 | -6.554588852 |
| 386597 | LOC386597 | 6.554588852 | 6.554588852 | -6.554588852 |
| 57047 | PLSCR2 | 6.554588852 | 6.554588852 | -6.554588852 |
| 80115 | BAIAP2L2 | 6.554588852 | 6.554588852 | -6.554588852 |
| 79369 | B3GNT4 | 6.554588852 | 6.554588852 | -1.306661338 |
| 113763 | C7orf29 | 6.554588852 | 6.554588852 | -6.554588852 |
| 284677 | C1orf204 | 6.554588852 | 6.554588852 | -6.554588852 |
| 11277 | TREX1 | 6.554588852 | 6.554588852 | -6.554588852 |
| 7773 | ZNF230 | 6.554588852 | 6.554588852 | -6.554588852 |
| 27120 | DKKL1 | 6.554588852 | 6.554588852 | -6.554588852 |
| 146540 | ZNF785 | 6.554588852 | 6.554588852 | -6.554588852 |
| 100128569 | C19orf71 | 6.554588852 | 6.554588852 | -6.554588852 |
| 79883 | PODNL1 | 6.554588852 | 6.554588852 | -6.554588852 |
| 644596 | LINC00087 | 6.554588852 | 6.554588852 | -1.306661338 |
| 79805 | VASH2 | 6.22881869 | 6.22881869 | -6.22881869 |
| 56971 | CEACAM19 | 6.22881869 | 6.22881869 | -6.22881869 |
| 100192386 | FLJ16779 | 6.22881869 | 6.22881869 | -6.22881869 |
| 100507266 | LOC100507266 | 6.22881869 | 6.22881869 | -6.22881869 |
| 135932 | TMEM139 | 6.22881869 | 6.22881869 | -6.22881869 |
| 83982 | IFI27L2 | 6.22881869 | 6.22881869 | -6.22881869 |
| 8626 | TP63 | 6.22881869 | 6.22881869 | -6.22881869 |
| 9381 | OTOF | 6.22881869 | 6.22881869 | -6.22881869 |
| 84448 | ABLIM2 | 6.22881869 | 6.22881869 | -6.22881869 |
| 9535 | GMFG | 6.22881869 | 6.22881869 | -6.22881869 |
| 6861 | SYT5 | 6.22881869 | 6.22881869 | -6.22881869 |
| 2737 | GLI3 | 6.22881869 | 6.22881869 | -6.22881869 |
| 56606 | SLC2A9 | 6.22881869 | 6.22881869 | -6.22881869 |
| 83538 | TTC25 | 6.22881869 | 6.22881869 | -6.22881869 |
| 8630 | HSD17B6 | 6.22881869 | 6.22881869 | -6.22881869 |
| 129804 | FBLN7 | 6.22881869 | 6.22881869 | -6.22881869 |
| 9900 | SV2A | 6.22881869 | 6.22881869 | -6.22881869 |
| 282973 | JAKMIP3 | 6.22881869 | 6.22881869 | -6.22881869 |
| 28514 | DLL1 | 6.22881869 | 6.22881869 | -6.22881869 |
| 51334 | PRR16 | 6.22881869 | 6.22881869 | -6.22881869 |
| 54103 | PION | 6.22881869 | 6.22881869 | -6.22881869 |
| 84699 | CREB3L3 | 6.22881869 | 6.22881869 | -6.22881869 |
| 79955 | PDZD7 | 6.22881869 | 6.22881869 | -6.22881869 |
| 729522 | AACSP1 | 6.22881869 | 6.22881869 | -6.22881869 |
| 162962 | ZNF836 | 6.22881869 | 6.22881869 | -6.22881869 |
| 130733 | TMEM178 | 6.22881869 | 6.22881869 | -6.22881869 |
| 7078 | TIMP3 | 6.22881869 | 6.22881869 | -6.22881869 |
| 23566 | LPAR3 | 6.22881869 | 6.22881869 | -6.22881869 |
| 221481 | ARMC12 | 6.22881869 | 6.22881869 | -6.22881869 |
| 100133957 | LOC100133957 | 6.22881869 | 6.22881869 | -6.22881869 |
| 85301 | COL27A1 | 6.22881869 | 6.22881869 | -6.22881869 |
| 54502 | RBM47 | 6.22881869 | 6.22881869 | -6.22881869 |
| 120071 | GYLTL1B | 6.22881869 | 6.22881869 | -6.22881869 |
| 641455 | POTEM | 6.22881869 | 6.22881869 | -6.22881869 |
| 54413 | NLGN3 | 6.22881869 | 6.22881869 | -6.22881869 |
| 5922 | RASA2 | 5.807354922 | 5.807354922 | -5.807354922 |
| 100302746 | NCRUPAR | 5.807354922 | 5.807354922 | -5.807354922 |
| 3563 | IL3RA | 5.807354922 | 5.807354922 | -5.807354922 |
| 3780 | KCNN1 | 5.807354922 | 5.807354922 | -5.807354922 |
| 26257 | NKX2-8 | 5.807354922 | 5.807354922 | -5.807354922 |
| 3208 | HPCA | 5.807354922 | 5.807354922 | -5.807354922 |
| 4610 | MYCL1 | 5.807354922 | 5.807354922 | -5.807354922 |
| 440299 | DNM1P41 | 5.807354922 | 5.807354922 | -5.807354922 |
| 8736 | MYOM1 | 5.807354922 | 5.807354922 | -5.807354922 |
| 145200 | LINC00239 | 5.807354922 | 5.807354922 | -5.807354922 |
| 100216001 | LOC100216001 | 5.807354922 | 5.807354922 | -5.807354922 |
| 51285 | RASL12 | 5.807354922 | 5.807354922 | -5.807354922 |
| 7503 | XIST | 5.807354922 | 5.807354922 | -5.807354922 |
| 1400 | CRMP1 | 5.807354922 | 5.807354922 | -5.807354922 |
| 100128124 | HGC6.3 | 5.807354922 | 5.807354922 | -5.807354922 |
| 27019 | DNAI1 | 5.807354922 | 5.807354922 | -5.807354922 |
| 440896 | LOC440896 | 5.807354922 | 5.807354922 | -5.807354922 |
| 128344 | C1orf88 | 5.807354922 | 5.807354922 | -5.807354922 |
| 2894 | GRID1 | 5.807354922 | 5.807354922 | -5.807354922 |
| 972 | CD74 | 5.807354922 | 5.807354922 | -5.807354922 |
| 160287 | LDHAL6A | 5.807354922 | 5.807354922 | -5.807354922 |
| 80258 | EFHC2 | 5.807354922 | 5.807354922 | -5.807354922 |
| 3777 | KCNK3 | 5.807354922 | 5.807354922 | -5.807354922 |
| 9547 | CXCL14 | 5.807354922 | 5.807354922 | -5.807354922 |
| 374407 | DNAJB13 | 5.807354922 | 5.807354922 | -5.807354922 |
| 80975 | TMPRSS5 | 5.807354922 | 5.807354922 | -5.807354922 |
| 26150 | RIBC2 | 5.807354922 | 5.807354922 | -5.807354922 |
| 54734 | RAB39A | 5.807354922 | 5.807354922 | -5.807354922 |
| 6288 | SAA1 | 5.807354922 | 5.807354922 | -5.807354922 |
| 5880 | RAC2 | 5.807354922 | 5.807354922 | -5.807354922 |
| 6425 | SFRP5 | 5.807354922 | 5.807354922 | -5.807354922 |
| 255101 | CCDC108 | 5.807354922 | 5.807354922 | -5.807354922 |
| 79258 | MMEL1 | 5.807354922 | 5.807354922 | -5.807354922 |
| 149465 | WDR65 | 5.807354922 | 5.807354922 | -5.807354922 |
| 5733 | PTGER3 | 5.807354922 | 5.807354922 | -5.807354922 |
| 9172 | MYOM2 | 5.807354922 | 5.807354922 | -5.807354922 |
| 387597 | ILDR2 | 5.807354922 | 5.807354922 | -5.807354922 |
| 440585 | FAM183A | 5.807354922 | 5.807354922 | -5.807354922 |
| 84879 | MFSD2A | 5.807354922 | 5.807354922 | -5.807354922 |
| 648791 | PPP1R3G | 5.807354922 | 5.807354922 | -5.807354922 |
| 26231 | LRRC29 | 5.807354922 | 5.807354922 | -5.807354922 |
| 195814 | SDR16C5 | 5.807354922 | 5.807354922 | -5.807354922 |
| 1991 | ELANE | 5.807354922 | 5.807354922 | -5.807354922 |
| 326342 | EMR4P | 5.807354922 | 5.807354922 | -5.807354922 |
| 432 | ASGR1 | 5.807354922 | 5.807354922 | -5.807354922 |
| 9914 | ATP2C2 | 5.807354922 | 5.807354922 | -5.807354922 |
| 8608 | RDH16 | 5.807354922 | 5.807354922 | -5.807354922 |
| 442421 | PTGER4P2 | 5.807354922 | 5.807354922 | -5.807354922 |
| 4311 | MME | 5.807354922 | 5.807354922 | -5.807354922 |
| 79935 | CNTD2 | 5.807354922 | 5.807354922 | -5.807354922 |
| 84750 | FUT10 | 5.807354922 | 5.807354922 | -5.807354922 |
| 81563 | C1orf21 | 5.807354922 | 5.807354922 | -5.807354922 |
| 126969 | SLC44A3 | 5.807354922 | 5.807354922 | -5.807354922 |
| 64097 | EPB41L4A | 5.807354922 | 5.807354922 | -5.807354922 |
| 642273 | FAM110C | 5.807354922 | 5.807354922 | -5.807354922 |
| 5452 | POU2F2 | 5.807354922 | 5.807354922 | -5.807354922 |
| 170692 | ADAMTS18 | 5.807354922 | 5.807354922 | -5.807354922 |
| 285588 | EFCAB9 | 5.807354922 | 5.807354922 | -5.807354922 |
| 168507 | PKD1L1 | 5.807354922 | 5.807354922 | -5.807354922 |
| 3386 | ICAM4 | 5.807354922 | 5.807354922 | -5.807354922 |
| 100302119 | MIR1538 | 5.807354922 | 5.807354922 | -5.807354922 |
| 440910 | LOC440910 | 5.807354922 | 5.807354922 | -5.807354922 |
| 9892 | SNAP91 | 5.807354922 | 5.807354922 | -5.807354922 |
| 158067 | AK8 | 5.807354922 | 5.807354922 | -5.807354922 |
| 619505 | SNORA21 | 5.807354922 | 5.807354922 | -5.807354922 |
| 146 | ADRA1D | 5.807354922 | 5.807354922 | -5.807354922 |
| 9514 | GAL3ST1 | 5.807354922 | 5.807354922 | -5.807354922 |
| 56271 | BEX4 | 5.807354922 | 5.807354922 | -5.807354922 |
| 51561 | IL23A | 5.807354922 | 5.807354922 | -5.807354922 |
| 285103 | LOC285103 | 5.807354922 | 5.807354922 | -5.807354922 |
| 127579 | DCST2 | 5.807354922 | 5.807354922 | -5.807354922 |
| 79963 | ABCA11P | 5.807354922 | 5.807354922 | -5.807354922 |
| 400866 | LINC00114 | 5.807354922 | 5.807354922 | -5.807354922 |
| 100506211 | MIR210HG | 5.492707774 | 3.841943214 | -1.568607888 |
| 54541 | DDIT4 | 5.343594833 | 2.353117607 | -1.833299132 |
| 54845 | ESRP1 | 5.247927513 | 5.247927513 | -5.247927513 |
| 100190940 | LOC100190940 | 5.247927513 | 5.247927513 | -5.247927513 |
| 7087 | ICAM5 | 5.247927513 | 5.247927513 | -5.247927513 |
| 677777 | SCARNA12 | 5.247927513 | 5.247927513 | -5.247927513 |
| 100130311 | C17orf107 | 5.247927513 | 5.247927513 | -5.247927513 |
| 26266 | SLC13A4 | 5.247927513 | 5.247927513 | -5.247927513 |
| 5625 | PRODH | 5.247927513 | 5.247927513 | -5.247927513 |
| 100132215 | LOC100132215 | 5.247927513 | 5.247927513 | -5.247927513 |
| 100506939 | LOC100506939 | 5.247927513 | 5.247927513 | -5.247927513 |
| 84249 | PSD2 | 5.247927513 | 5.247927513 | -5.247927513 |
| 1644 | DDC | 5.247927513 | 5.247927513 | -5.247927513 |
| 23281 | MTUS2 | 5.247927513 | 5.247927513 | -5.247927513 |
| 57167 | SALL4 | 5.247927513 | 5.247927513 | -5.247927513 |
| 3823 | KLRC3 | 5.247927513 | 5.247927513 | -5.247927513 |
| 100616668 | LOC100616668 | 5.247927513 | 5.247927513 | -5.247927513 |
| 129293 | C2orf89 | 5.247927513 | 5.247927513 | -5.247927513 |
| 197335 | WDR90 | 5.247927513 | 5.247927513 | -5.247927513 |
| 6092 | ROBO2 | 5.247927513 | 5.247927513 | -5.247927513 |
| 128853 | DUSP15 | 5.247927513 | 5.247927513 | -5.247927513 |
| 151835 | CPNE9 | 5.247927513 | 5.247927513 | -5.247927513 |
| 23563 | CHST5 | 5.247927513 | 5.247927513 | -5.247927513 |
| 283796 | GOLGA8IP | 5.247927513 | 5.247927513 | -5.247927513 |
| 51214 | IGF2-AS | 5.247927513 | 5.247927513 | -5.247927513 |
| 375298 | CERKL | 5.247927513 | 5.247927513 | -5.247927513 |
| 4320 | MMP11 | 5.247927513 | 5.247927513 | -5.247927513 |
| 283948 | NHLRC4 | 5.247927513 | 5.247927513 | -5.247927513 |
| 84960 | KIAA1984 | 5.247927513 | 5.247927513 | -5.247927513 |
| 3373 | HYAL1 | 5.247927513 | 5.247927513 | -5.247927513 |
| 1041 | CDSN | 5.247927513 | 5.247927513 | -5.247927513 |
| 5608 | MAP2K6 | 5.247927513 | 5.247927513 | -5.247927513 |
| 339674 | BK250D10.8 | 5.247927513 | 5.247927513 | -5.247927513 |
| 8743 | TNFSF10 | 5.247927513 | 5.247927513 | -5.247927513 |
| 2073 | ERCC5 | 5.247927513 | 5.247927513 | -5.247927513 |
| 152024 | LOC152024 | 5.247927513 | 5.247927513 | -5.247927513 |
| 692215 | SNORD112 | 5.247927513 | 5.247927513 | -5.247927513 |
| 100233209 | LOC100233209 | 5.247927513 | 5.247927513 | -5.247927513 |
| 6401 | SELE | 5.247927513 | 5.247927513 | -5.247927513 |
| 147670 | LOC147670 | 5.247927513 | 5.247927513 | -5.247927513 |
| 100422948 | MIR4284 | 5.247927513 | 5.247927513 | -5.247927513 |
| 7018 | TF | 5.247927513 | 5.247927513 | -5.247927513 |
| 151651 | EFHB | 5.247927513 | 5.247927513 | -5.247927513 |
| 100288123 | LOC100288123 | 5.247927513 | 5.247927513 | -5.247927513 |
| 27443 | CECR2 | 5.247927513 | 5.247927513 | -5.247927513 |
| 4803 | NGF | 5.247927513 | 5.247927513 | -5.247927513 |
| 7368 | UGT8 | 5.247927513 | 5.247927513 | -5.247927513 |
| 100128081 | JAZF1-AS1 | 5.247927513 | 5.247927513 | -5.247927513 |
| 117852 | TRIM78P | 5.247927513 | 5.247927513 | -5.247927513 |
| 100507050 | LOC100507050 | 5.247927513 | 5.247927513 | -5.247927513 |
| 7638 | ZNF221 | 5.247927513 | 5.247927513 | -5.247927513 |
| 653140 | C2orf84 | 5.247927513 | 5.247927513 | -5.247927513 |
| 5837 | PYGM | 5.247927513 | 5.247927513 | -5.247927513 |
| 4248 | MGAT3 | 5.247927513 | 5.247927513 | -5.247927513 |
| 29119 | CTNNA3 | 5.247927513 | 5.247927513 | -5.247927513 |
| 2244 | FGB | 5.247927513 | 5.247927513 | -5.247927513 |
| 388815 | LINC00478 | 5.247927513 | 5.247927513 | -5.247927513 |
| 158405 | KIAA1958 | 5.247927513 | 5.247927513 | -5.247927513 |
| 6542 | SLC7A2 | 5.247927513 | 5.247927513 | -5.247927513 |
| 121551 | BTBD11 | 5.247927513 | 5.247927513 | -5.247927513 |
| 29993 | PACSIN1 | 5.247927513 | 5.247927513 | -5.247927513 |
| 10901 | DHRS4 | 5.247927513 | 5.247927513 | -5.247927513 |
| 5738 | PTGFRN | 5.247927513 | 5.247927513 | -5.247927513 |
| 347051 | SLC10A5 | 5.247927513 | 5.247927513 | -5.247927513 |
| 1137 | CHRNA4 | 5.247927513 | 5.247927513 | -5.247927513 |
| 127495 | LRRC39 | 5.247927513 | 5.247927513 | -5.247927513 |
| 56159 | TEX11 | 5.247927513 | 5.247927513 | -5.247927513 |
| 23233 | EXOC6B | 5.247927513 | 5.247927513 | -5.247927513 |
| 2676 | GFRA3 | 5.247927513 | 5.247927513 | -5.247927513 |
| 11320 | MGAT4A | 5.247927513 | 5.247927513 | -5.247927513 |
| 7093 | TLL2 | 5.247927513 | 5.247927513 | -5.247927513 |
| 57468 | SLC12A5 | 5.247927513 | 5.247927513 | -5.247927513 |
| 643965 | TMEM88B | 5.247927513 | 5.247927513 | -5.247927513 |
| 9635 | CLCA2 | 5.247927513 | 5.247927513 | -5.247927513 |
| 8542 | APOL1 | 5.247927513 | 5.247927513 | -5.247927513 |
| 79827 | CLMP | 5.247927513 | 5.247927513 | -5.247927513 |
| 284390 | ZNF763 | 5.247927513 | 5.247927513 | -5.247927513 |
| 220001 | VWCE | 5.247927513 | 5.247927513 | -5.247927513 |
| 2560 | GABRB1 | 5.247927513 | 5.247927513 | -5.247927513 |
| 6854 | SYN2 | 5.247927513 | 5.247927513 | -5.247927513 |
| 100129827 | MRVI1-AS1 | 5.247927513 | 5.247927513 | -5.247927513 |
| 30816 | ERVW-1 | 5.247927513 | 5.247927513 | -5.247927513 |
| 65268 | WNK2 | 5.247927513 | 5.247927513 | -5.247927513 |
| 629 | CFB | 5.247927513 | 5.247927513 | -5.247927513 |
| 100134869 | UBE2Q2P2 | 5.247927513 | 5.247927513 | -5.247927513 |
| 8787 | RGS9 | 5.247927513 | 5.247927513 | -5.247927513 |
| 117583 | PARD3B | 5.247927513 | 5.247927513 | -5.247927513 |
| 482 | ATP1B2 | 5.247927513 | 5.247927513 | -5.247927513 |
| 7429 | VIL1 | 5.247927513 | 5.247927513 | -5.247927513 |
| 222389 | BEND7 | 5.247927513 | 5.247927513 | -5.247927513 |
| 773 | CACNA1A | 5.247927513 | 5.247927513 | -5.247927513 |
| 8358 | HIST1H3B | 5.247927513 | 5.247927513 | -5.247927513 |
| 1238 | CCBP2 | 5.247927513 | 5.247927513 | -5.247927513 |
| 8338 | HIST2H2AC | 5.247927513 | 5.247927513 | -5.247927513 |
| 374786 | EFCAB5 | 5.247927513 | 5.247927513 | -5.247927513 |
| 5603 | MAPK13 | 5.247927513 | 5.247927513 | -5.247927513 |
| 65217 | PCDH15 | 5.247927513 | 5.247927513 | -5.247927513 |
| 100506012 | LOC100506012 | 5.247927513 | 5.247927513 | -5.247927513 |
| 403315 | FAM92A1P2 | 5.247927513 | 5.247927513 | -5.247927513 |
| 100302650 | LOC100302650 | 5.247927513 | 5.247927513 | -5.247927513 |
| 389332 | LOC389332 | 5.247927513 | 5.247927513 | -5.247927513 |
| 5649 | RELN | 5.247927513 | 5.247927513 | -5.247927513 |
| 116443 | GRIN3A | 5.247927513 | 5.247927513 | -5.247927513 |
| 7075 | TIE1 | 5.247927513 | 5.247927513 | -5.247927513 |
| 3781 | KCNN2 | 5.247927513 | 5.247927513 | -5.247927513 |
| 90187 | EMILIN3 | 5.247927513 | 5.247927513 | -5.247927513 |
| 119395 | CALHM3 | 5.247927513 | 5.247927513 | -5.247927513 |
| 401494 | PTPLAD2 | 5.247927513 | 5.247927513 | -5.247927513 |
| 2053 | EPHX2 | 5.247927513 | 5.247927513 | -5.247927513 |
| 123099 | DEGS2 | 5.247927513 | 5.247927513 | -5.247927513 |
| 400830 | DEFB132 | 5.247927513 | 5.247927513 | -5.247927513 |
| 282763 | OR51B5 | 5.247927513 | 5.247927513 | -5.247927513 |
| 401081 | FLJ22763 | 5.247927513 | 5.247927513 | -5.247927513 |
| 116535 | MRGPRF | 5.247927513 | 5.247927513 | -5.247927513 |
| 79132 | DHX58 | 5.247927513 | 5.247927513 | -5.247927513 |
| 79411 | GLB1L | 5.247927513 | 5.247927513 | -5.247927513 |
| 100133144 | UBE2Q2P3 | 5.247927513 | 5.247927513 | -5.247927513 |
| 389813 | C9orf172 | 5.247927513 | 5.247927513 | -5.247927513 |
| 27124 | INPP5J | 5.247927513 | 5.247927513 | -5.247927513 |
| 7454 | WAS | 5.247927513 | 5.247927513 | -5.247927513 |
| 81854 | MGC3771 | 5.247927513 | 5.247927513 | -5.247927513 |
| 84734 | FAM167B | 5.247927513 | 5.247927513 | -5.247927513 |
| 1269 | CNR2 | 5.247927513 | 5.247927513 | -5.247927513 |
| 56477 | CCL28 | 5.247927513 | 5.247927513 | -5.247927513 |
| 3248 | HPGD | 5.247927513 | 5.247927513 | -5.247927513 |
| 692085 | SNORD45C | 5.247927513 | 5.247927513 | -5.247927513 |
| 6374 | CXCL5 | 5.247927513 | 5.247927513 | -5.247927513 |
| 22798 | LAMB4 | 5.247927513 | 5.247927513 | -5.247927513 |
| 25876 | SPEF1 | 5.247927513 | 5.247927513 | -5.247927513 |
| 4629 | MYH11 | 5.247927513 | 5.247927513 | -5.247927513 |
| 4359 | MPZ | 5.247927513 | 5.247927513 | -5.247927513 |
| 650 | BMP2 | 5.247927513 | 5.247927513 | -5.247927513 |
| 117286 | CIB3 | 5.247927513 | 5.247927513 | -5.247927513 |
| 10788 | IQGAP2 | 5.247927513 | 5.247927513 | -5.247927513 |
| 92359 | CRB3 | 5.247927513 | 5.247927513 | -5.247927513 |
| 116844 | LRG1 | 5.247927513 | 5.247927513 | -5.247927513 |
| 164668 | APOBEC3H | 5.247927513 | 5.247927513 | -5.247927513 |
| 79844 | ZDHHC11 | 5.247927513 | 5.247927513 | -5.247927513 |
| 362 | AQP5 | 5.247927513 | 5.247927513 | -5.247927513 |
| 286530 | P2RY8 | 5.247927513 | 5.247927513 | -5.247927513 |
| 374877 | C19orf45 | 5.247927513 | 5.247927513 | -5.247927513 |
| 79012 | CAMKV | 5.247927513 | 5.247927513 | -5.247927513 |
| 389812 | LCN15 | 5.247927513 | 5.247927513 | -5.247927513 |
| 162494 | RHBDL3 | 5.247927513 | 5.247927513 | -5.247927513 |
| 80003 | PCNXL2 | 5.247927513 | 5.247927513 | -5.247927513 |
| 56127 | PCDHB9 | 5.247927513 | 5.247927513 | -5.247927513 |
| 387486 | LINC00320 | 5.247927513 | 5.247927513 | -5.247927513 |
| 153770 | PLAC8L1 | 5.247927513 | 5.247927513 | -5.247927513 |
| 7096 | TLR1 | 5.247927513 | 5.247927513 | -5.247927513 |
| 56171 | DNAH7 | 5.247927513 | 5.247927513 | -5.247927513 |
| 9215 | LARGE | 5.247927513 | 5.247927513 | -5.247927513 |
| 130576 | LYPD6B | 5.247927513 | 5.247927513 | -5.247927513 |
| 441425 | ANKRD20A3 | 5.247927513 | 5.247927513 | -5.247927513 |
| 136853 | SRCRB4D | 5.247927513 | 5.247927513 | -5.247927513 |
| 255057 | C19orf26 | 5.247927513 | 5.247927513 | -5.247927513 |
| 6439 | SFTPB | 5.247927513 | 5.247927513 | -5.247927513 |
| 195977 | ANTXRL | 5.247927513 | 5.247927513 | -5.247927513 |
| 51299 | NRN1 | 5.247927513 | 5.247927513 | -5.247927513 |
| 10417 | SPON2 | 5.247927513 | 5.247927513 | -5.247927513 |
| 5555 | PRH2 | 5.247927513 | 5.247927513 | -5.247927513 |
| 26797 | SNORD52 | 5.247927513 | 5.247927513 | -5.247927513 |
| 255809 | C19orf38 | 5.247927513 | 5.247927513 | -5.247927513 |
| 387742 | FAM99A | 5.247927513 | 5.247927513 | -5.247927513 |
| 84174 | SLA2 | 5.247927513 | 5.247927513 | -5.247927513 |
| 84894 | LINGO1 | 5.247927513 | 5.247927513 | -5.247927513 |
| 100130950 | LOC100130950 | 5.247927513 | 5.247927513 | -5.247927513 |
| 3560 | IL2RB | 5.247927513 | 5.247927513 | -5.247927513 |
| 29765 | TMOD4 | 5.247927513 | 5.247927513 | -5.247927513 |
| 389084 | C2orf82 | 5.247927513 | 5.247927513 | -5.247927513 |
| 83650 | SLC35G5 | 5.247927513 | 5.247927513 | -5.247927513 |
| 123591 | C15orf27 | 5.247927513 | 5.247927513 | -5.247927513 |
| 286367 | LOC286367 | 5.247927513 | 5.247927513 | -5.247927513 |
| 10562 | OLFM4 | 5.247927513 | 5.247927513 | -5.247927513 |
| 92346 | C1orf105 | 5.247927513 | 5.247927513 | -5.247927513 |
| 130574 | LYPD6 | 5.247927513 | 5.247927513 | -5.247927513 |
| 8997 | KALRN | 5.247927513 | 5.247927513 | -5.247927513 |
| 647323 | LOC647323 | 5.247927513 | 5.247927513 | -5.247927513 |
| 151056 | PLB1 | 5.247927513 | 5.247927513 | -5.247927513 |
| 491 | ATP2B2 | 5.247927513 | 5.247927513 | -5.247927513 |
| 100129354 | NRADDP | 5.247927513 | 5.247927513 | -5.247927513 |
| 1588 | CYP19A1 | 5.247927513 | 5.247927513 | -5.247927513 |
| 56920 | SEMA3G | 5.247927513 | 5.247927513 | -5.247927513 |
| 56256 | SERTAD4 | 5.247927513 | 5.247927513 | -5.247927513 |
| 6775 | STAT4 | 5.247927513 | 5.247927513 | -5.247927513 |
| 1821 | DRP2 | 5.247927513 | 5.247927513 | -5.247927513 |
| 284100 | LOC284100 | 5.247927513 | 5.247927513 | -5.247927513 |
| 8326 | FZD9 | 5.247927513 | 5.247927513 | -5.247927513 |
| 114599 | SNORD15B | 5.247927513 | 5.247927513 | -5.247927513 |
| 145820 | LOC145820 | 5.247927513 | 5.247927513 | -5.247927513 |
| 196968 | DNM1P46 | 5.247927513 | 5.247927513 | -5.247927513 |
| 54718 | BTN2A3P | 5.247927513 | 5.247927513 | -5.247927513 |
| 146439 | CCDC64B | 5.247927513 | 5.247927513 | -5.247927513 |
| 11309 | SLCO2B1 | 5.247927513 | 5.247927513 | -5.247927513 |
| 654466 | KGFLP2 | 5.247927513 | 5.247927513 | -5.247927513 |
| 5176 | SERPINF1 | 5.247927513 | 5.247927513 | -5.247927513 |
| 55613 | MTMR8 | 5.247927513 | 5.247927513 | -5.247927513 |
| 4858 | NOVA2 | 5.247927513 | 5.247927513 | -5.247927513 |
| 123904 | NRN1L | 5.247927513 | 5.247927513 | -5.247927513 |
| 440300 | LOC440300 | 5.247927513 | 5.247927513 | -5.247927513 |
| 54436 | SH3TC1 | 5.247927513 | 5.247927513 | -5.247927513 |
| 375287 | RBM43 | 5.247927513 | 5.247927513 | -5.247927513 |
| 56000 | NXF3 | 5.247927513 | 5.247927513 | -5.247927513 |
| 388697 | HRNR | 5.247927513 | 5.247927513 | -5.247927513 |
| 2868 | GRK4 | 5.247927513 | 5.247927513 | -5.247927513 |
| 284805 | C20orf203 | 5.247927513 | 5.247927513 | -5.247927513 |
| 145226 | RDH12 | 5.247927513 | 5.247927513 | -5.247927513 |
| 132158 | GLYCTK | 5.247927513 | 5.247927513 | -5.247927513 |
| 2529 | FUT7 | 5.247927513 | 5.247927513 | -5.247927513 |
| 440078 | FAM66C | 5.247927513 | 5.247927513 | -5.247927513 |
| 390195 | OR5AN1 | 5.247927513 | 5.247927513 | -5.247927513 |
| 786 | CACNG1 | 5.247927513 | 5.247927513 | -5.247927513 |
| 284186 | TMEM105 | 5.247927513 | 5.247927513 | -5.247927513 |
| 440836 | ODF3B | 5.247927513 | 5.247927513 | -5.247927513 |
| 133491 | C5orf47 | 5.247927513 | 5.247927513 | -5.247927513 |
| 80307 | FER1L4 | 4.609531222 | 3.949467266 | -1.386745535 |
| 12 | SERPINA3 | 4.473317145 | 2.544090219 | -1.209323819 |
| 79661 | NEIL1 | 4.233871918 | 3.648909418 | -1.545161493 |
| 6038 | RNASE4 | 4.195015982 | 1.506305557 | -1.506305557 |
| 5163 | PDK1 | 4.091981858 | 1.691443929 | -1.695091706 |
| 51754 | TMEM8B | 3.918863237 | 2.48781342 | -1.100509316 |
| 5064 | PALM | 3.862616696 | 3.536846535 | -2.154797447 |
| 80221 | ACSF2 | 3.844829627 | 3.259867127 | -1.156119202 |
| 388228 | SBK1 | 3.749151133 | 1.411847812 | -1.322886378 |
| 168544 | ZNF467 | 3.740757173 | 8.988684687 | -1.052046748 |
| 123688 | AGPHD1 | 3.740757173 | 2.434095835 | -1.295197729 |
| 230 | ALDOC | 3.712228916 | 4.120868644 | -1.121666815 |
| 729359 | PLIN4 | 3.685763142 | 3.685763142 | -8.933690655 |
| 64122 | FN3K | 3.500621586 | 2.500621586 | -1.627313591 |
| 1731 | 1-Sep | 3.445559444 | 1.873307995 | -1.292607521 |
| 57715 | SEMA4G | 3.426264755 | 3.400729663 | -1.094068324 |
| 2921 | CXCL3 | 3.37778133 | 1.805529881 | -2.071119991 |
| 4883 | NPR3 | 3.285402219 | 1.156119202 | -1.272578179 |
| 5582 | PRKCG | 3.285402219 | 1.683366205 | -1.048363022 |
| 27165 | GLS2 | 3.231852751 | 2.64689025 | -1.435386145 |
| 5210 | PFKFB4 | 3.154735122 | 2.59964315 | -1.207421546 |
| 4303 | FOXO4 | 3.074000581 | 1.083523356 | -2.514573173 |
| 80164 | FLJ22184 | 3.069162025 | 2.647698256 | -1.638112207 |
| 219654 | ZCCHC24 | 3.027389998 | 2.020194497 | -1.562721731 |
| 26232 | FBXO2 | 3.019058336 | 1.166379423 | -1.091847756 |
| 197257 | LDHD | 2.985692163 | 1.679030825 | -1.679030825 |
| 7108 | TM7SF2 | 2.975798941 | 2.970323811 | -1.07955288 |
| 10675 | CSPG5 | 2.92721058 | 3.252980741 | -1.159871337 |
| 1373 | CPS1 | 2.891623839 | 2.306661338 | -1.584962501 |
| 54828 | BCAS3 | 2.886132035 | 1.649092838 | -1.138898106 |
| 89848 | FCHSD1 | 2.815199939 | 2.028443978 | -1.222914097 |
| 83546 | RTBDN | 2.790991476 | 2.206028975 | -1.484330138 |
| 322 | APBB1 | 2.754792591 | 1.724365557 | -1.214064062 |
| 25837 | RAB26 | 2.753708348 | 2.474848975 | -1.676264263 |
| 80235 | PIGZ | 2.723860607 | 1.458270496 | -1.138898106 |
| 4987 | OPRL1 | 2.69945729 | 1.475242133 | -1.286016576 |
| 84698 | CAPS2 | 2.688710426 | 1.382049087 | -7.936637939 |
| 26297 | SERGEF | 2.574657492 | 1.114759209 | -1.081549928 |
| 3643 | INSR | 2.572251449 | 1.591360272 | -1.265590111 |
| 283120 | H19 | 2.562706002 | 3.024811755 | -1.6395216 |
| 85315 | PAQR8 | 2.56175511 | 2.071949842 | -1.358120747 |
| 1999 | ELF3 | 2.503179096 | 1.646658117 | -1.01226673 |
| 10014 | HDAC5 | 2.478406399 | 2.755515991 | -1.610318075 |
| 348487 | FAM131C | 2.458270496 | 2.040044719 | -1.341811519 |
| 57326 | PBXIP1 | 2.447743504 | 1.522193453 | -2.294034089 |
| 150291 | MORC2-AS1 | 2.445559444 | 7.693486957 | -1.886132035 |
| 5144 | PDE4D | 2.445559444 | 1.860596943 | -7.693486957 |
| 8398 | PLA2G6 | 2.445559444 | 1.860596943 | -1.138898106 |
| 4329 | ALDH6A1 | 2.426264755 | 2.985692163 | -1.189225557 |
| 125058 | TBC1D16 | 2.426264755 | 2.985692163 | -2.985692163 |
| 4355 | MPP2 | 2.426264755 | 2.004800986 | -1.413440714 |
| 1831 | TSC22D3 | 2.413440714 | 2.189225557 | -1.296981738 |
| 50651 | SLC45A1 | 2.409520494 | 3.214124805 | -1.749456538 |
| 83543 | AIF1L | 2.387023123 | 1.090935033 | -2.233313708 |
| 64764 | CREB3L2 | 2.379101803 | 1.524299719 | -1.379101803 |
| 566 | AZU1 | 2.373570882 | 2.625109649 | -1.493865116 |
| 534 | ATP6V1G2 | 2.33219643 | 2.891623839 | -2.891623839 |
| 489 | ATP2A3 | 2.328914525 | 2.321928095 | -1.389706209 |
| 23604 | DAPK2 | 2.325770161 | 1.316184112 | -2.325770161 |
| 64077 | LHPP | 2.324486822 | 1.131041654 | -1.065370365 |
| 116843 | SLC18B1 | 2.306661338 | 1.325770161 | -2.306661338 |
| 79917 | MAGIX | 2.306661338 | 1.325770161 | -1.325770161 |
| 27076 | LYPD3 | 2.306661338 | 7.554588852 | -1.74723393 |
| 41 | ASIC1 | 2.282933963 | 1.954097499 | -1.140193791 |
| 11156 | PTP4A3 | 2.272578179 | 2.86393845 | -1.399270183 |
| 25809 | TTLL1 | 2.250961574 | 1.925191412 | -3.231852751 |
| 9886 | RHOBTB1 | 2.250961574 | 1.925191412 | -1.241375525 |
| 84189 | SLITRK6 | 2.231564067 | 8.038918989 | -2.231564067 |
| 9415 | FADS2 | 2.230040523 | 2.103379209 | -1.823092571 |
| 9096 | TBX18 | 2.189707614 | 1.106771273 | -1.770408087 |
| 2264 | FGFR4 | 2.176322773 | 8.405141463 | -3.15721395 |
| 7754 | ZNF204P | 2.176322773 | 1.850552611 | -1.360747344 |
| 112611 | RWDD2A | 2.168505724 | 1.579293751 | -1.434095835 |
| 55200 | PLEKHG6 | 2.152951923 | 1.172060746 | -1.593524514 |
| 9980 | DOPEY2 | 2.152951923 | 7.400879436 | -1.172060746 |
| 3202 | HOXA5 | 2.152951923 | 2.152951923 | -7.400879436 |
| 51129 | ANGPTL4 | 2.151330971 | 1.17973761 | -2.123472874 |
| 58191 | CXCL16 | 2.129283017 | 1.116458977 | -1.116458977 |
| 29121 | CLEC2D | 2.113511693 | 1.524299719 | -2.113511693 |
| 9744 | ACAP1 | 2.101402032 | 2.74350844 | -1.554282883 |
| 2941 | GSTA4 | 2.093109404 | 2.093109404 | -1.767339243 |
| 83943 | IMMP2L | 2.071119991 | 1.216317907 | -1.387304104 |
| 11240 | PADI2 | 2.069704549 | 3.390383016 | -1.155995833 |
| 51090 | PLLP | 2.059334139 | 1.399270183 | -1.854352402 |
| 83937 | RASSF4 | 2.049630768 | 2.723860607 | -1.044829781 |
| 55701 | ARHGEF40 | 2.035998198 | 2.181196114 | -1.653949111 |
| 154091 | SLC2A12 | 2.022236474 | 1.187101718 | -2.017974447 |
| 170961 | ANKRD24 | 2.01282404 | 7.820178962 | -2.572251449 |
| 256949 | KANK3 | 2.01282404 | 7.820178962 | -2.572251449 |
| 7137 | TNNI3 | 2.00823615 | 1.105021213 | -1.803254412 |
| 9537 | TP53I11 | 2.003198882 | 1.827060331 | -1.029863845 |
| 64788 | LMF1 | 2.0031977 | 1.845656423 | -1.125627678 |
| 6319 | SCD | 2.001339485 | 2.359059013 | -3.296257746 |
| 10156 | RASA4 | 2 | 3.306661338 | -1.153709415 |
| 158219 | TTC39B | 1.990477226 | 7.238404739 | -1.990477226 |
| 388743 | CAPN8 | 1.990477226 | 7.238404739 | -1.009586049 |
| 10148 | EBI3 | 1.990477226 | 1.009586049 | -7.238404739 |
| 5787 | PTPRB | 1.990477226 | 7.238404739 | -7.238404739 |
| 85026 | C9orf37 | 1.973495365 | 1.030639272 | -1.723357953 |
| 10610 | ST6GALNAC2 | 1.939208316 | 2.063466419 | -1.932221886 |
| 93 | ACVR2B | 1.938044252 | 1.348832279 | -1.519818475 |
| 11037 | STON1 | 1.925191412 | 2.250961574 | -1.925191412 |
| 80724 | ACAD10 | 1.925191412 | 3.231852751 | -1.241375525 |
| 5033 | P4HA1 | 1.924247778 | 2.195621484 | -1.52856858 |
| 644172 | LOC644172 | 1.910732662 | 8.139551352 | -1.095157233 |
| 22846 | VASH1 | 1.910732662 | 8.139551352 | -1.31937239 |
| 25850 | ZNF345 | 1.886132035 | 2.445559444 | -2.445559444 |
| 284751 | LOC284751 | 1.886132035 | 2.445559444 | -1.886132035 |
| 6289 | SAA2 | 1.886132035 | 1.138898106 | -1.886132035 |
| 414918 | FAM116B | 1.886132035 | 7.693486957 | -7.693486957 |
| 54800 | KLHL24 | 1.869939459 | 2.777961137 | -1.632763222 |
| 23179 | RGL1 | 1.86393845 | 1.272578179 | -1.854352402 |
| 10279 | PRSS16 | 1.857165222 | 1.967723597 | -1.029679345 |
| 153768 | PRELID2 | 1.850552611 | 1.850552611 | -2.176322773 |
| 9509 | ADAMTS2 | 1.850552611 | 2.176322773 | -1.166736724 |
| 1299 | COL9A3 | 1.848423172 | 1.993621087 | -1.582833061 |
| 50853 | VILL | 1.843093945 | 2.647698256 | -1.638112207 |
| 6528 | SLC5A5 | 1.811927652 | 2.063466419 | -1.486157491 |
| 25956 | SEC31B | 1.810843908 | 2.26243714 | -1.019042448 |
| 283870 | C16orf79 | 1.810100299 | 2.206028975 | -8.038918989 |
| 11118 | BTN3A2 | 1.799264335 | 2.240203697 | -1.794139303 |
| 7033 | TFF3 | 1.796466606 | 1.796466606 | -1.796466606 |
| 54854 | FAM83E | 1.796466606 | 1.796466606 | -1.237039197 |
| 55160 | ARHGEF10L | 1.796466606 | 7.044394119 | -1.796466606 |
| 91523 | PCED1B | 1.796466606 | 1.211504105 | -1.237039197 |
| 81621 | KAZALD1 | 1.796466606 | 7.044394119 | -1.237039197 |
| 400619 | LINC00511 | 1.796466606 | 1.796466606 | -1.237039197 |
| 10391 | CORO2B | 1.796466606 | 7.044394119 | -1.237039197 |
| 27112 | FAM155B | 1.796466606 | 7.044394119 | -1.237039197 |
| 66004 | LYNX1 | 1.764105077 | 2.004377233 | -1.637562369 |
| 1365 | CLDN3 | 1.759865996 | 1.052046748 | -1.434095835 |
| 727910 | TLCD2 | 1.752945524 | 1.681392263 | -1.164494038 |
| 8718 | TNFRSF25 | 1.74723393 | 1.325770161 | -1.74723393 |
| 445 | ASS1 | 1.74723393 | 7.554588852 | -7.554588852 |
| 112755 | STX1B | 1.738671916 | 1.31937239 | -1.31937239 |
| 11094 | CACFD1 | 1.734409889 | 1.145197916 | -1.510194732 |
| 64221 | ROBO3 | 1.733117939 | 1.098935364 | -1.189496233 |
| 23492 | CBX7 | 1.729258555 | 1.176180115 | -2.83981693 |
| 9649 | RALGPS1 | 1.71419662 | 2.286380516 | -1.090399726 |
| 11067 | C10orf10 | 1.707819249 | 7.936637939 | -7.936637939 |
| 51450 | PRRX2 | 1.70761477 | 2.64232331 | -1.140574178 |
| 421 | ARVCF | 1.704871964 | 3.100800641 | -1.113511693 |
| 23108 | RAP1GAP2 | 1.686520172 | 2.172756592 | -1.536744554 |
| 8226 | HDHD1 | 1.679030825 | 1.189225557 | -2.004800986 |
| 7059 | THBS3 | 1.659601302 | 1.659601302 | -8.479780264 |
| 641649 | TMEM91 | 1.659601302 | 1.070389328 | -1.659601302 |
| 23475 | QPRT | 1.659601302 | 2.250961574 | -1.078900828 |
| 949 | SCARB1 | 1.649948304 | 2.430263974 | -3.054994032 |
| 9311 | ASIC3 | 1.640779715 | 1.479416727 | -1.221480189 |
| 978 | CDA | 1.640779715 | 2.812840461 | -1.997265032 |
| 100527964 | LOC100527964 | 1.593524514 | 1.172060746 | -1.593524514 |
| 54756 | IL17RD | 1.593524514 | 7.400879436 | -1.593524514 |
| 100272217 | LOC100272217 | 1.592285842 | 2.071119991 | -2.818353921 |
| 57593 | EBF4 | 1.592285842 | 8.625708843 | -1.071119991 |
| 30008 | EFEMP2 | 1.591948931 | 4.030521945 | -2.458270496 |
| 79777 | ACBD4 | 1.591360272 | 7.820178962 | -7.820178962 |
| 7903 | ST8SIA4 | 1.591360272 | 1.591360272 | -1.591360272 |
| 21 | ABCA3 | 1.58780525 | 1.876679661 | -1.130703692 |
| 57010 | CABP4 | 1.584962501 | 1.584962501 | -1.004262027 |
| 147700 | KLC3 | 1.572251449 | 1.572251449 | -6.820178962 |
| 113451 | ADC | 1.572251449 | 6.820178962 | -1.01282404 |
| 4756 | NEO1 | 1.572251449 | 1.572251449 | -6.820178962 |
| 374500 | THSD1P1 | 1.572251449 | 1.572251449 | -1.572251449 |
| 219731 | LOC219731 | 1.572251449 | 6.820178962 | -1.572251449 |
| 204801 | NLRP11 | 1.572251449 | 6.820178962 | -1.572251449 |
| 27294 | DHDH | 1.572251449 | 6.820178962 | -1.572251449 |
| 10850 | CCL27 | 1.572251449 | 6.820178962 | -1.01282404 |
| 149840 | C20orf196 | 1.572251449 | 6.820178962 | -1.572251449 |
| 150379 | PNPLA5 | 1.572251449 | 6.820178962 | -1.572251449 |
| 257407 | C2orf72 | 1.572251449 | 6.820178962 | -1.572251449 |
| 53358 | SHC3 | 1.572251449 | 6.820178962 | -6.820178962 |
| 6376 | CX3CL1 | 1.548162583 | 1.035514287 | -1.961064337 |
| 79957 | PAQR6 | 1.538168289 | 9.092757141 | -2.048363022 |
| 132241 | RPL32P3 | 1.527737632 | 1.059588796 | -1.063069365 |
| 130612 | TMEM198 | 1.525569572 | 2.250604956 | -1.966508935 |
| 10062 | NR1H3 | 1.52116585 | 1.734409889 | -1.316184112 |
| 1241 | LTB4R | 1.508146904 | 1.759685671 | -2.501749132 |
| 93129 | ORAI3 | 1.504507569 | 1.666982266 | -1.671767328 |
| 140564 | APOBEC3D | 1.501749132 | 2.093109404 | -2.093109404 |
| 56977 | STOX2 | 1.501749132 | 1.501749132 | -2.093109404 |
| 56895 | AGPAT4 | 1.501321186 | 1.851067225 | -1.630499845 |
| 84988 | PPP1R16A | 1.500851546 | 1.142957954 | -1.047800721 |
| 205 | AK4 | 1.485197937 | 1.777198872 | -1.436368444 |
| 83986 | ITFG3 | 1.470243865 | 1.010628311 | -1.959317444 |
| 115330 | GPR146 | 1.464668267 | 7.693486957 | -1.464668267 |
| 9567 | GTPBP1 | 1.447743504 | 1.0252557 | -1.911985001 |
| 2934 | GSN | 1.445312982 | 2.607656212 | -1.116815889 |
| 84445 | LZTS2 | 1.431206436 | 9.361943774 | -1.317549654 |
| 149428 | BNIPL | 1.431049817 | 1.990477226 | -1.990477226 |
| 100287171 | WASH1 | 1.431049817 | 1.405514725 | -1.990477226 |
| 158801 | NKAPP1 | 1.431049817 | 7.238404739 | -1.009586049 |
| 26468 | LHX6 | 1.431049817 | 1.009586049 | -7.238404739 |
| 85442 | KNDC1 | 1.431049817 | 7.238404739 | -7.238404739 |
| 83959 | SLC4A11 | 1.421588834 | 1.705789698 | -2.451922567 |
| 339761 | CYP27C1 | 1.419838486 | 1.671377253 | -1.296981738 |
| 201625 | DNAH12 | 1.419299526 | 2.265590111 | -2.591360272 |
| 151174 | LOC151174 | 1.417702741 | 1.057030945 | -1.774188058 |
| 1512 | CTSH | 1.393707436 | 1.602856622 | -1.034494352 |
| 24142 | NAT6 | 1.385209933 | 1.475242133 | -1.475242133 |
| 160335 | TMTC2 | 1.382049087 | 2.688710426 | -1.382049087 |
| 55620 | STAP2 | 1.376091219 | 1.660947727 | -1.186505941 |
| 64772 | ENGASE | 1.376074344 | 1.288345494 | -1.459867281 |
| 8659 | ALDH4A1 | 1.375039431 | 1.226488081 | -2.32295092 |
| 134429 | STARD4 | 1.371718462 | 2.176322773 | -2.597786541 |
| 9001 | HAP1 | 1.371718462 | 8.405141463 | -3.15721395 |
| 440253 | WHAMMP2 | 1.371718462 | 1.584962501 | -8.405141463 |
| 78991 | PCYOX1L | 1.35166118 | 1.483238438 | -1.808039475 |
| 23151 | GRAMD4 | 1.342583279 | 1.254854429 | -1.105407041 |
| 26470 | SEZ6L2 | 1.34149854 | 1.739986574 | -1.525493765 |
| 10570 | DPYSL4 | 1.327304127 | 2.184563955 | -1.05787195 |
| 2035 | EPB41 | 1.325770161 | 1.145197916 | -1.316184112 |
| 25830 | SULT4A1 | 1.325770161 | 7.554588852 | -2.306661338 |
| 146880 | LOC146880 | 1.325770161 | 1.325770161 | -1.74723393 |
| 116931 | MED12L | 1.31937239 | 1.910732662 | -1.31937239 |
| 56241 | SUSD2 | 1.31937239 | 1.095157233 | -1.584962501 |
| 80345 | ZSCAN16 | 1.306661338 | 6.554588852 | -1.306661338 |
| 80320 | SP6 | 1.306661338 | 1.306661338 | -1.306661338 |
| 154761 | LOC154761 | 1.306661338 | 6.554588852 | -1.306661338 |
| 4804 | NGFR | 1.306661338 | 6.554588852 | -1.306661338 |
| 2969 | GTF2I | 1.306661338 | 6.554588852 | -6.554588852 |
| 84837 | ARHGAP5-AS1 | 1.306661338 | 6.554588852 | -1.306661338 |
| 3910 | LAMA4 | 1.306661338 | 1.306661338 | -1.306661338 |
| 374618 | TEX9 | 1.306661338 | 6.554588852 | -6.554588852 |
| 84144 | SYDE2 | 1.306661338 | 1.306661338 | -6.554588852 |
| 340348 | TSPAN33 | 1.306661338 | 6.554588852 | -6.554588852 |
| 197320 | ZNF778 | 1.306661338 | 6.554588852 | -6.554588852 |
| 379013 | RNF138P1 | 1.306661338 | 1.306661338 | -6.554588852 |
| 4660 | PPP1R12B | 1.302882339 | 3.004800986 | -3.426264755 |
| 687 | KLF9 | 1.302882339 | 2.413440714 | -2.413440714 |
| 63035 | BCORL1 | 1.302675134 | 1.574048841 | -1.166873459 |
| 389792 | IER5L | 1.30256277 | 1.632528184 | -2.296164999 |
| 65010 | SLC26A6 | 1.289302947 | 1.945439063 | -1.761202784 |
| 3306 | HSPA2 | 1.288505093 | 2.093109404 | -3.074000581 |
| 80853 | JHDM1D | 1.265590111 | 7.820178962 | -1.591360272 |
| 51222 | ZNF219 | 1.263034406 | 1.537656786 | -2.362570079 |
| 79641 | ROGDI | 1.257233254 | 1.821648378 | -1.281079996 |
| 26471 | NUPR1 | 1.252307999 | 2.245910228 | -1.582273413 |
| 10188 | TNK2 | 1.243271151 | 1.08979667 | -1.03922131 |
| 8519 | IFITM1 | 1.237039197 | 1.796466606 | -1.237039197 |
| 112703 | FAM71E1 | 1.237039197 | 7.044394119 | -7.044394119 |
| 7348 | UPK1B | 1.237039197 | 7.044394119 | -1.796466606 |
| 284348 | LYPD5 | 1.237039197 | 7.044394119 | -1.237039197 |
| 144486 | LOC144486 | 1.237039197 | 1.796466606 | -1.237039197 |
| 3137 | HLA-J | 1.237039197 | 7.044394119 | -7.044394119 |
| 9902 | MRC2 | 1.232312236 | 1.420008641 | -1.176497655 |
| 1384 | CRAT | 1.22787796 | 2.221480189 | -1.997265032 |
| 79924 | ADM2 | 1.218740027 | 8.038918989 | -2.790991476 |
| 80757 | TMEM121 | 1.218740027 | 8.038918989 | -1.218740027 |
| 285512 | FAM13A-AS1 | 1.218740027 | 1.484330138 | -1.484330138 |
| 57730 | ANKRD36B | 1.217753527 | 1.20967118 | -1.288345494 |
| 3727 | JUND | 1.209199821 | 2.3102103 | -1.703282468 |
| 6405 | SEMA3F | 1.201633861 | 1.078777113 | -1.135831803 |
| 8916 | HERC3 | 1.172060746 | 2.152951923 | -1.172060746 |
| 644890 | MEIG1 | 1.172060746 | 1.172060746 | -7.400879436 |
| 7005 | TEAD3 | 1.172060746 | 1.172060746 | -2.152951923 |
| 5212 | VIT | 1.172060746 | 7.400879436 | -7.400879436 |
| 64711 | HS3ST6 | 1.169147897 | 1.347546473 | -1.749456538 |
| 51626 | DYNC2LI1 | 1.158624969 | 1.522193453 | -1.364807163 |
| 79948 | LPPR3 | 1.158439287 | 2.149781854 | -1.201244897 |
| 3726 | JUNB | 1.158337027 | 1.772445874 | -1.227284381 |
| 91 | ACVR1B | 1.154987834 | 1.096435651 | -1.04807263 |
| 7001 | PRDX2 | 1.146719377 | 1.610623004 | -1.248117329 |
| 5351 | PLOD1 | 1.138900909 | 1.738591612 | -1.145298681 |
| 23043 | TNIK | 1.138898106 | 1.464668267 | -2.445559444 |
| 144193 | AMDHD1 | 1.138898106 | 2.445559444 | -2.445559444 |
| 192683 | SCAMP5 | 1.138898106 | 7.693486957 | -7.693486957 |
| 20 | ABCA2 | 1.134504503 | 2.347165386 | -2.712162203 |
| 2549 | GAB1 | 1.116458977 | 1.382049087 | -2.129283017 |
| 388963 | C2orf81 | 1.116458977 | 2.688710426 | -1.116458977 |
| 80162 | ATHL1 | 1.111929709 | 3.402533432 | -1.061417091 |
| 23373 | CRTC1 | 1.110921814 | 2.105021213 | -1.4870703 |
| 55344 | PLCXD1 | 1.108493933 | 1.31413499 | -1.250004653 |
| 84557 | MAP1LC3A | 1.104656158 | 1.996171528 | -1.261246521 |
| 51171 | HSD17B14 | 1.093109404 | 1.501749132 | -1.088308418 |
| 3783 | KCNN4 | 1.084677715 | 2.078777113 | -1.389706209 |
| 1748 | DLX4 | 1.074962058 | 1.689070904 | -1.581314724 |
| 79671 | NLRX1 | 1.074962058 | 1.996352223 | -1.584049691 |
| 399474 | TMEM200B | 1.071722688 | 1.348832279 | -1.357343778 |
| 256472 | TMEM151A | 1.071435138 | 1.996519431 | -2.756849018 |
| 5934 | RBL2 | 1.071435138 | 1.288345494 | -1.553935605 |
| 100133091 | LOC100133091 | 1.062735755 | 2.056337984 | -1.056337984 |
| 54813 | KLHL28 | 1.057947349 | 2.168505724 | -1.168505724 |
| 1364 | CLDN4 | 1.057947349 | 1.750279947 | -1.168505724 |
| 284207 | METRNL | 1.054786787 | 1.484545792 | -1.584081466 |
| 83862 | TMEM120A | 1.044199804 | 1.298285253 | -1.509252841 |
| 64856 | VWA1 | 1.038462518 | 1.807182804 | -1.608826456 |
| 254102 | EHBP1L1 | 1.031006057 | 2.089981367 | -1.648657176 |
| 140680 | C20orf96 | 1.02888112 | 2.179706022 | -1.182653306 |
| 5155 | PDGFB | 1.023829236 | 1.303403318 | -1.763645303 |
| 4133 | MAP2 | 1.018689073 | 1.31749461 | -1.109836962 |
| 147686 | ZNF418 | 1.01282404 | 6.820178962 | -6.820178962 |
| 7480 | WNT10B | 1.01282404 | 1.572251449 | -1.572251449 |
| 2668 | GDNF | 1.01282404 | 1.572251449 | -6.820178962 |
| 100272228 | LOC100272228 | 1.01282404 | 1.572251449 | -6.820178962 |
| 64838 | FNDC4 | 1.01282404 | 6.820178962 | -1.01282404 |
| 100131187 | TSTD1 | 1.01282404 | 6.820178962 | -1.01282404 |
| 10957 | PNRC1 | 1.012203123 | 1.425269919 | -1.608620695 |
| 9524 | TECR | 1.009879776 | 2.260335587 | -1.668635502 |
| 148979 | GLIS1 | 1.009586049 | 1.990477226 | -1.009586049 |
| 100130275 | LOC100130275 | 1.009586049 | 1.405514725 | -7.238404739 |
| 26048 | ZNF500 | 1.009586049 | 1.009586049 | -1.990477226 |
| 100499227 | LOC100499227 | 1.009586049 | 1.405514725 | -1.990477226 |
| 26160 | IFT172 | 1.004800986 | 2.004800986 | -2.004800986 |
| 55893 | ZNF395 | 1.003838659 | 1.511473151 | -1.382606556 |
| 29108 | PYCARD | -8.813781191 | -10.25974326 | 7.820178962 |
| 11249 | NXPH2 | -7.930737338 | -7.820178962 | 5.247927513 |
| 1415 | CRYBB2 | -7.813781191 | -6.22881869 | 7.238404739 |
| 7562 | ZNF708 | -7.813781191 | -7.409390936 | 6.554588852 |
| 348180 | CTU2 | -7.686500527 | -7.044394119 | 7.400879436 |
| 2707 | GJB3 | -7.686500527 | -7.238404739 | 7.400879436 |
| 55027 | HEATR3 | -7.686500527 | -6.22881869 | 5.247927513 |
| 154 | ADRB2 | -7.554588852 | -5.247927513 | 6.820178962 |
| 79631 | EFTUD1 | -7.400879436 | -6.820178962 | 7.044394119 |
| 7056 | THBD | -7.400879436 | -5.247927513 | 6.554588852 |
| 84504 | NKX6-2 | -7.400879436 | -5.247927513 | 5.247927513 |
| 269 | AMHR2 | -7.22881869 | -6.22881869 | 7.044394119 |
| 401491 | FLJ35024 | -7.22881869 | -9.236014192 | 9.187352073 |
| 399687 | MYO18A | -7.033423002 | -7.562242424 | 6.554588852 |
| 439994 | LOC439994 | -7.033423002 | -7.820178962 | 8.405141463 |
| 201562 | PTPLB | -7.033423002 | -6.820178962 | 7.554588852 |
| 374383 | NCR3LG1 | -7.033423002 | -5.832890014 | 5.247927513 |
| 283518 | KCNRG | -7.033423002 | -7.044394119 | 5.247927513 |
| 161835 | FSIP1 | -7.033423002 | -5.832890014 | 6.554588852 |
| 26272 | FBXO4 | -7.033423002 | -5.247927513 | 6.22881869 |
| 94025 | MUC16 | -7.033423002 | -5.247927513 | 6.22881869 |
| 636 | BICD1 | -6.820178962 | -6.554588852 | 6.22881869 |
| 440138 | ALG11 | -6.820178962 | -6.22881869 | 5.247927513 |
| 55753 | OGDHL | -6.820178962 | -5.832890014 | 6.22881869 |
| 85236 | HIST1H2BK | -6.820178962 | -6.554588852 | 5.247927513 |
| 51729 | WBP11 | -6.820178962 | -7.820178962 | 7.044394119 |
| 653275 | CFC1B | -6.820178962 | -8.144658243 | 6.820178962 |
| 5332 | PLCB4 | -6.820178962 | -6.554588852 | 5.807354922 |
| 6358 | CCL14 | -6.554588852 | -7.693486957 | 6.22881869 |
| 781 | CACNA2D1 | -6.554588852 | -5.247927513 | 5.247927513 |
| 160760 | PPTC7 | -6.554588852 | -7.044394119 | 6.554588852 |
| 165545 | DQX1 | -6.554588852 | -6.22881869 | 5.807354922 |
| 142913 | CFL1P1 | -6.554588852 | -5.247927513 | 5.247927513 |
| 4163 | MCC | -6.554588852 | -7.693486957 | 7.400879436 |
| 1024 | CDK8 | -6.554588852 | -6.554588852 | 6.554588852 |
| 752014 | CEMP1 | -6.554588852 | -5.247927513 | 6.22881869 |
| 26153 | KIF26A | -6.554588852 | -7.044394119 | 6.554588852 |
| 10389 | SCML2 | -6.554588852 | -5.247927513 | 5.247927513 |
| 9609 | RAB36 | -6.554588852 | -5.247927513 | 6.554588852 |
| 27065 | D4S234E | -6.554588852 | -7.044394119 | 6.820178962 |
| 401232 | DKFZP686I15217 | -6.554588852 | -6.22881869 | 7.238404739 |
| 84083 | ZRANB3 | -6.554588852 | -5.247927513 | 5.247927513 |
| 220972 | 8-Mar | -6.554588852 | -5.832890014 | 5.247927513 |
| 6637 | SNRPG | -6.22881869 | -7.936637939 | 5.807354922 |
| 1846 | DUSP4 | -6.22881869 | -6.22881869 | 5.247927513 |
| 55612 | FERMT1 | -6.22881869 | -5.247927513 | 6.554588852 |
| 27350 | APOBEC3C | -6.22881869 | -5.247927513 | 7.936637939 |
| 152485 | ZNF827 | -6.22881869 | -5.832890014 | 7.693486957 |
| 374355 | CCDC172 | -6.22881869 | -6.22881869 | 6.22881869 |
| 57623 | ZFAT | -6.22881869 | -6.22881869 | 6.554588852 |
| 116211 | TM4SF19 | -6.22881869 | -6.22881869 | 6.820178962 |
| 542767 | C1QTNF9B-AS1 | -6.22881869 | -5.247927513 | 5.807354922 |
| 200172 | SLFNL1 | -6.22881869 | -6.554588852 | 6.22881869 |
| 8490 | RGS5 | -6.22881869 | -7.238404739 | 7.400879436 |
| 85359 | DGCR6L | -6.22881869 | -5.832890014 | 5.807354922 |
| 1517 | CTSL1P2 | -5.807354922 | -7.238404739 | 6.554588852 |
| 56126 | PCDHB10 | -5.807354922 | -5.247927513 | 5.807354922 |
| 5029 | P2RY2 | -5.807354922 | -6.22881869 | 6.22881869 |
| 283416 | C12orf61 | -5.807354922 | -5.247927513 | 5.247927513 |
| 1193 | CLIC2 | -5.807354922 | -5.247927513 | 5.247927513 |
| 6795 | AURKC | -5.807354922 | -5.247927513 | 5.247927513 |
| 91683 | SYT12 | -5.807354922 | -5.247927513 | 6.554588852 |
| 100527978 | TMEM56-RWDD3 | -5.807354922 | -7.562242424 | 6.22881869 |
| 7136 | TNNI2 | -5.807354922 | -5.832890014 | 6.22881869 |
| 5729 | PTGDR | -5.807354922 | -7.044394119 | 6.820178962 |
| 84517 | ACTRT3 | -5.247927513 | -8.144658243 | 7.044394119 |
| 90233 | ZNF551 | -5.247927513 | -5.247927513 | 6.554588852 |
| 4117 | MAK | -5.247927513 | -5.247927513 | 5.247927513 |
| 349152 | DPY19L2P2 | -5.247927513 | -6.554588852 | 5.247927513 |
| 624 | BDKRB2 | -5.247927513 | -5.832890014 | 8.139551352 |
| 11213 | IRAK3 | -5.247927513 | -6.22881869 | 5.247927513 |
| 26005 | C2CD3 | -5.247927513 | -5.247927513 | 5.807354922 |
| 169841 | ZNF169 | -5.247927513 | -5.832890014 | 5.807354922 |
| 140686 | WFDC3 | -5.247927513 | -6.22881869 | 5.247927513 |
| 2246 | FGF1 | -5.247927513 | -5.247927513 | 6.22881869 |
| 154664 | ABCA13 | -5.247927513 | -7.820178962 | 7.400879436 |
| 84657 | GHRLOS2 | -5.247927513 | -6.22881869 | 7.044394119 |
| 10861 | SLC26A1 | -5.247927513 | -5.247927513 | 5.247927513 |
| 7004 | TEAD4 | -5.247927513 | -5.247927513 | 6.554588852 |
| 1368 | CPM | -5.247927513 | -6.554588852 | 5.807354922 |
| 347273 | MURC | -5.247927513 | -6.820178962 | 7.044394119 |
| 414189 | AGAP6 | -5.247927513 | -5.247927513 | 5.807354922 |
| 100271831 | - | -5.247927513 | -5.832890014 | 6.22881869 |
| 728927 | ZNF736 | -5.247927513 | -5.832890014 | 5.247927513 |
| 64091 | POPDC2 | -5.247927513 | -6.22881869 | 5.247927513 |
| 1993 | ELAVL2 | -5.247927513 | -7.562242424 | 7.820178962 |
| 146434 | ZNF597 | -5.247927513 | -6.22881869 | 5.807354922 |
| 84733 | CBX2 | -5.247927513 | -5.247927513 | 6.22881869 |
| 283932 | FBXL19-AS1 | -5.247927513 | -5.832890014 | 6.22881869 |
| 3624 | INHBA | -5.247927513 | -5.832890014 | 6.22881869 |
| 114905 | C1QTNF7 | -5.247927513 | -5.832890014 | 6.22881869 |
| 3887 | KRT81 | -5.247927513 | -6.820178962 | 6.554588852 |
| 83856 | FSD1L | -5.247927513 | -7.238404739 | 7.238404739 |
| 100288801 | FRG2C | -5.247927513 | -5.247927513 | 5.247927513 |
| 8360 | HIST1H4D | -5.247927513 | -5.247927513 | 5.247927513 |
| 9711 | KIAA0226 | -3.0694851 | -2.161463423 | 1.990477226 |
| 27063 | ANKRD1 | -2.94760731 | -2.198120447 | 1.507595068 |
| 6650 | SOLH | -2.663970375 | -1.906253504 | 2.421591153 |
| 3352 | HTR1D | -2.612386978 | -2.775796151 | 3.691565814 |
| 9329 | GTF3C4 | -2.584962501 | -1.707819249 | 1.325770161 |
| 79469 | DLEU2L | -2.565853678 | -2.161463423 | 1.796466606 |
| 53354 | PANK1 | -2.481584761 | -2.142378675 | 1.850552611 |
| 7771 | ZFP112 | -2.327071398 | -1.602036014 | 2.514573173 |
| 8863 | PER3 | -2.321928095 | -2.176322773 | 2.004800986 |
| 57460 | PPM1H | -2.318855261 | -1.007653573 | 1.265590111 |
| 729920 | ISPD | -2.306661338 | -1.990477226 | 1.990477226 |
| 9076 | CLDN1 | -2.254670933 | -1.480653638 | 1.565371772 |
| 9469 | CHST3 | -2.24691474 | -1.915839552 | 2.396890153 |
| 151354 | FAM84A | -2.24691474 | -2.176322773 | 1.591360272 |
| 26145 | IRF2BP1 | -2.231564067 | -2.337303321 | 2.514573173 |
| 55765 | C1orf106 | -2.152951923 | -1.796466606 | 1.796466606 |
| 100303728 | SLC25A5-AS1 | -2.152951923 | -3.513623719 | 3.306661338 |
| 221178 | SPATA13 | -2.123382416 | -2.822001698 | 2.426264755 |
| 50964 | SOST | -1.990413951 | -2.699704587 | 2.69676031 |
| 158747 | MOSPD2 | -1.980891177 | -1.572251449 | 2.306661338 |
| 283417 | DPY19L2 | -1.980891177 | -1.306661338 | 1.796466606 |
| 267004 | PGBD3 | -1.921144579 | -1.382049087 | 1.767339243 |
| 54826 | GIN1 | -1.846290585 | -1.382049087 | 2.324994398 |
| 65083 | NOL6 | -1.829115441 | -1.682860144 | 1.927614075 |
| 375444 | C5orf34 | -1.822212881 | -1.068064275 | 1.295197729 |
| 79785 | RERGL | -1.811632394 | -2.206839542 | 2.50973387 |
| 4312 | MMP1 | -1.810100299 | -3.562344198 | 2.464668267 |
| 22824 | HSPA4L | -1.805160709 | -1.206962381 | 1.538168289 |
| 100131691 | LOC100131691 | -1.731765836 | -2.293696279 | 2.695804476 |
| 79165 | LENG1 | -1.730567823 | -1.876788564 | 1.31937239 |
| 23221 | RHOBTB2 | -1.705256734 | -1.498805857 | 1.749456538 |
| 790955 | C11orf83 | -1.701918647 | -1.591360272 | 1.815575429 |
| 114609 | TIRAP | -1.692332598 | -1.523146493 | 1.64117851 |
| 11097 | NUPL2 | -1.692332598 | -2.042366031 | 1.903702318 |
| 7584 | ZNF35 | -1.636164953 | -1.280628466 | 1.692930997 |
| 133584 | EGFLAM | -1.617505874 | -1.842194971 | 1.553538048 |
| 100170220 | SNAR-E | -1.594994654 | -2.733476608 | 2.0541466 |
| 644961 | LOC644961 | -1.593524514 | -1.237039197 | 2.237039197 |
| 158431 | ZNF782 | -1.593524514 | -1.602036014 | 1.01282404 |
| 8320 | EOMES | -1.572251449 | -2.688710426 | 1.306661338 |
| 23764 | MAFF | -1.572251449 | -1.796466606 | 1.796466606 |
| 3398 | ID2 | -1.559199405 | -2.868071347 | 2.141994069 |
| 5090 | PBX3 | -1.518728802 | -2.45467613 | 2.958191824 |
| 205717 | KIAA2018 | -1.511827796 | -1.368348775 | 1.103188068 |
| 1947 | EFNB1 | -1.491591003 | -2.382738862 | 3.013936829 |
| 131034 | CPNE4 | -1.472564676 | -3.006388328 | 2.614535616 |
| 7516 | XRCC2 | -1.457681837 | -1.591360272 | 1.325770161 |
| 90025 | UBE3D | -1.457681837 | -1.464668267 | 1.325770161 |
| 126295 | ZNF57 | -1.457681837 | -1.707819249 | 1.815575429 |
| 284098 | PIGW | -1.457681837 | -1.464668267 | 1.009586049 |
| 9125 | RQCD1 | -1.457681837 | -2.650764559 | 2.093109404 |
| 339500 | ZNF678 | -1.451593232 | -1.088024748 | 1.316184112 |
| 1102 | RCBTB2 | -1.447288436 | -1.741562608 | 1.553935605 |
| 57582 | KCNT1 | -1.408639728 | -2.32447928 | 2.622764533 |
| 83449 | PMFBP1 | -1.393975884 | -2.298034889 | 2.373947403 |
| 100093630 | SNHG8 | -1.367779714 | -2.091010939 | 2.495831128 |
| 22881 | ANKRD6 | -1.353503489 | -2.530219536 | 2.857769286 |
| 256979 | SUN3 | -1.325770161 | -1.591360272 | 2.176322773 |
| 286333 | LINC00256A | -1.306661338 | -1.572251449 | 1.572251449 |
| 55602 | CDKN2AIP | -1.306661338 | -1.796466606 | 1.796466606 |
| 467 | ATF3 | -1.306661338 | -1.572251449 | 1.796466606 |
| 375248 | ANKRD36 | -1.306661338 | -2.161463423 | 1.572251449 |
| 378708 | APITD1 | -1.305028494 | -4.169722076 | 3.509359103 |
| 4814 | NINJ1 | -1.287366882 | -1.760295583 | 2.917357972 |
| 92935 | MARS2 | -1.282080162 | -1.132702537 | 1.426376216 |
| 1032 | CDKN2D | -1.260474166 | -2.193603998 | 2.767339243 |
| 631 | BFSP1 | -1.25919234 | -1.007653573 | 1.265590111 |
| 28964 | GIT1 | -1.2512834 | -1.080687846 | 1.636517689 |
| 253635 | CCDC75 | -1.237328692 | -2.425153365 | 1.316184112 |
| 162998 | OR7D2 | -1.23725038 | -1.003480569 | 1.826149295 |
| 8516 | ITGA8 | -1.23725038 | -3.158262084 | 2.574470127 |
| 5130 | PCYT1A | -1.226068079 | -1.01282404 | 1.237039197 |
| 57343 | ZNF304 | -1.221172383 | -1.478703813 | 1.004262027 |
| 54964 | C1orf56 | -1.218740027 | -1.116458977 | 1.938044252 |
| 387751 | GVINP1 | -1.218740027 | -2.27521806 | 1.584962501 |
| 63916 | ELMO2 | -1.204013892 | -2.600101098 | 3.051570348 |
| 388389 | CCDC103 | -1.19553894 | -1.767070609 | 2.085280096 |
| 84292 | WDR83 | -1.184424571 | -2.402689107 | 2.583139765 |
| 3420 | IDH3B | -1.179652369 | -2.479125355 | 2.63818744 |
| 9404 | LPXN | -1.172060746 | -1.333423734 | 1.325770161 |
| 54414 | SIAE | -1.162474697 | -2.355919865 | 2.083523356 |
| 79415 | C17orf62 | -1.151384309 | -1.750021747 | 1.538866086 |
| 8424 | BBOX1 | -1.150434094 | -1.473931188 | 2.155918143 |
| 100129196 | LOC100129196 | -1.145739574 | -1.867745836 | 2.081288211 |
| 100506677 | AA06 | -1.131911676 | -2.892494375 | 2.071119991 |
| 7701 | ZNF142 | -1.110558375 | -2.32447928 | 2.168505724 |
| 25939 | SAMHD1 | -1.098072123 | -1.720969194 | 1.148433084 |
| 131474 | CHCHD4 | -1.095356974 | -1.421672386 | 1.525329715 |
| 6875 | TAF4B | -1.090032201 | -1.652573407 | 1.277533976 |
| 4998 | ORC1 | -1.085097591 | -1.55378562 | 1.499489348 |
| 642361 | LOC642361 | -1.084590172 | -1.128605817 | 1.457275787 |
| 23166 | STAB1 | -1.07368113 | -2.037570254 | 2.209712328 |
| 340515 | LOC340515 | -1.072332726 | -2.753497756 | 2.731956231 |
| 55113 | XKR8 | -1.04817576 | -1.716083553 | 1.671756963 |
| 64421 | DCLRE1C | -1.040175922 | -1.360704927 | 1.044829781 |
| 4701 | NDUFA7 | -1.01282404 | -1.431049817 | 2.597786541 |

**Table S2. List of 990 DEGs for A^△^ *vs* Y.**

| **Gene ID** | **Symbol** | **log_2_Ratio (Y vs BC)** | **log_2_Ratio (Y vs NC)** | **log_2_Ratio (A^△^ vs Y)** |
| --- | --- | --- | --- | --- |
| 7089 | TLE2 | 9.361943774 | 2.317549654 | -3.554588852 |
| 90993 | CREB3L1 | 9.361943774 | 4.11401626 | -3.133125083 |
| 94015 | TTYH2 | 9.361943774 | 1.668456816 | -2.807354922 |
| 147138 | TMC8 | 9.139551352 | 2.31937239 | -1.325770161 |
| 4035 | LRP1 | 9.139551352 | 3.306661338 | -3.33219643 |
| 1944 | EFNA3 | 8.933690655 | 1.524299719 | -1.119909464 |
| 10023 | FRAT1 | 8.820178962 | 2.265590111 | -2.591360272 |
| 7464 | CORO2A | 8.820178962 | 2.591360272 | -2.01282404 |
| 79783 | C7orf10 | 8.758223215 | 1.519818475 | -1.724800213 |
| 11247 | NXPH4 | 8.758223215 | 8.758223215 | -1.529404524 |
| 11045 | UPK1A | 8.554588852 | 2 | -2.74723393 |
| 10382 | TUBB4A | 8.479780264 | 8.479780264 | -2.672425342 |
| 80022 | MYO15B | 8.479780264 | 8.479780264 | -8.479780264 |
| 165679 | SPTSSB | 8.405141463 | 2.176322773 | -1.597786541 |
| 93233 | CCDC114 | 8.405141463 | 2.572251449 | -8.405141463 |
| 64798 | DEPTOR | 8.405141463 | 8.405141463 | -8.405141463 |
| 113612 | CYP2U1 | 8.405141463 | 2.176322773 | -8.405141463 |
| 83715 | ESPN | 8.321928095 | 2.489038081 | -1.767339243 |
| 50649 | ARHGEF4 | 8.321928095 | 1.501749132 | -1.767339243 |
| 25759 | SHC2 | 8.321928095 | 8.321928095 | -8.321928095 |
| 4055 | LTBR | 8.321928095 | 2.489038081 | -8.321928095 |
| 2788 | GNG7 | 8.233619677 | 2.004800986 | -2.426264755 |
| 90293 | KLHL13 | 8.233619677 | 1.679030825 | -1.426264755 |
| 84152 | PPP1R1B | 8.233619677 | 8.233619677 | -8.233619677 |
| 388886 | FAM211B | 8.139551352 | 1.31937239 | -2.930097987 |
| 4854 | NOTCH3 | 8.038918989 | 8.038918989 | -2.829465624 |
| 54933 | RHBDL2 | 8.038918989 | 2.790991476 | -2.231564067 |
| 64284 | RAB17 | 7.936637939 | 2.688710426 | -7.936637939 |
| 84812 | PLCD4 | 7.936637939 | 1.707819249 | -1.129283017 |
| 163255 | ZNF540 | 7.820178962 | 1.987288948 | -1.591360272 |
| 285908 | LINC00174 | 7.820178962 | 1.265590111 | -7.820178962 |
| 51760 | SYT17 | 7.820178962 | 7.820178962 | -2.610725597 |
| 4868 | NPHS1 | 7.820178962 | 1.265590111 | -1.265590111 |
| 5630 | PRPH | 7.693486957 | 7.693486957 | -7.693486957 |
| 2563 | GABRD | 7.693486957 | 1.860596943 | -1.138898106 |
| 7592 | ZNF41 | 7.693486957 | 1.464668267 | -1.138898106 |
| 1066 | CES1 | 7.693486957 | 1.138898106 | -1.464668267 |
| 144132 | DNHD1 | 7.693486957 | 1.464668267 | -1.886132035 |
| 151473 | SLC16A14 | 7.693486957 | 1.464668267 | -7.693486957 |
| 9625 | AATK | 7.693486957 | 7.693486957 | -2.484033592 |
| 23416 | KCNH3 | 7.554588852 | 7.554588852 | -7.554588852 |
| 400242 | DICER1-AS1 | 7.554588852 | 2.306661338 | -1.74723393 |
| 342184 | FMN1 | 7.554588852 | 7.554588852 | -1.74723393 |
| 222962 | SLC29A4 | 7.554588852 | 7.554588852 | -7.554588852 |
| 643641 | ZNF862 | 7.554588852 | 7.554588852 | -7.554588852 |
| 9746 | CLSTN3 | 7.554588852 | 1.721698838 | -2.345135486 |
| 653160 | LOC653160 | 7.554588852 | 2.306661338 | -1.325770161 |
| 10384 | BTN3A3 | 7.554588852 | 2.306661338 | -2.345135486 |
| 5662 | PSD | 7.554588852 | 7.554588852 | -7.554588852 |
| 8497 | PPFIA4 | 7.554588852 | 7.554588852 | -7.554588852 |
| 9966 | TNFSF15 | 7.554588852 | 1.325770161 | -7.554588852 |
| 284069 | FAM171A2 | 7.400879436 | 7.400879436 | -7.400879436 |
| 139728 | PNCK | 7.400879436 | 7.400879436 | -2.191426071 |
| 3201 | HOXA4 | 7.400879436 | 2.152951923 | -2.191426071 |
| 139105 | BEND2 | 7.400879436 | 1.172060746 | -2.191426071 |
| 10761 | PLAC1 | 7.400879436 | 1.567989422 | -7.400879436 |
| 51279 | C1RL | 7.400879436 | 1.567989422 | -2.191426071 |
| 79603 | CERS4 | 7.400879436 | 1.172060746 | -7.400879436 |
| 100129518 | LOC100129518 | 7.400879436 | 1.567989422 | -1.172060746 |
| 4760 | NEUROD1 | 7.400879436 | 1.172060746 | -7.400879436 |
| 90019 | SYT8 | 7.400879436 | 7.400879436 | -1.172060746 |
| 27151 | CPAMD8 | 7.400879436 | 7.400879436 | -7.400879436 |
| 199699 | DAND5 | 7.400879436 | 1.172060746 | -7.400879436 |
| 9022 | CLIC3 | 7.238404739 | 1.405514725 | -1.431049817 |
| 9056 | SLC7A7 | 7.238404739 | 7.238404739 | -7.238404739 |
| 5333 | PLCD1 | 7.238404739 | 1.009586049 | -2.028951374 |
| 9473 | C1orf38 | 7.238404739 | 1.009586049 | -2.028951374 |
| 1298 | COL9A2 | 7.238404739 | 7.238404739 | -7.238404739 |
| 3934 | LCN2 | 7.238404739 | 7.238404739 | -7.238404739 |
| 5740 | PTGIS | 7.238404739 | 7.238404739 | -7.238404739 |
| 84440 | RAB11FIP4 | 7.238404739 | 7.238404739 | -1.009586049 |
| 221421 | RSPH9 | 7.238404739 | 1.009586049 | -2.028951374 |
| 10316 | NMUR1 | 7.238404739 | 7.238404739 | -7.238404739 |
| 146771 | TCAM1P | 7.238404739 | 1.990477226 | -7.238404739 |
| 100506046 | LOC100506046 | 7.238404739 | 7.238404739 | -1.431049817 |
| 259217 | HSPA12A | 7.238404739 | 7.238404739 | -7.238404739 |
| 388152 | LOC388152 | 7.238404739 | 7.238404739 | -7.238404739 |
| 4902 | NRTN | 7.044394119 | 7.044394119 | -7.044394119 |
| 79611 | ACSS3 | 7.044394119 | 7.044394119 | -7.044394119 |
| 105 | ADARB2 | 7.044394119 | 7.044394119 | -7.044394119 |
| 3108 | HLA-DMA | 7.044394119 | 7.044394119 | -7.044394119 |
| 284161 | GDPD1 | 7.044394119 | 7.044394119 | -1.834940754 |
| 58473 | PLEKHB1 | 7.044394119 | 1.211504105 | -7.044394119 |
| 338328 | GPIHBP1 | 7.044394119 | 1.211504105 | -7.044394119 |
| 54760 | PCSK4 | 7.044394119 | 1.796466606 | -1.834940754 |
| 390664 | C1QTNF8 | 7.044394119 | 1.211504105 | -7.044394119 |
| 634 | CEACAM1 | 7.044394119 | 1.796466606 | -7.044394119 |
| 10076 | PTPRU | 7.044394119 | 1.796466606 | -7.044394119 |
| 158046 | NXNL2 | 7.044394119 | 7.044394119 | -7.044394119 |
| 643180 | CCT6P3 | 7.044394119 | 1.796466606 | -1.834940754 |
| 50619 | DEF6 | 7.044394119 | 7.044394119 | -7.044394119 |
| 126129 | CPT1C | 7.044394119 | 7.044394119 | -7.044394119 |
| 400643 | LOC400643 | 6.820178962 | 1.572251449 | -1.610725597 |
| 100422970 | MIR1273D | 6.820178962 | 6.820178962 | -6.820178962 |
| 768 | CA9 | 6.820178962 | 6.820178962 | -6.820178962 |
| 55876 | GSDMB | 6.820178962 | 6.820178962 | -6.820178962 |
| 29935 | RPA4 | 6.820178962 | 6.820178962 | -6.820178962 |
| 60468 | BACH2 | 6.820178962 | 6.820178962 | -1.610725597 |
| 114818 | KLHL29 | 6.820178962 | 1.572251449 | -6.820178962 |
| 221806 | VWDE | 6.820178962 | 1.572251449 | -1.610725597 |
| 7379 | UPK2 | 6.820178962 | 6.820178962 | -6.820178962 |
| 160428 | ALDH1L2 | 6.820178962 | 1.572251449 | -1.610725597 |
| 440356 | LOC440356 | 6.820178962 | 6.820178962 | -6.820178962 |
| 90525 | SHF | 6.820178962 | 6.820178962 | -1.610725597 |
| 79750 | ZNF385D | 6.820178962 | 6.820178962 | -6.820178962 |
| 100132832 | LOC100132832 | 6.820178962 | 1.572251449 | -6.820178962 |
| 89846 | FGD3 | 6.820178962 | 1.572251449 | -6.820178962 |
| 283551 | C14orf182 | 6.820178962 | 6.820178962 | -1.01282404 |
| 5327 | PLAT | 6.554588852 | 6.554588852 | -6.554588852 |
| 2212 | FCGR2A | 6.554588852 | 1.306661338 | -6.554588852 |
| 5313 | PKLR | 6.554588852 | 6.554588852 | -6.554588852 |
| 114897 | C1QTNF1 | 6.554588852 | 6.554588852 | -6.554588852 |
| 7781 | SLC30A3 | 6.554588852 | 6.554588852 | -6.554588852 |
| 1815 | DRD4 | 6.554588852 | 6.554588852 | -6.554588852 |
| 2256 | FGF11 | 6.554588852 | 6.554588852 | -6.554588852 |
| 7103 | TSPAN8 | 6.554588852 | 6.554588852 | -6.554588852 |
| 112476 | PRRT2 | 6.554588852 | 1.306661338 | -6.554588852 |
| 25802 | LMOD1 | 6.554588852 | 6.554588852 | -1.345135486 |
| 4137 | MAPT | 6.554588852 | 6.554588852 | -1.345135486 |
| 727897 | MUC5B | 6.554588852 | 6.554588852 | -1.345135486 |
| 80725 | SRCIN1 | 6.554588852 | 6.554588852 | -6.554588852 |
| 4642 | MYO1D | 6.554588852 | 6.554588852 | -6.554588852 |
| 201229 | C17orf108 | 6.554588852 | 6.554588852 | -6.554588852 |
| 5657 | PRTN3 | 6.554588852 | 6.554588852 | -1.345135486 |
| 23508 | TTC9 | 6.554588852 | 1.306661338 | -6.554588852 |
| 55512 | SMPD3 | 6.554588852 | 6.554588852 | -6.554588852 |
| 79689 | STEAP4 | 6.554588852 | 6.554588852 | -6.554588852 |
| 122945 | NOXRED1 | 6.554588852 | 6.554588852 | -1.345135486 |
| 728743 | LOC728743 | 6.554588852 | 1.306661338 | -6.554588852 |
| 57572 | DOCK6 | 6.554588852 | 6.554588852 | -6.554588852 |
| 54518 | APBB1IP | 6.554588852 | 6.554588852 | -6.554588852 |
| 1756 | DMD | 6.554588852 | 6.554588852 | -6.554588852 |
| 386597 | LOC386597 | 6.554588852 | 6.554588852 | -6.554588852 |
| 57047 | PLSCR2 | 6.554588852 | 6.554588852 | -6.554588852 |
| 80115 | BAIAP2L2 | 6.554588852 | 6.554588852 | -6.554588852 |
| 162466 | PHOSPHO1 | 6.554588852 | 1.306661338 | -6.554588852 |
| 113763 | C7orf29 | 6.554588852 | 6.554588852 | -6.554588852 |
| 284677 | C1orf204 | 6.554588852 | 6.554588852 | -6.554588852 |
| 27120 | DKKL1 | 6.554588852 | 6.554588852 | -6.554588852 |
| 146540 | ZNF785 | 6.554588852 | 6.554588852 | -1.345135486 |
| 100128569 | C19orf71 | 6.554588852 | 6.554588852 | -6.554588852 |
| 644596 | LINC00087 | 6.554588852 | 6.554588852 | -6.554588852 |
| 10637 | LEFTY1 | 6.554588852 | 1.306661338 | -6.554588852 |
| 79805 | VASH2 | 6.22881869 | 6.22881869 | -6.22881869 |
| 56971 | CEACAM19 | 6.22881869 | 6.22881869 | -6.22881869 |
| 200844 | C3orf67 | 6.22881869 | 6.22881869 | -6.22881869 |
| 100192386 | FLJ16779 | 6.22881869 | 6.22881869 | -6.22881869 |
| 7869 | SEMA3B | 6.22881869 | 6.22881869 | -1.019365325 |
| 100507266 | LOC100507266 | 6.22881869 | 6.22881869 | -6.22881869 |
| 135932 | TMEM139 | 6.22881869 | 6.22881869 | -6.22881869 |
| 145788 | FLJ27352 | 6.22881869 | 6.22881869 | -6.22881869 |
| 83982 | IFI27L2 | 6.22881869 | 6.22881869 | -6.22881869 |
| 57616 | TSHZ3 | 6.22881869 | 6.22881869 | -6.22881869 |
| 8626 | TP63 | 6.22881869 | 6.22881869 | -6.22881869 |
| 55561 | CDC42BPG | 6.22881869 | 6.22881869 | -6.22881869 |
| 377007 | KLHL30 | 6.22881869 | 6.22881869 | -6.22881869 |
| 9381 | OTOF | 6.22881869 | 6.22881869 | -6.22881869 |
| 84448 | ABLIM2 | 6.22881869 | 6.22881869 | -6.22881869 |
| 50617 | ATP6V0A4 | 6.22881869 | 6.22881869 | -6.22881869 |
| 6861 | SYT5 | 6.22881869 | 6.22881869 | -6.22881869 |
| 2737 | GLI3 | 6.22881869 | 6.22881869 | -6.22881869 |
| 56606 | SLC2A9 | 6.22881869 | 6.22881869 | -6.22881869 |
| 83538 | TTC25 | 6.22881869 | 6.22881869 | -6.22881869 |
| 283152 | CCDC153 | 6.22881869 | 6.22881869 | -6.22881869 |
| 129804 | FBLN7 | 6.22881869 | 6.22881869 | -1.019365325 |
| 9900 | SV2A | 6.22881869 | 6.22881869 | -6.22881869 |
| 282973 | JAKMIP3 | 6.22881869 | 6.22881869 | -6.22881869 |
| 28514 | DLL1 | 6.22881869 | 6.22881869 | -6.22881869 |
| 150384 | CN5H6.4 | 6.22881869 | 6.22881869 | -1.019365325 |
| 51334 | PRR16 | 6.22881869 | 6.22881869 | -6.22881869 |
| 57088 | PLSCR4 | 6.22881869 | 6.22881869 | -6.22881869 |
| 54103 | PION | 6.22881869 | 6.22881869 | -6.22881869 |
| 2661 | GDF9 | 6.22881869 | 6.22881869 | -6.22881869 |
| 3811 | KIR3DL1 | 6.22881869 | 6.22881869 | -6.22881869 |
| 84699 | CREB3L3 | 6.22881869 | 6.22881869 | -6.22881869 |
| 79955 | PDZD7 | 6.22881869 | 6.22881869 | -6.22881869 |
| 56967 | C14orf132 | 6.22881869 | 6.22881869 | -6.22881869 |
| 729522 | AACSP1 | 6.22881869 | 6.22881869 | -6.22881869 |
| 389337 | ARHGEF37 | 6.22881869 | 6.22881869 | -6.22881869 |
| 349667 | RTN4RL2 | 6.22881869 | 6.22881869 | -1.019365325 |
| 284086 | NEK8 | 6.22881869 | 6.22881869 | -1.019365325 |
| 162962 | ZNF836 | 6.22881869 | 6.22881869 | -6.22881869 |
| 130733 | TMEM178 | 6.22881869 | 6.22881869 | -6.22881869 |
| 7078 | TIMP3 | 6.22881869 | 6.22881869 | -6.22881869 |
| 23566 | LPAR3 | 6.22881869 | 6.22881869 | -6.22881869 |
| 221481 | ARMC12 | 6.22881869 | 6.22881869 | -6.22881869 |
| 100133957 | LOC100133957 | 6.22881869 | 6.22881869 | -6.22881869 |
| 203102 | ADAM32 | 6.22881869 | 6.22881869 | -6.22881869 |
| 85301 | COL27A1 | 6.22881869 | 6.22881869 | -6.22881869 |
| 54502 | RBM47 | 6.22881869 | 6.22881869 | -6.22881869 |
| 120071 | GYLTL1B | 6.22881869 | 6.22881869 | -6.22881869 |
| 641455 | POTEM | 6.22881869 | 6.22881869 | -6.22881869 |
| 57480 | PLEKHG1 | 6.22881869 | 6.22881869 | -1.019365325 |
| 54413 | NLGN3 | 6.22881869 | 6.22881869 | -6.22881869 |
| 51147 | ING4 | 5.807354922 | 5.807354922 | -5.807354922 |
| 5922 | RASA2 | 5.807354922 | 5.807354922 | -5.807354922 |
| 3563 | IL3RA | 5.807354922 | 5.807354922 | -5.807354922 |
| 3780 | KCNN1 | 5.807354922 | 5.807354922 | -5.807354922 |
| 3208 | HPCA | 5.807354922 | 5.807354922 | -5.807354922 |
| 4610 | MYCL1 | 5.807354922 | 5.807354922 | -5.807354922 |
| 440299 | DNM1P41 | 5.807354922 | 5.807354922 | -5.807354922 |
| 8736 | MYOM1 | 5.807354922 | 5.807354922 | -5.807354922 |
| 145200 | LINC00239 | 5.807354922 | 5.807354922 | -5.807354922 |
| 100216001 | LOC100216001 | 5.807354922 | 5.807354922 | -5.807354922 |
| 51285 | RASL12 | 5.807354922 | 5.807354922 | -5.807354922 |
| 1400 | CRMP1 | 5.807354922 | 5.807354922 | -5.807354922 |
| 100128124 | HGC6.3 | 5.807354922 | 5.807354922 | -5.807354922 |
| 27019 | DNAI1 | 5.807354922 | 5.807354922 | -5.807354922 |
| 728118 | FAM22A | 5.807354922 | 5.807354922 | -5.807354922 |
| 401136 | TMPRSS11BNL | 5.807354922 | 5.807354922 | -5.807354922 |
| 972 | CD74 | 5.807354922 | 5.807354922 | -5.807354922 |
| 160287 | LDHAL6A | 5.807354922 | 5.807354922 | -5.807354922 |
| 80258 | EFHC2 | 5.807354922 | 5.807354922 | -5.807354922 |
| 285973 | ATG9B | 5.807354922 | 5.807354922 | -5.807354922 |
| 9547 | CXCL14 | 5.807354922 | 5.807354922 | -5.807354922 |
| 6297 | SALL2 | 5.807354922 | 5.807354922 | -5.807354922 |
| 374407 | DNAJB13 | 5.807354922 | 5.807354922 | -5.807354922 |
| 26150 | RIBC2 | 5.807354922 | 5.807354922 | -5.807354922 |
| 6288 | SAA1 | 5.807354922 | 5.807354922 | -5.807354922 |
| 6425 | SFRP5 | 5.807354922 | 5.807354922 | -5.807354922 |
| 55065 | SLC52A1 | 5.807354922 | 5.807354922 | -5.807354922 |
| 255101 | CCDC108 | 5.807354922 | 5.807354922 | -5.807354922 |
| 81931 | ZNF93 | 5.807354922 | 5.807354922 | -5.807354922 |
| 79258 | MMEL1 | 5.807354922 | 5.807354922 | -5.807354922 |
| 83401 | ELOVL3 | 5.807354922 | 5.807354922 | -5.807354922 |
| 5733 | PTGER3 | 5.807354922 | 5.807354922 | -5.807354922 |
| 9172 | MYOM2 | 5.807354922 | 5.807354922 | -5.807354922 |
| 84808 | C1orf170 | 5.807354922 | 5.807354922 | -5.807354922 |
| 84879 | MFSD2A | 5.807354922 | 5.807354922 | -5.807354922 |
| 648791 | PPP1R3G | 5.807354922 | 5.807354922 | -5.807354922 |
| 195814 | SDR16C5 | 5.807354922 | 5.807354922 | -5.807354922 |
| 1991 | ELANE | 5.807354922 | 5.807354922 | -5.807354922 |
| 326342 | EMR4P | 5.807354922 | 5.807354922 | -5.807354922 |
| 432 | ASGR1 | 5.807354922 | 5.807354922 | -5.807354922 |
| 3592 | IL12A | 5.807354922 | 5.807354922 | -5.807354922 |
| 10044 | SH2D3C | 5.807354922 | 5.807354922 | -5.807354922 |
| 441454 | LOC441454 | 5.807354922 | 5.807354922 | -5.807354922 |
| 9914 | ATP2C2 | 5.807354922 | 5.807354922 | -5.807354922 |
| 118663 | BTBD16 | 5.807354922 | 5.807354922 | -5.807354922 |
| 442421 | PTGER4P2 | 5.807354922 | 5.807354922 | -5.807354922 |
| 4311 | MME | 5.807354922 | 5.807354922 | -5.807354922 |
| 2122 | MECOM | 5.807354922 | 5.807354922 | -5.807354922 |
| 79935 | CNTD2 | 5.807354922 | 5.807354922 | -5.807354922 |
| 84750 | FUT10 | 5.807354922 | 5.807354922 | -5.807354922 |
| 81563 | C1orf21 | 5.807354922 | 5.807354922 | -5.807354922 |
| 126969 | SLC44A3 | 5.807354922 | 5.807354922 | -5.807354922 |
| 64097 | EPB41L4A | 5.807354922 | 5.807354922 | -5.807354922 |
| 64386 | MMP25 | 5.807354922 | 5.807354922 | -5.807354922 |
| 642273 | FAM110C | 5.807354922 | 5.807354922 | -5.807354922 |
| 285588 | EFCAB9 | 5.807354922 | 5.807354922 | -5.807354922 |
| 389118 | CDHR4 | 5.807354922 | 5.807354922 | -5.807354922 |
| 27134 | TJP3 | 5.807354922 | 5.807354922 | -5.807354922 |
| 51302 | CYP39A1 | 5.807354922 | 5.807354922 | -5.807354922 |
| 3386 | ICAM4 | 5.807354922 | 5.807354922 | -5.807354922 |
| 100302119 | MIR1538 | 5.807354922 | 5.807354922 | -5.807354922 |
| 440910 | LOC440910 | 5.807354922 | 5.807354922 | -5.807354922 |
| 158067 | AK8 | 5.807354922 | 5.807354922 | -5.807354922 |
| 619505 | SNORA21 | 5.807354922 | 5.807354922 | -5.807354922 |
| 347 | APOD | 5.807354922 | 5.807354922 | -5.807354922 |
| 9514 | GAL3ST1 | 5.807354922 | 5.807354922 | -5.807354922 |
| 56271 | BEX4 | 5.807354922 | 5.807354922 | -5.807354922 |
| 51561 | IL23A | 5.807354922 | 5.807354922 | -5.807354922 |
| 285103 | LOC285103 | 5.807354922 | 5.807354922 | -5.807354922 |
| 127579 | DCST2 | 5.807354922 | 5.807354922 | -5.807354922 |
| 79963 | ABCA11P | 5.807354922 | 5.807354922 | -5.807354922 |
| 400866 | LINC00114 | 5.807354922 | 5.807354922 | -5.807354922 |
| 100506211 | MIR210HG | 5.492707774 | 3.841943214 | -4.035025937 |
| 54541 | DDIT4 | 5.343594833 | 2.353117607 | -4.036933495 |
| 54845 | ESRP1 | 5.247927513 | 5.247927513 | -5.247927513 |
| 100190940 | LOC100190940 | 5.247927513 | 5.247927513 | -5.247927513 |
| 7087 | ICAM5 | 5.247927513 | 5.247927513 | -5.247927513 |
| 677777 | SCARNA12 | 5.247927513 | 5.247927513 | -5.247927513 |
| 100130311 | C17orf107 | 5.247927513 | 5.247927513 | -5.247927513 |
| 26266 | SLC13A4 | 5.247927513 | 5.247927513 | -5.247927513 |
| 5625 | PRODH | 5.247927513 | 5.247927513 | -5.247927513 |
| 100132215 | LOC100132215 | 5.247927513 | 5.247927513 | -5.247927513 |
| 100506939 | LOC100506939 | 5.247927513 | 5.247927513 | -5.247927513 |
| 84249 | PSD2 | 5.247927513 | 5.247927513 | -5.247927513 |
| 1644 | DDC | 5.247927513 | 5.247927513 | -5.247927513 |
| 23281 | MTUS2 | 5.247927513 | 5.247927513 | -5.247927513 |
| 57167 | SALL4 | 5.247927513 | 5.247927513 | -5.247927513 |
| 100616668 | LOC100616668 | 5.247927513 | 5.247927513 | -5.247927513 |
| 129293 | C2orf89 | 5.247927513 | 5.247927513 | -5.247927513 |
| 197335 | WDR90 | 5.247927513 | 5.247927513 | -5.247927513 |
| 6092 | ROBO2 | 5.247927513 | 5.247927513 | -5.247927513 |
| 128853 | DUSP15 | 5.247927513 | 5.247927513 | -5.247927513 |
| 23563 | CHST5 | 5.247927513 | 5.247927513 | -5.247927513 |
| 283796 | GOLGA8IP | 5.247927513 | 5.247927513 | -5.247927513 |
| 51214 | IGF2-AS | 5.247927513 | 5.247927513 | -5.247927513 |
| 4320 | MMP11 | 5.247927513 | 5.247927513 | -5.247927513 |
| 283948 | NHLRC4 | 5.247927513 | 5.247927513 | -5.247927513 |
| 84960 | KIAA1984 | 5.247927513 | 5.247927513 | -5.247927513 |
| 3373 | HYAL1 | 5.247927513 | 5.247927513 | -5.247927513 |
| 1041 | CDSN | 5.247927513 | 5.247927513 | -5.247927513 |
| 5608 | MAP2K6 | 5.247927513 | 5.247927513 | -5.247927513 |
| 339674 | BK250D10.8 | 5.247927513 | 5.247927513 | -5.247927513 |
| 8743 | TNFSF10 | 5.247927513 | 5.247927513 | -5.247927513 |
| 2073 | ERCC5 | 5.247927513 | 5.247927513 | -5.247927513 |
| 152024 | LOC152024 | 5.247927513 | 5.247927513 | -5.247927513 |
| 692215 | SNORD112 | 5.247927513 | 5.247927513 | -5.247927513 |
| 9963 | SLC23A1 | 5.247927513 | 5.247927513 | -5.247927513 |
| 6401 | SELE | 5.247927513 | 5.247927513 | -5.247927513 |
| 100422948 | MIR4284 | 5.247927513 | 5.247927513 | -5.247927513 |
| 7018 | TF | 5.247927513 | 5.247927513 | -5.247927513 |
| 100288123 | LOC100288123 | 5.247927513 | 5.247927513 | -5.247927513 |
| 7368 | UGT8 | 5.247927513 | 5.247927513 | -5.247927513 |
| 100128081 | JAZF1-AS1 | 5.247927513 | 5.247927513 | -5.247927513 |
| 5325 | PLAGL1 | 5.247927513 | 5.247927513 | -5.247927513 |
| 23231 | SEL1L3 | 5.247927513 | 5.247927513 | -5.247927513 |
| 100507050 | LOC100507050 | 5.247927513 | 5.247927513 | -5.247927513 |
| 7638 | ZNF221 | 5.247927513 | 5.247927513 | -5.247927513 |
| 653140 | C2orf84 | 5.247927513 | 5.247927513 | -5.247927513 |
| 5837 | PYGM | 5.247927513 | 5.247927513 | -5.247927513 |
| 3580 | CXCR2P1 | 5.247927513 | 5.247927513 | -5.247927513 |
| 4248 | MGAT3 | 5.247927513 | 5.247927513 | -5.247927513 |
| 29119 | CTNNA3 | 5.247927513 | 5.247927513 | -5.247927513 |
| 2244 | FGB | 5.247927513 | 5.247927513 | -5.247927513 |
| 7047 | TGM4 | 5.247927513 | 5.247927513 | -5.247927513 |
| 158405 | KIAA1958 | 5.247927513 | 5.247927513 | -5.247927513 |
| 6542 | SLC7A2 | 5.247927513 | 5.247927513 | -5.247927513 |
| 121551 | BTBD11 | 5.247927513 | 5.247927513 | -5.247927513 |
| 5143 | PDE4C | 5.247927513 | 5.247927513 | -5.247927513 |
| 80215 | RUNX1-IT1 | 5.247927513 | 5.247927513 | -5.247927513 |
| 29993 | PACSIN1 | 5.247927513 | 5.247927513 | -5.247927513 |
| 10901 | DHRS4 | 5.247927513 | 5.247927513 | -5.247927513 |
| 5738 | PTGFRN | 5.247927513 | 5.247927513 | -5.247927513 |
| 347051 | SLC10A5 | 5.247927513 | 5.247927513 | -5.247927513 |
| 1137 | CHRNA4 | 5.247927513 | 5.247927513 | -5.247927513 |
| 283876 | FLJ39639 | 5.247927513 | 5.247927513 | -5.247927513 |
| 127495 | LRRC39 | 5.247927513 | 5.247927513 | -5.247927513 |
| 56159 | TEX11 | 5.247927513 | 5.247927513 | -5.247927513 |
| 23233 | EXOC6B | 5.247927513 | 5.247927513 | -5.247927513 |
| 2676 | GFRA3 | 5.247927513 | 5.247927513 | -5.247927513 |
| 11320 | MGAT4A | 5.247927513 | 5.247927513 | -5.247927513 |
| 51296 | SLC15A3 | 5.247927513 | 5.247927513 | -5.247927513 |
| 57468 | SLC12A5 | 5.247927513 | 5.247927513 | -5.247927513 |
| 643965 | TMEM88B | 5.247927513 | 5.247927513 | -5.247927513 |
| 9635 | CLCA2 | 5.247927513 | 5.247927513 | -5.247927513 |
| 8542 | APOL1 | 5.247927513 | 5.247927513 | -5.247927513 |
| 285033 | LOC285033 | 5.247927513 | 5.247927513 | -5.247927513 |
| 79827 | CLMP | 5.247927513 | 5.247927513 | -5.247927513 |
| 91050 | CCDC149 | 5.247927513 | 5.247927513 | -5.247927513 |
| 284390 | ZNF763 | 5.247927513 | 5.247927513 | -5.247927513 |
| 220001 | VWCE | 5.247927513 | 5.247927513 | -5.247927513 |
| 10224 | ZNF443 | 5.247927513 | 5.247927513 | -5.247927513 |
| 2560 | GABRB1 | 5.247927513 | 5.247927513 | -5.247927513 |
| 6854 | SYN2 | 5.247927513 | 5.247927513 | -5.247927513 |
| 112817 | HOGA1 | 5.247927513 | 5.247927513 | -5.247927513 |
| 100129827 | MRVI1-AS1 | 5.247927513 | 5.247927513 | -5.247927513 |
| 30816 | ERVW-1 | 5.247927513 | 5.247927513 | -5.247927513 |
| 65268 | WNK2 | 5.247927513 | 5.247927513 | -5.247927513 |
| 629 | CFB | 5.247927513 | 5.247927513 | -5.247927513 |
| 100134869 | UBE2Q2P2 | 5.247927513 | 5.247927513 | -5.247927513 |
| 6000 | RGS7 | 5.247927513 | 5.247927513 | -5.247927513 |
| 8787 | RGS9 | 5.247927513 | 5.247927513 | -5.247927513 |
| 656 | BMP8B | 5.247927513 | 5.247927513 | -5.247927513 |
| 117583 | PARD3B | 5.247927513 | 5.247927513 | -5.247927513 |
| 482 | ATP1B2 | 5.247927513 | 5.247927513 | -5.247927513 |
| 7429 | VIL1 | 5.247927513 | 5.247927513 | -5.247927513 |
| 218 | ALDH3A1 | 5.247927513 | 5.247927513 | -5.247927513 |
| 1238 | CCBP2 | 5.247927513 | 5.247927513 | -5.247927513 |
| 374786 | EFCAB5 | 5.247927513 | 5.247927513 | -5.247927513 |
| 65217 | PCDH15 | 5.247927513 | 5.247927513 | -5.247927513 |
| 100302650 | LOC100302650 | 5.247927513 | 5.247927513 | -5.247927513 |
| 389332 | LOC389332 | 5.247927513 | 5.247927513 | -5.247927513 |
| 79623 | GALNT14 | 5.247927513 | 5.247927513 | -5.247927513 |
| 116443 | GRIN3A | 5.247927513 | 5.247927513 | -5.247927513 |
| 90338 | ZNF160 | 5.247927513 | 5.247927513 | -5.247927513 |
| 7075 | TIE1 | 5.247927513 | 5.247927513 | -5.247927513 |
| 3781 | KCNN2 | 5.247927513 | 5.247927513 | -5.247927513 |
| 90187 | EMILIN3 | 5.247927513 | 5.247927513 | -5.247927513 |
| 643719 | SCGB1B2P | 5.247927513 | 5.247927513 | -5.247927513 |
| 119395 | CALHM3 | 5.247927513 | 5.247927513 | -5.247927513 |
| 401494 | PTPLAD2 | 5.247927513 | 5.247927513 | -5.247927513 |
| 4440 | MSI1 | 5.247927513 | 5.247927513 | -5.247927513 |
| 150197 | LOC150197 | 5.247927513 | 5.247927513 | -5.247927513 |
| 2053 | EPHX2 | 5.247927513 | 5.247927513 | -5.247927513 |
| 123099 | DEGS2 | 5.247927513 | 5.247927513 | -5.247927513 |
| 400830 | DEFB132 | 5.247927513 | 5.247927513 | -5.247927513 |
| 282763 | OR51B5 | 5.247927513 | 5.247927513 | -5.247927513 |
| 401081 | FLJ22763 | 5.247927513 | 5.247927513 | -5.247927513 |
| 116535 | MRGPRF | 5.247927513 | 5.247927513 | -5.247927513 |
| 79132 | DHX58 | 5.247927513 | 5.247927513 | -5.247927513 |
| 79411 | GLB1L | 5.247927513 | 5.247927513 | -5.247927513 |
| 100133144 | UBE2Q2P3 | 5.247927513 | 5.247927513 | -5.247927513 |
| 389813 | C9orf172 | 5.247927513 | 5.247927513 | -5.247927513 |
| 27124 | INPP5J | 5.247927513 | 5.247927513 | -5.247927513 |
| 7454 | WAS | 5.247927513 | 5.247927513 | -5.247927513 |
| 81854 | MGC3771 | 5.247927513 | 5.247927513 | -5.247927513 |
| 84734 | FAM167B | 5.247927513 | 5.247927513 | -5.247927513 |
| 1269 | CNR2 | 5.247927513 | 5.247927513 | -5.247927513 |
| 56477 | CCL28 | 5.247927513 | 5.247927513 | -5.247927513 |
| 3248 | HPGD | 5.247927513 | 5.247927513 | -5.247927513 |
| 692085 | SNORD45C | 5.247927513 | 5.247927513 | -5.247927513 |
| 22798 | LAMB4 | 5.247927513 | 5.247927513 | -5.247927513 |
| 25876 | SPEF1 | 5.247927513 | 5.247927513 | -5.247927513 |
| 4629 | MYH11 | 5.247927513 | 5.247927513 | -5.247927513 |
| 4359 | MPZ | 5.247927513 | 5.247927513 | -5.247927513 |
| 650 | BMP2 | 5.247927513 | 5.247927513 | -5.247927513 |
| 162461 | TMEM92 | 5.247927513 | 5.247927513 | -5.247927513 |
| 117286 | CIB3 | 5.247927513 | 5.247927513 | -5.247927513 |
| 7832 | BTG2 | 5.247927513 | 5.247927513 | -5.247927513 |
| 10788 | IQGAP2 | 5.247927513 | 5.247927513 | -5.247927513 |
| 166336 | PRICKLE2 | 5.247927513 | 5.247927513 | -5.247927513 |
| 92359 | CRB3 | 5.247927513 | 5.247927513 | -5.247927513 |
| 116844 | LRG1 | 5.247927513 | 5.247927513 | -5.247927513 |
| 3902 | LAG3 | 5.247927513 | 5.247927513 | -5.247927513 |
| 164668 | APOBEC3H | 5.247927513 | 5.247927513 | -5.247927513 |
| 23148 | NACAD | 5.247927513 | 5.247927513 | -5.247927513 |
| 79844 | ZDHHC11 | 5.247927513 | 5.247927513 | -5.247927513 |
| 286530 | P2RY8 | 5.247927513 | 5.247927513 | -5.247927513 |
| 64881 | PCDH20 | 5.247927513 | 5.247927513 | -5.247927513 |
| 374877 | C19orf45 | 5.247927513 | 5.247927513 | -5.247927513 |
| 79012 | CAMKV | 5.247927513 | 5.247927513 | -5.247927513 |
| 389812 | LCN15 | 5.247927513 | 5.247927513 | -5.247927513 |
| 162494 | RHBDL3 | 5.247927513 | 5.247927513 | -5.247927513 |
| 80003 | PCNXL2 | 5.247927513 | 5.247927513 | -5.247927513 |
| 56127 | PCDHB9 | 5.247927513 | 5.247927513 | -5.247927513 |
| 23046 | KIF21B | 5.247927513 | 5.247927513 | -5.247927513 |
| 387486 | LINC00320 | 5.247927513 | 5.247927513 | -5.247927513 |
| 153770 | PLAC8L1 | 5.247927513 | 5.247927513 | -5.247927513 |
| 56171 | DNAH7 | 5.247927513 | 5.247927513 | -5.247927513 |
| 9215 | LARGE | 5.247927513 | 5.247927513 | -5.247927513 |
| 130576 | LYPD6B | 5.247927513 | 5.247927513 | -5.247927513 |
| 5016 | OVGP1 | 5.247927513 | 5.247927513 | -5.247927513 |
| 441425 | ANKRD20A3 | 5.247927513 | 5.247927513 | -5.247927513 |
| 136853 | SRCRB4D | 5.247927513 | 5.247927513 | -5.247927513 |
| 10157 | AASS | 5.247927513 | 5.247927513 | -5.247927513 |
| 255057 | C19orf26 | 5.247927513 | 5.247927513 | -5.247927513 |
| 6439 | SFTPB | 5.247927513 | 5.247927513 | -5.247927513 |
| 195977 | ANTXRL | 5.247927513 | 5.247927513 | -5.247927513 |
| 7051 | TGM1 | 5.247927513 | 5.247927513 | -5.247927513 |
| 51299 | NRN1 | 5.247927513 | 5.247927513 | -5.247927513 |
| 10417 | SPON2 | 5.247927513 | 5.247927513 | -5.247927513 |
| 5555 | PRH2 | 5.247927513 | 5.247927513 | -5.247927513 |
| 26797 | SNORD52 | 5.247927513 | 5.247927513 | -5.247927513 |
| 255809 | C19orf38 | 5.247927513 | 5.247927513 | -5.247927513 |
| 387742 | FAM99A | 5.247927513 | 5.247927513 | -5.247927513 |
| 56981 | PRDM11 | 5.247927513 | 5.247927513 | -5.247927513 |
| 84174 | SLA2 | 5.247927513 | 5.247927513 | -5.247927513 |
| 84894 | LINGO1 | 5.247927513 | 5.247927513 | -5.247927513 |
| 343450 | KCNT2 | 5.247927513 | 5.247927513 | -5.247927513 |
| 3560 | IL2RB | 5.247927513 | 5.247927513 | -5.247927513 |
| 389084 | C2orf82 | 5.247927513 | 5.247927513 | -5.247927513 |
| 83650 | SLC35G5 | 5.247927513 | 5.247927513 | -5.247927513 |
| 123591 | C15orf27 | 5.247927513 | 5.247927513 | -5.247927513 |
| 286367 | LOC286367 | 5.247927513 | 5.247927513 | -5.247927513 |
| 10562 | OLFM4 | 5.247927513 | 5.247927513 | -5.247927513 |
| 92346 | C1orf105 | 5.247927513 | 5.247927513 | -5.247927513 |
| 130574 | LYPD6 | 5.247927513 | 5.247927513 | -5.247927513 |
| 8997 | KALRN | 5.247927513 | 5.247927513 | -5.247927513 |
| 400655 | LOC400655 | 5.247927513 | 5.247927513 | -5.247927513 |
| 647323 | LOC647323 | 5.247927513 | 5.247927513 | -5.247927513 |
| 491 | ATP2B2 | 5.247927513 | 5.247927513 | -5.247927513 |
| 100129354 | NRADDP | 5.247927513 | 5.247927513 | -5.247927513 |
| 1588 | CYP19A1 | 5.247927513 | 5.247927513 | -5.247927513 |
| 56920 | SEMA3G | 5.247927513 | 5.247927513 | -5.247927513 |
| 56256 | SERTAD4 | 5.247927513 | 5.247927513 | -5.247927513 |
| 6775 | STAT4 | 5.247927513 | 5.247927513 | -5.247927513 |
| 1821 | DRP2 | 5.247927513 | 5.247927513 | -5.247927513 |
| 284100 | LOC284100 | 5.247927513 | 5.247927513 | -5.247927513 |
| 145820 | LOC145820 | 5.247927513 | 5.247927513 | -5.247927513 |
| 171022 | ABHD11-AS1 | 5.247927513 | 5.247927513 | -5.247927513 |
| 54718 | BTN2A3P | 5.247927513 | 5.247927513 | -5.247927513 |
| 146439 | CCDC64B | 5.247927513 | 5.247927513 | -5.247927513 |
| 11309 | SLCO2B1 | 5.247927513 | 5.247927513 | -5.247927513 |
| 654466 | KGFLP2 | 5.247927513 | 5.247927513 | -5.247927513 |
| 5176 | SERPINF1 | 5.247927513 | 5.247927513 | -5.247927513 |
| 55613 | MTMR8 | 5.247927513 | 5.247927513 | -5.247927513 |
| 2239 | GPC4 | 5.247927513 | 5.247927513 | -5.247927513 |
| 4858 | NOVA2 | 5.247927513 | 5.247927513 | -5.247927513 |
| 123904 | NRN1L | 5.247927513 | 5.247927513 | -5.247927513 |
| 375287 | RBM43 | 5.247927513 | 5.247927513 | -5.247927513 |
| 56000 | NXF3 | 5.247927513 | 5.247927513 | -5.247927513 |
| 388697 | HRNR | 5.247927513 | 5.247927513 | -5.247927513 |
| 2868 | GRK4 | 5.247927513 | 5.247927513 | -5.247927513 |
| 284805 | C20orf203 | 5.247927513 | 5.247927513 | -5.247927513 |
| 145226 | RDH12 | 5.247927513 | 5.247927513 | -5.247927513 |
| 5999 | RGS4 | 5.247927513 | 5.247927513 | -5.247927513 |
| 2529 | FUT7 | 5.247927513 | 5.247927513 | -5.247927513 |
| 440078 | FAM66C | 5.247927513 | 5.247927513 | -5.247927513 |
| 390195 | OR5AN1 | 5.247927513 | 5.247927513 | -5.247927513 |
| 786 | CACNG1 | 5.247927513 | 5.247927513 | -5.247927513 |
| 284186 | TMEM105 | 5.247927513 | 5.247927513 | -5.247927513 |
| 6916 | TBXAS1 | 5.247927513 | 5.247927513 | -5.247927513 |
| 440836 | ODF3B | 5.247927513 | 5.247927513 | -5.247927513 |
| 133491 | C5orf47 | 5.247927513 | 5.247927513 | -5.247927513 |
| 7067 | THRA | 5.030521945 | 1.455082218 | -1.660063956 |
| 80307 | FER1L4 | 4.609531222 | 3.949467266 | -2.057991723 |
| 12 | SERPINA3 | 4.473317145 | 2.544090219 | -5.220551075 |
| 6038 | RNASE4 | 4.195015982 | 1.506305557 | -3.214124805 |
| 9536 | PTGES | 4.15508451 | 1.993621087 | -2.369589022 |
| 5163 | PDK1 | 4.091981858 | 1.691443929 | -2.634300022 |
| 5064 | PALM | 3.862616696 | 3.536846535 | -2.69055595 |
| 115557 | ARHGEF25 | 3.844829627 | 1.854352402 | -2.059334139 |
| 80221 | ACSF2 | 3.844829627 | 3.259867127 | -9.092757141 |
| 388228 | SBK1 | 3.749151133 | 1.411847812 | -2.155626618 |
| 168544 | ZNF467 | 3.740757173 | 8.988684687 | -3.779231321 |
| 123688 | AGPHD1 | 3.740757173 | 2.434095835 | -8.988684687 |
| 230 | ALDOC | 3.712228916 | 4.120868644 | -4.54016817 |
| 29842 | TFCP2L1 | 3.685763142 | 2.704871964 | -3.126335733 |
| 729359 | PLIN4 | 3.685763142 | 3.685763142 | -8.933690655 |
| 125965 | COX6B2 | 3.628589433 | 1.832122827 | -3.069162025 |
| 9762 | ProSAPiP1 | 3.572251449 | 1.775784843 | -2.01282404 |
| 64122 | FN3K | 3.500621586 | 2.500621586 | -2.507019358 |
| 2550 | GABBR1 | 3.497399469 | 9.726218159 | -1.250484728 |
| 376497 | SLC27A1 | 3.462532523 | 3.043232997 | -1.548262397 |
| 1902 | LPAR1 | 3.445559444 | 1.131244533 | -3.484033592 |
| 1731 | 1-Sep | 3.445559444 | 1.873307995 | -2.138898106 |
| 57715 | SEMA4G | 3.426264755 | 3.400729663 | -2.200196675 |
| 2921 | CXCL3 | 3.37778133 | 1.805529881 | -3.416255477 |
| 51148 | CERCAM | 3.337194852 | 1.900261857 | -2.273398201 |
| 5582 | PRKCG | 3.285402219 | 1.683366205 | -1.691877705 |
| 27165 | GLS2 | 3.231852751 | 2.64689025 | -1.250961574 |
| 2036 | EPB41L1 | 3.222785687 | 2.211814569 | -1.709314229 |
| 5210 | PFKFB4 | 3.154735122 | 2.59964315 | -3.21510774 |
| 4303 | FOXO4 | 3.074000581 | 1.083523356 | -2.093109404 |
| 401303 | ZNF815P | 3.074000581 | 8.321928095 | -1.288505093 |
| 80164 | FLJ22184 | 3.069162025 | 2.647698256 | -1.647698256 |
| 58985 | IL22RA1 | 3.069162025 | 1.638112207 | -1.190016419 |
| 219654 | ZCCHC24 | 3.027389998 | 2.020194497 | -1.123066476 |
| 26232 | FBXO2 | 3.019058336 | 1.166379423 | -4.344828497 |
| 197257 | LDHD | 2.985692163 | 1.679030825 | -1.679030825 |
| 284297 | SSC5D | 2.985692163 | 2.400729663 | -1.004800986 |
| 275 | AMT | 2.985692163 | 2.004800986 | -1.004800986 |
| 7108 | TM7SF2 | 2.975798941 | 2.970323811 | -4.785899239 |
| 10675 | CSPG5 | 2.92721058 | 3.252980741 | -1.252980741 |
| 388588 | LOC388588 | 2.921951104 | 1.97987108 | -1.221511386 |
| 100507421 | LOC100507421 | 2.86393845 | 2.048363022 | -2.86393845 |
| 89848 | FCHSD1 | 2.815199939 | 2.028443978 | -2.162122413 |
| 5864 | RAB3A | 2.790991476 | 1.484330138 | -1.005495988 |
| 83546 | RTBDN | 2.790991476 | 2.206028975 | -8.038918989 |
| 322 | APBB1 | 2.754792591 | 1.724365557 | -1.758641491 |
| 25837 | RAB26 | 2.753708348 | 2.474848975 | -2.404459647 |
| 80235 | PIGZ | 2.723860607 | 1.458270496 | -2.245026457 |
| 81544 | GDPD5 | 2.704871964 | 3.685763142 | -1.704871964 |
| 2065 | ERBB3 | 2.688710426 | 1.707819249 | -2.727184573 |
| 84698 | CAPS2 | 2.688710426 | 1.382049087 | -2.727184573 |
| 138311 | FAM69B | 2.609334719 | 1.187101718 | -3.19429722 |
| 26297 | SERGEF | 2.574657492 | 1.114759209 | -3.90172889 |
| 3643 | INSR | 2.572251449 | 1.591360272 | -2.610725597 |
| 283120 | H19 | 2.562706002 | 3.024811755 | -3.033671732 |
| 85315 | PAQR8 | 2.56175511 | 2.071949842 | -1.305772326 |
| 79816 | TLE6 | 2.538168289 | 1.530514717 | -3.285402219 |
| 1999 | ELF3 | 2.503179096 | 1.646658117 | -3.129113378 |
| 119391 | GSTO2 | 2.4870703 | 2.221480189 | -3.23430423 |
| 10014 | HDAC5 | 2.478406399 | 2.755515991 | -1.936088236 |
| 57326 | PBXIP1 | 2.447743504 | 1.522193453 | -1.923810437 |
| 150291 | MORC2-AS1 | 2.445559444 | 7.693486957 | -2.484033592 |
| 5144 | PDE4D | 2.445559444 | 1.860596943 | -1.138898106 |
| 8398 | PLA2G6 | 2.445559444 | 1.860596943 | -1.886132035 |
| 125875 | CLDND2 | 2.445559444 | 2.445559444 | -1.464668267 |
| 125058 | TBC1D16 | 2.426264755 | 2.985692163 | -1.426264755 |
| 114088 | TRIM9 | 2.426264755 | 2.004800986 | -1.679030825 |
| 4355 | MPP2 | 2.426264755 | 2.004800986 | -3.024166311 |
| 1831 | TSC22D3 | 2.413440714 | 2.189225557 | -2.004800986 |
| 1838 | DTNB | 2.406860843 | 1.723044955 | -4.751996329 |
| 83543 | AIF1L | 2.387023123 | 1.090935033 | -2.754479558 |
| 64764 | CREB3L2 | 2.379101803 | 1.524299719 | -1.119909464 |
| 566 | AZU1 | 2.373570882 | 2.625109649 | -1.102543685 |
| 664 | BNIP3 | 2.36761714 | 1.414217553 | -2.196203572 |
| 534 | ATP6V1G2 | 2.33219643 | 2.891623839 | -1.33219643 |
| 489 | ATP2A3 | 2.328914525 | 2.321928095 | -1.263870993 |
| 23149 | FCHO1 | 2.327687364 | 1.619868116 | -1.080772624 |
| 64077 | LHPP | 2.324486822 | 1.131041654 | -3.496547567 |
| 283335 | LOC283335 | 2.306661338 | 2.306661338 | -1.325770161 |
| 116843 | SLC18B1 | 2.306661338 | 1.325770161 | -7.554588852 |
| 79917 | MAGIX | 2.306661338 | 1.325770161 | -7.554588852 |
| 27076 | LYPD3 | 2.306661338 | 7.554588852 | -7.554588852 |
| 41 | ASIC1 | 2.282933963 | 1.954097499 | -1.437075967 |
| 11156 | PTP4A3 | 2.272578179 | 2.86393845 | -1.545862681 |
| 25878 | MXRA5 | 2.265590111 | 1.581774223 | -1.006397771 |
| 25809 | TTLL1 | 2.250961574 | 1.925191412 | -3.270326898 |
| 3486 | IGFBP3 | 2.250961574 | 2.250961574 | -2.672425342 |
| 84189 | SLITRK6 | 2.231564067 | 8.038918989 | -1.231564067 |
| 2264 | FGFR4 | 2.176322773 | 8.405141463 | -8.405141463 |
| 2781 | GNAZ | 2.176322773 | 1.584962501 | -1.176322773 |
| 7754 | ZNF204P | 2.176322773 | 1.850552611 | -2.176322773 |
| 55200 | PLEKHG6 | 2.152951923 | 1.172060746 | -1.172060746 |
| 60489 | APOBEC3G | 2.152951923 | 1.567989422 | -2.191426071 |
| 64411 | ARAP3 | 2.152951923 | 7.400879436 | -1.172060746 |
| 80323 | CCDC68 | 2.152951923 | 1.172060746 | -1.172060746 |
| 51129 | ANGPTL4 | 2.151330971 | 1.17973761 | -1.927153699 |
| 6919 | TCEA2 | 2.143820599 | 1.136563045 | -1.497804063 |
| 83450 | LRRC48 | 2.129283017 | 2.103747925 | -1.707819249 |
| 58191 | CXCL16 | 2.129283017 | 1.116458977 | -2.727184573 |
| 29121 | CLEC2D | 2.113511693 | 1.524299719 | -1.704871964 |
| 114879 | OSBPL5 | 2.113511693 | 2.113511693 | -8.933690655 |
| 5165 | PDK3 | 2.101910781 | 1.996171528 | -1.454329244 |
| 9744 | ACAP1 | 2.101402032 | 2.74350844 | -1.391297778 |
| 3718 | JAK3 | 2.093109404 | 2.501749132 | -2.767339243 |
| 2941 | GSTA4 | 2.093109404 | 2.093109404 | -1.767339243 |
| 11240 | PADI2 | 2.069704549 | 3.390383016 | -1.657639261 |
| 51090 | PLLP | 2.059334139 | 1.399270183 | -1.691877705 |
| 83937 | RASSF4 | 2.049630768 | 2.723860607 | -1.591948931 |
| 9563 | H6PD | 2.025796001 | 2.219149498 | -1.773959802 |
| 154091 | SLC2A12 | 2.022236474 | 1.187101718 | -2.19429722 |
| 10161 | LPAR6 | 2.01282404 | 1.987288948 | -1.591360272 |
| 170961 | ANKRD24 | 2.01282404 | 7.820178962 | -2.610725597 |
| 256949 | KANK3 | 2.01282404 | 7.820178962 | -7.820178962 |
| 148170 | CDC42EP5 | 2.01282404 | 1.987288948 | -7.820178962 |
| 7137 | TNNI3 | 2.00823615 | 1.105021213 | -3.832205786 |
| 8448 | DOC2A | 2.00698643 | 2.455082218 | -2.660063956 |
| 3791 | KDR | 2.004800986 | 8.233619677 | -8.233619677 |
| 221914 | GPC2 | 2.004800986 | 1.057030945 | -1.785159176 |
| 9537 | TP53I11 | 2.003198882 | 1.827060331 | -1.783783215 |
| 64788 | LMF1 | 2.0031977 | 1.845656423 | -1.785691634 |
| 6319 | SCD | 2.001339485 | 2.359059013 | -2.485472996 |
| 727 | C5 | 2 | 1.734409889 | -2.74723393 |
| 158219 | TTC39B | 1.990477226 | 7.238404739 | -2.028951374 |
| 29767 | TMOD2 | 1.990477226 | 7.238404739 | -2.028951374 |
| 388743 | CAPN8 | 1.990477226 | 7.238404739 | -7.238404739 |
| 10148 | EBI3 | 1.990477226 | 1.009586049 | -2.028951374 |
| 5787 | PTPRB | 1.990477226 | 7.238404739 | -1.431049817 |
| 5793 | PTPRG | 1.990477226 | 1.405514725 | -7.238404739 |
| 1382 | CRABP2 | 1.98960408 | 2.99182243 | -1.023068378 |
| 2026 | ENO2 | 1.979506561 | 1.739302864 | -1.068440289 |
| 85026 | C9orf37 | 1.973495365 | 1.030639272 | -1.431177202 |
| 55084 | SOBP | 1.960455932 | 1.207747602 | -1.243069584 |
| 54885 | TBC1D8B | 1.958533383 | 1.625109649 | -1.500851546 |
| 10610 | ST6GALNAC2 | 1.939208316 | 2.063466419 | -2.396890153 |
| 93 | ACVR2B | 1.938044252 | 1.348832279 | -2.203634363 |
| 57153 | SLC44A2 | 1.934629911 | 1.52093383 | -1.636858464 |
| 6196 | RPS6KA2 | 1.925191412 | 1.659601302 | -1.672425342 |
| 11037 | STON1 | 1.925191412 | 2.250961574 | -8.479780264 |
| 5033 | P4HA1 | 1.924247778 | 2.195621484 | -1.676539103 |
| 8991 | SELENBP1 | 1.921048659 | 1.501749132 | -9.321928095 |
| 9249 | DHRS3 | 1.920781553 | 2.298148635 | -2.620608011 |
| 644172 | LOC644172 | 1.910732662 | 8.139551352 | -1.584962501 |
| 22846 | VASH1 | 1.910732662 | 8.139551352 | -2.930097987 |
| 22979 | EFR3B | 1.910732662 | 1.910732662 | -2.33219643 |
| 25850 | ZNF345 | 1.886132035 | 2.445559444 | -2.484033592 |
| 284751 | LOC284751 | 1.886132035 | 2.445559444 | -1.464668267 |
| 414918 | FAM116B | 1.886132035 | 7.693486957 | -1.138898106 |
| 54800 | KLHL24 | 1.869939459 | 2.777961137 | -1.640457613 |
| 23179 | RGL1 | 1.86393845 | 1.272578179 | -2.538168289 |
| 2250 | FGF5 | 1.850552611 | 1.360747344 | -2.176322773 |
| 153768 | PRELID2 | 1.850552611 | 1.850552611 | -1.004262027 |
| 339942 | H1FX-AS1 | 1.850552611 | 1.850552611 | -3.195688098 |
| 9509 | ADAMTS2 | 1.850552611 | 2.176322773 | -1.176322773 |
| 50853 | VILL | 1.843093945 | 2.647698256 | -2.069162025 |
| 408 | ARRB1 | 1.819519276 | 1.371903408 | -2.170369592 |
| 6528 | SLC5A5 | 1.811927652 | 2.063466419 | -2.224829407 |
| 283870 | C16orf79 | 1.810100299 | 2.206028975 | -2.829465624 |
| 11118 | BTN3A2 | 1.799264335 | 2.240203697 | -1.12311902 |
| 7033 | TFF3 | 1.796466606 | 1.796466606 | -1.237039197 |
| 81621 | KAZALD1 | 1.796466606 | 7.044394119 | -7.044394119 |
| 400619 | LINC00511 | 1.796466606 | 1.796466606 | -7.044394119 |
| 222183 | SRRM3 | 1.796466606 | 1.796466606 | -1.834940754 |
| 3696 | ITGB8 | 1.796466606 | 1.796466606 | -7.044394119 |
| 166012 | CHST13 | 1.796466606 | 7.044394119 | -7.044394119 |
| 27112 | FAM155B | 1.796466606 | 7.044394119 | -1.834940754 |
| 154796 | AMOT | 1.759865996 | 1.168505724 | -1.441790227 |
| 123 | PLIN2 | 1.751797333 | 1.009792877 | -1.137015621 |
| 8718 | TNFRSF25 | 1.74723393 | 1.325770161 | -7.554588852 |
| 114899 | C1QTNF3 | 1.74723393 | 2.306661338 | -1.74723393 |
| 445 | ASS1 | 1.74723393 | 7.554588852 | -1.325770161 |
| 92840 | REEP6 | 1.738671916 | 2.095157233 | -2.33219643 |
| 6508 | SLC4A3 | 1.738671916 | 2.095157233 | -1.214738849 |
| 28986 | MAGEH1 | 1.736965594 | 1.013251023 | -1.053750386 |
| 11094 | CACFD1 | 1.734409889 | 1.145197916 | -1.52116585 |
| 84961 | FBXL20 | 1.734409889 | 2.721698838 | -1.325770161 |
| 64221 | ROBO3 | 1.733117939 | 1.098935364 | -2.110232955 |
| 23492 | CBX7 | 1.729258555 | 1.176180115 | -1.263391111 |
| 11067 | C10orf10 | 1.707819249 | 7.936637939 | -1.707819249 |
| 51450 | PRRX2 | 1.70761477 | 2.64232331 | -2.149619317 |
| 5523 | PPP2R3A | 1.694971506 | 1.387304104 | -1.078814383 |
| 8226 | HDHD1 | 1.679030825 | 1.189225557 | -8.233619677 |
| 57333 | RCN3 | 1.679030825 | 1.679030825 | -2.426264755 |
| 1952 | CELSR2 | 1.676568315 | 1.968749066 | -1.871964003 |
| 6553 | SLC9A5 | 1.659601302 | 2.250961574 | -1.672425342 |
| 641649 | TMEM91 | 1.659601302 | 1.070389328 | -2.250961574 |
| 1513 | CTSK | 1.659601302 | 1.070389328 | -1.446357262 |
| 949 | SCARB1 | 1.649948304 | 2.430263974 | -2.854258367 |
| 978 | CDA | 1.640779715 | 2.812840461 | -2.00823615 |
| 65018 | PINK1 | 1.624881511 | 1.021723478 | -1.875594465 |
| 493 | ATP2B4 | 1.621076903 | 1.421591153 | -1.263391111 |
| 1759 | DNM1 | 1.615254574 | 2.193292551 | -2.981643532 |
| 79589 | RNF128 | 1.609334719 | 1.278457667 | -1.495337948 |
| 100527964 | LOC100527964 | 1.593524514 | 1.172060746 | -7.400879436 |
| 54756 | IL17RD | 1.593524514 | 7.400879436 | -7.400879436 |
| 7161 | TP73 | 1.593524514 | 1.567989422 | -1.172060746 |
| 100272217 | LOC100272217 | 1.592285842 | 2.071119991 | -2.818353921 |
| 57593 | EBF4 | 1.592285842 | 8.625708843 | -2.396890153 |
| 388564 | TMEM238 | 1.592285842 | 1.063466419 | -1.592285842 |
| 85458 | DIXDC1 | 1.591948931 | 2.040044719 | -4.068996093 |
| 30008 | EFEMP2 | 1.591948931 | 4.030521945 | -1.144023138 |
| 27128 | CYTH4 | 1.591791839 | 1.68182404 | -1.801405656 |
| 89849 | ATG16L2 | 1.591360272 | 1.126692005 | -1.786755961 |
| 79777 | ACBD4 | 1.591360272 | 7.820178962 | -7.820178962 |
| 80231 | CXorf21 | 1.591360272 | 2.572251449 | -2.01282404 |
| 7903 | ST8SIA4 | 1.591360272 | 1.591360272 | -2.610725597 |
| 338707 | B4GALNT4 | 1.588243592 | 2.008546407 | -4.070520428 |
| 21 | ABCA3 | 1.58780525 | 1.876679661 | -1.177042406 |
| 23331 | TTC28 | 1.586789854 | 1.141893066 | -1.939208316 |
| 8029 | CUBN | 1.584962501 | 2.584962501 | -1.106128351 |
| 147700 | KLC3 | 1.572251449 | 1.572251449 | -6.820178962 |
| 51364 | ZMYND10 | 1.572251449 | 1.572251449 | -6.820178962 |
| 113451 | ADC | 1.572251449 | 6.820178962 | -1.01282404 |
| 374500 | THSD1P1 | 1.572251449 | 1.572251449 | -6.820178962 |
| 219731 | LOC219731 | 1.572251449 | 6.820178962 | -6.820178962 |
| 27294 | DHDH | 1.572251449 | 6.820178962 | -6.820178962 |
| 10850 | CCL27 | 1.572251449 | 6.820178962 | -6.820178962 |
| 149840 | C20orf196 | 1.572251449 | 6.820178962 | -1.610725597 |
| 257407 | C2orf72 | 1.572251449 | 6.820178962 | -6.820178962 |
| 53358 | SHC3 | 1.572251449 | 6.820178962 | -6.820178962 |
| 115704 | EVI5L | 1.566210717 | 1.772392911 | -2.331396185 |
| 157638 | FAM84B | 1.559083869 | 1.54949782 | -2.754479558 |
| 6376 | CX3CL1 | 1.548162583 | 1.035514287 | -2.133125083 |
| 388650 | FAM69A | 1.543621705 | 1.536652151 | -1.54711915 |
| 79957 | PAQR6 | 1.538168289 | 9.092757141 | -3.883303775 |
| 1241 | LTB4R | 1.508146904 | 1.759685671 | -2.288505093 |
| 93129 | ORAI3 | 1.504507569 | 1.666982266 | -3.676568315 |
| 56895 | AGPAT4 | 1.501321186 | 1.851067225 | -3.448853766 |
| 84988 | PPP1R16A | 1.500851546 | 1.142957954 | -1.052925753 |
| 6857 | SYT1 | 1.496277347 | 1.46533364 | -1.078310517 |
| 2281 | FKBP1B | 1.493303253 | 1.152543483 | -2.828455043 |
| 205 | AK4 | 1.485197937 | 1.777198872 | -2.580391582 |
| 83986 | ITFG3 | 1.470243865 | 1.010628311 | -2.4722687 |
| 6352 | CCL5 | 1.464668267 | 2.464668267 | -1.00698643 |
| 115330 | GPR146 | 1.464668267 | 7.693486957 | -2.484033592 |
| 129807 | NEU4 | 1.464668267 | 1.464668267 | -1.138898106 |
| 9203 | ZMYM3 | 1.448906168 | 1.704553777 | -1.920584164 |
| 9567 | GTPBP1 | 1.447743504 | 1.0252557 | -2.61980425 |
| 126567 | C2CD4C | 1.446357262 | 1.241375525 | -1.446357262 |
| 494513 | DFNB59 | 1.434095835 | 1.168505724 | -1.441790227 |
| 84445 | LZTS2 | 1.431206436 | 9.361943774 | -1.049060818 |
| 84818 | IL17RC | 1.431177202 | 1.176180115 | -3.85264097 |
| 11174 | ADAMTS6 | 1.431049817 | 1.009586049 | -7.238404739 |
| 149428 | BNIPL | 1.431049817 | 1.990477226 | -2.028951374 |
| 29931 | LINC00312 | 1.431049817 | 1.990477226 | -2.028951374 |
| 100287171 | WASH1 | 1.431049817 | 1.405514725 | -1.431049817 |
| 158801 | NKAPP1 | 1.431049817 | 7.238404739 | -7.238404739 |
| 9028 | RHBDL1 | 1.431049817 | 1.405514725 | -2.028951374 |
| 26468 | LHX6 | 1.431049817 | 1.009586049 | -7.238404739 |
| 85442 | KNDC1 | 1.431049817 | 7.238404739 | -1.009586049 |
| 83959 | SLC4A11 | 1.421588834 | 1.705789698 | -2.782016429 |
| 776 | CACNA1D | 1.419299526 | 1.581774223 | -2.591360272 |
| 79366 | HMGN5 | 1.419299526 | 1.257936538 | -1.133678435 |
| 201625 | DNAH12 | 1.419299526 | 2.265590111 | -2.265590111 |
| 8425 | LTBP4 | 1.417730603 | 2.174236383 | -3.577983869 |
| 151174 | LOC151174 | 1.417702741 | 1.057030945 | -2.004800986 |
| 24142 | NAT6 | 1.385209933 | 1.475242133 | -3.712281331 |
| 152 | ADRA2C | 1.382049087 | 1.707819249 | -7.936637939 |
| 160335 | TMTC2 | 1.382049087 | 2.688710426 | -7.936637939 |
| 619383 | SCARNA9 | 1.382049087 | 1.707819249 | -7.936637939 |
| 64772 | ENGASE | 1.376074344 | 1.288345494 | -2.292607521 |
| 8659 | ALDH4A1 | 1.375039431 | 1.226488081 | -1.377693885 |
| 134429 | STARD4 | 1.371718462 | 2.176322773 | -1.004262027 |
| 440253 | WHAMMP2 | 1.371718462 | 1.584962501 | -8.405141463 |
| 78991 | PCYOX1L | 1.35166118 | 1.483238438 | -2.355158624 |
| 26470 | SEZ6L2 | 1.34149854 | 1.739986574 | -2.652000619 |
| 51385 | ZNF589 | 1.325770161 | 2.325770161 | -1.153709415 |
| 400954 | EML6 | 1.325770161 | 1.510194732 | -1.325770161 |
| 404550 | C16orf74 | 1.325338723 | 2.906039197 | -1.413335204 |
| 359821 | MRPL42P5 | 1.306661338 | 6.554588852 | -1.345135486 |
| 154761 | LOC154761 | 1.306661338 | 6.554588852 | -6.554588852 |
| 84837 | ARHGAP5-AS1 | 1.306661338 | 6.554588852 | -6.554588852 |
| 3910 | LAMA4 | 1.306661338 | 1.306661338 | -1.345135486 |
| 9597 | SMAD5-AS1 | 1.306661338 | 6.554588852 | -6.554588852 |
| 374618 | TEX9 | 1.306661338 | 6.554588852 | -6.554588852 |
| 645974 | PABPC1L2B | 1.306661338 | 6.554588852 | -1.345135486 |
| 340348 | TSPAN33 | 1.306661338 | 6.554588852 | -1.345135486 |
| 1117 | CHI3L2 | 1.306661338 | 6.554588852 | -1.345135486 |
| 197320 | ZNF778 | 1.306661338 | 6.554588852 | -1.345135486 |
| 9495 | AKAP5 | 1.306661338 | 6.554588852 | -6.554588852 |
| 379013 | RNF138P1 | 1.306661338 | 1.306661338 | -6.554588852 |
| 728577 | CNTNAP3B | 1.302882339 | 1.189225557 | -1.099193357 |
| 4660 | PPP1R12B | 1.302882339 | 3.004800986 | -3.004800986 |
| 883 | CCBL1 | 1.30256277 | 2.296164999 | -1.64061053 |
| 389792 | IER5L | 1.30256277 | 1.632528184 | -2.429843434 |
| 11322 | TMC6 | 1.292607521 | 1.131244533 | -1.146592498 |
| 65010 | SLC26A6 | 1.289302947 | 1.945439063 | -2.890032764 |
| 3306 | HSPA2 | 1.288505093 | 2.093109404 | -1.288505093 |
| 221935 | SDK1 | 1.279934006 | 1.331088094 | -1.177248342 |
| 80853 | JHDM1D | 1.265590111 | 7.820178962 | -2.610725597 |
| 63891 | RNF123 | 1.257233254 | 1.278077508 | -2.501470065 |
| 26471 | NUPR1 | 1.252307999 | 2.245910228 | -1.083095616 |
| 136288 | C7orf57 | 1.250961574 | 1.659601302 | -1.446357262 |
| 90139 | TSPAN18 | 1.250961574 | 2.250961574 | -1.078900828 |
| 3708 | ITPR1 | 1.243692684 | 1.375269942 | -2.119909464 |
| 116966 | WDR17 | 1.239530469 | 1.869058522 | -1.144023138 |
| 112703 | FAM71E1 | 1.237039197 | 7.044394119 | -1.834940754 |
| 7348 | UPK1B | 1.237039197 | 7.044394119 | -1.834940754 |
| 284348 | LYPD5 | 1.237039197 | 7.044394119 | -7.044394119 |
| 1384 | CRAT | 1.22787796 | 2.221480189 | -1.00823615 |
| 2745 | GLRX | 1.226537749 | 1.500553712 | -2.196092879 |
| 81622 | UNC93B1 | 1.226342007 | 1.589351598 | -1.785391697 |
| 64132 | XYLT2 | 1.226263698 | 1.03324179 | -2.899820122 |
| 10000 | AKT3 | 1.221914708 | 1.037022131 | -1.840705137 |
| 79924 | ADM2 | 1.218740027 | 8.038918989 | -2.829465624 |
| 80757 | TMEM121 | 1.218740027 | 8.038918989 | -2.829465624 |
| 285512 | FAM13A-AS1 | 1.218740027 | 1.484330138 | -2.231564067 |
| 6513 | SLC2A1 | 1.214500601 | 2.192474294 | -1.959414948 |
| 55337 | C19orf66 | 1.214124805 | 2.03355256 | -1.756442969 |
| 3727 | JUND | 1.209199821 | 2.3102103 | -1.265633272 |
| 55022 | PID1 | 1.208814015 | 1.31937239 | -2.910732662 |
| 6405 | SEMA3F | 1.201633861 | 1.078777113 | -1.539681621 |
| 91461 | PKDCC | 1.190016419 | 1.638112207 | -2.647698256 |
| 64598 | MOSPD3 | 1.190016419 | 1.056337984 | -1.329622487 |
| 611 | OPN1SW | 1.176322773 | 2.176322773 | -8.405141463 |
| 7915 | ALDH5A1 | 1.176322773 | 1.584962501 | -1.371718462 |
| 4091 | SMAD6 | 1.174903496 | 1.750279947 | -3.779231321 |
| 644890 | MEIG1 | 1.172060746 | 1.172060746 | -7.400879436 |
| 7005 | TEAD3 | 1.172060746 | 1.172060746 | -1.172060746 |
| 5212 | VIT | 1.172060746 | 7.400879436 | -2.191426071 |
| 64711 | HS3ST6 | 1.169147897 | 1.347546473 | -1.263034406 |
| 51626 | DYNC2LI1 | 1.158624969 | 1.522193453 | -1.097078881 |
| 3726 | JUNB | 1.158337027 | 1.772445874 | -1.675660811 |
| 91 | ACVR1B | 1.154987834 | 1.096435651 | -1.270465051 |
| 54344 | DPM3 | 1.149320311 | 1.79375929 | -1.338307522 |
| 7001 | PRDX2 | 1.146719377 | 1.610623004 | -3.172584698 |
| 23043 | TNIK | 1.138898106 | 1.464668267 | -7.693486957 |
| 192683 | SCAMP5 | 1.138898106 | 7.693486957 | -7.693486957 |
| 2217 | FCGRT | 1.135351853 | 1.304537958 | -2.252307999 |
| 20 | ABCA2 | 1.134504503 | 2.347165386 | -3.9492014 |
| 2549 | GAB1 | 1.116458977 | 1.382049087 | -2.129283017 |
| 100 | ADA | 1.116458977 | 7.936637939 | -7.936637939 |
| 388963 | C2orf81 | 1.116458977 | 2.688710426 | -7.936637939 |
| 23373 | CRTC1 | 1.110921814 | 2.105021213 | -1.907232831 |
| 100127888 | LOC100127888 | 1.106128351 | 1.31937239 | -1.33219643 |
| 84557 | MAP1LC3A | 1.104656158 | 1.996171528 | -2.91201108 |
| 30815 | ST6GALNAC6 | 1.10416634 | 1.096861539 | -1.039717632 |
| 23363 | OBSL1 | 1.100965146 | 1.093831905 | -1.946818375 |
| 51308 | REEP2 | 1.093109404 | 1.277533976 | -1.767339243 |
| 3783 | KCNN4 | 1.084677715 | 2.078777113 | -1.328914525 |
| 254065 | BRWD3 | 1.079254643 | 1.355901215 | -1.858956119 |
| 79671 | NLRX1 | 1.074962058 | 1.996352223 | -5.416255477 |
| 256472 | TMEM151A | 1.071435138 | 1.996519431 | -2.075101455 |
| 126014 | OSCAR | 1.06336635 | 1.056793696 | -3.389692909 |
| 100133091 | LOC100133091 | 1.062735755 | 2.056337984 | -2.321928095 |
| 79746 | ECHDC3 | 1.060246542 | 1.889296536 | -1.247190128 |
| 54813 | KLHL28 | 1.057947349 | 2.168505724 | -2.181329765 |
| 83862 | TMEM120A | 1.044199804 | 1.298285253 | -1.263034406 |
| 3433 | IFIT2 | 1.043902822 | 2.281231514 | -1.486213251 |
| 64856 | VWA1 | 1.038462518 | 1.807182804 | -1.418052897 |
| 254102 | EHBP1L1 | 1.031006057 | 2.089981367 | -3.043795118 |
| 10810 | WASF3 | 1.029573115 | 1.078777113 | -1.539681621 |
| 5155 | PDGFB | 1.023829236 | 1.303403318 | -2.108491633 |
| 57835 | SLC4A5 | 1.022900402 | 1.381354373 | -1.540224186 |
| 115 | ADCY9 | 1.019085841 | 1.262000586 | -2.574657492 |
| 4133 | MAP2 | 1.018689073 | 1.31749461 | -1.327590488 |
| 147686 | ZNF418 | 1.01282404 | 6.820178962 | -6.820178962 |
| 4909 | NTF4 | 1.01282404 | 6.820178962 | -6.820178962 |
| 100272228 | LOC100272228 | 1.01282404 | 1.572251449 | -6.820178962 |
| 64150 | DIO3OS | 1.01282404 | 6.820178962 | -6.820178962 |
| 100131187 | TSTD1 | 1.01282404 | 6.820178962 | -1.01282404 |
| 6515 | SLC2A3 | 1.01282404 | 6.820178962 | -6.820178962 |
| 10957 | PNRC1 | 1.012203123 | 1.425269919 | -1.219044128 |
| 5152 | PDE9A | 1.009586049 | 1.405514725 | -1.009586049 |
| 3623 | INHA | 1.009586049 | 1.990477226 | -2.028951374 |
| 117166 | WFIKKN1 | 1.009586049 | 1.009586049 | -1.431049817 |
| 100130275 | LOC100130275 | 1.009586049 | 1.405514725 | -7.238404739 |
| 26048 | ZNF500 | 1.009586049 | 1.009586049 | -1.431049817 |
| 54753 | ZNF853 | 1.009586049 | 1.405514725 | -1.431049817 |
| 100499227 | LOC100499227 | 1.009586049 | 1.405514725 | -1.431049817 |
| 25946 | ZNF385A | 1.005495988 | 2.206028975 | -8.038918989 |
| 57462 | KIAA1161 | 1.005495988 | 1.810100299 | -2.231564067 |
| 60412 | EXOC4 | 1.004800986 | 1.242097831 | -3.426264755 |
| 26160 | IFT172 | 1.004800986 | 2.004800986 | -3.024166311 |
| 55893 | ZNF395 | 1.003838659 | 1.511473151 | -1.593935311 |
| 8784 | TNFRSF18 | 1.002132587 | 2.848423172 | -1.589230832 |
| 29108 | PYCARD | -8.813781191 | -10.25974326 | 10.60177079 |
| 11249 | NXPH2 | -7.930737338 | -7.820178962 | 5.807354922 |
| 1415 | CRYBB2 | -7.813781191 | -6.22881869 | 6.22881869 |
| 7562 | ZNF708 | -7.813781191 | -7.409390936 | 6.22881869 |
| 348180 | CTU2 | -7.686500527 | -7.044394119 | 7.813781191 |
| 2707 | GJB3 | -7.686500527 | -7.238404739 | 6.22881869 |
| 55027 | HEATR3 | -7.686500527 | -6.22881869 | 5.209453366 |
| 154 | ADRB2 | -7.554588852 | -5.247927513 | 5.807354922 |
| 79631 | EFTUD1 | -7.400879436 | -6.820178962 | 7.033423002 |
| 399687 | MYO18A | -7.033423002 | -7.562242424 | 6.554588852 |
| 201562 | PTPLB | -7.033423002 | -6.820178962 | 6.554588852 |
| 374383 | NCR3LG1 | -7.033423002 | -5.832890014 | 7.033423002 |
| 283518 | KCNRG | -7.033423002 | -7.044394119 | 5.209453366 |
| 26272 | FBXO4 | -7.033423002 | -5.247927513 | 6.22881869 |
| 636 | BICD1 | -6.820178962 | -6.554588852 | 5.209453366 |
| 440138 | ALG11 | -6.820178962 | -6.22881869 | 5.807354922 |
| 85236 | HIST1H2BK | -6.820178962 | -6.554588852 | 5.209453366 |
| 51729 | WBP11 | -6.820178962 | -7.820178962 | 7.22881869 |
| 165545 | DQX1 | -6.554588852 | -6.22881869 | 6.807354922 |
| 142913 | CFL1P1 | -6.554588852 | -5.247927513 | 5.209453366 |
| 1024 | CDK8 | -6.554588852 | -6.554588852 | 7.033423002 |
| 26153 | KIF26A | -6.554588852 | -7.044394119 | 6.807354922 |
| 10389 | SCML2 | -6.554588852 | -5.247927513 | 5.209453366 |
| 9609 | RAB36 | -6.554588852 | -5.247927513 | 5.807354922 |
| 4901 | NRL | -6.554588852 | -6.22881869 | 5.209453366 |
| 6637 | SNRPG | -6.22881869 | -7.936637939 | 5.807354922 |
| 1846 | DUSP4 | -6.22881869 | -6.22881869 | 6.554588852 |
| 55612 | FERMT1 | -6.22881869 | -5.247927513 | 5.807354922 |
| 27350 | APOBEC3C | -6.22881869 | -5.247927513 | 6.22881869 |
| 728912 | NBPF24 | -6.22881869 | -5.247927513 | 5.209453366 |
| 256933 | NPB | -6.22881869 | -6.22881869 | 5.807354922 |
| 57623 | ZFAT | -6.22881869 | -6.22881869 | 6.807354922 |
| 56413 | LTB4R2 | -6.22881869 | -6.22881869 | 7.22881869 |
| 1517 | CTSL1P2 | -5.807354922 | -7.238404739 | 8.033423002 |
| 56126 | PCDHB10 | -5.807354922 | -5.247927513 | 5.209453366 |
| 7768 | ZNF225 | -5.807354922 | -6.22881869 | 5.209453366 |
| 728622 | SKP1P2 | -5.807354922 | -6.22881869 | 5.209453366 |
| 677846 | SNORA80 | -5.807354922 | -5.247927513 | 6.22881869 |
| 9047 | SH2D2A | -5.807354922 | -5.247927513 | 7.033423002 |
| 645121 | CCNI2 | -5.807354922 | -5.832890014 | 5.209453366 |
| 2637 | GBX2 | -5.807354922 | -5.247927513 | 7.033423002 |
| 1193 | CLIC2 | -5.807354922 | -5.247927513 | 5.209453366 |
| 90809 | TMEM55B | -5.807354922 | -7.044394119 | 5.209453366 |
| 100527978 | TMEM56-RWDD3 | -5.807354922 | -7.562242424 | 6.807354922 |
| 2628 | GATM | -5.807354922 | -5.247927513 | 5.209453366 |
| 7136 | TNNI2 | -5.807354922 | -5.832890014 | 5.807354922 |
| 5729 | PTGDR | -5.807354922 | -7.044394119 | 6.22881869 |
| 84517 | ACTRT3 | -5.247927513 | -8.144658243 | 5.807354922 |
| 10201 | NME6 | -5.247927513 | -5.247927513 | 5.209453366 |
| 90233 | ZNF551 | -5.247927513 | -5.247927513 | 5.209453366 |
| 349152 | DPY19L2P2 | -5.247927513 | -6.554588852 | 7.22881869 |
| 26005 | C2CD3 | -5.247927513 | -5.247927513 | 5.807354922 |
| 140686 | WFDC3 | -5.247927513 | -6.22881869 | 6.807354922 |
| 387646 | LRRC37A6P | -5.247927513 | -5.832890014 | 7.033423002 |
| 2246 | FGF1 | -5.247927513 | -5.247927513 | 5.209453366 |
| 154664 | ABCA13 | -5.247927513 | -7.820178962 | 5.209453366 |
| 84657 | GHRLOS2 | -5.247927513 | -6.22881869 | 8.13442632 |
| 112609 | MRAP2 | -5.247927513 | -6.22881869 | 6.807354922 |
| 120939 | C12orf59 | -5.247927513 | -5.247927513 | 6.554588852 |
| 7004 | TEAD4 | -5.247927513 | -5.247927513 | 6.554588852 |
| 347273 | MURC | -5.247927513 | -6.820178962 | 6.22881869 |
| 100271831 | - | -5.247927513 | -5.832890014 | 5.807354922 |
| 728927 | ZNF736 | -5.247927513 | -5.832890014 | 6.22881869 |
| 64091 | POPDC2 | -5.247927513 | -6.22881869 | 5.209453366 |
| 84733 | CBX2 | -5.247927513 | -5.247927513 | 6.22881869 |
| 5314 | PKHD1 | -5.247927513 | -5.247927513 | 7.400879436 |
| 3887 | KRT81 | -5.247927513 | -6.820178962 | 5.807354922 |
| 83856 | FSD1L | -5.247927513 | -7.238404739 | 5.807354922 |
| 100288801 | FRG2C | -5.247927513 | -5.247927513 | 7.033423002 |
| 8360 | HIST1H4D | -5.247927513 | -5.247927513 | 5.209453366 |
| 9711 | KIAA0226 | -3.0694851 | -2.161463423 | 2.785495488 |
| 27063 | ANKRD1 | -2.94760731 | -2.198120447 | 2.236733444 |
| 92255 | LMBRD2 | -2.886498807 | -2.161463423 | 1.306661338 |
| 6650 | SOLH | -2.663970375 | -1.906253504 | 2.796394223 |
| 9329 | GTF3C4 | -2.584962501 | -1.707819249 | 1.172060746 |
| 53354 | PANK1 | -2.481584761 | -2.142378675 | 2.196955207 |
| 7771 | ZFP112 | -2.327071398 | -1.602036014 | 2.117457582 |
| 8863 | PER3 | -2.321928095 | -2.176322773 | 1.172060746 |
| 729920 | ISPD | -2.306661338 | -1.990477226 | 1.559427409 |
| 22809 | ATF5 | -2.279697888 | -1.343471582 | 1.865480335 |
| 26145 | IRF2BP1 | -2.231564067 | -2.337303321 | 2.505528033 |
| 55765 | C1orf106 | -2.152951923 | -1.796466606 | 2.152951923 |
| 100303728 | SLC25A5-AS1 | -2.152951923 | -3.513623719 | 3.298966946 |
| 79862 | ZNF669 | -2.123382416 | -1.237039197 | 2.006426269 |
| 221178 | SPATA13 | -2.123382416 | -2.822001698 | 1.421463768 |
| 2318 | FLNC | -2.123382416 | -1.237039197 | 1.226068079 |
| 158747 | MOSPD2 | -1.980891177 | -1.572251449 | 1.559427409 |
| 306 | ANXA3 | -1.879145605 | -1.237039197 | 1.739539538 |
| 54826 | GIN1 | -1.846290585 | -1.382049087 | 1.992305608 |
| 65083 | NOL6 | -1.829115441 | -1.682860144 | 1.935660359 |
| 4312 | MMP1 | -1.810100299 | -3.562344198 | 2.855989697 |
| 83541 | FAM110A | -1.701918647 | -1.333423734 | 1.172060746 |
| 790955 | C11orf83 | -1.701918647 | -1.591360272 | 2 |
| 643837 | LOC643837 | -1.695520876 | -1.504001584 | 1.359730128 |
| 11097 | NUPL2 | -1.692332598 | -2.042366031 | 1.074478216 |
| 4147 | MATN2 | -1.674229839 | -1.489805268 | 1.992305608 |
| 133584 | EGFLAM | -1.617505874 | -1.842194971 | 2.118427946 |
| 100132707 | LOC100132707 | -1.572251449 | -1.572251449 | 1.785495488 |
| 285352 | FLJ39534 | -1.572251449 | -1.306661338 | 1.980891177 |
| 27153 | ZNF777 | -1.547920799 | -1.368348775 | 1.140990101 |
| 121053 | C12orf45 | -1.53287399 | -1.003831862 | 1.131911676 |
| 10495 | ENOX2 | -1.516873511 | -1.524006752 | 1.17559821 |
| 205717 | KIAA2018 | -1.511827796 | -1.368348775 | 1.232544039 |
| 64897 | C12orf43 | -1.497233651 | -2.224215157 | 1.866321565 |
| 54619 | CCNJ | -1.484330138 | -1.590069391 | 1.674229839 |
| 7057 | THBS1 | -1.482958102 | -1.236574037 | 1.626563405 |
| 7516 | XRCC2 | -1.457681837 | -1.591360272 | 1.172060746 |
| 284098 | PIGW | -1.457681837 | -1.464668267 | 1.172060746 |
| 9125 | RQCD1 | -1.457681837 | -2.650764559 | 1.584962501 |
| 1102 | RCBTB2 | -1.447288436 | -1.741562608 | 1.814644958 |
| 27304 | MOCS3 | -1.376148486 | -1.382049087 | 1.131911676 |
| 57082 | CASC5 | -1.359824703 | -1.105739254 | 1.710950438 |
| 9507 | ADAMTS4 | -1.356485317 | -1.652573407 | 2.431339312 |
| 2730 | GCLM | -1.333313102 | -1.028979067 | 1.350907162 |
| 221079 | ARL5B | -1.318855261 | -1.382049087 | 1.47883415 |
| 467 | ATF3 | -1.306661338 | -1.572251449 | 2.785495488 |
| 375248 | ANKRD36 | -1.306661338 | -2.161463423 | 1.559427409 |
| 378708 | APITD1 | -1.305028494 | -4.169722076 | 1.507433369 |
| 92935 | MARS2 | -1.282080162 | -1.132702537 | 1.694158285 |
| 631 | BFSP1 | -1.25919234 | -1.007653573 | 1.131911676 |
| 81282 | OR51G2 | -1.172060746 | -1.591360272 | 1.318075769 |
| 9404 | LPXN | -1.172060746 | -1.333423734 | 1.318075769 |
| 150290 | DUSP18 | -1.162474697 | -1.584962501 | 1.379980763 |
| 83448 | PUS7L | -1.142271555 | -1.18377784 | 1.239608557 |
| 167555 | FAM151B | -1.110558375 | -1.663636815 | 1.931365097 |
| 25939 | SAMHD1 | -1.098072123 | -1.720969194 | 2.020425472 |
| 4998 | ORC1 | -1.085097591 | -1.55378562 | 1.225779949 |
| 4701 | NDUFA7 | -1.01282404 | -1.431049817 | 2.81103058 |

**Table S3. List of 1033 DEGs for V^△^ *vs* Y.**

| **Gene ID** | **Symbol** | **log_2_Ratio (Y vs BC)** | **log_2_Ratio (Y vs NC)** | | | **log_2_Ratio (V^△^ vs Y)** |
| --- | --- | --- | --- | --- | --- | --- |
| 7089 | TLE2 | 9.361943774 | | 2.317549654 | -3.554588852 | |
| 90993 | CREB3L1 | 9.361943774 | | 4.11401626 | -3.133125083 | |
| 94015 | TTYH2 | 9.361943774 | | 1.668456816 | -2.317549654 | |
| 147138 | TMC8 | 9.139551352 | | 2.31937239 | -1.910732662 | |
| 4035 | LRP1 | 9.139551352 | | 3.306661338 | -3.33219643 | |
| 1944 | EFNA3 | 8.933690655 | | 1.524299719 | -2.113511693 | |
| 10023 | FRAT1 | 8.820178962 | | 2.265590111 | -2 | |
| 7464 | CORO2A | 8.820178962 | | 2.591360272 | -1.419299526 | |
| 79783 | C7orf10 | 8.758223215 | | 1.519818475 | -1.064736257 | |
| 11247 | NXPH4 | 8.758223215 | | 8.758223215 | -2.203634363 | |
| 11045 | UPK1A | 8.554588852 | | 2 | -8.554588852 | |
| 10382 | TUBB4A | 8.479780264 | | 8.479780264 | -2.250961574 | |
| 80022 | MYO15B | 8.479780264 | | 8.479780264 | -8.479780264 | |
| 165679 | SPTSSB | 8.405141463 | | 2.176322773 | -1.584962501 | |
| 93233 | CCDC114 | 8.405141463 | | 2.572251449 | -2.597786541 | |
| 64798 | DEPTOR | 8.405141463 | | 8.405141463 | -1.584962501 | |
| 113612 | CYP2U1 | 8.405141463 | | 2.176322773 | -8.405141463 | |
| 83715 | ESPN | 8.321928095 | | 2.489038081 | -8.321928095 | |
| 50649 | ARHGEF4 | 8.321928095 | | 1.501749132 | -2.514573173 | |
| 25759 | SHC2 | 8.321928095 | | 8.321928095 | -8.321928095 | |
| 4055 | LTBR | 8.321928095 | | 2.489038081 | -3.074000581 | |
| 90293 | KLHL13 | 8.233619677 | | 1.679030825 | -1.004800986 | |
| 84152 | PPP1R1B | 8.233619677 | | 8.233619677 | -2.985692163 | |
| 6820 | SULT2B1 | 8.233619677 | | 2.985692163 | -2.004800986 | |
| 4854 | NOTCH3 | 8.038918989 | | 8.038918989 | -8.038918989 | |
| 54933 | RHBDL2 | 8.038918989 | | 2.790991476 | -8.038918989 | |
| 64284 | RAB17 | 7.936637939 | | 2.688710426 | -1.707819249 | |
| 84812 | PLCD4 | 7.936637939 | | 1.707819249 | -7.936637939 | |
| 285908 | LINC00174 | 7.820178962 | | 1.265590111 | -7.820178962 | |
| 51760 | SYT17 | 7.820178962 | | 7.820178962 | -2.572251449 | |
| 57168 | ASPHD2 | 7.820178962 | | 2.572251449 | -2.572251449 | |
| 4868 | NPHS1 | 7.820178962 | | 1.265590111 | -7.820178962 | |
| 5630 | PRPH | 7.693486957 | | 7.693486957 | -7.693486957 | |
| 7592 | ZNF41 | 7.693486957 | | 1.464668267 | -2.445559444 | |
| 1066 | CES1 | 7.693486957 | | 1.138898106 | -7.693486957 | |
| 151473 | SLC16A14 | 7.693486957 | | 1.464668267 | -2.445559444 | |
| 9625 | AATK | 7.693486957 | | 7.693486957 | -1.464668267 | |
| 56521 | DNAJC12 | 7.693486957 | | 1.138898106 | -7.693486957 | |
| 23416 | KCNH3 | 7.554588852 | | 7.554588852 | -7.554588852 | |
| 400242 | DICER1-AS1 | 7.554588852 | | 2.306661338 | -1.74723393 | |
| 342184 | FMN1 | 7.554588852 | | 7.554588852 | -7.554588852 | |
| 222962 | SLC29A4 | 7.554588852 | | 7.554588852 | -7.554588852 | |
| 6364 | CCL20 | 7.554588852 | | 1.721698838 | -7.554588852 | |
| 643641 | ZNF862 | 7.554588852 | | 7.554588852 | -7.554588852 | |
| 9746 | CLSTN3 | 7.554588852 | | 1.721698838 | -7.554588852 | |
| 653160 | LOC653160 | 7.554588852 | | 2.306661338 | -2.306661338 | |
| 10384 | BTN3A3 | 7.554588852 | | 2.306661338 | -7.554588852 | |
| 5662 | PSD | 7.554588852 | | 7.554588852 | -2.306661338 | |
| 8497 | PPFIA4 | 7.554588852 | | 7.554588852 | -7.554588852 | |
| 199731 | CADM4 | 7.554588852 | | 2.306661338 | -1.74723393 | |
| 284069 | FAM171A2 | 7.400879436 | | 7.400879436 | -7.400879436 | |
| 79148 | MMP28 | 7.400879436 | | 7.400879436 | -7.400879436 | |
| 139728 | PNCK | 7.400879436 | | 7.400879436 | -7.400879436 | |
| 3429 | IFI27 | 7.400879436 | | 1.172060746 | -2.152951923 | |
| 3201 | HOXA4 | 7.400879436 | | 2.152951923 | -2.152951923 | |
| 139105 | BEND2 | 7.400879436 | | 1.172060746 | -2.152951923 | |
| 10761 | PLAC1 | 7.400879436 | | 1.567989422 | -2.152951923 | |
| 51279 | C1RL | 7.400879436 | | 1.567989422 | -1.172060746 | |
| 79603 | CERS4 | 7.400879436 | | 1.172060746 | -2.152951923 | |
| 100129518 | LOC100129518 | 7.400879436 | | 1.567989422 | -7.400879436 | |
| 4760 | NEUROD1 | 7.400879436 | | 1.172060746 | -7.400879436 | |
| 90019 | SYT8 | 7.400879436 | | 7.400879436 | -2.152951923 | |
| 27151 | CPAMD8 | 7.400879436 | | 7.400879436 | -7.400879436 | |
| 199699 | DAND5 | 7.400879436 | | 1.172060746 | -1.593524514 | |
| 10235 | RASGRP2 | 7.238404739 | | 7.238404739 | -1.431049817 | |
| 5333 | PLCD1 | 7.238404739 | | 1.009586049 | -7.238404739 | |
| 9473 | C1orf38 | 7.238404739 | | 1.009586049 | -1.990477226 | |
| 1298 | COL9A2 | 7.238404739 | | 7.238404739 | -7.238404739 | |
| 3934 | LCN2 | 7.238404739 | | 7.238404739 | -7.238404739 | |
| 5740 | PTGIS | 7.238404739 | | 7.238404739 | -1.431049817 | |
| 221421 | RSPH9 | 7.238404739 | | 1.009586049 | -1.009586049 | |
| 10316 | NMUR1 | 7.238404739 | | 7.238404739 | -7.238404739 | |
| 146771 | TCAM1P | 7.238404739 | | 1.990477226 | -7.238404739 | |
| 100506046 | LOC100506046 | 7.238404739 | | 7.238404739 | -1.431049817 | |
| 259217 | HSPA12A | 7.238404739 | | 7.238404739 | -7.238404739 | |
| 83999 | KREMEN1 | 7.238404739 | | 7.238404739 | -7.238404739 | |
| 388152 | LOC388152 | 7.238404739 | | 7.238404739 | -1.990477226 | |
| 4902 | NRTN | 7.044394119 | | 7.044394119 | -7.044394119 | |
| 79611 | ACSS3 | 7.044394119 | | 7.044394119 | -7.044394119 | |
| 344148 | NCKAP5 | 7.044394119 | | 7.044394119 | -1.237039197 | |
| 105 | ADARB2 | 7.044394119 | | 7.044394119 | -7.044394119 | |
| 3108 | HLA-DMA | 7.044394119 | | 7.044394119 | -7.044394119 | |
| 284161 | GDPD1 | 7.044394119 | | 7.044394119 | -7.044394119 | |
| 58473 | PLEKHB1 | 7.044394119 | | 1.211504105 | -7.044394119 | |
| 54760 | PCSK4 | 7.044394119 | | 1.796466606 | -7.044394119 | |
| 4059 | BCAM | 7.044394119 | | 7.044394119 | -7.044394119 | |
| 390664 | C1QTNF8 | 7.044394119 | | 1.211504105 | -7.044394119 | |
| 634 | CEACAM1 | 7.044394119 | | 1.796466606 | -7.044394119 | |
| 10076 | PTPRU | 7.044394119 | | 1.796466606 | -7.044394119 | |
| 643180 | CCT6P3 | 7.044394119 | | 1.796466606 | -1.796466606 | |
| 50619 | DEF6 | 7.044394119 | | 7.044394119 | -7.044394119 | |
| 126129 | CPT1C | 7.044394119 | | 7.044394119 | -7.044394119 | |
| 100422970 | MIR1273D | 6.820178962 | | 6.820178962 | -6.820178962 | |
| 10966 | RAB40B | 6.820178962 | | 1.572251449 | -6.820178962 | |
| 768 | CA9 | 6.820178962 | | 6.820178962 | -1.572251449 | |
| 54510 | PCDH18 | 6.820178962 | | 6.820178962 | -1.01282404 | |
| 55876 | GSDMB | 6.820178962 | | 6.820178962 | -6.820178962 | |
| 29935 | RPA4 | 6.820178962 | | 6.820178962 | -6.820178962 | |
| 60468 | BACH2 | 6.820178962 | | 6.820178962 | -6.820178962 | |
| 114818 | KLHL29 | 6.820178962 | | 1.572251449 | -1.572251449 | |
| 221806 | VWDE | 6.820178962 | | 1.572251449 | -6.820178962 | |
| 7379 | UPK2 | 6.820178962 | | 6.820178962 | -6.820178962 | |
| 440356 | LOC440356 | 6.820178962 | | 6.820178962 | -6.820178962 | |
| 3039 | HBA1 | 6.820178962 | | 1.572251449 | -1.572251449 | |
| 79750 | ZNF385D | 6.820178962 | | 6.820178962 | -6.820178962 | |
| 100132832 | LOC100132832 | 6.820178962 | | 1.572251449 | -1.01282404 | |
| 89846 | FGD3 | 6.820178962 | | 1.572251449 | -6.820178962 | |
| 283551 | C14orf182 | 6.820178962 | | 6.820178962 | -6.820178962 | |
| 9363 | RAB33A | 6.554588852 | | 6.554588852 | -6.554588852 | |
| 5327 | PLAT | 6.554588852 | | 6.554588852 | -1.306661338 | |
| 5313 | PKLR | 6.554588852 | | 6.554588852 | -6.554588852 | |
| 7781 | SLC30A3 | 6.554588852 | | 6.554588852 | -6.554588852 | |
| 1815 | DRD4 | 6.554588852 | | 6.554588852 | -6.554588852 | |
| 7103 | TSPAN8 | 6.554588852 | | 6.554588852 | -6.554588852 | |
| 112476 | PRRT2 | 6.554588852 | | 1.306661338 | -6.554588852 | |
| 25802 | LMOD1 | 6.554588852 | | 6.554588852 | -6.554588852 | |
| 4137 | MAPT | 6.554588852 | | 6.554588852 | -6.554588852 | |
| 80725 | SRCIN1 | 6.554588852 | | 6.554588852 | -6.554588852 | |
| 4642 | MYO1D | 6.554588852 | | 6.554588852 | -6.554588852 | |
| 201229 | C17orf108 | 6.554588852 | | 6.554588852 | -6.554588852 | |
| 5657 | PRTN3 | 6.554588852 | | 6.554588852 | -6.554588852 | |
| 23508 | TTC9 | 6.554588852 | | 1.306661338 | -6.554588852 | |
| 55512 | SMPD3 | 6.554588852 | | 6.554588852 | -6.554588852 | |
| 79689 | STEAP4 | 6.554588852 | | 6.554588852 | -1.306661338 | |
| 122945 | NOXRED1 | 6.554588852 | | 6.554588852 | -6.554588852 | |
| 728743 | LOC728743 | 6.554588852 | | 1.306661338 | -6.554588852 | |
| 401106 | FLJ34208 | 6.554588852 | | 6.554588852 | -6.554588852 | |
| 57572 | DOCK6 | 6.554588852 | | 6.554588852 | -6.554588852 | |
| 54518 | APBB1IP | 6.554588852 | | 6.554588852 | -6.554588852 | |
| 1756 | DMD | 6.554588852 | | 6.554588852 | -6.554588852 | |
| 386597 | LOC386597 | 6.554588852 | | 6.554588852 | -1.306661338 | |
| 2957 | GTF2A1 | 6.554588852 | | 6.554588852 | -6.554588852 | |
| 80115 | BAIAP2L2 | 6.554588852 | | 6.554588852 | -6.554588852 | |
| 79369 | B3GNT4 | 6.554588852 | | 6.554588852 | -6.554588852 | |
| 113763 | C7orf29 | 6.554588852 | | 6.554588852 | -6.554588852 | |
| 284677 | C1orf204 | 6.554588852 | | 6.554588852 | -6.554588852 | |
| 7773 | ZNF230 | 6.554588852 | | 6.554588852 | -6.554588852 | |
| 27120 | DKKL1 | 6.554588852 | | 6.554588852 | -6.554588852 | |
| 146540 | ZNF785 | 6.554588852 | | 6.554588852 | -1.306661338 | |
| 100128569 | C19orf71 | 6.554588852 | | 6.554588852 | -6.554588852 | |
| 79883 | PODNL1 | 6.554588852 | | 6.554588852 | -1.306661338 | |
| 644596 | LINC00087 | 6.554588852 | | 6.554588852 | -1.306661338 | |
| 10637 | LEFTY1 | 6.554588852 | | 1.306661338 | -6.554588852 | |
| 56971 | CEACAM19 | 6.22881869 | | 6.22881869 | -6.22881869 | |
| 200844 | C3orf67 | 6.22881869 | | 6.22881869 | -6.22881869 | |
| 100192386 | FLJ16779 | 6.22881869 | | 6.22881869 | -6.22881869 | |
| 283314 | MATL2963 | 6.22881869 | | 6.22881869 | -6.22881869 | |
| 100507266 | LOC100507266 | 6.22881869 | | 6.22881869 | -6.22881869 | |
| 145788 | FLJ27352 | 6.22881869 | | 6.22881869 | -6.22881869 | |
| 83982 | IFI27L2 | 6.22881869 | | 6.22881869 | -6.22881869 | |
| 8626 | TP63 | 6.22881869 | | 6.22881869 | -6.22881869 | |
| 55561 | CDC42BPG | 6.22881869 | | 6.22881869 | -6.22881869 | |
| 377007 | KLHL30 | 6.22881869 | | 6.22881869 | -6.22881869 | |
| 9381 | OTOF | 6.22881869 | | 6.22881869 | -6.22881869 | |
| 50617 | ATP6V0A4 | 6.22881869 | | 6.22881869 | -6.22881869 | |
| 6861 | SYT5 | 6.22881869 | | 6.22881869 | -6.22881869 | |
| 2737 | GLI3 | 6.22881869 | | 6.22881869 | -6.22881869 | |
| 56606 | SLC2A9 | 6.22881869 | | 6.22881869 | -6.22881869 | |
| 83538 | TTC25 | 6.22881869 | | 6.22881869 | -6.22881869 | |
| 9900 | SV2A | 6.22881869 | | 6.22881869 | -6.22881869 | |
| 282973 | JAKMIP3 | 6.22881869 | | 6.22881869 | -6.22881869 | |
| 28514 | DLL1 | 6.22881869 | | 6.22881869 | -6.22881869 | |
| 51334 | PRR16 | 6.22881869 | | 6.22881869 | -6.22881869 | |
| 57088 | PLSCR4 | 6.22881869 | | 6.22881869 | -6.22881869 | |
| 54103 | PION | 6.22881869 | | 6.22881869 | -6.22881869 | |
| 2661 | GDF9 | 6.22881869 | | 6.22881869 | -6.22881869 | |
| 3811 | KIR3DL1 | 6.22881869 | | 6.22881869 | -6.22881869 | |
| 84699 | CREB3L3 | 6.22881869 | | 6.22881869 | -6.22881869 | |
| 79955 | PDZD7 | 6.22881869 | | 6.22881869 | -6.22881869 | |
| 56967 | C14orf132 | 6.22881869 | | 6.22881869 | -6.22881869 | |
| 729522 | AACSP1 | 6.22881869 | | 6.22881869 | -6.22881869 | |
| 389337 | ARHGEF37 | 6.22881869 | | 6.22881869 | -6.22881869 | |
| 162962 | ZNF836 | 6.22881869 | | 6.22881869 | -6.22881869 | |
| 3140 | MR1 | 6.22881869 | | 6.22881869 | -6.22881869 | |
| 130733 | TMEM178 | 6.22881869 | | 6.22881869 | -6.22881869 | |
| 7078 | TIMP3 | 6.22881869 | | 6.22881869 | -6.22881869 | |
| 23566 | LPAR3 | 6.22881869 | | 6.22881869 | -6.22881869 | |
| 221481 | ARMC12 | 6.22881869 | | 6.22881869 | -6.22881869 | |
| 100133957 | LOC100133957 | 6.22881869 | | 6.22881869 | -6.22881869 | |
| 203102 | ADAM32 | 6.22881869 | | 6.22881869 | -6.22881869 | |
| 85301 | COL27A1 | 6.22881869 | | 6.22881869 | -6.22881869 | |
| 120071 | GYLTL1B | 6.22881869 | | 6.22881869 | -6.22881869 | |
| 4211 | MEIS1 | 6.22881869 | | 6.22881869 | -6.22881869 | |
| 641455 | POTEM | 6.22881869 | | 6.22881869 | -6.22881869 | |
| 54413 | NLGN3 | 6.22881869 | | 6.22881869 | -6.22881869 | |
| 51147 | ING4 | 5.807354922 | | 5.807354922 | -5.807354922 | |
| 3780 | KCNN1 | 5.807354922 | | 5.807354922 | -5.807354922 | |
| 3208 | HPCA | 5.807354922 | | 5.807354922 | -5.807354922 | |
| 4610 | MYCL1 | 5.807354922 | | 5.807354922 | -5.807354922 | |
| 440299 | DNM1P41 | 5.807354922 | | 5.807354922 | -5.807354922 | |
| 145200 | LINC00239 | 5.807354922 | | 5.807354922 | -5.807354922 | |
| 7503 | XIST | 5.807354922 | | 5.807354922 | -5.807354922 | |
| 100128124 | HGC6.3 | 5.807354922 | | 5.807354922 | -5.807354922 | |
| 27019 | DNAI1 | 5.807354922 | | 5.807354922 | -5.807354922 | |
| 440896 | LOC440896 | 5.807354922 | | 5.807354922 | -5.807354922 | |
| 128344 | C1orf88 | 5.807354922 | | 5.807354922 | -5.807354922 | |
| 972 | CD74 | 5.807354922 | | 5.807354922 | -5.807354922 | |
| 160287 | LDHAL6A | 5.807354922 | | 5.807354922 | -5.807354922 | |
| 80258 | EFHC2 | 5.807354922 | | 5.807354922 | -5.807354922 | |
| 285973 | ATG9B | 5.807354922 | | 5.807354922 | -5.807354922 | |
| 3777 | KCNK3 | 5.807354922 | | 5.807354922 | -5.807354922 | |
| 9547 | CXCL14 | 5.807354922 | | 5.807354922 | -5.807354922 | |
| 6297 | SALL2 | 5.807354922 | | 5.807354922 | -5.807354922 | |
| 374407 | DNAJB13 | 5.807354922 | | 5.807354922 | -5.807354922 | |
| 80975 | TMPRSS5 | 5.807354922 | | 5.807354922 | -5.807354922 | |
| 54734 | RAB39A | 5.807354922 | | 5.807354922 | -5.807354922 | |
| 6288 | SAA1 | 5.807354922 | | 5.807354922 | -5.807354922 | |
| 5880 | RAC2 | 5.807354922 | | 5.807354922 | -5.807354922 | |
| 6425 | SFRP5 | 5.807354922 | | 5.807354922 | -5.807354922 | |
| 55065 | SLC52A1 | 5.807354922 | | 5.807354922 | -5.807354922 | |
| 255101 | CCDC108 | 5.807354922 | | 5.807354922 | -5.807354922 | |
| 79258 | MMEL1 | 5.807354922 | | 5.807354922 | -5.807354922 | |
| 149465 | WDR65 | 5.807354922 | | 5.807354922 | -5.807354922 | |
| 83401 | ELOVL3 | 5.807354922 | | 5.807354922 | -5.807354922 | |
| 5733 | PTGER3 | 5.807354922 | | 5.807354922 | -5.807354922 | |
| 79690 | GAL3ST4 | 5.807354922 | | 5.807354922 | -5.807354922 | |
| 4914 | NTRK1 | 5.807354922 | | 5.807354922 | -5.807354922 | |
| 387597 | ILDR2 | 5.807354922 | | 5.807354922 | -5.807354922 | |
| 440585 | FAM183A | 5.807354922 | | 5.807354922 | -5.807354922 | |
| 84808 | C1orf170 | 5.807354922 | | 5.807354922 | -5.807354922 | |
| 84879 | MFSD2A | 5.807354922 | | 5.807354922 | -5.807354922 | |
| 648791 | PPP1R3G | 5.807354922 | | 5.807354922 | -5.807354922 | |
| 195814 | SDR16C5 | 5.807354922 | | 5.807354922 | -5.807354922 | |
| 1991 | ELANE | 5.807354922 | | 5.807354922 | -5.807354922 | |
| 326342 | EMR4P | 5.807354922 | | 5.807354922 | -5.807354922 | |
| 3592 | IL12A | 5.807354922 | | 5.807354922 | -5.807354922 | |
| 10044 | SH2D3C | 5.807354922 | | 5.807354922 | -5.807354922 | |
| 441454 | LOC441454 | 5.807354922 | | 5.807354922 | -5.807354922 | |
| 9914 | ATP2C2 | 5.807354922 | | 5.807354922 | -5.807354922 | |
| 118663 | BTBD16 | 5.807354922 | | 5.807354922 | -5.807354922 | |
| 644242 | LOC644242 | 5.807354922 | | 5.807354922 | -5.807354922 | |
| 8608 | RDH16 | 5.807354922 | | 5.807354922 | -5.807354922 | |
| 442421 | PTGER4P2 | 5.807354922 | | 5.807354922 | -5.807354922 | |
| 4311 | MME | 5.807354922 | | 5.807354922 | -5.807354922 | |
| 79935 | CNTD2 | 5.807354922 | | 5.807354922 | -5.807354922 | |
| 84750 | FUT10 | 5.807354922 | | 5.807354922 | -5.807354922 | |
| 81563 | C1orf21 | 5.807354922 | | 5.807354922 | -5.807354922 | |
| 161247 | FITM1 | 5.807354922 | | 5.807354922 | -5.807354922 | |
| 126969 | SLC44A3 | 5.807354922 | | 5.807354922 | -5.807354922 | |
| 64386 | MMP25 | 5.807354922 | | 5.807354922 | -5.807354922 | |
| 29974 | A1CF | 5.807354922 | | 5.807354922 | -5.807354922 | |
| 642273 | FAM110C | 5.807354922 | | 5.807354922 | -5.807354922 | |
| 285588 | EFCAB9 | 5.807354922 | | 5.807354922 | -5.807354922 | |
| 389118 | CDHR4 | 5.807354922 | | 5.807354922 | -5.807354922 | |
| 27134 | TJP3 | 5.807354922 | | 5.807354922 | -5.807354922 | |
| 51302 | CYP39A1 | 5.807354922 | | 5.807354922 | -5.807354922 | |
| 3386 | ICAM4 | 5.807354922 | | 5.807354922 | -5.807354922 | |
| 100302119 | MIR1538 | 5.807354922 | | 5.807354922 | -5.807354922 | |
| 9892 | SNAP91 | 5.807354922 | | 5.807354922 | -5.807354922 | |
| 158067 | AK8 | 5.807354922 | | 5.807354922 | -5.807354922 | |
| 146 | ADRA1D | 5.807354922 | | 5.807354922 | -5.807354922 | |
| 353497 | POLN | 5.807354922 | | 5.807354922 | -5.807354922 | |
| 347 | APOD | 5.807354922 | | 5.807354922 | -5.807354922 | |
| 9514 | GAL3ST1 | 5.807354922 | | 5.807354922 | -5.807354922 | |
| 56271 | BEX4 | 5.807354922 | | 5.807354922 | -5.807354922 | |
| 4807 | NHLH1 | 5.807354922 | | 5.807354922 | -5.807354922 | |
| 285103 | LOC285103 | 5.807354922 | | 5.807354922 | -5.807354922 | |
| 843 | CASP10 | 5.807354922 | | 5.807354922 | -5.807354922 | |
| 79963 | ABCA11P | 5.807354922 | | 5.807354922 | -5.807354922 | |
| 400866 | LINC00114 | 5.807354922 | | 5.807354922 | -5.807354922 | |
| 100506211 | MIR210HG | 5.492707774 | | 3.841943214 | -3.904542841 | |
| 54541 | DDIT4 | 5.343594833 | | 2.353117607 | -2.898035389 | |
| 54845 | ESRP1 | 5.247927513 | | 5.247927513 | -5.247927513 | |
| 100190940 | LOC100190940 | 5.247927513 | | 5.247927513 | -5.247927513 | |
| 7087 | ICAM5 | 5.247927513 | | 5.247927513 | -5.247927513 | |
| 677777 | SCARNA12 | 5.247927513 | | 5.247927513 | -5.247927513 | |
| 100130311 | C17orf107 | 5.247927513 | | 5.247927513 | -5.247927513 | |
| 26266 | SLC13A4 | 5.247927513 | | 5.247927513 | -5.247927513 | |
| 5625 | PRODH | 5.247927513 | | 5.247927513 | -5.247927513 | |
| 100132215 | LOC100132215 | 5.247927513 | | 5.247927513 | -5.247927513 | |
| 84249 | PSD2 | 5.247927513 | | 5.247927513 | -5.247927513 | |
| 1644 | DDC | 5.247927513 | | 5.247927513 | -5.247927513 | |
| 23281 | MTUS2 | 5.247927513 | | 5.247927513 | -5.247927513 | |
| 57167 | SALL4 | 5.247927513 | | 5.247927513 | -5.247927513 | |
| 3823 | KLRC3 | 5.247927513 | | 5.247927513 | -5.247927513 | |
| 100616668 | LOC100616668 | 5.247927513 | | 5.247927513 | -5.247927513 | |
| 129293 | C2orf89 | 5.247927513 | | 5.247927513 | -5.247927513 | |
| 6092 | ROBO2 | 5.247927513 | | 5.247927513 | -5.247927513 | |
| 128853 | DUSP15 | 5.247927513 | | 5.247927513 | -5.247927513 | |
| 151835 | CPNE9 | 5.247927513 | | 5.247927513 | -5.247927513 | |
| 283796 | GOLGA8IP | 5.247927513 | | 5.247927513 | -5.247927513 | |
| 51214 | IGF2-AS | 5.247927513 | | 5.247927513 | -5.247927513 | |
| 4320 | MMP11 | 5.247927513 | | 5.247927513 | -5.247927513 | |
| 283948 | NHLRC4 | 5.247927513 | | 5.247927513 | -5.247927513 | |
| 3373 | HYAL1 | 5.247927513 | | 5.247927513 | -5.247927513 | |
| 1041 | CDSN | 5.247927513 | | 5.247927513 | -5.247927513 | |
| 5608 | MAP2K6 | 5.247927513 | | 5.247927513 | -5.247927513 | |
| 339674 | BK250D10.8 | 5.247927513 | | 5.247927513 | -5.247927513 | |
| 8743 | TNFSF10 | 5.247927513 | | 5.247927513 | -5.247927513 | |
| 152024 | LOC152024 | 5.247927513 | | 5.247927513 | -5.247927513 | |
| 692215 | SNORD112 | 5.247927513 | | 5.247927513 | -5.247927513 | |
| 100233209 | LOC100233209 | 5.247927513 | | 5.247927513 | -5.247927513 | |
| 9963 | SLC23A1 | 5.247927513 | | 5.247927513 | -5.247927513 | |
| 6401 | SELE | 5.247927513 | | 5.247927513 | -5.247927513 | |
| 25862 | USP49 | 5.247927513 | | 5.247927513 | -5.247927513 | |
| 147670 | LOC147670 | 5.247927513 | | 5.247927513 | -5.247927513 | |
| 100422948 | MIR4284 | 5.247927513 | | 5.247927513 | -5.247927513 | |
| 7018 | TF | 5.247927513 | | 5.247927513 | -5.247927513 | |
| 100288123 | LOC100288123 | 5.247927513 | | 5.247927513 | -5.247927513 | |
| 27443 | CECR2 | 5.247927513 | | 5.247927513 | -5.247927513 | |
| 4803 | NGF | 5.247927513 | | 5.247927513 | -5.247927513 | |
| 7368 | UGT8 | 5.247927513 | | 5.247927513 | -5.247927513 | |
| 100128081 | JAZF1-AS1 | 5.247927513 | | 5.247927513 | -5.247927513 | |
| 5325 | PLAGL1 | 5.247927513 | | 5.247927513 | -5.247927513 | |
| 23231 | SEL1L3 | 5.247927513 | | 5.247927513 | -5.247927513 | |
| 7638 | ZNF221 | 5.247927513 | | 5.247927513 | -5.247927513 | |
| 653140 | C2orf84 | 5.247927513 | | 5.247927513 | -5.247927513 | |
| 339416 | ANKRD45 | 5.247927513 | | 5.247927513 | -5.247927513 | |
| 3580 | CXCR2P1 | 5.247927513 | | 5.247927513 | -5.247927513 | |
| 4248 | MGAT3 | 5.247927513 | | 5.247927513 | -5.247927513 | |
| 29119 | CTNNA3 | 5.247927513 | | 5.247927513 | -5.247927513 | |
| 2244 | FGB | 5.247927513 | | 5.247927513 | -5.247927513 | |
| 7047 | TGM4 | 5.247927513 | | 5.247927513 | -5.247927513 | |
| 388815 | LINC00478 | 5.247927513 | | 5.247927513 | -5.247927513 | |
| 158405 | KIAA1958 | 5.247927513 | | 5.247927513 | -5.247927513 | |
| 6542 | SLC7A2 | 5.247927513 | | 5.247927513 | -5.247927513 | |
| 121551 | BTBD11 | 5.247927513 | | 5.247927513 | -5.247927513 | |
| 80215 | RUNX1-IT1 | 5.247927513 | | 5.247927513 | -5.247927513 | |
| 29993 | PACSIN1 | 5.247927513 | | 5.247927513 | -5.247927513 | |
| 10901 | DHRS4 | 5.247927513 | | 5.247927513 | -5.247927513 | |
| 6791 | AURKAPS1 | 5.247927513 | | 5.247927513 | -5.247927513 | |
| 5738 | PTGFRN | 5.247927513 | | 5.247927513 | -5.247927513 | |
| 347051 | SLC10A5 | 5.247927513 | | 5.247927513 | -5.247927513 | |
| 1137 | CHRNA4 | 5.247927513 | | 5.247927513 | -5.247927513 | |
| 127495 | LRRC39 | 5.247927513 | | 5.247927513 | -5.247927513 | |
| 56159 | TEX11 | 5.247927513 | | 5.247927513 | -5.247927513 | |
| 23233 | EXOC6B | 5.247927513 | | 5.247927513 | -5.247927513 | |
| 2676 | GFRA3 | 5.247927513 | | 5.247927513 | -5.247927513 | |
| 11320 | MGAT4A | 5.247927513 | | 5.247927513 | -5.247927513 | |
| 7093 | TLL2 | 5.247927513 | | 5.247927513 | -5.247927513 | |
| 51296 | SLC15A3 | 5.247927513 | | 5.247927513 | -5.247927513 | |
| 57468 | SLC12A5 | 5.247927513 | | 5.247927513 | -5.247927513 | |
| 643965 | TMEM88B | 5.247927513 | | 5.247927513 | -5.247927513 | |
| 9635 | CLCA2 | 5.247927513 | | 5.247927513 | -5.247927513 | |
| 8542 | APOL1 | 5.247927513 | | 5.247927513 | -5.247927513 | |
| 79827 | CLMP | 5.247927513 | | 5.247927513 | -5.247927513 | |
| 284390 | ZNF763 | 5.247927513 | | 5.247927513 | -5.247927513 | |
| 2560 | GABRB1 | 5.247927513 | | 5.247927513 | -5.247927513 | |
| 84645 | C22orf23 | 5.247927513 | | 5.247927513 | -5.247927513 | |
| 6854 | SYN2 | 5.247927513 | | 5.247927513 | -5.247927513 | |
| 163351 | GBP6 | 5.247927513 | | 5.247927513 | -5.247927513 | |
| 112817 | HOGA1 | 5.247927513 | | 5.247927513 | -5.247927513 | |
| 100129827 | MRVI1-AS1 | 5.247927513 | | 5.247927513 | -5.247927513 | |
| 30816 | ERVW-1 | 5.247927513 | | 5.247927513 | -5.247927513 | |
| 65268 | WNK2 | 5.247927513 | | 5.247927513 | -5.247927513 | |
| 629 | CFB | 5.247927513 | | 5.247927513 | -5.247927513 | |
| 100134869 | UBE2Q2P2 | 5.247927513 | | 5.247927513 | -5.247927513 | |
| 6000 | RGS7 | 5.247927513 | | 5.247927513 | -5.247927513 | |
| 8787 | RGS9 | 5.247927513 | | 5.247927513 | -5.247927513 | |
| 117583 | PARD3B | 5.247927513 | | 5.247927513 | -5.247927513 | |
| 482 | ATP1B2 | 5.247927513 | | 5.247927513 | -5.247927513 | |
| 7429 | VIL1 | 5.247927513 | | 5.247927513 | -5.247927513 | |
| 222389 | BEND7 | 5.247927513 | | 5.247927513 | -5.247927513 | |
| 218 | ALDH3A1 | 5.247927513 | | 5.247927513 | -5.247927513 | |
| 773 | CACNA1A | 5.247927513 | | 5.247927513 | -5.247927513 | |
| 8358 | HIST1H3B | 5.247927513 | | 5.247927513 | -5.247927513 | |
| 374786 | EFCAB5 | 5.247927513 | | 5.247927513 | -5.247927513 | |
| 5603 | MAPK13 | 5.247927513 | | 5.247927513 | -5.247927513 | |
| 65217 | PCDH15 | 5.247927513 | | 5.247927513 | -5.247927513 | |
| 403315 | FAM92A1P2 | 5.247927513 | | 5.247927513 | -5.247927513 | |
| 389332 | LOC389332 | 5.247927513 | | 5.247927513 | -5.247927513 | |
| 5649 | RELN | 5.247927513 | | 5.247927513 | -5.247927513 | |
| 79623 | GALNT14 | 5.247927513 | | 5.247927513 | -5.247927513 | |
| 116443 | GRIN3A | 5.247927513 | | 5.247927513 | -5.247927513 | |
| 90338 | ZNF160 | 5.247927513 | | 5.247927513 | -5.247927513 | |
| 3781 | KCNN2 | 5.247927513 | | 5.247927513 | -5.247927513 | |
| 90187 | EMILIN3 | 5.247927513 | | 5.247927513 | -5.247927513 | |
| 643719 | SCGB1B2P | 5.247927513 | | 5.247927513 | -5.247927513 | |
| 119395 | CALHM3 | 5.247927513 | | 5.247927513 | -5.247927513 | |
| 401494 | PTPLAD2 | 5.247927513 | | 5.247927513 | -5.247927513 | |
| 53833 | IL20RB | 5.247927513 | | 5.247927513 | -5.247927513 | |
| 4440 | MSI1 | 5.247927513 | | 5.247927513 | -5.247927513 | |
| 150197 | LOC150197 | 5.247927513 | | 5.247927513 | -5.247927513 | |
| 353500 | BMP8A | 5.247927513 | | 5.247927513 | -5.247927513 | |
| 2053 | EPHX2 | 5.247927513 | | 5.247927513 | -5.247927513 | |
| 123099 | DEGS2 | 5.247927513 | | 5.247927513 | -5.247927513 | |
| 400830 | DEFB132 | 5.247927513 | | 5.247927513 | -5.247927513 | |
| 282763 | OR51B5 | 5.247927513 | | 5.247927513 | -5.247927513 | |
| 401081 | FLJ22763 | 5.247927513 | | 5.247927513 | -5.247927513 | |
| 116535 | MRGPRF | 5.247927513 | | 5.247927513 | -5.247927513 | |
| 79132 | DHX58 | 5.247927513 | | 5.247927513 | -5.247927513 | |
| 79411 | GLB1L | 5.247927513 | | 5.247927513 | -5.247927513 | |
| 389813 | C9orf172 | 5.247927513 | | 5.247927513 | -5.247927513 | |
| 27124 | INPP5J | 5.247927513 | | 5.247927513 | -5.247927513 | |
| 7454 | WAS | 5.247927513 | | 5.247927513 | -5.247927513 | |
| 81854 | MGC3771 | 5.247927513 | | 5.247927513 | -5.247927513 | |
| 84734 | FAM167B | 5.247927513 | | 5.247927513 | -5.247927513 | |
| 1269 | CNR2 | 5.247927513 | | 5.247927513 | -5.247927513 | |
| 56477 | CCL28 | 5.247927513 | | 5.247927513 | -5.247927513 | |
| 3248 | HPGD | 5.247927513 | | 5.247927513 | -5.247927513 | |
| 692085 | SNORD45C | 5.247927513 | | 5.247927513 | -5.247927513 | |
| 6374 | CXCL5 | 5.247927513 | | 5.247927513 | -5.247927513 | |
| 22798 | LAMB4 | 5.247927513 | | 5.247927513 | -5.247927513 | |
| 4629 | MYH11 | 5.247927513 | | 5.247927513 | -5.247927513 | |
| 4359 | MPZ | 5.247927513 | | 5.247927513 | -5.247927513 | |
| 650 | BMP2 | 5.247927513 | | 5.247927513 | -5.247927513 | |
| 117286 | CIB3 | 5.247927513 | | 5.247927513 | -5.247927513 | |
| 7832 | BTG2 | 5.247927513 | | 5.247927513 | -5.247927513 | |
| 10788 | IQGAP2 | 5.247927513 | | 5.247927513 | -5.247927513 | |
| 92359 | CRB3 | 5.247927513 | | 5.247927513 | -5.247927513 | |
| 116844 | LRG1 | 5.247927513 | | 5.247927513 | -5.247927513 | |
| 164668 | APOBEC3H | 5.247927513 | | 5.247927513 | -5.247927513 | |
| 362 | AQP5 | 5.247927513 | | 5.247927513 | -5.247927513 | |
| 286530 | P2RY8 | 5.247927513 | | 5.247927513 | -5.247927513 | |
| 64881 | PCDH20 | 5.247927513 | | 5.247927513 | -5.247927513 | |
| 374877 | C19orf45 | 5.247927513 | | 5.247927513 | -5.247927513 | |
| 79012 | CAMKV | 5.247927513 | | 5.247927513 | -5.247927513 | |
| 389812 | LCN15 | 5.247927513 | | 5.247927513 | -5.247927513 | |
| 162494 | RHBDL3 | 5.247927513 | | 5.247927513 | -5.247927513 | |
| 56127 | PCDHB9 | 5.247927513 | | 5.247927513 | -5.247927513 | |
| 23046 | KIF21B | 5.247927513 | | 5.247927513 | -5.247927513 | |
| 387486 | LINC00320 | 5.247927513 | | 5.247927513 | -5.247927513 | |
| 153770 | PLAC8L1 | 5.247927513 | | 5.247927513 | -5.247927513 | |
| 7096 | TLR1 | 5.247927513 | | 5.247927513 | -5.247927513 | |
| 56171 | DNAH7 | 5.247927513 | | 5.247927513 | -5.247927513 | |
| 9215 | LARGE | 5.247927513 | | 5.247927513 | -5.247927513 | |
| 130576 | LYPD6B | 5.247927513 | | 5.247927513 | -5.247927513 | |
| 5016 | OVGP1 | 5.247927513 | | 5.247927513 | -5.247927513 | |
| 441425 | ANKRD20A3 | 5.247927513 | | 5.247927513 | -5.247927513 | |
| 136853 | SRCRB4D | 5.247927513 | | 5.247927513 | -5.247927513 | |
| 10157 | AASS | 5.247927513 | | 5.247927513 | -5.247927513 | |
| 255057 | C19orf26 | 5.247927513 | | 5.247927513 | -5.247927513 | |
| 6439 | SFTPB | 5.247927513 | | 5.247927513 | -5.247927513 | |
| 195977 | ANTXRL | 5.247927513 | | 5.247927513 | -5.247927513 | |
| 7051 | TGM1 | 5.247927513 | | 5.247927513 | -5.247927513 | |
| 51299 | NRN1 | 5.247927513 | | 5.247927513 | -5.247927513 | |
| 10417 | SPON2 | 5.247927513 | | 5.247927513 | -5.247927513 | |
| 5555 | PRH2 | 5.247927513 | | 5.247927513 | -5.247927513 | |
| 26797 | SNORD52 | 5.247927513 | | 5.247927513 | -5.247927513 | |
| 255809 | C19orf38 | 5.247927513 | | 5.247927513 | -5.247927513 | |
| 387742 | FAM99A | 5.247927513 | | 5.247927513 | -5.247927513 | |
| 84174 | SLA2 | 5.247927513 | | 5.247927513 | -5.247927513 | |
| 84894 | LINGO1 | 5.247927513 | | 5.247927513 | -5.247927513 | |
| 343450 | KCNT2 | 5.247927513 | | 5.247927513 | -5.247927513 | |
| 3560 | IL2RB | 5.247927513 | | 5.247927513 | -5.247927513 | |
| 29765 | TMOD4 | 5.247927513 | | 5.247927513 | -5.247927513 | |
| 83650 | SLC35G5 | 5.247927513 | | 5.247927513 | -5.247927513 | |
| 123591 | C15orf27 | 5.247927513 | | 5.247927513 | -5.247927513 | |
| 286367 | LOC286367 | 5.247927513 | | 5.247927513 | -5.247927513 | |
| 10562 | OLFM4 | 5.247927513 | | 5.247927513 | -5.247927513 | |
| 92346 | C1orf105 | 5.247927513 | | 5.247927513 | -5.247927513 | |
| 130574 | LYPD6 | 5.247927513 | | 5.247927513 | -5.247927513 | |
| 8997 | KALRN | 5.247927513 | | 5.247927513 | -5.247927513 | |
| 400655 | LOC400655 | 5.247927513 | | 5.247927513 | -5.247927513 | |
| 647323 | LOC647323 | 5.247927513 | | 5.247927513 | -5.247927513 | |
| 151056 | PLB1 | 5.247927513 | | 5.247927513 | -5.247927513 | |
| 491 | ATP2B2 | 5.247927513 | | 5.247927513 | -5.247927513 | |
| 100129354 | NRADDP | 5.247927513 | | 5.247927513 | -5.247927513 | |
| 1588 | CYP19A1 | 5.247927513 | | 5.247927513 | -5.247927513 | |
| 56920 | SEMA3G | 5.247927513 | | 5.247927513 | -5.247927513 | |
| 56256 | SERTAD4 | 5.247927513 | | 5.247927513 | -5.247927513 | |
| 1821 | DRP2 | 5.247927513 | | 5.247927513 | -5.247927513 | |
| 284100 | LOC284100 | 5.247927513 | | 5.247927513 | -5.247927513 | |
| 8326 | FZD9 | 5.247927513 | | 5.247927513 | -5.247927513 | |
| 114599 | SNORD15B | 5.247927513 | | 5.247927513 | -5.247927513 | |
| 145820 | LOC145820 | 5.247927513 | | 5.247927513 | -5.247927513 | |
| 171022 | ABHD11-AS1 | 5.247927513 | | 5.247927513 | -5.247927513 | |
| 146439 | CCDC64B | 5.247927513 | | 5.247927513 | -5.247927513 | |
| 11309 | SLCO2B1 | 5.247927513 | | 5.247927513 | -5.247927513 | |
| 654466 | KGFLP2 | 5.247927513 | | 5.247927513 | -5.247927513 | |
| 5176 | SERPINF1 | 5.247927513 | | 5.247927513 | -5.247927513 | |
| 2239 | GPC4 | 5.247927513 | | 5.247927513 | -5.247927513 | |
| 4858 | NOVA2 | 5.247927513 | | 5.247927513 | -5.247927513 | |
| 1800 | DPEP1 | 5.247927513 | | 5.247927513 | -5.247927513 | |
| 54436 | SH3TC1 | 5.247927513 | | 5.247927513 | -5.247927513 | |
| 56000 | NXF3 | 5.247927513 | | 5.247927513 | -5.247927513 | |
| 388697 | HRNR | 5.247927513 | | 5.247927513 | -5.247927513 | |
| 2868 | GRK4 | 5.247927513 | | 5.247927513 | -5.247927513 | |
| 284805 | C20orf203 | 5.247927513 | | 5.247927513 | -5.247927513 | |
| 145226 | RDH12 | 5.247927513 | | 5.247927513 | -5.247927513 | |
| 5999 | RGS4 | 5.247927513 | | 5.247927513 | -5.247927513 | |
| 132158 | GLYCTK | 5.247927513 | | 5.247927513 | -5.247927513 | |
| 2529 | FUT7 | 5.247927513 | | 5.247927513 | -5.247927513 | |
| 390195 | OR5AN1 | 5.247927513 | | 5.247927513 | -5.247927513 | |
| 786 | CACNG1 | 5.247927513 | | 5.247927513 | -5.247927513 | |
| 284186 | TMEM105 | 5.247927513 | | 5.247927513 | -5.247927513 | |
| 51062 | ATL1 | 5.247927513 | | 5.247927513 | -5.247927513 | |
| 440836 | ODF3B | 5.247927513 | | 5.247927513 | -5.247927513 | |
| 133491 | C5orf47 | 5.247927513 | | 5.247927513 | -5.247927513 | |
| 7067 | THRA | 5.030521945 | | 1.455082218 | -1.461465835 | |
| 80307 | FER1L4 | 4.609531222 | | 3.949467266 | -2.366829818 | |
| 23031 | MAST3 | 4.508628809 | | 1.712162203 | -1.130847479 | |
| 12 | SERPINA3 | 4.473317145 | | 2.544090219 | -3.207727034 | |
| 79661 | NEIL1 | 4.233871918 | | 3.648909418 | -2.661620469 | |
| 6038 | RNASE4 | 4.195015982 | | 1.506305557 | -2.622764533 | |
| 9536 | PTGES | 4.15508451 | | 1.993621087 | -2.174193333 | |
| 5163 | PDK1 | 4.091981858 | | 1.691443929 | -2.28188156 | |
| 51754 | TMEM8B | 3.918863237 | | 2.48781342 | -1.246437895 | |
| 5064 | PALM | 3.862616696 | | 3.536846535 | -2.69055595 | |
| 80221 | ACSF2 | 3.844829627 | | 3.259867127 | -3.844829627 | |
| 388228 | SBK1 | 3.749151133 | | 1.411847812 | -1.625768717 | |
| 168544 | ZNF467 | 3.740757173 | | 8.988684687 | -8.988684687 | |
| 123688 | AGPHD1 | 3.740757173 | | 2.434095835 | -1.168505724 | |
| 230 | ALDOC | 3.712228916 | | 4.120868644 | -3.461267342 | |
| 29842 | TFCP2L1 | 3.685763142 | | 2.704871964 | -2.704871964 | |
| 729359 | PLIN4 | 3.685763142 | | 3.685763142 | -3.685763142 | |
| 125965 | COX6B2 | 3.628589433 | | 1.832122827 | -2.321928095 | |
| 64122 | FN3K | 3.500621586 | | 2.500621586 | -2.181249196 | |
| 2550 | GABBR1 | 3.497399469 | | 9.726218159 | -2.032731202 | |
| 376497 | SLC27A1 | 3.462532523 | | 3.043232997 | -1.17166844 | |
| 1902 | LPAR1 | 3.445559444 | | 1.131244533 | -8.693486957 | |
| 1731 | 1-Sep | 3.445559444 | | 1.873307995 | -1.292607521 | |
| 57715 | SEMA4G | 3.426264755 | | 3.400729663 | -3.004800986 | |
| 2921 | CXCL3 | 3.37778133 | | 1.805529881 | -2.818353921 | |
| 51148 | CERCAM | 3.337194852 | | 1.900261857 | -2.490904267 | |
| 4883 | NPR3 | 3.285402219 | | 1.156119202 | -1.399270183 | |
| 5582 | PRKCG | 3.285402219 | | 1.683366205 | -2.86393845 | |
| 27165 | GLS2 | 3.231852751 | | 2.64689025 | -2.250961574 | |
| 2036 | EPB41L1 | 3.222785687 | | 2.211814569 | -1.934280594 | |
| 51161 | C3orf18 | 3.19523609 | | 1.456994339 | -1.321928095 | |
| 5210 | PFKFB4 | 3.154735122 | | 2.59964315 | -2.851852782 | |
| 4303 | FOXO4 | 3.074000581 | | 1.083523356 | -2.093109404 | |
| 401303 | ZNF815P | 3.074000581 | | 8.321928095 | -1.501749132 | |
| 924 | CD7 | 3.074000581 | | 8.321928095 | -2.514573173 | |
| 80164 | FLJ22184 | 3.069162025 | | 2.647698256 | -1.647698256 | |
| 58985 | IL22RA1 | 3.069162025 | | 1.638112207 | -2.056337984 | |
| 219654 | ZCCHC24 | 3.027389998 | | 2.020194497 | -1.268944676 | |
| 26232 | FBXO2 | 3.019058336 | | 1.166379423 | -2.434095835 | |
| 197257 | LDHD | 2.985692163 | | 1.679030825 | -1.004800986 | |
| 275 | AMT | 2.985692163 | | 2.004800986 | -2.004800986 | |
| 7108 | TM7SF2 | 2.975798941 | | 2.970323811 | -2.975798941 | |
| 575 | BAI1 | 2.958533383 | | 2.948947334 | -1.865423978 | |
| 10675 | CSPG5 | 2.92721058 | | 3.252980741 | -1.342248079 | |
| 388588 | LOC388588 | 2.921951104 | | 1.97987108 | -1.636548885 | |
| 100507421 | LOC100507421 | 2.86393845 | | 2.048363022 | -3.844829627 | |
| 89848 | FCHSD1 | 2.815199939 | | 2.028443978 | -1.294034089 | |
| 5864 | RAB3A | 2.790991476 | | 1.484330138 | -2.231564067 | |
| 83546 | RTBDN | 2.790991476 | | 2.206028975 | -1.810100299 | |
| 322 | APBB1 | 2.754792591 | | 1.724365557 | -1.216708783 | |
| 25837 | RAB26 | 2.753708348 | | 2.474848975 | -2.024187566 | |
| 80235 | PIGZ | 2.723860607 | | 1.458270496 | -2.458270496 | |
| 81544 | GDPD5 | 2.704871964 | | 3.685763142 | -1.889296536 | |
| 4987 | OPRL1 | 2.69945729 | | 1.475242133 | -1.286016576 | |
| 2065 | ERBB3 | 2.688710426 | | 1.707819249 | -2.688710426 | |
| 138311 | FAM69B | 2.609334719 | | 1.187101718 | -2.189496233 | |
| 26297 | SERGEF | 2.574657492 | | 1.114759209 | -1.306071789 | |
| 3643 | INSR | 2.572251449 | | 1.591360272 | -7.820178962 | |
| 728730 | LOC728730 | 2.572251449 | | 1.987288948 | -1.265590111 | |
| 283120 | H19 | 2.562706002 | | 3.024811755 | -2.269706175 | |
| 85315 | PAQR8 | 2.56175511 | | 2.071949842 | -1.239827015 | |
| 79816 | TLE6 | 2.538168289 | | 1.530514717 | -2.86393845 | |
| 1999 | ELF3 | 2.503179096 | | 1.646658117 | -1.777801476 | |
| 119391 | GSTO2 | 2.4870703 | | 2.221480189 | -1.110921814 | |
| 10014 | HDAC5 | 2.478406399 | | 2.755515991 | -1.685126663 | |
| 348487 | FAM131C | 2.458270496 | | 2.040044719 | -1.044829781 | |
| 57326 | PBXIP1 | 2.447743504 | | 1.522193453 | -2.294034089 | |
| 150291 | MORC2-AS1 | 2.445559444 | | 7.693486957 | -1.138898106 | |
| 5144 | PDE4D | 2.445559444 | | 1.860596943 | -7.693486957 | |
| 125875 | CLDND2 | 2.445559444 | | 2.445559444 | -1.886132035 | |
| 54739 | XAF1 | 2.445559444 | | 1.860596943 | -1.464668267 | |
| 4329 | ALDH6A1 | 2.426264755 | | 2.985692163 | -1.004800986 | |
| 125058 | TBC1D16 | 2.426264755 | | 2.985692163 | -8.233619677 | |
| 114088 | TRIM9 | 2.426264755 | | 2.004800986 | -1.413440714 | |
| 4355 | MPP2 | 2.426264755 | | 2.004800986 | -2.004800986 | |
| 1831 | TSC22D3 | 2.413440714 | | 2.189225557 | -1.83274024 | |
| 50651 | SLC45A1 | 2.409520494 | | 3.214124805 | -1.622764533 | |
| 1838 | DTNB | 2.406860843 | | 1.723044955 | -1.206562192 | |
| 29911 | HOOK2 | 2.400739484 | | 1.007296275 | -1.076448252 | |
| 83543 | AIF1L | 2.387023123 | | 1.090935033 | -3.559083869 | |
| 566 | AZU1 | 2.373570882 | | 2.625109649 | -1.56164323 | |
| 664 | BNIP3 | 2.36761714 | | 1.414217553 | -1.511023434 | |
| 534 | ATP6V1G2 | 2.33219643 | | 2.891623839 | -2.33219643 | |
| 489 | ATP2A3 | 2.328914525 | | 2.321928095 | -1.8758637 | |
| 64077 | LHPP | 2.324486822 | | 1.131041654 | -2.170777406 | |
| 283335 | LOC283335 | 2.306661338 | | 2.306661338 | -1.74723393 | |
| 116843 | SLC18B1 | 2.306661338 | | 1.325770161 | -1.74723393 | |
| 79917 | MAGIX | 2.306661338 | | 1.325770161 | -7.554588852 | |
| 27076 | LYPD3 | 2.306661338 | | 7.554588852 | -2.306661338 | |
| 41 | ASIC1 | 2.282933963 | | 1.954097499 | -1.600061072 | |
| 11156 | PTP4A3 | 2.272578179 | | 2.86393845 | -2.048363022 | |
| 25878 | MXRA5 | 2.265590111 | | 1.581774223 | -3.01282404 | |
| 25809 | TTLL1 | 2.250961574 | | 1.925191412 | -1.435386145 | |
| 9886 | RHOBTB1 | 2.250961574 | | 1.925191412 | -1.250961574 | |
| 1606 | DGKA | 2.249277972 | | 2.821461868 | -1.021760519 | |
| 84189 | SLITRK6 | 2.231564067 | | 8.038918989 | -1.810100299 | |
| 148252 | DIRAS1 | 2.183429266 | | 2.095700416 | -1.267222203 | |
| 2264 | FGFR4 | 2.176322773 | | 8.405141463 | -2.176322773 | |
| 2781 | GNAZ | 2.176322773 | | 1.584962501 | -1.584962501 | |
| 7754 | ZNF204P | 2.176322773 | | 1.850552611 | -2.597786541 | |
| 55200 | PLEKHG6 | 2.152951923 | | 1.172060746 | -7.400879436 | |
| 51268 | PIPOX | 2.152951923 | | 7.400879436 | -1.172060746 | |
| 9980 | DOPEY2 | 2.152951923 | | 7.400879436 | -1.593524514 | |
| 3202 | HOXA5 | 2.152951923 | | 2.152951923 | -7.400879436 | |
| 60489 | APOBEC3G | 2.152951923 | | 1.567989422 | -1.593524514 | |
| 51129 | ANGPTL4 | 2.151330971 | | 1.17973761 | -2.323054211 | |
| 6919 | TCEA2 | 2.143820599 | | 1.136563045 | -1.26294033 | |
| 83450 | LRRC48 | 2.129283017 | | 2.103747925 | -1.382049087 | |
| 58191 | CXCL16 | 2.129283017 | | 1.116458977 | -1.707819249 | |
| 29121 | CLEC2D | 2.113511693 | | 1.524299719 | -1.889296536 | |
| 114879 | OSBPL5 | 2.113511693 | | 2.113511693 | -2.704871964 | |
| 5165 | PDK3 | 2.101910781 | | 1.996171528 | -1.152145084 | |
| 9744 | ACAP1 | 2.101402032 | | 2.74350844 | -1.648351207 | |
| 3718 | JAK3 | 2.093109404 | | 2.501749132 | -1.767339243 | |
| 2941 | GSTA4 | 2.093109404 | | 2.093109404 | -2.093109404 | |
| 11240 | PADI2 | 2.069704549 | | 3.390383016 | -2.127088408 | |
| 51090 | PLLP | 2.059334139 | | 1.399270183 | -2.86393845 | |
| 83937 | RASSF4 | 2.049630768 | | 2.723860607 | -1.347712121 | |
| 9563 | H6PD | 2.025796001 | | 2.219149498 | -1.424401716 | |
| 154091 | SLC2A12 | 2.022236474 | | 1.187101718 | -1.283564558 | |
| 170961 | ANKRD24 | 2.01282404 | | 7.820178962 | -1.265590111 | |
| 256949 | KANK3 | 2.01282404 | | 7.820178962 | -1.265590111 | |
| 148170 | CDC42EP5 | 2.01282404 | | 1.987288948 | -1.265590111 | |
| 79867 | TCTN2 | 2.01282404 | | 1.591360272 | -2.01282404 | |
| 7137 | TNNI3 | 2.00823615 | | 1.105021213 | -1.110921814 | |
| 8448 | DOC2A | 2.00698643 | | 2.455082218 | -2.464668267 | |
| 3791 | KDR | 2.004800986 | | 8.233619677 | -1.679030825 | |
| 221914 | GPC2 | 2.004800986 | | 1.057030945 | -1.263993326 | |
| 9537 | TP53I11 | 2.003198882 | | 1.827060331 | -1.971524831 | |
| 64788 | LMF1 | 2.0031977 | | 1.845656423 | -1.170457459 | |
| 6319 | SCD | 2.001339485 | | 2.359059013 | -2.418288289 | |
| 727 | C5 | 2 | | 1.734409889 | -1.325770161 | |
| 158219 | TTC39B | 1.990477226 | | 7.238404739 | -1.431049817 | |
| 388743 | CAPN8 | 1.990477226 | | 7.238404739 | -7.238404739 | |
| 10148 | EBI3 | 1.990477226 | | 1.009586049 | -1.431049817 | |
| 5787 | PTPRB | 1.990477226 | | 7.238404739 | -7.238404739 | |
| 1382 | CRABP2 | 1.98960408 | | 2.99182243 | -1.71105166 | |
| 2026 | ENO2 | 1.979506561 | | 1.739302864 | -1.17899515 | |
| 85026 | C9orf37 | 1.973495365 | | 1.030639272 | -3.105407041 | |
| 54885 | TBC1D8B | 1.958533383 | | 1.625109649 | -1.256614736 | |
| 10610 | ST6GALNAC2 | 1.939208316 | | 2.063466419 | -2.581314724 | |
| 93 | ACVR2B | 1.938044252 | | 1.348832279 | -2.950868293 | |
| 6196 | RPS6KA2 | 1.925191412 | | 1.659601302 | -1.435386145 | |
| 11037 | STON1 | 1.925191412 | | 2.250961574 | -8.479780264 | |
| 5033 | P4HA1 | 1.924247778 | | 2.195621484 | -1.638525777 | |
| 8991 | SELENBP1 | 1.921048659 | | 1.501749132 | -2.093109404 | |
| 9249 | DHRS3 | 1.920781553 | | 2.298148635 | -1.997194669 | |
| 644172 | LOC644172 | 1.910732662 | | 8.139551352 | -1.095157233 | |
| 22846 | VASH1 | 1.910732662 | | 8.139551352 | -2.33219643 | |
| 25850 | ZNF345 | 1.886132035 | | 2.445559444 | -7.693486957 | |
| 284751 | LOC284751 | 1.886132035 | | 2.445559444 | -1.138898106 | |
| 6289 | SAA2 | 1.886132035 | | 1.138898106 | -1.886132035 | |
| 414918 | FAM116B | 1.886132035 | | 7.693486957 | -1.886132035 | |
| 54800 | KLHL24 | 1.869939459 | | 2.777961137 | -2.148433084 | |
| 23179 | RGL1 | 1.86393845 | | 1.272578179 | -1.272578179 | |
| 57555 | NLGN2 | 1.858760535 | | 2.13177903 | -1.451659296 | |
| 10279 | PRSS16 | 1.857165222 | | 1.967723597 | -1.387023123 | |
| 153768 | PRELID2 | 1.850552611 | | 1.850552611 | -3.15721395 | |
| 339942 | H1FX-AS1 | 1.850552611 | | 1.850552611 | -2.597786541 | |
| 9509 | ADAMTS2 | 1.850552611 | | 2.176322773 | -1.584962501 | |
| 84063 | KIRREL2 | 1.848423172 | | 2.848423172 | -1.709525066 | |
| 1299 | COL9A3 | 1.848423172 | | 1.993621087 | -1.472274686 | |
| 50853 | VILL | 1.843093945 | | 2.647698256 | -3.628589433 | |
| 408 | ARRB1 | 1.819519276 | | 1.371903408 | -1.637010156 | |
| 6528 | SLC5A5 | 1.811927652 | | 2.063466419 | -1.586789854 | |
| 283870 | C16orf79 | 1.810100299 | | 2.206028975 | -8.038918989 | |
| 11118 | BTN3A2 | 1.799264335 | | 2.240203697 | -1.61176256 | |
| 7033 | TFF3 | 1.796466606 | | 1.796466606 | -7.044394119 | |
| 55160 | ARHGEF10L | 1.796466606 | | 7.044394119 | -7.044394119 | |
| 91523 | PCED1B | 1.796466606 | | 1.211504105 | -7.044394119 | |
| 400619 | LINC00511 | 1.796466606 | | 1.796466606 | -1.796466606 | |
| 222183 | SRRM3 | 1.796466606 | | 1.796466606 | -1.796466606 | |
| 10391 | CORO2B | 1.796466606 | | 7.044394119 | -7.044394119 | |
| 140825 | NEURL2 | 1.796466606 | | 7.044394119 | -1.796466606 | |
| 929 | CD14 | 1.796466606 | | 7.044394119 | -1.796466606 | |
| 3696 | ITGB8 | 1.796466606 | | 1.796466606 | -7.044394119 | |
| 166012 | CHST13 | 1.796466606 | | 7.044394119 | -7.044394119 | |
| 27112 | FAM155B | 1.796466606 | | 7.044394119 | -1.237039197 | |
| 1365 | CLDN3 | 1.759865996 | | 1.052046748 | -1.434095835 | |
| 123 | PLIN2 | 1.751797333 | | 1.009792877 | -1.061597571 | |
| 8718 | TNFRSF25 | 1.74723393 | | 1.325770161 | -7.554588852 | |
| 144406 | WDR66 | 1.74723393 | | 1.721698838 | -1.74723393 | |
| 114899 | C1QTNF3 | 1.74723393 | | 2.306661338 | -2.306661338 | |
| 445 | ASS1 | 1.74723393 | | 7.554588852 | -7.554588852 | |
| 92840 | REEP6 | 1.738671916 | | 2.095157233 | -1.100632363 | |
| 6508 | SLC4A3 | 1.738671916 | | 2.095157233 | -1.31937239 | |
| 28986 | MAGEH1 | 1.736965594 | | 1.013251023 | -1.218550355 | |
| 11094 | CACFD1 | 1.734409889 | | 1.145197916 | -1.734409889 | |
| 84961 | FBXL20 | 1.734409889 | | 2.721698838 | -1.510194732 | |
| 64221 | ROBO3 | 1.733117939 | | 1.098935364 | -2.492378573 | |
| 23492 | CBX7 | 1.729258555 | | 1.176180115 | -1.621076903 | |
| 79885 | HDAC11 | 1.717600269 | | 1.880329769 | -1.026231542 | |
| 9649 | RALGPS1 | 1.71419662 | | 2.286380516 | -1.155135983 | |
| 11067 | C10orf10 | 1.707819249 | | 7.936637939 | -2.129283017 | |
| 51450 | PRRX2 | 1.70761477 | | 2.64232331 | -1.907913421 | |
| 421 | ARVCF | 1.704871964 | | 3.100800641 | -1.113511693 | |
| 23108 | RAP1GAP2 | 1.686520172 | | 2.172756592 | -1.258891433 | |
| 8226 | HDHD1 | 1.679030825 | | 1.189225557 | -1.189225557 | |
| 57333 | RCN3 | 1.679030825 | | 1.679030825 | -2.426264755 | |
| 1952 | CELSR2 | 1.676568315 | | 1.968749066 | -2.085208043 | |
| 7059 | THBS3 | 1.659601302 | | 1.659601302 | -2.250961574 | |
| 641649 | TMEM91 | 1.659601302 | | 1.070389328 | -1.250961574 | |
| 23475 | QPRT | 1.659601302 | | 2.250961574 | -1.078900828 | |
| 949 | SCARB1 | 1.649948304 | | 2.430263974 | -3 | |
| 9311 | ASIC3 | 1.640779715 | | 1.479416727 | -1.002740162 | |
| 978 | CDA | 1.640779715 | | 2.812840461 | -3.23430423 | |
| 1852 | DUSP9 | 1.628935126 | | 2.052046748 | -1.141627341 | |
| 65018 | PINK1 | 1.624881511 | | 1.021723478 | -1.410357978 | |
| 8839 | WISP2 | 1.620398897 | | 2.612731767 | -2.06564649 | |
| 1759 | DNM1 | 1.615254574 | | 2.193292551 | -2.781446856 | |
| 100527964 | LOC100527964 | 1.593524514 | | 1.172060746 | -2.152951923 | |
| 54756 | IL17RD | 1.593524514 | | 7.400879436 | -1.593524514 | |
| 27152 | INTU | 1.593524514 | | 1.567989422 | -1.172060746 | |
| 1264 | CNN1 | 1.593524514 | | 2.152951923 | -1.172060746 | |
| 100272217 | LOC100272217 | 1.592285842 | | 2.071119991 | -2.818353921 | |
| 57593 | EBF4 | 1.592285842 | | 8.625708843 | -1.396890153 | |
| 85458 | DIXDC1 | 1.591948931 | | 2.040044719 | -2.458270496 | |
| 30008 | EFEMP2 | 1.591948931 | | 4.030521945 | -2.723860607 | |
| 27128 | CYTH4 | 1.591791839 | | 1.68182404 | -1.404290064 | |
| 79777 | ACBD4 | 1.591360272 | | 7.820178962 | -2.01282404 | |
| 80231 | CXorf21 | 1.591360272 | | 2.572251449 | -7.820178962 | |
| 7903 | ST8SIA4 | 1.591360272 | | 1.591360272 | -7.820178962 | |
| 338707 | B4GALNT4 | 1.588243592 | | 2.008546407 | -3.283764467 | |
| 21 | ABCA3 | 1.58780525 | | 1.876679661 | -1.130703692 | |
| 23331 | TTC28 | 1.586789854 | | 1.141893066 | -1.694971506 | |
| 57010 | CABP4 | 1.584962501 | | 1.584962501 | -1.360747344 | |
| 147700 | KLC3 | 1.572251449 | | 1.572251449 | -6.820178962 | |
| 51364 | ZMYND10 | 1.572251449 | | 1.572251449 | -1.01282404 | |
| 113451 | ADC | 1.572251449 | | 6.820178962 | -1.572251449 | |
| 374500 | THSD1P1 | 1.572251449 | | 1.572251449 | -1.01282404 | |
| 219731 | LOC219731 | 1.572251449 | | 6.820178962 | -6.820178962 | |
| 204801 | NLRP11 | 1.572251449 | | 6.820178962 | -6.820178962 | |
| 27294 | DHDH | 1.572251449 | | 6.820178962 | -6.820178962 | |
| 9912 | ARHGAP44 | 1.572251449 | | 6.820178962 | -1.572251449 | |
| 10850 | CCL27 | 1.572251449 | | 6.820178962 | -1.572251449 | |
| 149840 | C20orf196 | 1.572251449 | | 6.820178962 | -6.820178962 | |
| 257407 | C2orf72 | 1.572251449 | | 6.820178962 | -6.820178962 | |
| 53358 | SHC3 | 1.572251449 | | 6.820178962 | -6.820178962 | |
| 115704 | EVI5L | 1.566210717 | | 1.772392911 | -1.701619837 | |
| 157638 | FAM84B | 1.559083869 | | 1.54949782 | -1.033015057 | |
| 6376 | CX3CL1 | 1.548162583 | | 1.035514287 | -2.317549654 | |
| 388650 | FAM69A | 1.543621705 | | 1.536652151 | -1.299929022 | |
| 79957 | PAQR6 | 1.538168289 | | 9.092757141 | -1.053838152 | |
| 1241 | LTB4R | 1.508146904 | | 1.759685671 | -2.093109404 | |
| 93129 | ORAI3 | 1.504507569 | | 1.666982266 | -2.211900048 | |
| 56977 | STOX2 | 1.501749132 | | 1.501749132 | -1.277533976 | |
| 56895 | AGPAT4 | 1.501321186 | | 1.851067225 | -2.217289699 | |
| 283070 | LOC283070 | 1.497399469 | | 1.581559917 | -1.325338723 | |
| 6857 | SYT1 | 1.496277347 | | 1.46533364 | -1.445067178 | |
| 2281 | FKBP1B | 1.493303253 | | 1.152543483 | -1.658234861 | |
| 284615 | ANKRD34A | 1.4870703 | | 2.4870703 | -1.110921814 | |
| 205 | AK4 | 1.485197937 | | 1.777198872 | -2.067359476 | |
| 83986 | ITFG3 | 1.470243865 | | 1.010628311 | -1.784352455 | |
| 115330 | GPR146 | 1.464668267 | | 7.693486957 | -1.138898106 | |
| 129807 | NEU4 | 1.464668267 | | 1.464668267 | -7.693486957 | |
| 9203 | ZMYM3 | 1.448906168 | | 1.704553777 | -1.011259861 | |
| 9567 | GTPBP1 | 1.447743504 | | 1.0252557 | -1.155135983 | |
| 2934 | GSN | 1.445312982 | | 2.607656212 | -1.236518572 | |
| 6253 | RTN2 | 1.438640841 | | 1.721371049 | -1.063896048 | |
| 84445 | LZTS2 | 1.431206436 | | 9.361943774 | -2.541764811 | |
| 84818 | IL17RC | 1.431177202 | | 1.176180115 | -1.621076903 | |
| 11174 | ADAMTS6 | 1.431049817 | | 1.009586049 | -7.238404739 | |
| 149428 | BNIPL | 1.431049817 | | 1.990477226 | -1.431049817 | |
| 29931 | LINC00312 | 1.431049817 | | 1.990477226 | -1.009586049 | |
| 150000 | ABCC13 | 1.431049817 | | 7.238404739 | -1.990477226 | |
| 158801 | NKAPP1 | 1.431049817 | | 7.238404739 | -7.238404739 | |
| 9028 | RHBDL1 | 1.431049817 | | 1.405514725 | -7.238404739 | |
| 26468 | LHX6 | 1.431049817 | | 1.009586049 | -7.238404739 | |
| 85442 | KNDC1 | 1.431049817 | | 7.238404739 | -7.238404739 | |
| 83959 | SLC4A11 | 1.421588834 | | 1.705789698 | -2.565485126 | |
| 776 | CACNA1D | 1.419299526 | | 1.581774223 | -1.419299526 | |
| 79366 | HMGN5 | 1.419299526 | | 1.257936538 | -1.265590111 | |
| 201625 | DNAH12 | 1.419299526 | | 2.265590111 | -3.572251449 | |
| 8425 | LTBP4 | 1.417730603 | | 2.174236383 | -1.815855277 | |
| 151174 | LOC151174 | 1.417702741 | | 1.057030945 | -3.263993326 | |
| 1512 | CTSH | 1.393707436 | | 1.602856622 | -1.036012979 | |
| 24142 | NAT6 | 1.385209933 | | 1.475242133 | -2.290817562 | |
| 152 | ADRA2C | 1.382049087 | | 1.707819249 | -2.688710426 | |
| 160335 | TMTC2 | 1.382049087 | | 2.688710426 | -2.129283017 | |
| 619383 | SCARNA9 | 1.382049087 | | 1.707819249 | -1.707819249 | |
| 55620 | STAP2 | 1.376091219 | | 1.660947727 | -1.077404504 | |
| 64772 | ENGASE | 1.376074344 | | 1.288345494 | -2 | |
| 8659 | ALDH4A1 | 1.375039431 | | 1.226488081 | -1.528811618 | |
| 30819 | KCNIP2 | 1.371718462 | | 8.405141463 | -1.850552611 | |
| 9001 | HAP1 | 1.371718462 | | 8.405141463 | -1.584962501 | |
| 440253 | WHAMMP2 | 1.371718462 | | 1.584962501 | -3.15721395 | |
| 79774 | GRTP1 | 1.355158624 | | 2.221480189 | -1.221480189 | |
| 78991 | PCYOX1L | 1.35166118 | | 1.483238438 | -1.808039475 | |
| 9479 | MAPK8IP1 | 1.342848747 | | 2.12509522 | -1.063694675 | |
| 26470 | SEZ6L2 | 1.34149854 | | 1.739986574 | -2.169925001 | |
| 10570 | DPYSL4 | 1.327304127 | | 2.184563955 | -1.123163411 | |
| 51385 | ZNF589 | 1.325770161 | | 2.325770161 | -1.153709415 | |
| 8786 | RGS11 | 1.325770161 | | 7.554588852 | -2.306661338 | |
| 25830 | SULT4A1 | 1.325770161 | | 7.554588852 | -1.74723393 | |
| 404550 | C16orf74 | 1.325338723 | | 2.906039197 | -1.906039197 | |
| 116931 | MED12L | 1.31937239 | | 1.910732662 | -1.095157233 | |
| 80320 | SP6 | 1.306661338 | | 1.306661338 | -1.306661338 | |
| 154761 | LOC154761 | 1.306661338 | | 6.554588852 | -1.306661338 | |
| 4804 | NGFR | 1.306661338 | | 6.554588852 | -1.306661338 | |
| 84837 | ARHGAP5-AS1 | 1.306661338 | | 6.554588852 | -1.306661338 | |
| 3910 | LAMA4 | 1.306661338 | | 1.306661338 | -6.554588852 | |
| 374618 | TEX9 | 1.306661338 | | 6.554588852 | -6.554588852 | |
| 1117 | CHI3L2 | 1.306661338 | | 6.554588852 | -6.554588852 | |
| 197320 | ZNF778 | 1.306661338 | | 6.554588852 | -1.306661338 | |
| 9495 | AKAP5 | 1.306661338 | | 6.554588852 | -1.306661338 | |
| 379013 | RNF138P1 | 1.306661338 | | 1.306661338 | -6.554588852 | |
| 728577 | CNTNAP3B | 1.302882339 | | 1.189225557 | -1.194700687 | |
| 4660 | PPP1R12B | 1.302882339 | | 3.004800986 | -1.679030825 | |
| 883 | CCBL1 | 1.30256277 | | 2.296164999 | -1.026231542 | |
| 389792 | IER5L | 1.30256277 | | 1.632528184 | -2.185606624 | |
| 11322 | TMC6 | 1.292607521 | | 1.131244533 | -2.138898106 | |
| 65010 | SLC26A6 | 1.289302947 | | 1.945439063 | -1.70633312 | |
| 283130 | SLC25A45 | 1.288505093 | | 3.074000581 | -1.277533976 | |
| 3306 | HSPA2 | 1.288505093 | | 2.093109404 | -1.277533976 | |
| 284370 | ZNF615 | 1.265590111 | | 1.265590111 | -2.01282404 | |
| 80853 | JHDM1D | 1.265590111 | | 7.820178962 | -2.572251449 | |
| 51222 | ZNF219 | 1.263034406 | | 1.537656786 | -1.310523332 | |
| 63891 | RNF123 | 1.257233254 | | 1.278077508 | -1.20070658 | |
| 26471 | NUPR1 | 1.252307999 | | 2.245910228 | -1.132398536 | |
| 136288 | C7orf57 | 1.250961574 | | 1.659601302 | -1.925191412 | |
| 90139 | TSPAN18 | 1.250961574 | | 2.250961574 | -1.435386145 | |
| 3708 | ITPR1 | 1.243692684 | | 1.375269942 | -1.453910391 | |
| 10188 | TNK2 | 1.243271151 | | 1.08979667 | -1.271349674 | |
| 8519 | IFITM1 | 1.237039197 | | 1.796466606 | -1.796466606 | |
| 112703 | FAM71E1 | 1.237039197 | | 7.044394119 | -1.796466606 | |
| 284348 | LYPD5 | 1.237039197 | | 7.044394119 | -1.796466606 | |
| 144486 | LOC144486 | 1.237039197 | | 1.796466606 | -1.237039197 | |
| 3137 | HLA-J | 1.237039197 | | 7.044394119 | -1.796466606 | |
| 1384 | CRAT | 1.22787796 | | 2.221480189 | -2.4870703 | |
| 81622 | UNC93B1 | 1.226342007 | | 1.589351598 | -1.262683854 | |
| 64132 | XYLT2 | 1.226263698 | | 1.03324179 | -1.281581467 | |
| 284716 | RIMKLA | 1.225767148 | | 2.503062247 | -1.50784731 | |
| 10000 | AKT3 | 1.221914708 | | 1.037022131 | -1.09861451 | |
| 79924 | ADM2 | 1.218740027 | | 8.038918989 | -2.231564067 | |
| 80757 | TMEM121 | 1.218740027 | | 8.038918989 | -2.790991476 | |
| 285512 | FAM13A-AS1 | 1.218740027 | | 1.484330138 | -1.218740027 | |
| 1788 | DNMT3A | 1.217991274 | | 1.047041267 | -1.001322967 | |
| 6513 | SLC2A1 | 1.214500601 | | 2.192474294 | -1.954110916 | |
| 55337 | C19orf66 | 1.214124805 | | 2.03355256 | -1.121015401 | |
| 3727 | JUND | 1.209199821 | | 2.3102103 | -1.863824857 | |
| 122525 | C14orf28 | 1.203634363 | | 1.348832279 | -1.203634363 | |
| 6405 | SEMA3F | 1.201633861 | | 1.078777113 | -1.389706209 | |
| 10385 | BTN2A2 | 1.190016419 | | 1.314274522 | -1.647698256 | |
| 611 | OPN1SW | 1.176322773 | | 2.176322773 | -2.176322773 | |
| 4091 | SMAD6 | 1.174903496 | | 1.750279947 | -1.944290567 | |
| 54716 | SLC6A20 | 1.174903496 | | 1.295197729 | -1.295197729 | |
| 6620 | SNCB | 1.172060746 | | 1.172060746 | -1.172060746 | |
| 23114 | NFASC | 1.172060746 | | 7.400879436 | -2.152951923 | |
| 644890 | MEIG1 | 1.172060746 | | 1.172060746 | -2.152951923 | |
| 7005 | TEAD3 | 1.172060746 | | 1.172060746 | -7.400879436 | |
| 5212 | VIT | 1.172060746 | | 7.400879436 | -2.152951923 | |
| 64711 | HS3ST6 | 1.169147897 | | 1.347546473 | -2.121015401 | |
| 24139 | EML2 | 1.158624969 | | 2.0252557 | -1.258035891 | |
| 3726 | JUNB | 1.158337027 | | 1.772445874 | -2.015596855 | |
| 7001 | PRDX2 | 1.146719377 | | 1.610623004 | -1.659552592 | |
| 5351 | PLOD1 | 1.138900909 | | 1.738591612 | -1.03130191 | |
| 23043 | TNIK | 1.138898106 | | 1.464668267 | -2.445559444 | |
| 192683 | SCAMP5 | 1.138898106 | | 7.693486957 | -1.886132035 | |
| 2217 | FCGRT | 1.135351853 | | 1.304537958 | -1.249105567 | |
| 20 | ABCA2 | 1.134504503 | | 2.347165386 | -3.9492014 | |
| 2549 | GAB1 | 1.116458977 | | 1.382049087 | -2.129283017 | |
| 100 | ADA | 1.116458977 | | 7.936637939 | -7.936637939 | |
| 388963 | C2orf81 | 1.116458977 | | 2.688710426 | -2.688710426 | |
| 23373 | CRTC1 | 1.110921814 | | 2.105021213 | -1.415950309 | |
| 84557 | MAP1LC3A | 1.104656158 | | 1.996171528 | -2.001278418 | |
| 30815 | ST6GALNAC6 | 1.10416634 | | 1.096861539 | -1.100509316 | |
| 100133941 | CD24 | 1.103282817 | | 1.774507557 | -1.025786121 | |
| 79444 | BIRC7 | 1.094104828 | | 2.086433815 | -1.492754097 | |
| 51308 | REEP2 | 1.093109404 | | 1.277533976 | -1.277533976 | |
| 51171 | HSD17B14 | 1.093109404 | | 1.501749132 | -1.767339243 | |
| 54626 | HES2 | 1.086867596 | | 1.36188982 | -1.06527823 | |
| 3783 | KCNN4 | 1.084677715 | | 2.078777113 | -1.321928095 | |
| 254065 | BRWD3 | 1.079254643 | | 1.355901215 | -1.424019062 | |
| 1748 | DLX4 | 1.074962058 | | 1.689070904 | -1.303780748 | |
| 79671 | NLRX1 | 1.074962058 | | 1.996352223 | -1.749191896 | |
| 256472 | TMEM151A | 1.071435138 | | 1.996519431 | -2.873307995 | |
| 126014 | OSCAR | 1.06336635 | | 1.056793696 | -2.492378573 | |
| 100133091 | LOC100133091 | 1.062735755 | | 2.056337984 | -1.832122827 | |
| 100128071 | LOC100128071 | 1.062050027 | | 2.174412392 | -1.267222203 | |
| 79746 | ECHDC3 | 1.060246542 | | 1.889296536 | -1.61176256 | |
| 54813 | KLHL28 | 1.057947349 | | 2.168505724 | -1.759865996 | |
| 1364 | CLDN4 | 1.057947349 | | 1.750279947 | -1.168505724 | |
| 83862 | TMEM120A | 1.044199804 | | 1.298285253 | -1.039931472 | |
| 3433 | IFIT2 | 1.043902822 | | 2.281231514 | -1.69945729 | |
| 64856 | VWA1 | 1.038462518 | | 1.807182804 | -1.249789854 | |
| 254102 | EHBP1L1 | 1.031006057 | | 2.089981367 | -1.73776465 | |
| 10810 | WASF3 | 1.029573115 | | 1.078777113 | -1.081724397 | |
| 5155 | PDGFB | 1.023829236 | | 1.303403318 | -1.606657572 | |
| 57835 | SLC4A5 | 1.022900402 | | 1.381354373 | -1.584962501 | |
| 115 | ADCY9 | 1.019085841 | | 1.262000586 | -1.389411692 | |
| 4133 | MAP2 | 1.018689073 | | 1.31749461 | -1.145112436 | |
| 147686 | ZNF418 | 1.01282404 | | 6.820178962 | -1.572251449 | |
| 4909 | NTF4 | 1.01282404 | | 6.820178962 | -1.01282404 | |
| 619190 | FDPSL2A | 1.01282404 | | 6.820178962 | -1.572251449 | |
| 2668 | GDNF | 1.01282404 | | 1.572251449 | -1.572251449 | |
| 2784 | GNB3 | 1.01282404 | | 6.820178962 | -6.820178962 | |
| 100272228 | LOC100272228 | 1.01282404 | | 1.572251449 | -6.820178962 | |
| 64150 | DIO3OS | 1.01282404 | | 6.820178962 | -6.820178962 | |
| 100131187 | TSTD1 | 1.01282404 | | 6.820178962 | -1.572251449 | |
| 6515 | SLC2A3 | 1.01282404 | | 6.820178962 | -6.820178962 | |
| 10957 | PNRC1 | 1.012203123 | | 1.425269919 | -1.307543761 | |
| 3623 | INHA | 1.009586049 | | 1.990477226 | -7.238404739 | |
| 148979 | GLIS1 | 1.009586049 | | 1.990477226 | -7.238404739 | |
| 100130275 | LOC100130275 | 1.009586049 | | 1.405514725 | -1.009586049 | |
| 54753 | ZNF853 | 1.009586049 | | 1.405514725 | -1.431049817 | |
| 100499227 | LOC100499227 | 1.009586049 | | 1.405514725 | -1.431049817 | |
| 25946 | ZNF385A | 1.005495988 | | 2.206028975 | -1.484330138 | |
| 10870 | HCST | 1.004800986 | | 1.679030825 | -1.189225557 | |
| 60412 | EXOC4 | 1.004800986 | | 1.242097831 | -1.540132719 | |
| 26160 | IFT172 | 1.004800986 | | 2.004800986 | -2.985692163 | |
| 7091 | TLE4 | 1.004262027 | | 1.166736724 | -1.004262027 | |
| 8784 | TNFRSF18 | 1.002132587 | | 2.848423172 | -1.002132587 | |
| 29108 | PYCARD | -8.813781191 | | -10.25974326 | 9.233619677 | |
| 11249 | NXPH2 | -7.930737338 | | -7.820178962 | 7.554588852 | |
| 1415 | CRYBB2 | -7.813781191 | | -6.22881869 | 5.807354922 | |
| 7562 | ZNF708 | -7.813781191 | | -7.409390936 | 7.22881869 | |
| 348180 | CTU2 | -7.686500527 | | -7.044394119 | 7.554588852 | |
| 2707 | GJB3 | -7.686500527 | | -7.238404739 | 5.807354922 | |
| 154 | ADRB2 | -7.554588852 | | -5.247927513 | 7.22881869 | |
| 79631 | EFTUD1 | -7.400879436 | | -6.820178962 | 8.321928095 | |
| 7056 | THBD | -7.400879436 | | -5.247927513 | 6.554588852 | |
| 269 | AMHR2 | -7.22881869 | | -6.22881869 | 5.807354922 | |
| 399687 | MYO18A | -7.033423002 | | -7.562242424 | 5.247927513 | |
| 201562 | PTPLB | -7.033423002 | | -6.820178962 | 6.820178962 | |
| 374383 | NCR3LG1 | -7.033423002 | | -5.832890014 | 7.820178962 | |
| 161835 | FSIP1 | -7.033423002 | | -5.832890014 | 5.807354922 | |
| 283208 | P4HA3 | -7.033423002 | | -5.247927513 | 5.247927513 | |
| 26272 | FBXO4 | -7.033423002 | | -5.247927513 | 5.247927513 | |
| 4360 | MRC1 | -6.820178962 | | -6.22881869 | 6.22881869 | |
| 440138 | ALG11 | -6.820178962 | | -6.22881869 | 5.807354922 | |
| 51729 | WBP11 | -6.820178962 | | -7.820178962 | 7.693486957 | |
| 348926 | FAM86EP | -6.554588852 | | -7.693486957 | 7.22881869 | |
| 3670 | ISL1 | -6.554588852 | | -5.247927513 | 5.247927513 | |
| 1844 | DUSP2 | -6.554588852 | | -7.238404739 | 6.554588852 | |
| 165545 | DQX1 | -6.554588852 | | -6.22881869 | 6.22881869 | |
| 142913 | CFL1P1 | -6.554588852 | | -5.247927513 | 5.247927513 | |
| 4163 | MCC | -6.554588852 | | -7.693486957 | 7.22881869 | |
| 10389 | SCML2 | -6.554588852 | | -5.247927513 | 5.247927513 | |
| 9609 | RAB36 | -6.554588852 | | -5.247927513 | 6.22881869 | |
| 4901 | NRL | -6.554588852 | | -6.22881869 | 5.807354922 | |
| 84083 | ZRANB3 | -6.554588852 | | -5.247927513 | 5.247927513 | |
| 220972 | 8-Mar | -6.554588852 | | -5.832890014 | 5.247927513 | |
| 6302 | TSPAN31 | -6.22881869 | | -5.832890014 | 5.247927513 | |
| 1846 | DUSP4 | -6.22881869 | | -6.22881869 | 5.247927513 | |
| 27350 | APOBEC3C | -6.22881869 | | -5.247927513 | 6.22881869 | |
| 152485 | ZNF827 | -6.22881869 | | -5.832890014 | 5.247927513 | |
| 57623 | ZFAT | -6.22881869 | | -6.22881869 | 5.247927513 | |
| 116211 | TM4SF19 | -6.22881869 | | -6.22881869 | 5.247927513 | |
| 56413 | LTB4R2 | -6.22881869 | | -6.22881869 | 7.400879436 | |
| 8490 | RGS5 | -6.22881869 | | -7.238404739 | 7.044394119 | |
| 84631 | SLITRK2 | -6.22881869 | | -6.22881869 | 5.247927513 | |
| 85359 | DGCR6L | -6.22881869 | | -5.832890014 | 5.247927513 | |
| 55057 | AIM1L | -6.22881869 | | -6.554588852 | 6.820178962 | |
| 1517 | CTSL1P2 | -5.807354922 | | -7.238404739 | 5.807354922 | |
| 56126 | PCDHB10 | -5.807354922 | | -5.247927513 | 5.807354922 | |
| 7768 | ZNF225 | -5.807354922 | | -6.22881869 | 5.807354922 | |
| 5029 | P2RY2 | -5.807354922 | | -6.22881869 | 6.22881869 | |
| 9047 | SH2D2A | -5.807354922 | | -5.247927513 | 5.247927513 | |
| 2637 | GBX2 | -5.807354922 | | -5.247927513 | 7.22881869 | |
| 6795 | AURKC | -5.807354922 | | -5.247927513 | 5.807354922 | |
| 90809 | TMEM55B | -5.807354922 | | -7.044394119 | 6.22881869 | |
| 100527978 | TMEM56-RWDD3 | -5.807354922 | | -7.562242424 | 7.044394119 | |
| 2628 | GATM | -5.807354922 | | -5.247927513 | 5.807354922 | |
| 7136 | TNNI2 | -5.807354922 | | -5.832890014 | 6.22881869 | |
| 5729 | PTGDR | -5.807354922 | | -7.044394119 | 7.554588852 | |
| 121364 | OR10A7 | -5.807354922 | | -6.554588852 | 6.22881869 | |
| 84517 | ACTRT3 | -5.247927513 | | -8.144658243 | 5.807354922 | |
| 5724 | PTAFR | -5.247927513 | | -5.832890014 | 6.554588852 | |
| 22998 | LIMCH1 | -5.247927513 | | -5.247927513 | 5.247927513 | |
| 349152 | DPY19L2P2 | -5.247927513 | | -6.554588852 | 5.247927513 | |
| 11213 | IRAK3 | -5.247927513 | | -6.22881869 | 7.044394119 | |
| 26005 | C2CD3 | -5.247927513 | | -5.247927513 | 6.22881869 | |
| 5157 | PDGFRL | -5.247927513 | | -6.22881869 | 6.820178962 | |
| 169841 | ZNF169 | -5.247927513 | | -5.832890014 | 6.22881869 | |
| 140686 | WFDC3 | -5.247927513 | | -6.22881869 | 5.247927513 | |
| 2246 | FGF1 | -5.247927513 | | -5.247927513 | 6.22881869 | |
| 3908 | LAMA2 | -5.247927513 | | -5.247927513 | 5.247927513 | |
| 84657 | GHRLOS2 | -5.247927513 | | -6.22881869 | 5.247927513 | |
| 112609 | MRAP2 | -5.247927513 | | -6.22881869 | 5.247927513 | |
| 7099 | TLR4 | -5.247927513 | | -6.22881869 | 6.22881869 | |
| 10861 | SLC26A1 | -5.247927513 | | -5.247927513 | 5.807354922 | |
| 7004 | TEAD4 | -5.247927513 | | -5.247927513 | 5.807354922 | |
| 1368 | CPM | -5.247927513 | | -6.554588852 | 6.554588852 | |
| 347273 | MURC | -5.247927513 | | -6.820178962 | 5.247927513 | |
| 64091 | POPDC2 | -5.247927513 | | -6.22881869 | 5.807354922 | |
| 146434 | ZNF597 | -5.247927513 | | -6.22881869 | 5.807354922 | |
| 84733 | CBX2 | -5.247927513 | | -5.247927513 | 5.247927513 | |
| 283932 | FBXL19-AS1 | -5.247927513 | | -5.832890014 | 5.247927513 | |
| 5314 | PKHD1 | -5.247927513 | | -5.247927513 | 5.247927513 | |
| 80333 | KCNIP4 | -5.247927513 | | -5.832890014 | 5.247927513 | |
| 3887 | KRT81 | -5.247927513 | | -6.820178962 | 6.820178962 | |
| 83856 | FSD1L | -5.247927513 | | -7.238404739 | 7.554588852 | |
| 8360 | HIST1H4D | -5.247927513 | | -5.247927513 | 5.247927513 | |
| 3601 | IL15RA | -3.0694851 | | -1.306661338 | 1.306661338 | |
| 9711 | KIAA0226 | -3.0694851 | | -2.161463423 | 1.796466606 | |
| 27063 | ANKRD1 | -2.94760731 | | -2.198120447 | 2.266393418 | |
| 6650 | SOLH | -2.663970375 | | -1.906253504 | 2.693810013 | |
| 9329 | GTF3C4 | -2.584962501 | | -1.707819249 | 1.464668267 | |
| 53354 | PANK1 | -2.481584761 | | -2.142378675 | 2.434095835 | |
| 7771 | ZFP112 | -2.327071398 | | -1.602036014 | 2.01282404 | |
| 100131454 | DBIL5P | -2.306661338 | | -1.990477226 | 1.980891177 | |
| 22809 | ATF5 | -2.279697888 | | -1.343471582 | 1.432111013 | |
| 9469 | CHST3 | -2.24691474 | | -1.915839552 | 2.004800986 | |
| 26145 | IRF2BP1 | -2.231564067 | | -2.337303321 | 3.069162025 | |
| 55765 | C1orf106 | -2.152951923 | | -1.796466606 | 1.796466606 | |
| 84314 | TMEM107 | -2.152951923 | | -2.688710426 | 2.306661338 | |
| 100303728 | SLC25A5-AS1 | -2.152951923 | | -3.513623719 | 3.231852751 | |
| 152078 | C3orf55 | -1.980891177 | | -1.796466606 | 1.306661338 | |
| 158747 | MOSPD2 | -1.980891177 | | -1.572251449 | 1.306661338 | |
| 283417 | DPY19L2 | -1.980891177 | | -1.306661338 | 1.306661338 | |
| 6273 | S100A2 | -1.973394643 | | -1.557543212 | 1.284049711 | |
| 267004 | PGBD3 | -1.921144579 | | -1.382049087 | 1.484330138 | |
| 306 | ANXA3 | -1.879145605 | | -1.237039197 | 1.593524514 | |
| 5599 | MAPK8 | -1.879145605 | | -1.602036014 | 2.123382416 | |
| 54826 | GIN1 | -1.846290585 | | -1.382049087 | 1.376148486 | |
| 65083 | NOL6 | -1.829115441 | | -1.682860144 | 1.864107098 | |
| 375444 | C5orf34 | -1.822212881 | | -1.068064275 | 1.123496666 | |
| 4312 | MMP1 | -1.810100299 | | -3.562344198 | 2.810100299 | |
| 790955 | C11orf83 | -1.701918647 | | -1.591360272 | 2.172060746 | |
| 114609 | TIRAP | -1.692332598 | | -1.523146493 | 1.083523356 | |
| 133584 | EGFLAM | -1.617505874 | | -1.842194971 | 1.747350638 | |
| 644961 | LOC644961 | -1.593524514 | | -1.237039197 | 1.237039197 | |
| 8320 | EOMES | -1.572251449 | | -2.688710426 | 1.980891177 | |
| 285352 | FLJ39534 | -1.572251449 | | -1.306661338 | 1.306661338 | |
| 3398 | ID2 | -1.559199405 | | -2.868071347 | 1.412241965 | |
| 121053 | C12orf45 | -1.53287399 | | -1.003831862 | 1.138898106 | |
| 205717 | KIAA2018 | -1.511827796 | | -1.368348775 | 1.584962501 | |
| 64897 | C12orf43 | -1.497233651 | | -2.224215157 | 1.31937239 | |
| 54619 | CCNJ | -1.484330138 | | -1.590069391 | 1.376148486 | |
| 7057 | THBS1 | -1.482958102 | | -1.236574037 | 1.350798153 | |
| 131034 | CPNE4 | -1.472564676 | | -3.006388328 | 1.078900828 | |
| 90025 | UBE3D | -1.457681837 | | -1.464668267 | 1.325770161 | |
| 126295 | ZNF57 | -1.457681837 | | -1.707819249 | 2.172060746 | |
| 284098 | PIGW | -1.457681837 | | -1.464668267 | 2.250961574 | |
| 9125 | RQCD1 | -1.457681837 | | -2.650764559 | 1.701918647 | |
| 1102 | RCBTB2 | -1.447288436 | | -1.741562608 | 1.484330138 | |
| 27304 | MOCS3 | -1.376148486 | | -1.382049087 | 1.434095835 | |
| 57082 | CASC5 | -1.359824703 | | -1.105739254 | 1.237205416 | |
| 9507 | ADAMTS4 | -1.356485317 | | -1.652573407 | 1.356485317 | |
| 2730 | GCLM | -1.333313102 | | -1.028979067 | 1.194038313 | |
| 92 | ACVR2A | -1.325770161 | | -2.097610797 | 1.701918647 | |
| 221079 | ARL5B | -1.318855261 | | -1.382049087 | 1.071119991 | |
| 166793 | ZBTB49 | -1.314247358 | | -1.809177658 | 1.501749132 | |
| 467 | ATF3 | -1.306661338 | | -1.572251449 | 2.682809824 | |
| 378708 | APITD1 | -1.305028494 | | -4.169722076 | 2.476923046 | |
| 92935 | MARS2 | -1.282080162 | | -1.132702537 | 1.128324097 | |
| 631 | BFSP1 | -1.25919234 | | -1.007653573 | 1.376148486 | |
| 54964 | C1orf56 | -1.218740027 | | -1.116458977 | 1.110558375 | |
| 84292 | WDR83 | -1.184424571 | | -2.402689107 | 1.944290567 | |
| 150290 | DUSP18 | -1.162474697 | | -1.584962501 | 1.083523356 | |
| 83448 | PUS7L | -1.142271555 | | -1.18377784 | 1.244733589 | |
| 167555 | FAM151B | -1.110558375 | | -1.663636815 | 1.501749132 | |
| 25939 | SAMHD1 | -1.098072123 | | -1.720969194 | 1.619292493 | |
| 6875 | TAF4B | -1.090032201 | | -1.652573407 | 1.189225557 | |

**Table S4. List of 1214 DEGs for NAV^△^ *vs* Y.**

| **Gene ID** | **Symbol** | **log_2_Ratio (Y vs BC)** | **log_2_Ratio (Y vs NC)** | **log_2_Ratio (NAV^△^ vs Y)** |
| --- | --- | --- | --- | --- |
| 7089 | TLE2 | 9.361943774 | 2.317549654 | -2.317549654 |
| 90993 | CREB3L1 | 9.361943774 | 4.11401626 | -2.807354922 |
| 94015 | TTYH2 | 9.361943774 | 1.668456816 | -1.040015679 |
| 147138 | TMC8 | 9.139551352 | 2.31937239 | -1.577308928 |
| 4035 | LRP1 | 9.139551352 | 3.306661338 | -9.139551352 |
| 1944 | EFNA3 | 8.933690655 | 1.524299719 | -1.889296536 |
| 10023 | FRAT1 | 8.820178962 | 2.265590111 | -1.257936538 |
| 7464 | CORO2A | 8.820178962 | 2.591360272 | -8.820178962 |
| 79783 | C7orf10 | 8.758223215 | 1.519818475 | -1.713829095 |
| 11247 | NXPH4 | 8.758223215 | 8.758223215 | -8.758223215 |
| 11045 | UPK1A | 8.554588852 | 2 | -8.554588852 |
| 10382 | TUBB4A | 8.479780264 | 8.479780264 | -8.479780264 |
| 80022 | MYO15B | 8.479780264 | 8.479780264 | -3.231852751 |
| 165679 | SPTSSB | 8.405141463 | 2.176322773 | -2.176322773 |
| 93233 | CCDC114 | 8.405141463 | 2.572251449 | -2.572251449 |
| 64798 | DEPTOR | 8.405141463 | 8.405141463 | -8.405141463 |
| 113612 | CYP2U1 | 8.405141463 | 2.176322773 | -8.405141463 |
| 83715 | ESPN | 8.321928095 | 2.489038081 | -2.093109404 |
| 50649 | ARHGEF4 | 8.321928095 | 1.501749132 | -2.093109404 |
| 25759 | SHC2 | 8.321928095 | 8.321928095 | -8.321928095 |
| 4055 | LTBR | 8.321928095 | 2.489038081 | -8.321928095 |
| 2788 | GNG7 | 8.233619677 | 2.004800986 | -8.233619677 |
| 84152 | PPP1R1B | 8.233619677 | 8.233619677 | -8.233619677 |
| 6820 | SULT2B1 | 8.233619677 | 2.985692163 | -8.233619677 |
| 388886 | FAM211B | 8.139551352 | 1.31937239 | -1.31937239 |
| 647024 | C6orf132 | 8.038918989 | 2.790991476 | -1.484330138 |
| 4854 | NOTCH3 | 8.038918989 | 8.038918989 | -8.038918989 |
| 54933 | RHBDL2 | 8.038918989 | 2.790991476 | -2.790991476 |
| 64284 | RAB17 | 7.936637939 | 2.688710426 | -2.688710426 |
| 84812 | PLCD4 | 7.936637939 | 1.707819249 | -7.936637939 |
| 285908 | LINC00174 | 7.820178962 | 1.265590111 | -1.987288948 |
| 51760 | SYT17 | 7.820178962 | 7.820178962 | -7.820178962 |
| 57168 | ASPHD2 | 7.820178962 | 2.572251449 | -1.591360272 |
| 4093 | SMAD9 | 7.820178962 | 1.987288948 | -2.572251449 |
| 4868 | NPHS1 | 7.820178962 | 1.265590111 | -7.820178962 |
| 5630 | PRPH | 7.693486957 | 7.693486957 | -1.860596943 |
| 2563 | GABRD | 7.693486957 | 1.860596943 | -1.860596943 |
| 7592 | ZNF41 | 7.693486957 | 1.464668267 | -7.693486957 |
| 1066 | CES1 | 7.693486957 | 1.138898106 | -1.860596943 |
| 144132 | DNHD1 | 7.693486957 | 1.464668267 | -2.445559444 |
| 151473 | SLC16A14 | 7.693486957 | 1.464668267 | -2.445559444 |
| 9625 | AATK | 7.693486957 | 7.693486957 | -7.693486957 |
| 56521 | DNAJC12 | 7.693486957 | 1.138898106 | -2.445559444 |
| 23416 | KCNH3 | 7.554588852 | 7.554588852 | -7.554588852 |
| 400242 | DICER1-AS1 | 7.554588852 | 2.306661338 | -7.554588852 |
| 222962 | SLC29A4 | 7.554588852 | 7.554588852 | -2.306661338 |
| 6364 | CCL20 | 7.554588852 | 1.721698838 | -7.554588852 |
| 643641 | ZNF862 | 7.554588852 | 7.554588852 | -7.554588852 |
| 9746 | CLSTN3 | 7.554588852 | 1.721698838 | -7.554588852 |
| 10384 | BTN3A3 | 7.554588852 | 2.306661338 | -7.554588852 |
| 5662 | PSD | 7.554588852 | 7.554588852 | -1.721698838 |
| 8497 | PPFIA4 | 7.554588852 | 7.554588852 | -7.554588852 |
| 9966 | TNFSF15 | 7.554588852 | 1.325770161 | -7.554588852 |
| 284069 | FAM171A2 | 7.400879436 | 7.400879436 | -7.400879436 |
| 79148 | MMP28 | 7.400879436 | 7.400879436 | -2.152951923 |
| 139728 | PNCK | 7.400879436 | 7.400879436 | -7.400879436 |
| 3429 | IFI27 | 7.400879436 | 1.172060746 | -2.152951923 |
| 3201 | HOXA4 | 7.400879436 | 2.152951923 | -1.172060746 |
| 139105 | BEND2 | 7.400879436 | 1.172060746 | -2.152951923 |
| 51279 | C1RL | 7.400879436 | 1.567989422 | -1.567989422 |
| 79603 | CERS4 | 7.400879436 | 1.172060746 | -1.567989422 |
| 100129518 | LOC100129518 | 7.400879436 | 1.567989422 | -7.400879436 |
| 4760 | NEUROD1 | 7.400879436 | 1.172060746 | -7.400879436 |
| 90019 | SYT8 | 7.400879436 | 7.400879436 | -1.567989422 |
| 27151 | CPAMD8 | 7.400879436 | 7.400879436 | -7.400879436 |
| 199699 | DAND5 | 7.400879436 | 1.172060746 | -7.400879436 |
| 9022 | CLIC3 | 7.238404739 | 1.405514725 | -1.405514725 |
| 10235 | RASGRP2 | 7.238404739 | 7.238404739 | -7.238404739 |
| 9056 | SLC7A7 | 7.238404739 | 7.238404739 | -7.238404739 |
| 5333 | PLCD1 | 7.238404739 | 1.009586049 | -7.238404739 |
| 26249 | KLHL3 | 7.238404739 | 1.405514725 | -1.405514725 |
| 9473 | C1orf38 | 7.238404739 | 1.009586049 | -7.238404739 |
| 1298 | COL9A2 | 7.238404739 | 7.238404739 | -7.238404739 |
| 3934 | LCN2 | 7.238404739 | 7.238404739 | -7.238404739 |
| 5740 | PTGIS | 7.238404739 | 7.238404739 | -7.238404739 |
| 84440 | RAB11FIP4 | 7.238404739 | 7.238404739 | -1.009586049 |
| 10316 | NMUR1 | 7.238404739 | 7.238404739 | -7.238404739 |
| 146771 | TCAM1P | 7.238404739 | 1.990477226 | -1.990477226 |
| 594842 | HAS2-AS1 | 7.238404739 | 1.405514725 | -1.009586049 |
| 100506046 | LOC100506046 | 7.238404739 | 7.238404739 | -1.990477226 |
| 259217 | HSPA12A | 7.238404739 | 7.238404739 | -7.238404739 |
| 83999 | KREMEN1 | 7.238404739 | 7.238404739 | -7.238404739 |
| 388152 | LOC388152 | 7.238404739 | 7.238404739 | -7.238404739 |
| 4902 | NRTN | 7.044394119 | 7.044394119 | -7.044394119 |
| 79611 | ACSS3 | 7.044394119 | 7.044394119 | -7.044394119 |
| 344148 | NCKAP5 | 7.044394119 | 7.044394119 | -1.796466606 |
| 105 | ADARB2 | 7.044394119 | 7.044394119 | -1.796466606 |
| 3108 | HLA-DMA | 7.044394119 | 7.044394119 | -7.044394119 |
| 284161 | GDPD1 | 7.044394119 | 7.044394119 | -7.044394119 |
| 338328 | GPIHBP1 | 7.044394119 | 1.211504105 | -1.211504105 |
| 4059 | BCAM | 7.044394119 | 7.044394119 | -7.044394119 |
| 390664 | C1QTNF8 | 7.044394119 | 1.211504105 | -1.211504105 |
| 634 | CEACAM1 | 7.044394119 | 1.796466606 | -7.044394119 |
| 10076 | PTPRU | 7.044394119 | 1.796466606 | -7.044394119 |
| 158046 | NXNL2 | 7.044394119 | 7.044394119 | -7.044394119 |
| 50619 | DEF6 | 7.044394119 | 7.044394119 | -7.044394119 |
| 126129 | CPT1C | 7.044394119 | 7.044394119 | -7.044394119 |
| 6712 | SPTBN2 | 6.820178962 | 6.820178962 | -1.572251449 |
| 11092 | C9orf9 | 6.820178962 | 1.572251449 | -6.820178962 |
| 9481 | SLC25A27 | 6.820178962 | 6.820178962 | -1.572251449 |
| 100422970 | MIR1273D | 6.820178962 | 6.820178962 | -1.572251449 |
| 10966 | RAB40B | 6.820178962 | 1.572251449 | -6.820178962 |
| 768 | CA9 | 6.820178962 | 6.820178962 | -6.820178962 |
| 54510 | PCDH18 | 6.820178962 | 6.820178962 | -6.820178962 |
| 55876 | GSDMB | 6.820178962 | 6.820178962 | -6.820178962 |
| 29935 | RPA4 | 6.820178962 | 6.820178962 | -1.572251449 |
| 60468 | BACH2 | 6.820178962 | 6.820178962 | -6.820178962 |
| 221806 | VWDE | 6.820178962 | 1.572251449 | -1.572251449 |
| 7379 | UPK2 | 6.820178962 | 6.820178962 | -1.572251449 |
| 440356 | LOC440356 | 6.820178962 | 6.820178962 | -1.572251449 |
| 90525 | SHF | 6.820178962 | 6.820178962 | -6.820178962 |
| 79750 | ZNF385D | 6.820178962 | 6.820178962 | -6.820178962 |
| 89846 | FGD3 | 6.820178962 | 1.572251449 | -6.820178962 |
| 283551 | C14orf182 | 6.820178962 | 6.820178962 | -1.572251449 |
| 9363 | RAB33A | 6.554588852 | 6.554588852 | -6.554588852 |
| 5327 | PLAT | 6.554588852 | 6.554588852 | -1.306661338 |
| 5313 | PKLR | 6.554588852 | 6.554588852 | -6.554588852 |
| 114897 | C1QTNF1 | 6.554588852 | 6.554588852 | -6.554588852 |
| 1815 | DRD4 | 6.554588852 | 6.554588852 | -6.554588852 |
| 2256 | FGF11 | 6.554588852 | 6.554588852 | -1.306661338 |
| 7103 | TSPAN8 | 6.554588852 | 6.554588852 | -6.554588852 |
| 112476 | PRRT2 | 6.554588852 | 1.306661338 | -6.554588852 |
| 25802 | LMOD1 | 6.554588852 | 6.554588852 | -6.554588852 |
| 4137 | MAPT | 6.554588852 | 6.554588852 | -6.554588852 |
| 80725 | SRCIN1 | 6.554588852 | 6.554588852 | -6.554588852 |
| 4642 | MYO1D | 6.554588852 | 6.554588852 | -6.554588852 |
| 201229 | C17orf108 | 6.554588852 | 6.554588852 | -6.554588852 |
| 5657 | PRTN3 | 6.554588852 | 6.554588852 | -6.554588852 |
| 57731 | SPTBN4 | 6.554588852 | 6.554588852 | -6.554588852 |
| 23508 | TTC9 | 6.554588852 | 1.306661338 | -6.554588852 |
| 55512 | SMPD3 | 6.554588852 | 6.554588852 | -6.554588852 |
| 79689 | STEAP4 | 6.554588852 | 6.554588852 | -1.306661338 |
| 122945 | NOXRED1 | 6.554588852 | 6.554588852 | -6.554588852 |
| 728743 | LOC728743 | 6.554588852 | 1.306661338 | -6.554588852 |
| 401106 | FLJ34208 | 6.554588852 | 6.554588852 | -6.554588852 |
| 57572 | DOCK6 | 6.554588852 | 6.554588852 | -6.554588852 |
| 54518 | APBB1IP | 6.554588852 | 6.554588852 | -6.554588852 |
| 1756 | DMD | 6.554588852 | 6.554588852 | -6.554588852 |
| 386597 | LOC386597 | 6.554588852 | 6.554588852 | -6.554588852 |
| 2957 | GTF2A1 | 6.554588852 | 6.554588852 | -1.306661338 |
| 57047 | PLSCR2 | 6.554588852 | 6.554588852 | -6.554588852 |
| 80115 | BAIAP2L2 | 6.554588852 | 6.554588852 | -6.554588852 |
| 145645 | C15orf43 | 6.554588852 | 6.554588852 | -6.554588852 |
| 79369 | B3GNT4 | 6.554588852 | 6.554588852 | -6.554588852 |
| 162466 | PHOSPHO1 | 6.554588852 | 1.306661338 | -6.554588852 |
| 113763 | C7orf29 | 6.554588852 | 6.554588852 | -6.554588852 |
| 284677 | C1orf204 | 6.554588852 | 6.554588852 | -6.554588852 |
| 7773 | ZNF230 | 6.554588852 | 6.554588852 | -6.554588852 |
| 100128569 | C19orf71 | 6.554588852 | 6.554588852 | -6.554588852 |
| 79883 | PODNL1 | 6.554588852 | 6.554588852 | -6.554588852 |
| 644596 | LINC00087 | 6.554588852 | 6.554588852 | -1.306661338 |
| 10637 | LEFTY1 | 6.554588852 | 1.306661338 | -6.554588852 |
| 79805 | VASH2 | 6.22881869 | 6.22881869 | -6.22881869 |
| 56971 | CEACAM19 | 6.22881869 | 6.22881869 | -6.22881869 |
| 200844 | C3orf67 | 6.22881869 | 6.22881869 | -6.22881869 |
| 100192386 | FLJ16779 | 6.22881869 | 6.22881869 | -6.22881869 |
| 100302254 | MIR1282 | 6.22881869 | 6.22881869 | -6.22881869 |
| 7869 | SEMA3B | 6.22881869 | 6.22881869 | -6.22881869 |
| 8708 | B3GALT1 | 6.22881869 | 6.22881869 | -6.22881869 |
| 100507266 | LOC100507266 | 6.22881869 | 6.22881869 | -6.22881869 |
| 135932 | TMEM139 | 6.22881869 | 6.22881869 | -6.22881869 |
| 145788 | FLJ27352 | 6.22881869 | 6.22881869 | -6.22881869 |
| 83982 | IFI27L2 | 6.22881869 | 6.22881869 | -6.22881869 |
| 57616 | TSHZ3 | 6.22881869 | 6.22881869 | -6.22881869 |
| 8626 | TP63 | 6.22881869 | 6.22881869 | -6.22881869 |
| 55561 | CDC42BPG | 6.22881869 | 6.22881869 | -6.22881869 |
| 9381 | OTOF | 6.22881869 | 6.22881869 | -6.22881869 |
| 6861 | SYT5 | 6.22881869 | 6.22881869 | -6.22881869 |
| 2737 | GLI3 | 6.22881869 | 6.22881869 | -6.22881869 |
| 56606 | SLC2A9 | 6.22881869 | 6.22881869 | -6.22881869 |
| 83538 | TTC25 | 6.22881869 | 6.22881869 | -6.22881869 |
| 283914 | LOC283914 | 6.22881869 | 6.22881869 | -6.22881869 |
| 8630 | HSD17B6 | 6.22881869 | 6.22881869 | -6.22881869 |
| 129804 | FBLN7 | 6.22881869 | 6.22881869 | -6.22881869 |
| 9900 | SV2A | 6.22881869 | 6.22881869 | -6.22881869 |
| 282973 | JAKMIP3 | 6.22881869 | 6.22881869 | -6.22881869 |
| 595099 | SNORD18B | 6.22881869 | 6.22881869 | -6.22881869 |
| 1576 | CYP3A4 | 6.22881869 | 6.22881869 | -6.22881869 |
| 28514 | DLL1 | 6.22881869 | 6.22881869 | -6.22881869 |
| 51334 | PRR16 | 6.22881869 | 6.22881869 | -6.22881869 |
| 57088 | PLSCR4 | 6.22881869 | 6.22881869 | -6.22881869 |
| 100130519 | TMEM221 | 6.22881869 | 6.22881869 | -6.22881869 |
| 2661 | GDF9 | 6.22881869 | 6.22881869 | -6.22881869 |
| 3811 | KIR3DL1 | 6.22881869 | 6.22881869 | -6.22881869 |
| 84699 | CREB3L3 | 6.22881869 | 6.22881869 | -6.22881869 |
| 79955 | PDZD7 | 6.22881869 | 6.22881869 | -6.22881869 |
| 26102 | DKFZP434A062 | 6.22881869 | 6.22881869 | -6.22881869 |
| 56967 | C14orf132 | 6.22881869 | 6.22881869 | -6.22881869 |
| 389337 | ARHGEF37 | 6.22881869 | 6.22881869 | -6.22881869 |
| 349667 | RTN4RL2 | 6.22881869 | 6.22881869 | -6.22881869 |
| 162962 | ZNF836 | 6.22881869 | 6.22881869 | -6.22881869 |
| 130733 | TMEM178 | 6.22881869 | 6.22881869 | -6.22881869 |
| 7078 | TIMP3 | 6.22881869 | 6.22881869 | -6.22881869 |
| 23566 | LPAR3 | 6.22881869 | 6.22881869 | -6.22881869 |
| 221481 | ARMC12 | 6.22881869 | 6.22881869 | -6.22881869 |
| 203102 | ADAM32 | 6.22881869 | 6.22881869 | -6.22881869 |
| 85301 | COL27A1 | 6.22881869 | 6.22881869 | -6.22881869 |
| 54502 | RBM47 | 6.22881869 | 6.22881869 | -6.22881869 |
| 57664 | PLEKHA4 | 6.22881869 | 6.22881869 | -6.22881869 |
| 120071 | GYLTL1B | 6.22881869 | 6.22881869 | -6.22881869 |
| 4211 | MEIS1 | 6.22881869 | 6.22881869 | -6.22881869 |
| 54413 | NLGN3 | 6.22881869 | 6.22881869 | -6.22881869 |
| 51147 | ING4 | 5.807354922 | 5.807354922 | -5.807354922 |
| 5922 | RASA2 | 5.807354922 | 5.807354922 | -5.807354922 |
| 3563 | IL3RA | 5.807354922 | 5.807354922 | -5.807354922 |
| 3780 | KCNN1 | 5.807354922 | 5.807354922 | -5.807354922 |
| 3208 | HPCA | 5.807354922 | 5.807354922 | -5.807354922 |
| 4610 | MYCL1 | 5.807354922 | 5.807354922 | -5.807354922 |
| 440299 | DNM1P41 | 5.807354922 | 5.807354922 | -5.807354922 |
| 8736 | MYOM1 | 5.807354922 | 5.807354922 | -5.807354922 |
| 145200 | LINC00239 | 5.807354922 | 5.807354922 | -5.807354922 |
| 51285 | RASL12 | 5.807354922 | 5.807354922 | -5.807354922 |
| 100128124 | HGC6.3 | 5.807354922 | 5.807354922 | -5.807354922 |
| 27019 | DNAI1 | 5.807354922 | 5.807354922 | -5.807354922 |
| 440896 | LOC440896 | 5.807354922 | 5.807354922 | -5.807354922 |
| 728118 | FAM22A | 5.807354922 | 5.807354922 | -5.807354922 |
| 401136 | TMPRSS11BNL | 5.807354922 | 5.807354922 | -5.807354922 |
| 128344 | C1orf88 | 5.807354922 | 5.807354922 | -5.807354922 |
| 2894 | GRID1 | 5.807354922 | 5.807354922 | -5.807354922 |
| 972 | CD74 | 5.807354922 | 5.807354922 | -5.807354922 |
| 80258 | EFHC2 | 5.807354922 | 5.807354922 | -5.807354922 |
| 285973 | ATG9B | 5.807354922 | 5.807354922 | -5.807354922 |
| 3777 | KCNK3 | 5.807354922 | 5.807354922 | -5.807354922 |
| 6297 | SALL2 | 5.807354922 | 5.807354922 | -5.807354922 |
| 374407 | DNAJB13 | 5.807354922 | 5.807354922 | -5.807354922 |
| 80975 | TMPRSS5 | 5.807354922 | 5.807354922 | -5.807354922 |
| 54734 | RAB39A | 5.807354922 | 5.807354922 | -5.807354922 |
| 6288 | SAA1 | 5.807354922 | 5.807354922 | -5.807354922 |
| 6425 | SFRP5 | 5.807354922 | 5.807354922 | -5.807354922 |
| 55065 | SLC52A1 | 5.807354922 | 5.807354922 | -5.807354922 |
| 255101 | CCDC108 | 5.807354922 | 5.807354922 | -5.807354922 |
| 81931 | ZNF93 | 5.807354922 | 5.807354922 | -5.807354922 |
| 79258 | MMEL1 | 5.807354922 | 5.807354922 | -5.807354922 |
| 149465 | WDR65 | 5.807354922 | 5.807354922 | -5.807354922 |
| 83401 | ELOVL3 | 5.807354922 | 5.807354922 | -5.807354922 |
| 5733 | PTGER3 | 5.807354922 | 5.807354922 | -5.807354922 |
| 79690 | GAL3ST4 | 5.807354922 | 5.807354922 | -5.807354922 |
| 4914 | NTRK1 | 5.807354922 | 5.807354922 | -5.807354922 |
| 387597 | ILDR2 | 5.807354922 | 5.807354922 | -5.807354922 |
| 648791 | PPP1R3G | 5.807354922 | 5.807354922 | -5.807354922 |
| 26231 | LRRC29 | 5.807354922 | 5.807354922 | -5.807354922 |
| 195814 | SDR16C5 | 5.807354922 | 5.807354922 | -5.807354922 |
| 1991 | ELANE | 5.807354922 | 5.807354922 | -5.807354922 |
| 326342 | EMR4P | 5.807354922 | 5.807354922 | -5.807354922 |
| 3592 | IL12A | 5.807354922 | 5.807354922 | -5.807354922 |
| 10044 | SH2D3C | 5.807354922 | 5.807354922 | -5.807354922 |
| 441454 | LOC441454 | 5.807354922 | 5.807354922 | -5.807354922 |
| 9914 | ATP2C2 | 5.807354922 | 5.807354922 | -5.807354922 |
| 118663 | BTBD16 | 5.807354922 | 5.807354922 | -5.807354922 |
| 644242 | LOC644242 | 5.807354922 | 5.807354922 | -5.807354922 |
| 8608 | RDH16 | 5.807354922 | 5.807354922 | -5.807354922 |
| 4311 | MME | 5.807354922 | 5.807354922 | -5.807354922 |
| 2122 | MECOM | 5.807354922 | 5.807354922 | -5.807354922 |
| 79935 | CNTD2 | 5.807354922 | 5.807354922 | -5.807354922 |
| 84750 | FUT10 | 5.807354922 | 5.807354922 | -5.807354922 |
| 81563 | C1orf21 | 5.807354922 | 5.807354922 | -5.807354922 |
| 126969 | SLC44A3 | 5.807354922 | 5.807354922 | -5.807354922 |
| 64097 | EPB41L4A | 5.807354922 | 5.807354922 | -5.807354922 |
| 64386 | MMP25 | 5.807354922 | 5.807354922 | -5.807354922 |
| 642273 | FAM110C | 5.807354922 | 5.807354922 | -5.807354922 |
| 5452 | POU2F2 | 5.807354922 | 5.807354922 | -5.807354922 |
| 170692 | ADAMTS18 | 5.807354922 | 5.807354922 | -5.807354922 |
| 285588 | EFCAB9 | 5.807354922 | 5.807354922 | -5.807354922 |
| 389118 | CDHR4 | 5.807354922 | 5.807354922 | -5.807354922 |
| 168507 | PKD1L1 | 5.807354922 | 5.807354922 | -5.807354922 |
| 27134 | TJP3 | 5.807354922 | 5.807354922 | -5.807354922 |
| 51302 | CYP39A1 | 5.807354922 | 5.807354922 | -5.807354922 |
| 3386 | ICAM4 | 5.807354922 | 5.807354922 | -5.807354922 |
| 440910 | LOC440910 | 5.807354922 | 5.807354922 | -5.807354922 |
| 9892 | SNAP91 | 5.807354922 | 5.807354922 | -5.807354922 |
| 158067 | AK8 | 5.807354922 | 5.807354922 | -5.807354922 |
| 619505 | SNORA21 | 5.807354922 | 5.807354922 | -5.807354922 |
| 146 | ADRA1D | 5.807354922 | 5.807354922 | -5.807354922 |
| 347 | APOD | 5.807354922 | 5.807354922 | -5.807354922 |
| 9514 | GAL3ST1 | 5.807354922 | 5.807354922 | -5.807354922 |
| 56271 | BEX4 | 5.807354922 | 5.807354922 | -5.807354922 |
| 4807 | NHLH1 | 5.807354922 | 5.807354922 | -5.807354922 |
| 285103 | LOC285103 | 5.807354922 | 5.807354922 | -5.807354922 |
| 843 | CASP10 | 5.807354922 | 5.807354922 | -5.807354922 |
| 127579 | DCST2 | 5.807354922 | 5.807354922 | -5.807354922 |
| 79963 | ABCA11P | 5.807354922 | 5.807354922 | -5.807354922 |
| 5602 | MAPK10 | 5.807354922 | 5.807354922 | -5.807354922 |
| 400866 | LINC00114 | 5.807354922 | 5.807354922 | -5.807354922 |
| 100506211 | MIR210HG | 5.492707774 | 3.841943214 | -3.440755694 |
| 54541 | DDIT4 | 5.343594833 | 2.353117607 | -2.547128227 |
| 54845 | ESRP1 | 5.247927513 | 5.247927513 | -5.247927513 |
| 100190940 | LOC100190940 | 5.247927513 | 5.247927513 | -5.247927513 |
| 7087 | ICAM5 | 5.247927513 | 5.247927513 | -5.247927513 |
| 677777 | SCARNA12 | 5.247927513 | 5.247927513 | -5.247927513 |
| 100130311 | C17orf107 | 5.247927513 | 5.247927513 | -5.247927513 |
| 26266 | SLC13A4 | 5.247927513 | 5.247927513 | -5.247927513 |
| 5625 | PRODH | 5.247927513 | 5.247927513 | -5.247927513 |
| 100132215 | LOC100132215 | 5.247927513 | 5.247927513 | -5.247927513 |
| 100506939 | LOC100506939 | 5.247927513 | 5.247927513 | -5.247927513 |
| 84249 | PSD2 | 5.247927513 | 5.247927513 | -5.247927513 |
| 1644 | DDC | 5.247927513 | 5.247927513 | -5.247927513 |
| 23281 | MTUS2 | 5.247927513 | 5.247927513 | -5.247927513 |
| 57167 | SALL4 | 5.247927513 | 5.247927513 | -5.247927513 |
| 100616668 | LOC100616668 | 5.247927513 | 5.247927513 | -5.247927513 |
| 129293 | C2orf89 | 5.247927513 | 5.247927513 | -5.247927513 |
| 197335 | WDR90 | 5.247927513 | 5.247927513 | -5.247927513 |
| 6092 | ROBO2 | 5.247927513 | 5.247927513 | -5.247927513 |
| 128853 | DUSP15 | 5.247927513 | 5.247927513 | -5.247927513 |
| 151835 | CPNE9 | 5.247927513 | 5.247927513 | -5.247927513 |
| 23563 | CHST5 | 5.247927513 | 5.247927513 | -5.247927513 |
| 283796 | GOLGA8IP | 5.247927513 | 5.247927513 | -5.247927513 |
| 51214 | IGF2-AS | 5.247927513 | 5.247927513 | -5.247927513 |
| 375298 | CERKL | 5.247927513 | 5.247927513 | -5.247927513 |
| 4320 | MMP11 | 5.247927513 | 5.247927513 | -5.247927513 |
| 283948 | NHLRC4 | 5.247927513 | 5.247927513 | -5.247927513 |
| 84960 | KIAA1984 | 5.247927513 | 5.247927513 | -5.247927513 |
| 1041 | CDSN | 5.247927513 | 5.247927513 | -5.247927513 |
| 5608 | MAP2K6 | 5.247927513 | 5.247927513 | -5.247927513 |
| 339674 | BK250D10.8 | 5.247927513 | 5.247927513 | -5.247927513 |
| 8743 | TNFSF10 | 5.247927513 | 5.247927513 | -5.247927513 |
| 2073 | ERCC5 | 5.247927513 | 5.247927513 | -5.247927513 |
| 152024 | LOC152024 | 5.247927513 | 5.247927513 | -5.247927513 |
| 692215 | SNORD112 | 5.247927513 | 5.247927513 | -5.247927513 |
| 100233209 | LOC100233209 | 5.247927513 | 5.247927513 | -5.247927513 |
| 9963 | SLC23A1 | 5.247927513 | 5.247927513 | -5.247927513 |
| 6401 | SELE | 5.247927513 | 5.247927513 | -5.247927513 |
| 100422948 | MIR4284 | 5.247927513 | 5.247927513 | -5.247927513 |
| 7018 | TF | 5.247927513 | 5.247927513 | -5.247927513 |
| 151651 | EFHB | 5.247927513 | 5.247927513 | -5.247927513 |
| 100288123 | LOC100288123 | 5.247927513 | 5.247927513 | -5.247927513 |
| 27443 | CECR2 | 5.247927513 | 5.247927513 | -5.247927513 |
| 4803 | NGF | 5.247927513 | 5.247927513 | -5.247927513 |
| 7368 | UGT8 | 5.247927513 | 5.247927513 | -5.247927513 |
| 100128081 | JAZF1-AS1 | 5.247927513 | 5.247927513 | -5.247927513 |
| 117852 | TRIM78P | 5.247927513 | 5.247927513 | -5.247927513 |
| 23231 | SEL1L3 | 5.247927513 | 5.247927513 | -5.247927513 |
| 100507050 | LOC100507050 | 5.247927513 | 5.247927513 | -5.247927513 |
| 7638 | ZNF221 | 5.247927513 | 5.247927513 | -5.247927513 |
| 653140 | C2orf84 | 5.247927513 | 5.247927513 | -5.247927513 |
| 5837 | PYGM | 5.247927513 | 5.247927513 | -5.247927513 |
| 4248 | MGAT3 | 5.247927513 | 5.247927513 | -5.247927513 |
| 29119 | CTNNA3 | 5.247927513 | 5.247927513 | -5.247927513 |
| 388815 | LINC00478 | 5.247927513 | 5.247927513 | -5.247927513 |
| 158405 | KIAA1958 | 5.247927513 | 5.247927513 | -5.247927513 |
| 6542 | SLC7A2 | 5.247927513 | 5.247927513 | -5.247927513 |
| 121551 | BTBD11 | 5.247927513 | 5.247927513 | -5.247927513 |
| 5143 | PDE4C | 5.247927513 | 5.247927513 | -5.247927513 |
| 29993 | PACSIN1 | 5.247927513 | 5.247927513 | -5.247927513 |
| 10901 | DHRS4 | 5.247927513 | 5.247927513 | -5.247927513 |
| 6791 | AURKAPS1 | 5.247927513 | 5.247927513 | -5.247927513 |
| 5738 | PTGFRN | 5.247927513 | 5.247927513 | -5.247927513 |
| 347051 | SLC10A5 | 5.247927513 | 5.247927513 | -5.247927513 |
| 1137 | CHRNA4 | 5.247927513 | 5.247927513 | -5.247927513 |
| 127495 | LRRC39 | 5.247927513 | 5.247927513 | -5.247927513 |
| 56159 | TEX11 | 5.247927513 | 5.247927513 | -5.247927513 |
| 23233 | EXOC6B | 5.247927513 | 5.247927513 | -5.247927513 |
| 2676 | GFRA3 | 5.247927513 | 5.247927513 | -5.247927513 |
| 11320 | MGAT4A | 5.247927513 | 5.247927513 | -5.247927513 |
| 7093 | TLL2 | 5.247927513 | 5.247927513 | -5.247927513 |
| 643965 | TMEM88B | 5.247927513 | 5.247927513 | -5.247927513 |
| 9635 | CLCA2 | 5.247927513 | 5.247927513 | -5.247927513 |
| 285033 | LOC285033 | 5.247927513 | 5.247927513 | -5.247927513 |
| 286336 | FAM78A | 5.247927513 | 5.247927513 | -5.247927513 |
| 284390 | ZNF763 | 5.247927513 | 5.247927513 | -5.247927513 |
| 220001 | VWCE | 5.247927513 | 5.247927513 | -5.247927513 |
| 2560 | GABRB1 | 5.247927513 | 5.247927513 | -5.247927513 |
| 84645 | C22orf23 | 5.247927513 | 5.247927513 | -5.247927513 |
| 163351 | GBP6 | 5.247927513 | 5.247927513 | -5.247927513 |
| 112817 | HOGA1 | 5.247927513 | 5.247927513 | -5.247927513 |
| 100129827 | MRVI1-AS1 | 5.247927513 | 5.247927513 | -5.247927513 |
| 65268 | WNK2 | 5.247927513 | 5.247927513 | -5.247927513 |
| 629 | CFB | 5.247927513 | 5.247927513 | -5.247927513 |
| 8787 | RGS9 | 5.247927513 | 5.247927513 | -5.247927513 |
| 117583 | PARD3B | 5.247927513 | 5.247927513 | -5.247927513 |
| 482 | ATP1B2 | 5.247927513 | 5.247927513 | -5.247927513 |
| 7429 | VIL1 | 5.247927513 | 5.247927513 | -5.247927513 |
| 222389 | BEND7 | 5.247927513 | 5.247927513 | -5.247927513 |
| 218 | ALDH3A1 | 5.247927513 | 5.247927513 | -5.247927513 |
| 773 | CACNA1A | 5.247927513 | 5.247927513 | -5.247927513 |
| 8358 | HIST1H3B | 5.247927513 | 5.247927513 | -5.247927513 |
| 8338 | HIST2H2AC | 5.247927513 | 5.247927513 | -5.247927513 |
| 5603 | MAPK13 | 5.247927513 | 5.247927513 | -5.247927513 |
| 65217 | PCDH15 | 5.247927513 | 5.247927513 | -5.247927513 |
| 100506012 | LOC100506012 | 5.247927513 | 5.247927513 | -5.247927513 |
| 403315 | FAM92A1P2 | 5.247927513 | 5.247927513 | -5.247927513 |
| 100302650 | LOC100302650 | 5.247927513 | 5.247927513 | -5.247927513 |
| 389332 | LOC389332 | 5.247927513 | 5.247927513 | -5.247927513 |
| 5649 | RELN | 5.247927513 | 5.247927513 | -5.247927513 |
| 79623 | GALNT14 | 5.247927513 | 5.247927513 | -5.247927513 |
| 116443 | GRIN3A | 5.247927513 | 5.247927513 | -5.247927513 |
| 90338 | ZNF160 | 5.247927513 | 5.247927513 | -5.247927513 |
| 7075 | TIE1 | 5.247927513 | 5.247927513 | -5.247927513 |
| 3781 | KCNN2 | 5.247927513 | 5.247927513 | -5.247927513 |
| 90187 | EMILIN3 | 5.247927513 | 5.247927513 | -5.247927513 |
| 643719 | SCGB1B2P | 5.247927513 | 5.247927513 | -5.247927513 |
| 119395 | CALHM3 | 5.247927513 | 5.247927513 | -5.247927513 |
| 401494 | PTPLAD2 | 5.247927513 | 5.247927513 | -5.247927513 |
| 4440 | MSI1 | 5.247927513 | 5.247927513 | -5.247927513 |
| 353500 | BMP8A | 5.247927513 | 5.247927513 | -5.247927513 |
| 123099 | DEGS2 | 5.247927513 | 5.247927513 | -5.247927513 |
| 400830 | DEFB132 | 5.247927513 | 5.247927513 | -5.247927513 |
| 282763 | OR51B5 | 5.247927513 | 5.247927513 | -5.247927513 |
| 401081 | FLJ22763 | 5.247927513 | 5.247927513 | -5.247927513 |
| 116535 | MRGPRF | 5.247927513 | 5.247927513 | -5.247927513 |
| 79132 | DHX58 | 5.247927513 | 5.247927513 | -5.247927513 |
| 79411 | GLB1L | 5.247927513 | 5.247927513 | -5.247927513 |
| 100133144 | UBE2Q2P3 | 5.247927513 | 5.247927513 | -5.247927513 |
| 389813 | C9orf172 | 5.247927513 | 5.247927513 | -5.247927513 |
| 27124 | INPP5J | 5.247927513 | 5.247927513 | -5.247927513 |
| 7454 | WAS | 5.247927513 | 5.247927513 | -5.247927513 |
| 81854 | MGC3771 | 5.247927513 | 5.247927513 | -5.247927513 |
| 84734 | FAM167B | 5.247927513 | 5.247927513 | -5.247927513 |
| 1269 | CNR2 | 5.247927513 | 5.247927513 | -5.247927513 |
| 56477 | CCL28 | 5.247927513 | 5.247927513 | -5.247927513 |
| 3248 | HPGD | 5.247927513 | 5.247927513 | -5.247927513 |
| 692085 | SNORD45C | 5.247927513 | 5.247927513 | -5.247927513 |
| 6374 | CXCL5 | 5.247927513 | 5.247927513 | -5.247927513 |
| 22798 | LAMB4 | 5.247927513 | 5.247927513 | -5.247927513 |
| 25876 | SPEF1 | 5.247927513 | 5.247927513 | -5.247927513 |
| 4629 | MYH11 | 5.247927513 | 5.247927513 | -5.247927513 |
| 4015 | LOX | 5.247927513 | 5.247927513 | -5.247927513 |
| 4359 | MPZ | 5.247927513 | 5.247927513 | -5.247927513 |
| 650 | BMP2 | 5.247927513 | 5.247927513 | -5.247927513 |
| 162461 | TMEM92 | 5.247927513 | 5.247927513 | -5.247927513 |
| 554236 | DPY19L2P1 | 5.247927513 | 5.247927513 | -5.247927513 |
| 117286 | CIB3 | 5.247927513 | 5.247927513 | -5.247927513 |
| 7832 | BTG2 | 5.247927513 | 5.247927513 | -5.247927513 |
| 10788 | IQGAP2 | 5.247927513 | 5.247927513 | -5.247927513 |
| 166336 | PRICKLE2 | 5.247927513 | 5.247927513 | -5.247927513 |
| 92359 | CRB3 | 5.247927513 | 5.247927513 | -5.247927513 |
| 116844 | LRG1 | 5.247927513 | 5.247927513 | -5.247927513 |
| 3902 | LAG3 | 5.247927513 | 5.247927513 | -5.247927513 |
| 164668 | APOBEC3H | 5.247927513 | 5.247927513 | -5.247927513 |
| 23148 | NACAD | 5.247927513 | 5.247927513 | -5.247927513 |
| 79844 | ZDHHC11 | 5.247927513 | 5.247927513 | -5.247927513 |
| 362 | AQP5 | 5.247927513 | 5.247927513 | -5.247927513 |
| 286530 | P2RY8 | 5.247927513 | 5.247927513 | -5.247927513 |
| 64881 | PCDH20 | 5.247927513 | 5.247927513 | -5.247927513 |
| 374877 | C19orf45 | 5.247927513 | 5.247927513 | -5.247927513 |
| 79012 | CAMKV | 5.247927513 | 5.247927513 | -5.247927513 |
| 389812 | LCN15 | 5.247927513 | 5.247927513 | -5.247927513 |
| 162494 | RHBDL3 | 5.247927513 | 5.247927513 | -5.247927513 |
| 56127 | PCDHB9 | 5.247927513 | 5.247927513 | -5.247927513 |
| 23046 | KIF21B | 5.247927513 | 5.247927513 | -5.247927513 |
| 387486 | LINC00320 | 5.247927513 | 5.247927513 | -5.247927513 |
| 153770 | PLAC8L1 | 5.247927513 | 5.247927513 | -5.247927513 |
| 7096 | TLR1 | 5.247927513 | 5.247927513 | -5.247927513 |
| 9215 | LARGE | 5.247927513 | 5.247927513 | -5.247927513 |
| 130576 | LYPD6B | 5.247927513 | 5.247927513 | -5.247927513 |
| 5016 | OVGP1 | 5.247927513 | 5.247927513 | -5.247927513 |
| 136853 | SRCRB4D | 5.247927513 | 5.247927513 | -5.247927513 |
| 10157 | AASS | 5.247927513 | 5.247927513 | -5.247927513 |
| 255057 | C19orf26 | 5.247927513 | 5.247927513 | -5.247927513 |
| 6439 | SFTPB | 5.247927513 | 5.247927513 | -5.247927513 |
| 195977 | ANTXRL | 5.247927513 | 5.247927513 | -5.247927513 |
| 7051 | TGM1 | 5.247927513 | 5.247927513 | -5.247927513 |
| 51299 | NRN1 | 5.247927513 | 5.247927513 | -5.247927513 |
| 10417 | SPON2 | 5.247927513 | 5.247927513 | -5.247927513 |
| 5555 | PRH2 | 5.247927513 | 5.247927513 | -5.247927513 |
| 26797 | SNORD52 | 5.247927513 | 5.247927513 | -5.247927513 |
| 255809 | C19orf38 | 5.247927513 | 5.247927513 | -5.247927513 |
| 387742 | FAM99A | 5.247927513 | 5.247927513 | -5.247927513 |
| 56981 | PRDM11 | 5.247927513 | 5.247927513 | -5.247927513 |
| 84174 | SLA2 | 5.247927513 | 5.247927513 | -5.247927513 |
| 84894 | LINGO1 | 5.247927513 | 5.247927513 | -5.247927513 |
| 100130950 | LOC100130950 | 5.247927513 | 5.247927513 | -5.247927513 |
| 389084 | C2orf82 | 5.247927513 | 5.247927513 | -5.247927513 |
| 83650 | SLC35G5 | 5.247927513 | 5.247927513 | -5.247927513 |
| 123591 | C15orf27 | 5.247927513 | 5.247927513 | -5.247927513 |
| 286367 | LOC286367 | 5.247927513 | 5.247927513 | -5.247927513 |
| 10562 | OLFM4 | 5.247927513 | 5.247927513 | -5.247927513 |
| 92346 | C1orf105 | 5.247927513 | 5.247927513 | -5.247927513 |
| 130574 | LYPD6 | 5.247927513 | 5.247927513 | -5.247927513 |
| 8997 | KALRN | 5.247927513 | 5.247927513 | -5.247927513 |
| 80167 | C4orf29 | 5.247927513 | 5.247927513 | -5.247927513 |
| 400655 | LOC400655 | 5.247927513 | 5.247927513 | -5.247927513 |
| 647323 | LOC647323 | 5.247927513 | 5.247927513 | -5.247927513 |
| 151056 | PLB1 | 5.247927513 | 5.247927513 | -5.247927513 |
| 491 | ATP2B2 | 5.247927513 | 5.247927513 | -5.247927513 |
| 100129354 | NRADDP | 5.247927513 | 5.247927513 | -5.247927513 |
| 1588 | CYP19A1 | 5.247927513 | 5.247927513 | -5.247927513 |
| 56920 | SEMA3G | 5.247927513 | 5.247927513 | -5.247927513 |
| 56256 | SERTAD4 | 5.247927513 | 5.247927513 | -5.247927513 |
| 6775 | STAT4 | 5.247927513 | 5.247927513 | -5.247927513 |
| 1821 | DRP2 | 5.247927513 | 5.247927513 | -5.247927513 |
| 284100 | LOC284100 | 5.247927513 | 5.247927513 | -5.247927513 |
| 8326 | FZD9 | 5.247927513 | 5.247927513 | -5.247927513 |
| 145820 | LOC145820 | 5.247927513 | 5.247927513 | -5.247927513 |
| 196968 | DNM1P46 | 5.247927513 | 5.247927513 | -5.247927513 |
| 171022 | ABHD11-AS1 | 5.247927513 | 5.247927513 | -5.247927513 |
| 54718 | BTN2A3P | 5.247927513 | 5.247927513 | -5.247927513 |
| 146439 | CCDC64B | 5.247927513 | 5.247927513 | -5.247927513 |
| 11309 | SLCO2B1 | 5.247927513 | 5.247927513 | -5.247927513 |
| 654466 | KGFLP2 | 5.247927513 | 5.247927513 | -5.247927513 |
| 5176 | SERPINF1 | 5.247927513 | 5.247927513 | -5.247927513 |
| 55613 | MTMR8 | 5.247927513 | 5.247927513 | -5.247927513 |
| 4858 | NOVA2 | 5.247927513 | 5.247927513 | -5.247927513 |
| 1800 | DPEP1 | 5.247927513 | 5.247927513 | -5.247927513 |
| 123904 | NRN1L | 5.247927513 | 5.247927513 | -5.247927513 |
| 440300 | LOC440300 | 5.247927513 | 5.247927513 | -5.247927513 |
| 54436 | SH3TC1 | 5.247927513 | 5.247927513 | -5.247927513 |
| 375287 | RBM43 | 5.247927513 | 5.247927513 | -5.247927513 |
| 56000 | NXF3 | 5.247927513 | 5.247927513 | -5.247927513 |
| 388697 | HRNR | 5.247927513 | 5.247927513 | -5.247927513 |
| 2868 | GRK4 | 5.247927513 | 5.247927513 | -5.247927513 |
| 284805 | C20orf203 | 5.247927513 | 5.247927513 | -5.247927513 |
| 145226 | RDH12 | 5.247927513 | 5.247927513 | -5.247927513 |
| 5999 | RGS4 | 5.247927513 | 5.247927513 | -5.247927513 |
| 132158 | GLYCTK | 5.247927513 | 5.247927513 | -5.247927513 |
| 2529 | FUT7 | 5.247927513 | 5.247927513 | -5.247927513 |
| 440078 | FAM66C | 5.247927513 | 5.247927513 | -5.247927513 |
| 390195 | OR5AN1 | 5.247927513 | 5.247927513 | -5.247927513 |
| 786 | CACNG1 | 5.247927513 | 5.247927513 | -5.247927513 |
| 284186 | TMEM105 | 5.247927513 | 5.247927513 | -5.247927513 |
| 51062 | ATL1 | 5.247927513 | 5.247927513 | -5.247927513 |
| 6916 | TBXAS1 | 5.247927513 | 5.247927513 | -5.247927513 |
| 440836 | ODF3B | 5.247927513 | 5.247927513 | -5.247927513 |
| 133491 | C5orf47 | 5.247927513 | 5.247927513 | -5.247927513 |
| 7067 | THRA | 5.030521945 | 1.455082218 | -2.040044719 |
| 80307 | FER1L4 | 4.609531222 | 3.949467266 | -2.822775261 |
| 23031 | MAST3 | 4.508628809 | 1.712162203 | -1.059588796 |
| 12 | SERPINA3 | 4.473317145 | 2.544090219 | -3.789501257 |
| 79661 | NEIL1 | 4.233871918 | 3.648909418 | -2.243394692 |
| 6038 | RNASE4 | 4.195015982 | 1.506305557 | -2.622764533 |
| 9536 | PTGES | 4.15508451 | 1.993621087 | -3.570122009 |
| 5163 | PDK1 | 4.091981858 | 1.691443929 | -2.08239581 |
| 51754 | TMEM8B | 3.918863237 | 2.48781342 | -2.906039197 |
| 5064 | PALM | 3.862616696 | 3.536846535 | -2.853030647 |
| 115557 | ARHGEF25 | 3.844829627 | 1.854352402 | -2.048363022 |
| 80221 | ACSF2 | 3.844829627 | 3.259867127 | -9.092757141 |
| 388228 | SBK1 | 3.749151133 | 1.411847812 | -1.619868116 |
| 168544 | ZNF467 | 3.740757173 | 8.988684687 | -2.759865996 |
| 123688 | AGPHD1 | 3.740757173 | 2.434095835 | -2.434095835 |
| 230 | ALDOC | 3.712228916 | 4.120868644 | -3.457231829 |
| 29842 | TFCP2L1 | 3.685763142 | 2.704871964 | -1.695285916 |
| 729359 | PLIN4 | 3.685763142 | 3.685763142 | -8.933690655 |
| 125965 | COX6B2 | 3.628589433 | 1.832122827 | -3.043626932 |
| 9762 | ProSAPiP1 | 3.572251449 | 1.775784843 | -2 |
| 64122 | FN3K | 3.500621586 | 2.500621586 | -2.27640643 |
| 2550 | GABBR1 | 3.497399469 | 9.726218159 | -2.032731202 |
| 376497 | SLC27A1 | 3.462532523 | 3.043232997 | -2.62500722 |
| 1902 | LPAR1 | 3.445559444 | 1.131244533 | -2.138898106 |
| 1731 | 1-Sep | 3.445559444 | 1.873307995 | -2.464668267 |
| 57715 | SEMA4G | 3.426264755 | 3.400729663 | -9.233619677 |
| 2921 | CXCL3 | 3.37778133 | 1.805529881 | -1.216317907 |
| 51148 | CERCAM | 3.337194852 | 1.900261857 | -2.194816177 |
| 114881 | OSBPL7 | 3.306661338 | 1.145197916 | -1.145197916 |
| 5582 | PRKCG | 3.285402219 | 1.683366205 | -1.854352402 |
| 27165 | GLS2 | 3.231852751 | 2.64689025 | -3.231852751 |
| 2036 | EPB41L1 | 3.222785687 | 2.211814569 | -1.934280594 |
| 51161 | C3orf18 | 3.19523609 | 1.456994339 | -1.777010313 |
| 5210 | PFKFB4 | 3.154735122 | 2.59964315 | -2.940814216 |
| 4303 | FOXO4 | 3.074000581 | 1.083523356 | -3.074000581 |
| 401303 | ZNF815P | 3.074000581 | 8.321928095 | -1.767339243 |
| 924 | CD7 | 3.074000581 | 8.321928095 | -1.767339243 |
| 80164 | FLJ22184 | 3.069162025 | 2.647698256 | -3.043626932 |
| 58985 | IL22RA1 | 3.069162025 | 1.638112207 | -1.638112207 |
| 219654 | ZCCHC24 | 3.027389998 | 2.020194497 | -2.06638413 |
| 26232 | FBXO2 | 3.019058336 | 1.166379423 | -2.88016023 |
| 197257 | LDHD | 2.985692163 | 1.679030825 | -1.189225557 |
| 284297 | SSC5D | 2.985692163 | 2.400729663 | -2.400729663 |
| 275 | AMT | 2.985692163 | 2.004800986 | -8.233619677 |
| 7108 | TM7SF2 | 2.975798941 | 2.970323811 | -3.452475506 |
| 575 | BAI1 | 2.958533383 | 2.948947334 | -2.948947334 |
| 10675 | CSPG5 | 2.92721058 | 3.252980741 | -2.437405312 |
| 388588 | LOC388588 | 2.921951104 | 1.97987108 | -3.193022829 |
| 1373 | CPS1 | 2.891623839 | 2.306661338 | -8.139551352 |
| 54828 | BCAS3 | 2.886132035 | 1.649092838 | -2.138898106 |
| 100507421 | LOC100507421 | 2.86393845 | 2.048363022 | -9.092757141 |
| 89848 | FCHSD1 | 2.815199939 | 2.028443978 | -1.290202227 |
| 84102 | SLC41A2 | 2.790991476 | 1.218740027 | -2.790991476 |
| 83546 | RTBDN | 2.790991476 | 2.206028975 | -8.038918989 |
| 322 | APBB1 | 2.754792591 | 1.724365557 | -2.070009721 |
| 25837 | RAB26 | 2.753708348 | 2.474848975 | -2.516990643 |
| 80235 | PIGZ | 2.723860607 | 1.458270496 | -1.458270496 |
| 81544 | GDPD5 | 2.704871964 | 3.685763142 | -3.100800641 |
| 4987 | OPRL1 | 2.69945729 | 1.475242133 | -1.37497801 |
| 2065 | ERBB3 | 2.688710426 | 1.707819249 | -1.707819249 |
| 84698 | CAPS2 | 2.688710426 | 1.382049087 | -2.103747925 |
| 138311 | FAM69B | 2.609334719 | 1.187101718 | -1.79375929 |
| 26297 | SERGEF | 2.574657492 | 1.114759209 | -2.56442557 |
| 84033 | OBSCN | 2.572251449 | 7.820178962 | -1.265590111 |
| 3643 | INSR | 2.572251449 | 1.591360272 | -2.572251449 |
| 728730 | LOC728730 | 2.572251449 | 1.987288948 | -7.820178962 |
| 283120 | H19 | 2.562706002 | 3.024811755 | -2.771625136 |
| 85315 | PAQR8 | 2.56175511 | 2.071949842 | -1.020946938 |
| 79816 | TLE6 | 2.538168289 | 1.530514717 | -3.844829627 |
| 1999 | ELF3 | 2.503179096 | 1.646658117 | -2.424569261 |
| 119391 | GSTO2 | 2.4870703 | 2.221480189 | -2.221480189 |
| 10014 | HDAC5 | 2.478406399 | 2.755515991 | -2.344727964 |
| 348487 | FAM131C | 2.458270496 | 2.040044719 | -1.040044719 |
| 57326 | PBXIP1 | 2.447743504 | 1.522193453 | -2.804228821 |
| 150291 | MORC2-AS1 | 2.445559444 | 7.693486957 | -7.693486957 |
| 5144 | PDE4D | 2.445559444 | 1.860596943 | -7.693486957 |
| 54739 | XAF1 | 2.445559444 | 1.860596943 | -1.860596943 |
| 125058 | TBC1D16 | 2.426264755 | 2.985692163 | -8.233619677 |
| 4355 | MPP2 | 2.426264755 | 2.004800986 | -1.413440714 |
| 770 | CA11 | 2.417702741 | 1.189225557 | -1.673923935 |
| 1831 | TSC22D3 | 2.413440714 | 2.189225557 | -1.413440714 |
| 50651 | SLC45A1 | 2.409520494 | 3.214124805 | -2.888354644 |
| 1838 | DTNB | 2.406860843 | 1.723044955 | -1.477633917 |
| 29911 | HOOK2 | 2.400739484 | 1.007296275 | -1.237399136 |
| 83543 | AIF1L | 2.387023123 | 1.090935033 | -2.54949782 |
| 566 | AZU1 | 2.373570882 | 2.625109649 | -3.142957954 |
| 664 | BNIP3 | 2.36761714 | 1.414217553 | -1.373980422 |
| 534 | ATP6V1G2 | 2.33219643 | 2.891623839 | -8.139551352 |
| 489 | ATP2A3 | 2.328914525 | 2.321928095 | -2.078777113 |
| 23149 | FCHO1 | 2.327687364 | 1.619868116 | -3.001917203 |
| 23604 | DAPK2 | 2.325770161 | 1.316184112 | -1.316184112 |
| 64077 | LHPP | 2.324486822 | 1.131041654 | -1.845783008 |
| 283335 | LOC283335 | 2.306661338 | 2.306661338 | -7.554588852 |
| 116843 | SLC18B1 | 2.306661338 | 1.325770161 | -1.325770161 |
| 27076 | LYPD3 | 2.306661338 | 7.554588852 | -2.306661338 |
| 4111 | MAGEA12 | 2.306661338 | 7.554588852 | -7.554588852 |
| 41 | ASIC1 | 2.282933963 | 1.954097499 | -1.954097499 |
| 11156 | PTP4A3 | 2.272578179 | 2.86393845 | -9.092757141 |
| 25878 | MXRA5 | 2.265590111 | 1.581774223 | -2 |
| 100506835 | LOC100506835 | 2.252980741 | 1.545161493 | -1.337141189 |
| 25809 | TTLL1 | 2.250961574 | 1.925191412 | -2.250961574 |
| 9886 | RHOBTB1 | 2.250961574 | 1.925191412 | -2.64689025 |
| 1606 | DGKA | 2.249277972 | 2.821461868 | -2.447066353 |
| 84189 | SLITRK6 | 2.231564067 | 8.038918989 | -8.038918989 |
| 9415 | FADS2 | 2.230040523 | 2.103379209 | -1.257286405 |
| 4013 | VWA5A | 2.218886478 | 1.276030385 | -1.666982266 |
| 9096 | TBX18 | 2.189707614 | 1.106771273 | -1.032166337 |
| 148252 | DIRAS1 | 2.183429266 | 2.095700416 | -2.178913785 |
| 2264 | FGFR4 | 2.176322773 | 8.405141463 | -2.176322773 |
| 2781 | GNAZ | 2.176322773 | 1.584962501 | -2.176322773 |
| 7754 | ZNF204P | 2.176322773 | 1.850552611 | -2.176322773 |
| 5654 | HTRA1 | 2.155626618 | 1.619868116 | -1.318101315 |
| 55200 | PLEKHG6 | 2.152951923 | 1.172060746 | -7.400879436 |
| 9980 | DOPEY2 | 2.152951923 | 7.400879436 | -7.400879436 |
| 3202 | HOXA5 | 2.152951923 | 2.152951923 | -2.152951923 |
| 60489 | APOBEC3G | 2.152951923 | 1.567989422 | -2.152951923 |
| 64411 | ARAP3 | 2.152951923 | 7.400879436 | -1.567989422 |
| 51129 | ANGPTL4 | 2.151330971 | 1.17973761 | -2.472009438 |
| 6919 | TCEA2 | 2.143820599 | 1.136563045 | -1.645974512 |
| 2979 | GUCA1B | 2.129283017 | 1.116458977 | -1.707819249 |
| 83450 | LRRC48 | 2.129283017 | 2.103747925 | -2.103747925 |
| 58191 | CXCL16 | 2.129283017 | 1.116458977 | -2.103747925 |
| 29923 | HILPDA | 2.126211605 | 1.179360705 | -1.500185046 |
| 29121 | CLEC2D | 2.113511693 | 1.524299719 | -1.240203697 |
| 114879 | OSBPL5 | 2.113511693 | 2.113511693 | -8.933690655 |
| 5165 | PDK3 | 2.101910781 | 1.996171528 | -2.320650808 |
| 9744 | ACAP1 | 2.101402032 | 2.74350844 | -2.378511623 |
| 3757 | KCNH2 | 2.093109404 | 1.083523356 | -1.277533976 |
| 3718 | JAK3 | 2.093109404 | 2.501749132 | -1.277533976 |
| 115908 | CTHRC1 | 2.093109404 | 1.083523356 | -1.277533976 |
| 2941 | GSTA4 | 2.093109404 | 2.093109404 | -1.767339243 |
| 83943 | IMMP2L | 2.071119991 | 1.216317907 | -1.216317907 |
| 11240 | PADI2 | 2.069704549 | 3.390383016 | -2.702004019 |
| 51090 | PLLP | 2.059334139 | 1.399270183 | -1.683366205 |
| 83937 | RASSF4 | 2.049630768 | 2.723860607 | -1.234055339 |
| 9563 | H6PD | 2.025796001 | 2.219149498 | -2.066013101 |
| 154091 | SLC2A12 | 2.022236474 | 1.187101718 | -1.79375929 |
| 10161 | LPAR6 | 2.01282404 | 1.987288948 | -2.572251449 |
| 170961 | ANKRD24 | 2.01282404 | 7.820178962 | -2.572251449 |
| 256949 | KANK3 | 2.01282404 | 7.820178962 | -7.820178962 |
| 148170 | CDC42EP5 | 2.01282404 | 1.987288948 | -1.591360272 |
| 7137 | TNNI3 | 2.00823615 | 1.105021213 | -1.348172194 |
| 8448 | DOC2A | 2.00698643 | 2.455082218 | -2.649092838 |
| 3791 | KDR | 2.004800986 | 8.233619677 | -2.004800986 |
| 9537 | TP53I11 | 2.003198882 | 1.827060331 | -2.371498951 |
| 64788 | LMF1 | 2.0031977 | 1.845656423 | -2.359683017 |
| 6319 | SCD | 2.001339485 | 2.359059013 | -2.596690474 |
| 10156 | RASA4 | 2 | 3.306661338 | -2.721698838 |
| 727 | C5 | 2 | 1.734409889 | -1.316184112 |
| 158219 | TTC39B | 1.990477226 | 7.238404739 | -1.009586049 |
| 29767 | TMOD2 | 1.990477226 | 7.238404739 | -1.009586049 |
| 388743 | CAPN8 | 1.990477226 | 7.238404739 | -7.238404739 |
| 5787 | PTPRB | 1.990477226 | 7.238404739 | -7.238404739 |
| 124045 | C16orf55 | 1.990477226 | 1.405514725 | -1.405514725 |
| 1382 | CRABP2 | 1.98960408 | 2.99182243 | -1.92605953 |
| 2026 | ENO2 | 1.979506561 | 1.739302864 | -1.309953888 |
| 55084 | SOBP | 1.960455932 | 1.207747602 | -1.183790861 |
| 54885 | TBC1D8B | 1.958533383 | 1.625109649 | -2.958533383 |
| 92689 | FAM114A1 | 1.954598605 | 1.069209911 | -1.375190279 |
| 10610 | ST6GALNAC2 | 1.939208316 | 2.063466419 | -2.805529881 |
| 57153 | SLC44A2 | 1.934629911 | 1.52093383 | -2.069454334 |
| 6196 | RPS6KA2 | 1.925191412 | 1.659601302 | -1.241375525 |
| 11037 | STON1 | 1.925191412 | 2.250961574 | -3.231852751 |
| 80724 | ACAD10 | 1.925191412 | 3.231852751 | -1.435386145 |
| 5033 | P4HA1 | 1.924247778 | 2.195621484 | -2.171980416 |
| 8991 | SELENBP1 | 1.921048659 | 1.501749132 | -2.277533976 |
| 9249 | DHRS3 | 1.920781553 | 2.298148635 | -2.76877846 |
| 644172 | LOC644172 | 1.910732662 | 8.139551352 | -1.31937239 |
| 22846 | VASH1 | 1.910732662 | 8.139551352 | -1.910732662 |
| 284751 | LOC284751 | 1.886132035 | 2.445559444 | -7.693486957 |
| 6289 | SAA2 | 1.886132035 | 1.138898106 | -1.138898106 |
| 414918 | FAM116B | 1.886132035 | 7.693486957 | -7.693486957 |
| 114822 | RHPN1 | 1.879124854 | 1.648511926 | -1.2863467 |
| 54800 | KLHL24 | 1.869939459 | 2.777961137 | -1.490384547 |
| 23179 | RGL1 | 1.86393845 | 1.272578179 | -1.272578179 |
| 57555 | NLGN2 | 1.858760535 | 2.13177903 | -1.354399167 |
| 10279 | PRSS16 | 1.857165222 | 1.967723597 | -2.378511623 |
| 2250 | FGF5 | 1.850552611 | 1.360747344 | -2.176322773 |
| 153768 | PRELID2 | 1.850552611 | 1.850552611 | -1.850552611 |
| 339942 | H1FX-AS1 | 1.850552611 | 1.850552611 | -1.850552611 |
| 9509 | ADAMTS2 | 1.850552611 | 2.176322773 | -1.166736724 |
| 84063 | KIRREL2 | 1.848423172 | 2.848423172 | -2.848423172 |
| 1299 | COL9A3 | 1.848423172 | 1.993621087 | -1.582833061 |
| 50853 | VILL | 1.843093945 | 2.647698256 | -8.876516947 |
| 408 | ARRB1 | 1.819519276 | 1.371903408 | -2.570147355 |
| 6528 | SLC5A5 | 1.811927652 | 2.063466419 | -1.689070904 |
| 25956 | SEC31B | 1.810843908 | 2.26243714 | -1.405444857 |
| 283870 | C16orf79 | 1.810100299 | 2.206028975 | -1.484330138 |
| 11118 | BTN3A2 | 1.799264335 | 2.240203697 | -2.695285916 |
| 54854 | FAM83E | 1.796466606 | 1.796466606 | -1.211504105 |
| 55160 | ARHGEF10L | 1.796466606 | 7.044394119 | -7.044394119 |
| 91523 | PCED1B | 1.796466606 | 1.211504105 | -1.796466606 |
| 400619 | LINC00511 | 1.796466606 | 1.796466606 | -7.044394119 |
| 10391 | CORO2B | 1.796466606 | 7.044394119 | -7.044394119 |
| 140825 | NEURL2 | 1.796466606 | 7.044394119 | -1.796466606 |
| 929 | CD14 | 1.796466606 | 7.044394119 | -1.796466606 |
| 3696 | ITGB8 | 1.796466606 | 1.796466606 | -1.796466606 |
| 166012 | CHST13 | 1.796466606 | 7.044394119 | -7.044394119 |
| 27112 | FAM155B | 1.796466606 | 7.044394119 | -1.796466606 |
| 374654 | KIF7 | 1.767339243 | 2.489038081 | -2.489038081 |
| 66004 | LYNX1 | 1.764105077 | 2.004377233 | -2.418883451 |
| 1365 | CLDN3 | 1.759865996 | 1.052046748 | -1.168505724 |
| 727910 | TLCD2 | 1.752945524 | 1.681392263 | -1.681392263 |
| 79187 | FSD1 | 1.74898357 | 1.74350844 | -1.026351327 |
| 53345 | TM6SF2 | 1.74723393 | 7.554588852 | -7.554588852 |
| 8718 | TNFRSF25 | 1.74723393 | 1.325770161 | -1.721698838 |
| 114899 | C1QTNF3 | 1.74723393 | 2.306661338 | -1.721698838 |
| 445 | ASS1 | 1.74723393 | 7.554588852 | -7.554588852 |
| 92840 | REEP6 | 1.738671916 | 2.095157233 | -2.095157233 |
| 112755 | STX1B | 1.738671916 | 1.31937239 | -1.202913413 |
| 6508 | SLC4A3 | 1.738671916 | 2.095157233 | -2.910732662 |
| 28986 | MAGEH1 | 1.736965594 | 1.013251023 | -1.214718493 |
| 84961 | FBXL20 | 1.734409889 | 2.721698838 | -1.734409889 |
| 64221 | ROBO3 | 1.733117939 | 1.098935364 | -1.543532661 |
| 23492 | CBX7 | 1.729258555 | 1.176180115 | -1.615601773 |
| 79885 | HDAC11 | 1.717600269 | 1.880329769 | -2.071949842 |
| 9649 | RALGPS1 | 1.71419662 | 2.286380516 | -3.028443978 |
| 11067 | C10orf10 | 1.707819249 | 7.936637939 | -1.382049087 |
| 340061 | TMEM173 | 1.707819249 | 1.382049087 | -1.116458977 |
| 51450 | PRRX2 | 1.70761477 | 2.64232331 | -3.224097533 |
| 83723 | FAM57B | 1.68729917 | 2.032731202 | -2.163975735 |
| 23108 | RAP1GAP2 | 1.686520172 | 2.172756592 | -1.928264246 |
| 57333 | RCN3 | 1.679030825 | 1.679030825 | -1.679030825 |
| 1952 | CELSR2 | 1.676568315 | 1.968749066 | -1.666982266 |
| 7059 | THBS3 | 1.659601302 | 1.659601302 | -1.925191412 |
| 6553 | SLC9A5 | 1.659601302 | 2.250961574 | -2.250961574 |
| 23475 | QPRT | 1.659601302 | 2.250961574 | -1.435386145 |
| 949 | SCARB1 | 1.649948304 | 2.430263974 | -3.291717161 |
| 8623 | ASMTL | 1.647468523 | 1.133030314 | -1.590573588 |
| 9311 | ASIC3 | 1.640779715 | 1.479416727 | -1.221480189 |
| 978 | CDA | 1.640779715 | 2.812840461 | -2.812840461 |
| 1852 | DUSP9 | 1.628935126 | 2.052046748 | -2.052046748 |
| 65018 | PINK1 | 1.624881511 | 1.021723478 | -1.661778098 |
| 493 | ATP2B4 | 1.621076903 | 1.421591153 | -1.030639272 |
| 8839 | WISP2 | 1.620398897 | 2.612731767 | -3.273574232 |
| 6604 | SMARCD3 | 1.61980425 | 1.610218201 | -1.703964698 |
| 1759 | DNM1 | 1.615254574 | 2.193292551 | -2.385709913 |
| 79589 | RNF128 | 1.609334719 | 1.278457667 | -1.79375929 |
| 54756 | IL17RD | 1.593524514 | 7.400879436 | -2.152951923 |
| 1264 | CNN1 | 1.593524514 | 2.152951923 | -1.567989422 |
| 100272217 | LOC100272217 | 1.592285842 | 2.071119991 | -1.387304104 |
| 57593 | EBF4 | 1.592285842 | 8.625708843 | -8.625708843 |
| 30008 | EFEMP2 | 1.591948931 | 4.030521945 | -4.030521945 |
| 27128 | CYTH4 | 1.591791839 | 1.68182404 | -1.78958022 |
| 89849 | ATG16L2 | 1.591360272 | 1.126692005 | -1.126692005 |
| 79777 | ACBD4 | 1.591360272 | 7.820178962 | -7.820178962 |
| 80231 | CXorf21 | 1.591360272 | 2.572251449 | -7.820178962 |
| 7903 | ST8SIA4 | 1.591360272 | 1.591360272 | -1.591360272 |
| 338707 | B4GALNT4 | 1.588243592 | 2.008546407 | -1.867929238 |
| 21 | ABCA3 | 1.58780525 | 1.876679661 | -1.337632996 |
| 23331 | TTC28 | 1.586789854 | 1.141893066 | -1.141893066 |
| 8029 | CUBN | 1.584962501 | 2.584962501 | -1.730160416 |
| 57010 | CABP4 | 1.584962501 | 1.584962501 | -1.360747344 |
| 147700 | KLC3 | 1.572251449 | 1.572251449 | -6.820178962 |
| 51364 | ZMYND10 | 1.572251449 | 1.572251449 | -6.820178962 |
| 113451 | ADC | 1.572251449 | 6.820178962 | -1.572251449 |
| 4756 | NEO1 | 1.572251449 | 1.572251449 | -6.820178962 |
| 374500 | THSD1P1 | 1.572251449 | 1.572251449 | -1.572251449 |
| 204801 | NLRP11 | 1.572251449 | 6.820178962 | -6.820178962 |
| 27294 | DHDH | 1.572251449 | 6.820178962 | -6.820178962 |
| 149840 | C20orf196 | 1.572251449 | 6.820178962 | -6.820178962 |
| 150379 | PNPLA5 | 1.572251449 | 6.820178962 | -6.820178962 |
| 257407 | C2orf72 | 1.572251449 | 6.820178962 | -6.820178962 |
| 53358 | SHC3 | 1.572251449 | 6.820178962 | -6.820178962 |
| 115704 | EVI5L | 1.566210717 | 1.772392911 | -1.559241162 |
| 157638 | FAM84B | 1.559083869 | 1.54949782 | -1.967723597 |
| 6376 | CX3CL1 | 1.548162583 | 1.035514287 | -1.79970135 |
| 26508 | HEYL | 1.54711915 | 1.824228741 | -1.413440714 |
| 100128822 | LOC100128822 | 1.54711915 | 1.540132719 | -1.413440714 |
| 388650 | FAM69A | 1.543621705 | 1.536652151 | -1.911691582 |
| 79957 | PAQR6 | 1.538168289 | 9.092757141 | -2.048363022 |
| 9518 | GDF15 | 1.533030723 | 1.515061821 | -1.844528509 |
| 130612 | TMEM198 | 1.525569572 | 2.250604956 | -1.51533765 |
| 10062 | NR1H3 | 1.52116585 | 1.734409889 | -1.145197916 |
| 1241 | LTB4R | 1.508146904 | 1.759685671 | -1.083523356 |
| 93129 | ORAI3 | 1.504507569 | 1.666982266 | -3.350798153 |
| 140564 | APOBEC3D | 1.501749132 | 2.093109404 | -2.489038081 |
| 56977 | STOX2 | 1.501749132 | 1.501749132 | -2.093109404 |
| 140462 | ASB9 | 1.501749132 | 2.093109404 | -1.501749132 |
| 56895 | AGPAT4 | 1.501321186 | 1.851067225 | -1.559241162 |
| 84988 | PPP1R16A | 1.500851546 | 1.142957954 | -1.625109649 |
| 283070 | LOC283070 | 1.497399469 | 1.581559917 | -1.242402382 |
| 6857 | SYT1 | 1.496277347 | 1.46533364 | -1.46533364 |
| 2281 | FKBP1B | 1.493303253 | 1.152543483 | -2.153375247 |
| 284615 | ANKRD34A | 1.4870703 | 2.4870703 | -2.812840461 |
| 205 | AK4 | 1.485197937 | 1.777198872 | -1.975599856 |
| 83986 | ITFG3 | 1.470243865 | 1.010628311 | -2.188113948 |
| 6352 | CCL5 | 1.464668267 | 2.464668267 | -1.284096021 |
| 115330 | GPR146 | 1.464668267 | 7.693486957 | -7.693486957 |
| 129807 | NEU4 | 1.464668267 | 1.464668267 | -1.860596943 |
| 2644 | GCHFR | 1.460955861 | 1.825624406 | -1.316732385 |
| 93145 | OLFM2 | 1.45616073 | 1.264157563 | -1.445928807 |
| 9203 | ZMYM3 | 1.448906168 | 1.704553777 | -1.363874859 |
| 9567 | GTPBP1 | 1.447743504 | 1.0252557 | -2.155135983 |
| 126567 | C2CD4C | 1.446357262 | 1.241375525 | -1.070389328 |
| 2934 | GSN | 1.445312982 | 2.607656212 | -2.294868771 |
| 6253 | RTN2 | 1.438640841 | 1.721371049 | -2.30845985 |
| 494513 | DFNB59 | 1.434095835 | 1.168505724 | -1.426442263 |
| 84445 | LZTS2 | 1.431206436 | 9.361943774 | -2.807354922 |
| 11174 | ADAMTS6 | 1.431049817 | 1.009586049 | -1.009586049 |
| 149428 | BNIPL | 1.431049817 | 1.990477226 | -1.405514725 |
| 29931 | LINC00312 | 1.431049817 | 1.990477226 | -7.238404739 |
| 150000 | ABCC13 | 1.431049817 | 7.238404739 | -7.238404739 |
| 10603 | SH2B2 | 1.431049817 | 7.238404739 | -1.405514725 |
| 158801 | NKAPP1 | 1.431049817 | 7.238404739 | -7.238404739 |
| 9028 | RHBDL1 | 1.431049817 | 1.405514725 | -1.009586049 |
| 26468 | LHX6 | 1.431049817 | 1.009586049 | -1.990477226 |
| 340554 | ZC3H12B | 1.431049817 | 1.990477226 | -7.238404739 |
| 85442 | KNDC1 | 1.431049817 | 7.238404739 | -7.238404739 |
| 83959 | SLC4A11 | 1.421588834 | 1.705789698 | -2.727657852 |
| 339761 | CYP27C1 | 1.419838486 | 1.671377253 | -1.671377253 |
| 79366 | HMGN5 | 1.419299526 | 1.257936538 | -1.775784843 |
| 201625 | DNAH12 | 1.419299526 | 2.265590111 | -2 |
| 151174 | LOC151174 | 1.417702741 | 1.057030945 | -1.998403215 |
| 4054 | LTBP3 | 1.410709985 | 1.527481152 | -1.127244598 |
| 55076 | TMEM45A | 1.397862355 | 1.059523746 | -1.825283579 |
| 84446 | BRSK1 | 1.396890153 | 2.792818829 | -2.071119991 |
| 1512 | CTSH | 1.393707436 | 1.602856622 | -2.882643049 |
| 24142 | NAT6 | 1.385209933 | 1.475242133 | -1.957393829 |
| 152 | ADRA2C | 1.382049087 | 1.707819249 | -7.936637939 |
| 160335 | TMTC2 | 1.382049087 | 2.688710426 | -2.688710426 |
| 619383 | SCARNA9 | 1.382049087 | 1.707819249 | -1.382049087 |
| 55620 | STAP2 | 1.376091219 | 1.660947727 | -2.129451251 |
| 8659 | ALDH4A1 | 1.375039431 | 1.226488081 | -1.765534746 |
| 134429 | STARD4 | 1.371718462 | 2.176322773 | -3.15721395 |
| 9001 | HAP1 | 1.371718462 | 8.405141463 | -1.360747344 |
| 51162 | EGFL7 | 1.357077333 | 1.476807668 | -1.997289695 |
| 79774 | GRTP1 | 1.355158624 | 2.221480189 | -1.997265032 |
| 78991 | PCYOX1L | 1.35166118 | 1.483238438 | -1.719731057 |
| 9479 | MAPK8IP1 | 1.342848747 | 2.12509522 | -1.189225557 |
| 23151 | GRAMD4 | 1.342583279 | 1.254854429 | -1.338067798 |
| 26470 | SEZ6L2 | 1.34149854 | 1.739986574 | -2.037222464 |
| 5754 | PTK7 | 1.340059529 | 2.004616628 | -1.973840185 |
| 10570 | DPYSL4 | 1.327304127 | 2.184563955 | -1.73339267 |
| 6339 | SCNN1D | 1.326537348 | 1.832890014 | -1.025535092 |
| 400954 | EML6 | 1.325770161 | 1.510194732 | -1.734409889 |
| 2035 | EPB41 | 1.325770161 | 1.145197916 | -1.510194732 |
| 8786 | RGS11 | 1.325770161 | 7.554588852 | -2.306661338 |
| 146880 | LOC146880 | 1.325770161 | 1.325770161 | -1.721698838 |
| 404550 | C16orf74 | 1.325338723 | 2.906039197 | -2.68182404 |
| 56241 | SUSD2 | 1.31937239 | 1.095157233 | -1.31937239 |
| 80320 | SP6 | 1.306661338 | 1.306661338 | -1.306661338 |
| 154761 | LOC154761 | 1.306661338 | 6.554588852 | -6.554588852 |
| 3910 | LAMA4 | 1.306661338 | 1.306661338 | -6.554588852 |
| 9597 | SMAD5-AS1 | 1.306661338 | 6.554588852 | -6.554588852 |
| 340348 | TSPAN33 | 1.306661338 | 6.554588852 | -6.554588852 |
| 1117 | CHI3L2 | 1.306661338 | 6.554588852 | -6.554588852 |
| 197320 | ZNF778 | 1.306661338 | 6.554588852 | -1.306661338 |
| 379013 | RNF138P1 | 1.306661338 | 1.306661338 | -6.554588852 |
| 4660 | PPP1R12B | 1.302882339 | 3.004800986 | -2.413440714 |
| 687 | KLF9 | 1.302882339 | 2.413440714 | -1.189225557 |
| 63035 | BCORL1 | 1.302675134 | 1.574048841 | -2.183046273 |
| 883 | CCBL1 | 1.30256277 | 2.296164999 | -2.706953025 |
| 389792 | IER5L | 1.30256277 | 1.632528184 | -1.419376435 |
| 65010 | SLC26A6 | 1.289302947 | 1.945439063 | -2.610857722 |
| 283130 | SLC25A45 | 1.288505093 | 3.074000581 | -1.501749132 |
| 3306 | HSPA2 | 1.288505093 | 2.093109404 | -8.321928095 |
| 5211 | PFKL | 1.281538971 | 2.43920791 | -2.126441387 |
| 221935 | SDK1 | 1.279934006 | 1.331088094 | -1.066013101 |
| 388581 | FAM132A | 1.267835392 | 1.827060331 | -2.3347664 |
| 80853 | JHDM1D | 1.265590111 | 7.820178962 | -1.987288948 |
| 51222 | ZNF219 | 1.263034406 | 1.537656786 | -2.154549776 |
| 63891 | RNF123 | 1.257233254 | 1.278077508 | -1.396807703 |
| 79641 | ROGDI | 1.257233254 | 1.821648378 | -1.593645988 |
| 26471 | NUPR1 | 1.252307999 | 2.245910228 | -2.129451251 |
| 136288 | C7orf57 | 1.250961574 | 1.659601302 | -8.479780264 |
| 90139 | TSPAN18 | 1.250961574 | 2.250961574 | -3.231852751 |
| 57139 | RGL3 | 1.248895027 | 1.906714754 | -1.55502855 |
| 3708 | ITPR1 | 1.243692684 | 1.375269942 | -1.236723129 |
| 116966 | WDR17 | 1.239530469 | 1.869058522 | -1.584962501 |
| 8519 | IFITM1 | 1.237039197 | 1.796466606 | -1.211504105 |
| 112703 | FAM71E1 | 1.237039197 | 7.044394119 | -7.044394119 |
| 284348 | LYPD5 | 1.237039197 | 7.044394119 | -7.044394119 |
| 144486 | LOC144486 | 1.237039197 | 1.796466606 | -7.044394119 |
| 3137 | HLA-J | 1.237039197 | 7.044394119 | -7.044394119 |
| 4494 | MT1F | 1.237039197 | 1.211504105 | -1.796466606 |
| 64061 | TSPYL2 | 1.235467938 | 1.430263974 | -1.66450414 |
| 9902 | MRC2 | 1.232312236 | 1.420008641 | -2.017354867 |
| 6523 | SLC5A1 | 1.226571121 | 1.364807163 | -1.290202227 |
| 2745 | GLRX | 1.226537749 | 1.500553712 | -1.540369174 |
| 81622 | UNC93B1 | 1.226342007 | 1.589351598 | -2.494924062 |
| 284716 | RIMKLA | 1.225767148 | 2.503062247 | -2.04449946 |
| 10000 | AKT3 | 1.221914708 | 1.037022131 | -1.094017496 |
| 79924 | ADM2 | 1.218740027 | 8.038918989 | -8.038918989 |
| 80757 | TMEM121 | 1.218740027 | 8.038918989 | -8.038918989 |
| 285512 | FAM13A-AS1 | 1.218740027 | 1.484330138 | -2.206028975 |
| 1788 | DNMT3A | 1.217991274 | 1.047041267 | -1.686293923 |
| 6513 | SLC2A1 | 1.214500601 | 2.192474294 | -2.348125165 |
| 3727 | JUND | 1.209199821 | 2.3102103 | -2.068119154 |
| 55022 | PID1 | 1.208814015 | 1.31937239 | -1.446064395 |
| 122525 | C14orf28 | 1.203634363 | 1.348832279 | -1.519818475 |
| 6405 | SEMA3F | 1.201633861 | 1.078777113 | -1.971020933 |
| 4351 | MPI | 1.19908034 | 1.632444487 | -1.35265806 |
| 652968 | GATSL3 | 1.190720201 | 1.804829047 | -1.460696216 |
| 91461 | PKDCC | 1.190016419 | 1.638112207 | -1.183029989 |
| 10385 | BTN2A2 | 1.190016419 | 1.314274522 | -1.314274522 |
| 147 | ADRA1B | 1.189707614 | 1.653949111 | -1.185445587 |
| 611 | OPN1SW | 1.176322773 | 2.176322773 | -2.572251449 |
| 7915 | ALDH5A1 | 1.176322773 | 1.584962501 | -1.166736724 |
| 4091 | SMAD6 | 1.174903496 | 1.750279947 | -2.168505724 |
| 23114 | NFASC | 1.172060746 | 7.400879436 | -2.152951923 |
| 5212 | VIT | 1.172060746 | 7.400879436 | -7.400879436 |
| 64711 | HS3ST6 | 1.169147897 | 1.347546473 | -1.884522783 |
| 24139 | EML2 | 1.158624969 | 2.0252557 | -2.443481477 |
| 51626 | DYNC2LI1 | 1.158624969 | 1.522193453 | -1.526694846 |
| 79948 | LPPR3 | 1.158439287 | 2.149781854 | -1.174656522 |
| 3726 | JUNB | 1.158337027 | 1.772445874 | -1.61368679 |
| 91 | ACVR1B | 1.154987834 | 1.096435651 | -1.204191832 |
| 7001 | PRDX2 | 1.146719377 | 1.610623004 | -1.91397815 |
| 5351 | PLOD1 | 1.138900909 | 1.738591612 | -1.446967936 |
| 23043 | TNIK | 1.138898106 | 1.464668267 | -7.693486957 |
| 144193 | AMDHD1 | 1.138898106 | 2.445559444 | -1.464668267 |
| 192683 | SCAMP5 | 1.138898106 | 7.693486957 | -2.445559444 |
| 8989 | TRPA1 | 1.134855335 | 1.750279947 | -1.052046748 |
| 20 | ABCA2 | 1.134504503 | 2.347165386 | -1.712162203 |
| 100129148 | LOC100129148 | 1.133678435 | 1.257936538 | -1.257936538 |
| 51257 | 2-Mar | 1.116755919 | 1.148280007 | -1.189225557 |
| 2549 | GAB1 | 1.116458977 | 1.382049087 | -2.103747925 |
| 100 | ADA | 1.116458977 | 7.936637939 | -2.688710426 |
| 5595 | MAPK3 | 1.114737888 | 1.745017078 | -1.522928222 |
| 80162 | ATHL1 | 1.111929709 | 3.402533432 | -2.482157736 |
| 81794 | ADAMTS10 | 1.110921814 | 1.897000909 | -1.280107919 |
| 23373 | CRTC1 | 1.110921814 | 2.105021213 | -2.105021213 |
| 163732 | CITED4 | 1.110719412 | 2.135633132 | -2.11409151 |
| 147015 | DHRS13 | 1.109249107 | 1.421591153 | -2.615601773 |
| 84557 | MAP1LC3A | 1.104656158 | 1.996171528 | -1.582409058 |
| 100133941 | CD24 | 1.103282817 | 1.774507557 | -2.096435651 |
| 23363 | OBSL1 | 1.100965146 | 1.093831905 | -1.765868163 |
| 1026 | CDKN1A | 1.094849059 | 1.109404895 | -1.027380436 |
| 79444 | BIRC7 | 1.094104828 | 2.086433815 | -2.26293147 |
| 51308 | REEP2 | 1.093109404 | 1.277533976 | -1.277533976 |
| 51171 | HSD17B14 | 1.093109404 | 1.501749132 | -1.277533976 |
| 4489 | MT1A | 1.089244031 | 1.081109603 | -1.038859084 |
| 54626 | HES2 | 1.086867596 | 1.36188982 | -1.546425684 |
| 3783 | KCNN4 | 1.084677715 | 2.078777113 | -1.531599275 |
| 254065 | BRWD3 | 1.079254643 | 1.355901215 | -1.557923248 |
| 727800 | RNF208 | 1.077789505 | 1.795659588 | -2.148280007 |
| 1748 | DLX4 | 1.074962058 | 1.689070904 | -2.387304104 |
| 79671 | NLRX1 | 1.074962058 | 1.996352223 | -1.634186997 |
| 256472 | TMEM151A | 1.071435138 | 1.996519431 | -3 |
| 5934 | RBL2 | 1.071435138 | 1.288345494 | -1.649092838 |
| 9123 | SLC16A3 | 1.063388942 | 1.193415055 | -1.377090384 |
| 126014 | OSCAR | 1.06336635 | 1.056793696 | -1.79375929 |
| 100133091 | LOC100133091 | 1.062735755 | 2.056337984 | -1.832122827 |
| 100128071 | LOC100128071 | 1.062050027 | 2.174412392 | -1.134519665 |
| 54813 | KLHL28 | 1.057947349 | 2.168505724 | -2.168505724 |
| 1364 | CLDN4 | 1.057947349 | 1.750279947 | -2.168505724 |
| 6764 | ST5 | 1.057947349 | 1.295197729 | -1.052046748 |
| 284207 | METRNL | 1.054786787 | 1.484545792 | -1.013280502 |
| 23096 | IQSEC2 | 1.046908743 | 1.596172309 | -1.824955833 |
| 83862 | TMEM120A | 1.044199804 | 1.298285253 | -1.563360246 |
| 3433 | IFIT2 | 1.043902822 | 2.281231514 | -1.37497801 |
| 1891 | ECH1 | 1.042223513 | 1.653263817 | -1.199314696 |
| 9064 | MAP3K6 | 1.039840265 | 1.264157563 | -1.770408087 |
| 64856 | VWA1 | 1.038462518 | 1.807182804 | -1.988940213 |
| 4343 | MOV10 | 1.034841749 | 1.443481477 | -1.028443978 |
| 254102 | EHBP1L1 | 1.031006057 | 2.089981367 | -1.537347593 |
| 140680 | C20orf96 | 1.02888112 | 2.179706022 | -2.296164999 |
| 2879 | GPX4 | 1.026703043 | 1.147332381 | -1.698727719 |
| 5155 | PDGFB | 1.023829236 | 1.303403318 | -1.405441016 |
| 115 | ADCY9 | 1.019085841 | 1.262000586 | -2.150663099 |
| 4133 | MAP2 | 1.018689073 | 1.31749461 | -1.567309727 |
| 147686 | ZNF418 | 1.01282404 | 6.820178962 | -6.820178962 |
| 4909 | NTF4 | 1.01282404 | 6.820178962 | -6.820178962 |
| 7480 | WNT10B | 1.01282404 | 1.572251449 | -6.820178962 |
| 2668 | GDNF | 1.01282404 | 1.572251449 | -1.572251449 |
| 64150 | DIO3OS | 1.01282404 | 6.820178962 | -1.572251449 |
| 64838 | FNDC4 | 1.01282404 | 6.820178962 | -6.820178962 |
| 100131187 | TSTD1 | 1.01282404 | 6.820178962 | -6.820178962 |
| 6515 | SLC2A3 | 1.01282404 | 6.820178962 | -6.820178962 |
| 10957 | PNRC1 | 1.012203123 | 1.425269919 | -2.468679695 |
| 9524 | TECR | 1.009879776 | 2.260335587 | -1.921751691 |
| 5152 | PDE9A | 1.009586049 | 1.405514725 | -1.009586049 |
| 3623 | INHA | 1.009586049 | 1.990477226 | -7.238404739 |
| 117166 | WFIKKN1 | 1.009586049 | 1.009586049 | -1.009586049 |
| 100130275 | LOC100130275 | 1.009586049 | 1.405514725 | -1.405514725 |
| 54753 | ZNF853 | 1.009586049 | 1.405514725 | -1.990477226 |
| 1397 | CRIP2 | 1.008256366 | 1.783516674 | -1.505987654 |
| 25946 | ZNF385A | 1.005495988 | 2.206028975 | -2.206028975 |
| 57462 | KIAA1161 | 1.005495988 | 1.810100299 | -1.218740027 |
| 200942 | KLHDC8B | 1.005125032 | 2.31937239 | -1.095157233 |
| 2710 | GK | 1.004800986 | 1.413440714 | -1.413440714 |
| 26160 | IFT172 | 1.004800986 | 2.004800986 | -2.400729663 |
| 7091 | TLE4 | 1.004262027 | 1.166736724 | -1.360747344 |
| 55893 | ZNF395 | 1.003838659 | 1.511473151 | -2.204191832 |
| 8912 | CACNA1H | 1.003571029 | 1.615601773 | -2.101575179 |
| 8784 | TNFRSF18 | 1.002132587 | 2.848423172 | -2.582833061 |
| 29108 | PYCARD | -8.813781191 | -10.25974326 | 7.562242424 |
| 11249 | NXPH2 | -7.930737338 | -7.820178962 | 7.409390936 |
| 1415 | CRYBB2 | -7.813781191 | -6.22881869 | 7.562242424 |
| 7562 | ZNF708 | -7.813781191 | -7.409390936 | 6.554588852 |
| 348180 | CTU2 | -7.686500527 | -7.044394119 | 8.144658243 |
| 2707 | GJB3 | -7.686500527 | -7.238404739 | 7.936637939 |
| 55027 | HEATR3 | -7.686500527 | -6.22881869 | 5.832890014 |
| 154 | ADRB2 | -7.554588852 | -5.247927513 | 6.820178962 |
| 79631 | EFTUD1 | -7.400879436 | -6.820178962 | 6.554588852 |
| 7056 | THBD | -7.400879436 | -5.247927513 | 5.247927513 |
| 269 | AMHR2 | -7.22881869 | -6.22881869 | 7.562242424 |
| 401491 | FLJ35024 | -7.22881869 | -9.236014192 | 8.405141463 |
| 439994 | LOC439994 | -7.033423002 | -7.820178962 | 8.62935662 |
| 201562 | PTPLB | -7.033423002 | -6.820178962 | 7.044394119 |
| 374383 | NCR3LG1 | -7.033423002 | -5.832890014 | 6.554588852 |
| 283518 | KCNRG | -7.033423002 | -7.044394119 | 5.247927513 |
| 94025 | MUC16 | -7.033423002 | -5.247927513 | 5.247927513 |
| 4360 | MRC1 | -6.820178962 | -6.22881869 | 5.247927513 |
| 636 | BICD1 | -6.820178962 | -6.554588852 | 7.409390936 |
| 440138 | ALG11 | -6.820178962 | -6.22881869 | 5.832890014 |
| 55753 | OGDHL | -6.820178962 | -5.832890014 | 5.832890014 |
| 85236 | HIST1H2BK | -6.820178962 | -6.554588852 | 6.820178962 |
| 51729 | WBP11 | -6.820178962 | -7.820178962 | 6.22881869 |
| 653275 | CFC1B | -6.820178962 | -8.144658243 | 6.554588852 |
| 5332 | PLCB4 | -6.820178962 | -6.554588852 | 5.247927513 |
| 6358 | CCL14 | -6.554588852 | -7.693486957 | 7.693486957 |
| 160760 | PPTC7 | -6.554588852 | -7.044394119 | 5.247927513 |
| 348926 | FAM86EP | -6.554588852 | -7.693486957 | 6.820178962 |
| 3670 | ISL1 | -6.554588852 | -5.247927513 | 5.247927513 |
| 1844 | DUSP2 | -6.554588852 | -7.238404739 | 6.22881869 |
| 165545 | DQX1 | -6.554588852 | -6.22881869 | 6.554588852 |
| 4163 | MCC | -6.554588852 | -7.693486957 | 8.238404739 |
| 1024 | CDK8 | -6.554588852 | -6.554588852 | 6.22881869 |
| 752014 | CEMP1 | -6.554588852 | -5.247927513 | 6.22881869 |
| 26153 | KIF26A | -6.554588852 | -7.044394119 | 6.820178962 |
| 9609 | RAB36 | -6.554588852 | -5.247927513 | 6.820178962 |
| 401232 | DKFZP686I15217 | -6.554588852 | -6.22881869 | 6.820178962 |
| 84083 | ZRANB3 | -6.554588852 | -5.247927513 | 7.044394119 |
| 6387 | CXCL12 | -6.22881869 | -6.820178962 | 5.832890014 |
| 6637 | SNRPG | -6.22881869 | -7.936637939 | 6.820178962 |
| 6302 | TSPAN31 | -6.22881869 | -5.832890014 | 5.832890014 |
| 55612 | FERMT1 | -6.22881869 | -5.247927513 | 5.247927513 |
| 27350 | APOBEC3C | -6.22881869 | -5.247927513 | 5.247927513 |
| 152485 | ZNF827 | -6.22881869 | -5.832890014 | 6.554588852 |
| 256933 | NPB | -6.22881869 | -6.22881869 | 5.832890014 |
| 374355 | CCDC172 | -6.22881869 | -6.22881869 | 5.832890014 |
| 57623 | ZFAT | -6.22881869 | -6.22881869 | 7.044394119 |
| 116211 | TM4SF19 | -6.22881869 | -6.22881869 | 6.554588852 |
| 200172 | SLFNL1 | -6.22881869 | -6.554588852 | 5.247927513 |
| 56413 | LTB4R2 | -6.22881869 | -6.22881869 | 6.22881869 |
| 8490 | RGS5 | -6.22881869 | -7.238404739 | 6.554588852 |
| 84631 | SLITRK2 | -6.22881869 | -6.22881869 | 5.247927513 |
| 55057 | AIM1L | -6.22881869 | -6.554588852 | 6.554588852 |
| 1517 | CTSL1P2 | -5.807354922 | -7.238404739 | 7.820178962 |
| 56126 | PCDHB10 | -5.807354922 | -5.247927513 | 5.247927513 |
| 7768 | ZNF225 | -5.807354922 | -6.22881869 | 6.22881869 |
| 677846 | SNORA80 | -5.807354922 | -5.247927513 | 5.832890014 |
| 9047 | SH2D2A | -5.807354922 | -5.247927513 | 5.247927513 |
| 2637 | GBX2 | -5.807354922 | -5.247927513 | 6.820178962 |
| 1193 | CLIC2 | -5.807354922 | -5.247927513 | 5.832890014 |
| 26686 | OR4E2 | -5.807354922 | -5.247927513 | 6.820178962 |
| 6795 | AURKC | -5.807354922 | -5.247927513 | 6.820178962 |
| 10202 | DHRS2 | -5.807354922 | -6.820178962 | 6.22881869 |
| 90809 | TMEM55B | -5.807354922 | -7.044394119 | 6.554588852 |
| 100527978 | TMEM56-RWDD3 | -5.807354922 | -7.562242424 | 6.554588852 |
| 2628 | GATM | -5.807354922 | -5.247927513 | 5.832890014 |
| 5729 | PTGDR | -5.807354922 | -7.044394119 | 6.554588852 |
| 84517 | ACTRT3 | -5.247927513 | -8.144658243 | 6.820178962 |
| 10201 | NME6 | -5.247927513 | -5.247927513 | 5.247927513 |
| 349152 | DPY19L2P2 | -5.247927513 | -6.554588852 | 6.820178962 |
| 624 | BDKRB2 | -5.247927513 | -5.832890014 | 7.562242424 |
| 11213 | IRAK3 | -5.247927513 | -6.22881869 | 7.238404739 |
| 5157 | PDGFRL | -5.247927513 | -6.22881869 | 6.22881869 |
| 169841 | ZNF169 | -5.247927513 | -5.832890014 | 5.247927513 |
| 51352 | WT1-AS | -5.247927513 | -6.22881869 | 5.247927513 |
| 387646 | LRRC37A6P | -5.247927513 | -5.832890014 | 5.247927513 |
| 154664 | ABCA13 | -5.247927513 | -7.820178962 | 7.238404739 |
| 84657 | GHRLOS2 | -5.247927513 | -6.22881869 | 5.832890014 |
| 112609 | MRAP2 | -5.247927513 | -6.22881869 | 5.247927513 |
| 120939 | C12orf59 | -5.247927513 | -5.247927513 | 6.22881869 |
| 100126793 | GHRLOS | -5.247927513 | -6.22881869 | 6.554588852 |
| 7004 | TEAD4 | -5.247927513 | -5.247927513 | 7.044394119 |
| 1368 | CPM | -5.247927513 | -6.554588852 | 5.832890014 |
| 728927 | ZNF736 | -5.247927513 | -5.832890014 | 5.832890014 |
| 1993 | ELAVL2 | -5.247927513 | -7.562242424 | 8.238404739 |
| 146434 | ZNF597 | -5.247927513 | -6.22881869 | 5.247927513 |
| 84733 | CBX2 | -5.247927513 | -5.247927513 | 5.832890014 |
| 5314 | PKHD1 | -5.247927513 | -5.247927513 | 5.832890014 |
| 3624 | INHBA | -5.247927513 | -5.832890014 | 5.247927513 |
| 114905 | C1QTNF7 | -5.247927513 | -5.832890014 | 5.247927513 |
| 3887 | KRT81 | -5.247927513 | -6.820178962 | 5.247927513 |
| 83856 | FSD1L | -5.247927513 | -7.238404739 | 5.832890014 |
| 3601 | IL15RA | -3.0694851 | -1.306661338 | 1.990477226 |
| 9711 | KIAA0226 | -3.0694851 | -2.161463423 | 2.572251449 |
| 27063 | ANKRD1 | -2.94760731 | -2.198120447 | 1.625417644 |
| 100329109 | GCSHP3 | -2.886498807 | -1.796466606 | 1.306661338 |
| 6650 | SOLH | -2.663970375 | -1.906253504 | 2.425153365 |
| 3352 | HTR1D | -2.612386978 | -2.775796151 | 2.771872482 |
| 9329 | GTF3C4 | -2.584962501 | -1.707819249 | 1.180572246 |
| 79469 | DLEU2L | -2.565853678 | -2.161463423 | 3.381429107 |
| 53354 | PANK1 | -2.481584761 | -2.142378675 | 2.852678913 |
| 7771 | ZFP112 | -2.327071398 | -1.602036014 | 2.751065791 |
| 8863 | PER3 | -2.321928095 | -2.176322773 | 1.815575429 |
| 57460 | PPM1H | -2.318855261 | -1.007653573 | 2.074767768 |
| 100131454 | DBIL5P | -2.306661338 | -1.990477226 | 1.796466606 |
| 729920 | ISPD | -2.306661338 | -1.990477226 | 3.074000581 |
| 22809 | ATF5 | -2.279697888 | -1.343471582 | 1.379889556 |
| 9076 | CLDN1 | -2.254670933 | -1.480653638 | 1.947779649 |
| 9469 | CHST3 | -2.24691474 | -1.915839552 | 1.333423734 |
| 151354 | FAM84A | -2.24691474 | -2.176322773 | 2.40053793 |
| 26145 | IRF2BP1 | -2.231564067 | -2.337303321 | 2.95419631 |
| 55765 | C1orf106 | -2.152951923 | -1.796466606 | 2.314314911 |
| 84314 | TMEM107 | -2.152951923 | -2.688710426 | 1.990477226 |
| 100303728 | SLC25A5-AS1 | -2.152951923 | -3.513623719 | 3.15721395 |
| 79862 | ZNF669 | -2.123382416 | -1.237039197 | 1.602036014 |
| 221178 | SPATA13 | -2.123382416 | -2.822001698 | 3.237039197 |
| 2318 | FLNC | -2.123382416 | -1.237039197 | 1.237039197 |
| 50964 | SOST | -1.990413951 | -2.699704587 | 2.245411038 |
| 57335 | ZNF286A | -1.980891177 | -1.572251449 | 1.796466606 |
| 158747 | MOSPD2 | -1.980891177 | -1.572251449 | 2.161463423 |
| 283417 | DPY19L2 | -1.980891177 | -1.306661338 | 2.161463423 |
| 6273 | S100A2 | -1.973394643 | -1.557543212 | 1.674772944 |
| 267004 | PGBD3 | -1.921144579 | -1.382049087 | 1.767339243 |
| 306 | ANXA3 | -1.879145605 | -1.237039197 | 1.01282404 |
| 5599 | MAPK8 | -1.879145605 | -1.602036014 | 1.602036014 |
| 54826 | GIN1 | -1.846290585 | -1.382049087 | 1.265590111 |
| 65083 | NOL6 | -1.829115441 | -1.682860144 | 2.278933785 |
| 375444 | C5orf34 | -1.822212881 | -1.068064275 | 1.243150982 |
| 79785 | RERGL | -1.811632394 | -2.206839542 | 2.078616145 |
| 100113407 | TMEM170B | -1.810100299 | -1.009586049 | 1.180572246 |
| 4312 | MMP1 | -1.810100299 | -3.562344198 | 3.365505913 |
| 22824 | HSPA4L | -1.805160709 | -1.206962381 | 1.265590111 |
| 100131691 | LOC100131691 | -1.731765836 | -2.293696279 | 2.340214042 |
| 79165 | LENG1 | -1.730567823 | -1.876788564 | 1.116458977 |
| 23221 | RHOBTB2 | -1.705256734 | -1.498805857 | 1.864933756 |
| 83541 | FAM110A | -1.701918647 | -1.333423734 | 1.464668267 |
| 790955 | C11orf83 | -1.701918647 | -1.591360272 | 2.591360272 |
| 643837 | LOC643837 | -1.695520876 | -1.504001584 | 1.415835229 |
| 11097 | NUPL2 | -1.692332598 | -2.042366031 | 2.127917475 |
| 4147 | MATN2 | -1.674229839 | -1.489805268 | 1.382049087 |
| 7584 | ZNF35 | -1.636164953 | -1.280628466 | 1.303519306 |
| 133584 | EGFLAM | -1.617505874 | -1.842194971 | 1.97928119 |
| 100170220 | SNAR-E | -1.594994654 | -2.733476608 | 1.333497484 |
| 644961 | LOC644961 | -1.593524514 | -1.237039197 | 2.431049817 |
| 8320 | EOMES | -1.572251449 | -2.688710426 | 1.306661338 |
| 3398 | ID2 | -1.559199405 | -2.868071347 | 3.157816406 |
| 27153 | ZNF777 | -1.547920799 | -1.368348775 | 1.519501724 |
| 121053 | C12orf45 | -1.53287399 | -1.003831862 | 1.540808171 |
| 5090 | PBX3 | -1.518728802 | -2.45467613 | 2.672809293 |
| 10495 | ENOX2 | -1.516873511 | -1.524006752 | 1.050273206 |
| 10186 | LHFP | -1.515979371 | -1.205853894 | 1.878648826 |
| 205717 | KIAA2018 | -1.511827796 | -1.368348775 | 1.658048537 |
| 64897 | C12orf43 | -1.497233651 | -2.224215157 | 1.94137227 |
| 89795 | NAV3 | -1.497233651 | -1.116458977 | 1.876788564 |
| 1947 | EFNB1 | -1.491591003 | -2.382738862 | 2.030148415 |
| 54619 | CCNJ | -1.484330138 | -1.590069391 | 1.489805268 |
| 7057 | THBS1 | -1.482958102 | -1.236574037 | 1.539488834 |
| 131034 | CPNE4 | -1.472564676 | -3.006388328 | 3.157541277 |
| 7516 | XRCC2 | -1.457681837 | -1.591360272 | 1.591360272 |
| 90025 | UBE3D | -1.457681837 | -1.464668267 | 1.707819249 |
| 126295 | ZNF57 | -1.457681837 | -1.707819249 | 2.093109404 |
| 284098 | PIGW | -1.457681837 | -1.464668267 | 1.591360272 |
| 9125 | RQCD1 | -1.457681837 | -2.650764559 | 1.815575429 |
| 1102 | RCBTB2 | -1.447288436 | -1.741562608 | 1.68222263 |
| 202 | AIM1 | -1.431275491 | -1.131553191 | 1.569736026 |
| 57582 | KCNT1 | -1.408639728 | -2.32447928 | 2.059404287 |
| 83449 | PMFBP1 | -1.393975884 | -2.298034889 | 1.753596269 |
| 100093630 | SNHG8 | -1.367779714 | -2.091010939 | 2.208701834 |
| 57082 | CASC5 | -1.359824703 | -1.105739254 | 1.368348775 |
| 9507 | ADAMTS4 | -1.356485317 | -1.652573407 | 2 |
| 22881 | ANKRD6 | -1.353503489 | -2.530219536 | 2.230700129 |
| 2730 | GCLM | -1.333313102 | -1.028979067 | 1.276331228 |
| 23329 | TBC1D30 | -1.328727927 | -1.106712336 | 1.35672056 |
| 92 | ACVR2A | -1.325770161 | -2.097610797 | 1.464668267 |
| 256979 | SUN3 | -1.325770161 | -1.591360272 | 1.815575429 |
| 221079 | ARL5B | -1.318855261 | -1.382049087 | 1.324994398 |
| 166793 | ZBTB49 | -1.314247358 | -1.809177658 | 1.663636815 |
| 286333 | LINC00256A | -1.306661338 | -1.572251449 | 1.796466606 |
| 467 | ATF3 | -1.306661338 | -1.572251449 | 2.314314911 |
| 152195 | NUDT16P1 | -1.306661338 | -1.572251449 | 1.572251449 |
| 378708 | APITD1 | -1.305028494 | -4.169722076 | 3.529741888 |
| 100144748 | KLLN | -1.29235498 | -1.298034889 | 1.401910065 |
| 4814 | NINJ1 | -1.287366882 | -1.760295583 | 1.99611403 |
| 92935 | MARS2 | -1.282080162 | -1.132702537 | 1.957439538 |
| 1032 | CDKN2D | -1.260474166 | -2.193603998 | 2.47883415 |
| 253635 | CCDC75 | -1.237328692 | -2.425153365 | 1.6982332 |
| 162998 | OR7D2 | -1.23725038 | -1.003480569 | 1.451171285 |
| 8516 | ITGA8 | -1.23725038 | -3.158262084 | 2.651918289 |
| 64318 | NOC3L | -1.229481846 | -1.452631787 | 1.575256683 |
| 57343 | ZNF304 | -1.221172383 | -1.478703813 | 1.082936341 |
| 54964 | C1orf56 | -1.218740027 | -1.116458977 | 1.116458977 |
| 387751 | GVINP1 | -1.218740027 | -2.27521806 | 1.809177658 |
| 3556 | IL1RAP | -1.21599465 | -1.003188278 | 1.224215157 |
| 63916 | ELMO2 | -1.204013892 | -2.600101098 | 2.297618642 |
| 388389 | CCDC103 | -1.19553894 | -1.767070609 | 1.500813964 |
| 3420 | IDH3B | -1.179652369 | -2.479125355 | 2.368873559 |
| 81282 | OR51G2 | -1.172060746 | -1.591360272 | 1.009586049 |
| 54414 | SIAE | -1.162474697 | -2.355919865 | 2.2831957 |
| 402055 | SRRD | -1.161030007 | -1.717807657 | 1.589049458 |
| 8424 | BBOX1 | -1.150434094 | -1.473931188 | 1.882997389 |
| 100129196 | LOC100129196 | -1.145739574 | -1.867745836 | 1.297297968 |
| 83448 | PUS7L | -1.142271555 | -1.18377784 | 1.341993718 |
| 100506677 | AA06 | -1.131911676 | -2.892494375 | 2.436932994 |
| 7701 | ZNF142 | -1.110558375 | -2.32447928 | 2.369645596 |
| 25939 | SAMHD1 | -1.098072123 | -1.720969194 | 1.590437631 |
| 131474 | CHCHD4 | -1.095356974 | -1.421672386 | 1.736809111 |
| 6875 | TAF4B | -1.090032201 | -1.652573407 | 1.89224382 |
| 4998 | ORC1 | -1.085097591 | -1.55378562 | 1.443964685 |
| 642361 | LOC642361 | -1.084590172 | -1.128605817 | 1.066733989 |
| 23166 | STAB1 | -1.07368113 | -2.037570254 | 1.560624721 |
| 340515 | LOC340515 | -1.072332726 | -2.753497756 | 2.574646539 |
| 10436 | EMG1 | -1.070278696 | -1.013872175 | 1.002901057 |
| 55113 | XKR8 | -1.04817576 | -1.716083553 | 1.45736472 |
| 4701 | NDUFA7 | -1.01282404 | -1.431049817 | 1.431049817 |
