# Supplementary material for: NIDO, AMOP and vWD domains of MUC4 play synergic role in MUC4 mediated signaling
Source: Oncotarget. 2017 Jan 2;8(6):10385–99. doi: 10.18632/oncotarget.14420 (PMC5354666; doi:10.18632/oncotarget.14420)
Supplement: Supplementary file 3 [file oncotarget-08-10385-s003.docx]

**Additional file 3(Table S5-10): Sequence-based global annotation of GO function and the KEGG pathway analysis illustrate the universality and individuality of the role of these three unique domains.**

**Table S5. Functional categories of common differentially expressed genes (DEGs) in PANC-1 cells of overexpressing MUC4/Y-N^△^ as compared with PANC-1 cells of overexpressing MUC4/Y. DEGs were annotated according to the indicated Gene Ontology system. *P*-values indicate the statistical significance of the enrichment of these terms as calculated by Fisher’s exact test and corrected for multiple testing using the Bonferroni method, *P* ≤ 0.05.**

| **Subdirectory of GO** | **Accession** | **Gene Ontology term** | **Cluster frequency** | **Corrected *P*-value** | **General description** |
| --- | --- | --- | --- | --- | --- |
| Cellular Component | GO:0042995 | cell projection | 60 out of 688 genes, 8.7% | 0.03743 | A prolongation or process extending from a cell, e.g. a flagellum or axon. |
|  | GO: 0008021 | synaptic vesicle | 9 out of 688 genes, 1.3% | 0.00213 | A secretory organelle, typically 50 nm in diameter, of presynaptic nerve terminals; accumulates in high concentrations of neurotransmitters and secretes these into the synaptic cleft by fusion with the 'active zone' of the presynaptic plasma membrane. |
|  |  |  |  |  |  |

**Table S6. Functional categories of common differentially expressed genes (DEGs) in PANC-1 cells of overexpressing MUC4/Y-A^△^ as compared with PANC-1 cells of overexpressing MUC4/Y. DEGs were annotated according to the indicated Gene Ontology system. *P*-values indicate the statistical significance of the enrichment of these terms as calculated by Fisher’s exact test and corrected for multiple testing using the Bonferroni method, *P* ≤ 0.05.**

| **Subdirectory of GO** | **Accession** | **Gene Ontology term** | **Cluster frequency** | **Corrected *P*-value** | **General description** |
| --- | --- | --- | --- | --- | --- |
| Cellular Component | GO:0042995 | cell projection | 75 out of 750 genes, 10.0% | 4.74e-05 | A prolongation or process extending from a cell, e.g. a flagellum or axon. |
|  | GO:0016020 | membrane | 387 out of 750 genes, 51.6% | 0.00100 | Double layer of lipid molecules that encloses all cells, and, in eukaryotes, many organelles; may be a single or double lipid bilayer; also includes associated proteins. |
|  | GO:0044425 | membrane part | 329 out of 750 genes, 43.9% | 0.00207 | Any constituent part of a membrane, a double layer of lipid molecules that encloses all cells, and, in eukaryotes, many organelles; may be a single or double lipid bilayer; also includes associated proteins. |
|  | GO:0031224 | intrinsic to membrane | 292 out of 750 genes, 38.9% | 0.00385 | Located in a membrane such that some covalently attached portion of the gene product, for example part of a peptide sequence or some other covalently attached group such as a GPI anchor, spans or is embedded in one or both leaflets of the membrane. |
|  | GO:0005576 | extracellular region | 80 out of 750 genes, 10.7% | 0.01009 | The space external to the outermost structure of a cell |
|  | GO:0044421 | extracellular region part | 78 out of 750 genes, 10.4% | 0.01542 | The space external to the outermost structure of a cell |
|  | GO:004300 | neuron projection | 60 out of 1177 genes, 5.1% | 0.00213 | A prolongation or process extending from a nerve cell, e.g. an axon or dendrite. |
| Biological Process | GO:0023052 | signaling | 227 out of 667 genes, 34.0% | 0.02800 | The entirety of a process in which information is transmitted within a biological system. This process begins with an active signal and ends when a cellular response has been triggered. |
|  | GO:0023060 | signal transmission | 174 out of 667 genes, 26.1% | 0.04822 | The process in which a signal is released and/or conveyed from one location to another. |
|  |  |  |  |  |  |

**Table S7. Functional categories of common differentially expressed genes (DEGs) in PANC-1 cells of overexpressing MUC4/Y-V^△^ as compared with PANC-1 cells of overexpressing MUC4/Y. DEGs were annotated according to the indicated Gene Ontology system. *P*-values indicate the statistical significance of the enrichment of these terms as calculated by Fisher’s exact test and corrected for multiple testing using the Bonferroni method, *P* ≤ 0.05.**

| **Subdirectory of GO** | **Accession** | **Gene Ontology term** | **Cluster frequency** | **Corrected *P*-value** | **General description** |
| --- | --- | --- | --- | --- | --- |
| Cellular Component | GO:0042995 | cell projection | 86 out of 790 genes, 10.9% | 1.08e-07 | A prolongation or process extending from a cell, e.g. a flagellum or axon. |
|  | GO:004300 | neuron projection | 50 out of 790 genes, 6.3% | 2.56e-05 | A prolongation or process extending from a nerve cell, e.g. an axon or dendrite. |
|  | GO:0016020 | membrane | 413 out of 790 genes, 52.3% | 8.63e-05 | Double layer of lipid molecules that encloses all cells, and, in eukaryotes, many organelles; may be a single or double lipid bilayer; also includes associated proteins. |
|  | GO:0044425 | membrane part | 349 out of 790 genes, 44.2% | 0.00054 | Any constituent part of a membrane, a double layer of lipid molecules that encloses all cells, and, in eukaryotes, many organelles; may be a single or double lipid bilayer; also includes associated proteins. |
|  | GO:0031224 | intrinsic to membrane | 310 out of 790 genes, 39.2% | 0.00105 | Located in a membrane such that some covalently attached portion of the gene product, for example part of a peptide sequence or some other covalently attached group such as a GPI anchor, spans or is embedded in one or both leaflets of the membrane. |
|  | GO:0005576 | extracellular region | 81 out of 790 genes, 10.3% | 0.03525 | The space external to the outermost structure of a cell |
| Biological Process | GO:0023052 | signaling | 257 out of 718 genes, 35.8% | 8.42e-05 | The entirety of a process in which information is transmitted within a biological system. This process begins with an active signal and ends when a cellular response has been triggered. |
|  | GO:0023060 | signal transmission | 199 out of 718 genes, 27.7% | 0.00016 | The process in which a signal is released and/or conveyed from one location to another. |
|  | GO:0023052 | signaling process | 199 out of 718 genes, 27.7% | 0.00020 | The entirety of a process in which information is transmitted within a biological system. This process begins with an active signal and ends when a cellular response has been triggered. |
|  | GO:0022008 | neurogenesis | 50 out of 718 genes, 7.0% | 0.00121 | Generation of cells within the nervous system. |
|  | GO:0048699 | generation of neurons | 45 out of 718 genes, 6.3% | 0.00659 | The process in which nerve cells are generated. This includes the production of neuroblasts and their differentiation into neurons. |
|  | GO:0007399 | nervous system development | 81 out of 718 genes, 11.3% | 0.00860 | The process whose specific outcome is the progression of nervous tissue over time, from its formation to its mature state. |
|  | GO:0030182 | neuron differentiation | 35 out of 718 genes, 4.9% | 0.01776 | The process in which a relatively unspecialized cell acquires specialized features of a neuron. |
|  | GO:2000026 | regulation of multicellular organismal development | 43 out of 718 genes, 6.0% | 0.01919 | Any process that modulates the frequency, rate or extent of multicellular organismal development. |
|  | GO:0007165 | signal transduction | 94 out of 718 genes, 13.1% | 0.02644 | The cellular process in which a signal is conveyed to trigger a change in the activity or state of a cell. Signal transduction begins with reception of a signal (e.g. a ligand binding to a receptor or receptor activation by a stimulus such as light), or for signal transduction in the absence of ligand, signal-withdrawal or the activity of a constitutively active receptor. Signal transduction ends with regulation of a downstream cellular process, e.g. regulation of transcription or regulation of a metabolic process. Signal transduction covers signaling from receptors located on the surface of the cell and signaling via molecules located within the cell. For signaling between cells, signal transduction is restricted to events at and within the receiving cell. |
|  |  |  |  |  |  |

**Table S8. Functional categories of common differentially expressed genes (DEGs) in PANC-1 cells of overexpressing MUC4/Y-NAV^△^ as compared with PANC-1 cells of overexpressing MUC4/Y. DEGs were annotated according to the indicated Gene Ontology system. *P*-values indicate the statistical significance of the enrichment of these terms as calculated by Fisher’s exact test and corrected for multiple testing using the Bonferroni method, *P* ≤ 0.05.**

| **Subdirectory of GO** | **Accession** | **Gene Ontology term** | **Cluster frequency** | **Corrected *P*-value** | **General description** |
| --- | --- | --- | --- | --- | --- |
| Cellular Component | GO:0042995 | cell projection | 84 out of 915 genes, 9.2% | 0.00033 | A prolongation or process extending from a cell, e.g. a flagellum or axon. |
|  | GO:0016020 | membrane | 464 out of 915 genes, 50.7% | 0.00138 | Double layer of lipid molecules that encloses all cells, and, in eukaryotes, many organelles; may be a single or double lipid bilayer; also includes associated proteins. |
|  | GO:0031224 | intrinsic to membrane | 349 out of 915 genes, 38.1% | 0.00502 | Located in a membrane such that some covalently attached portion of the gene product, for example part of a peptide sequence or some other covalently attached group such as a GPI anchor, spans or is embedded in one or both leaflets of the membrane. |
|  | GO:0044425 | membrane part | 387 out of 915 genes, 42.3% | 0.01920 | Any constituent part of a membrane, a double layer of lipid molecules that encloses all cells, and, in eukaryotes, many organelles; may be a single or double lipid bilayer; also includes associated proteins. |
|  | GO:0016021 | integral to membrane | 113 out of 915 genes, 12.3% | 0.04597 | Penetrating at least one phospholipid bilayer of a membrane. May also refer to the state of being buried in the bilayer with no exposure outside the bilayer. When used to describe a protein, indicates that all or part of the peptide sequence is embedded in the membrane. |
| Molecular Function | GO:0030695 | GTPase regulator activity | 38 out of 874 genes, 4.3% | 0.04792 | Modulates the rate of GTP hydrolysis by a GTPase. |
| Biological Process | GO:0023052 | signaling | 299 out of 828 genes, 36.1% | 2.52e-06 | The entirety of a process in which information is transmitted within a biological system. This process begins with an active signal and ends when a cellular response has been triggered. |
|  | GO:0023060 | signal transmission | 228 out of 828 genes, 27.5% | 3.68e-05 | The process in which a signal is released and/or conveyed from one location to another. |
|  | GO:0023052 | signaling process | 228 out of 828 genes, 27.5% | 4.65e-05 | The entirety of a process in which information is transmitted within a biological system. This process begins with an active signal and ends when a cellular response has been triggered. |
|  |  |  |  |  |  |

**Table S9. Representative KEGG pathways from signaling pathway impact analysis of DEGs in PANC-1 cells of overexpressing MUC4/Y-V^△^ as compared with PANC-1 cells of overexpressing MUC4/Y. DEGs were annotated with the indicated KEGG database. *P*-values were FDR-corrected for multiple testing, *P≤0.05*. Status predictions were obtained by signaling pathway impact analysis[50]taking fold-change estimates and pathway topology into account.**

| **Pathway ID** | **Pathway** | **DEGs with pathway annotation (835)** | **Corrected *P-* value** | **Predicted status in PANC-1-MUC4/Y-N^△^ cell** | **Class** |
| --- | --- | --- | --- | --- | --- |
| ko04010 | MAPK signaling pathway | 41 (4.91%) | 0.01527118 | Inactivated | Environmental Information Processing; Signal transduction |
| ko04610 | Complement and coagulation cascades | 21 (2.51%) | 0.03150312 | Inactivated | Organismal Systems; Immune system |
| ko04062 | Chemokine signaling pathway | 31 (3.71%) | 0.03150312 | Inactivated | Organismal Systems; Immune system |
| ko04020 | Calcium signaling pathway | 27 (3.23%) | 0.04294926 | Inactivated | Environmental Information Processing; Signal transduction |
| ko04060 | Cytokine-cytokine receptor interaction | 29 (3.47%) | 0.04324608 | Inactivated | Environmental Information Processing; Signaling molecules and interaction |

**Table S10. Representative KEGG pathways from signaling pathway impact analysis of DEGs in PANC-1 cells of overexpressing MUC4/Y-NAV^△^ as compared with PANC-1 cells of overexpressing MUC4/Y. DEGs were annotated with the indicated KEGG database. *P*-values were FDR-corrected for multiple testing, *P≤0.05*. Status predictions were obtained by signaling pathway impact analysis[**[**46**](#_ENREF_46)**], taking fold-change estimates and pathway topology into account.**

| **Pathway ID** | **Pathway** | **DEGs with pathway annotation (965)** | **Corrected *P-* value** | **Predicted status in PANC-1-MUC4/Y-N^△^ cell** | **Class** |
| --- | --- | --- | --- | --- | --- |
| ko04010 | MAPK signaling pathway | 50 (5.18%) | 0.000554462 | Inactivated | Environmental Information Processing; Signal transduction |
